# Revision of the *Exechiaparva* group (Diptera: Mycetophilidae)

**DOI:** 10.3897/BDJ.9.e67134

**Published:** 2021-09-24

**Authors:** Jon Peder Lindemann, Geir Søli, Jostein Kjærandsen

**Affiliations:** 1 UiT – The Arctic University of Norway, Tromsø, Norway UiT – The Arctic University of Norway Tromsø Norway; 2 Natural History Museum, Oslo, Norway Natural History Museum Oslo Norway

**Keywords:** fungus gnats, *
Exechia
*, parva, repanda, taxonomy, DNA barcodes

## Abstract

**Background:**

*Exechia* is a diverse genus of small fungus gnats, widespread in the Holarctic Region, while the fauna is largely unknown elsewhere, such as in the Afrotropical and Oriental Region. Members of *Exechia* can be arranged into several species groups, based on homologies in the male and female terminalia. The *Exechiaparva* group is delimited, based on male terminalia possessing a pair of gonocoxal lobes on the apicoventral gonocoxal margin. Eight previously-described species can be placed in this group, of which six are from the Holarctic Region, while one is recorded each from the Oriental and the Afrotropical Regions.

**New information:**

The *Exechiaparva* group was reviewed and found to include 33 species, of which 24 were described as new to science and six were re-described. Identification keys to 32 species for males and nine species for females are provided together with illustrations and photos of male and female terminalia. Species delimitations were based on morphological examination of 94 male and female specimens, as well as DNA barcodes obtained from 124 specimens. Molecular and morphological species delimitations were mostly congruent, except in two cases where two species were delimited within a single Barcode Index Number (BIN). We found that each species is only known from a single zoogeographical region and that several species complexes are largely congruent with zoogeographical divisions, indicating that intercontinental barriers may have a strong impact on the species diversity of the group.

## Introduction

The genus *Exechia* Winnertz, 1863 is known from all zoogeographical regions, except the Neotropical Region and Antarctica (Bechev 1999). With 158 valid species (Burdíková et al. 2019), it is the largest amongst 20 genera of the tribe Exechiini, where it is considered to have diverged as one of the most apical lineages (Burdíková et al. 2019, Rindal et al. 2007). The larval development takes place in fruit-bodies of a wide range of macrofungi (Ševčík 2006, Jakovlev 2011, Chandler 2010, Jakovlev 1994, Hackman and Meinander 1979). Like most other members of the subfamily Mycetophilinae, they hibernate as adults. Different species have been reported hibernating in caves (Kjærandsen 1993, Kurina 1996, Hedmark 2000) or under bark of conifers (Hedmark 2000). At least two species of the group in question, *E.parva* Lundström, 1909 and *E.repanda* Johannsen, 1912 [*E.neorepanda* sp. n. as revised in this article], are reported to hibernate in hollow, usually broken umbelliferous stems (Fig. 1c, Plassmann 1971, Väisänen 1981, Kurina 1997). Species of *Exechia* seem to be well adapted to the low temperatures through high freeze tolerance; it is demonstrated that *E.nugatoria* can survive temperatures down to -32^o^C (Sformo et al. 2009).

While most of the species have been described from the Holarctic Region, where the collecting effort traditionally has been greatest, it is likely that a large part of the fauna from other regions still remains to be described. It is estimated that two thirds of the Afrotropical Diptera diversity is undescribed and Mycetophilidae is considered to be one of the lesser known Diptera families of the Region (Kirk-Spriggs and Stuckenberg 2010). Only ten species of *Exechia* have been described from the Afrotropical Region (Søli 2017) and 18 from the Oriental Region, but several undescribed species are mentioned in literature (Edwards 1926, Matile 1978). Nine species have been described from the Australian Region (Skuse 1888, Tonnoir and Edwards 1927). The majority of species in the Holarctic is considered to have a circumpolar distribution, with 48 out of 62 Palaearctic species also recorded from the Nearctic Region (Bechev 1999). The Nearctic fauna have received little attention the last decades (Shaw and Fisher 1952, Laffoon 1965), while the Palaearctic fauna have been treated in several recent regional surveys (Zaitzev 2003, Gammelmo and Søli 2006, Kjærandsen et al. 2007, Jakovlev 2014, Kjærandsen 2017a, Kjærandsen and Søli 2020).

Species identification is mainly based on comparison of the male or female terminalia. Some identification keys have been published (Shaw and Fisher 1952, Zaitzev 2003), but these are limited to species of certain geographical regions and of limited use when treating the total fauna. Females are often more difficult to determine due to few described and illustrated morphological characters, although many of them seemingly have characteristic and species-specific features in their terminalia.

A few species groups in *Exechia* have been sorted out and characterised. Tuomikoski (1966) recognised a distinct group composed of *Exechiaseriata* (Meigen 1830) and several non-European species. Based on female specimens, Chandler (1977) recognised two groups, the *Exechiafusca* group comprised of *E.fusca* (Meigen, 1804) and *E.confinis* Winnertz, 1864 and the *E.contaminata* group comprised of *E.contaminata* Winnertz, 1864, *Exechianigroscutellata* Landrock, 1912 and *E.pseudocincta* Strobl, 1910. Kallweit and Martens (1995) recognised, but did not name, a species group comprised of *E.parva*, *E.repanda*, *E.repandoides* Caspers, 1984 and *E.pararepanda* Kallweit, 1995. The Nearctic species, *E.capillata* Johannsen, 1912, can also be added to this group as the close relationship between *E.parva* and *E.capillata* has been known for a long time (Barendrecht 1938). The morphological differences between *E.repanda*, *E.parva* and *E.repandoides* have also been treated in detail by Kurina (1997). Chandler (2000) argued that *E.adenaparva* Chandler, 2000, together with *E.cinctiformis* Storå, 1941, belongs to this group, referring to the "*Exechiaparva* group" and that the group is characterised by “a pair of elongate setose processes on the apicoventral margin of the gonocoxites”. Based on this definition, we can also add *E.rohdendorfi* Zaitzev, 1996 to the group.

In this study, we will revise the *Exechiaparva* group, which involves descriptions of 24 new species and re-descriptions of six species. We apply an integrative approach, based on morphological and molecular delimitation methods, to revise the species and identify species boundaries.

## Materials and methods

### Insect material

The study is based on material from North-Western Europe, North America, East Africa, South Africa, Madagascar, Japan, Nepal and Bhutan, obtained from several different insect collections. The material was collected in the time span between 1971 and 2019, mostly by sweep net and malaise traps. Material from Nepal was collected by the Kyushu University Expedition to the Himalayas in 1971 and 1972 and by U. Emoto during an expedition to East Nepal in 1981. Material from Bhutan was collected by T. Saigusa during his collecting trip to Bhutan in 1993. Afrotropical material has partly been received from M. Mostovski and M. Jaschhof, collected during their travel in South Africa in 2005 and from one of the authors, J. Kjærandsen, as well as a loan from National Museum, Bloemfontein to the Natural History Museum in Oslo.

Specimens from the following collections have been examined:

- **BMSA** National Museum - Bloemfontein, South Africa
- **CBG** Center for Biodiversity Genomics, Canada
- **CUIC** The Cornell University Insect Collection, Ithaca, New York, USA
- **KUEC** Kyushu University Museum, Japan
- **MZHF** Zoological Museum, Helsinki, Finland
- **NHM** Natural History Museum, London, United Kingdom
- **TMU** Tromsø University Museum, Norway
- **ZMM** Zoological Museum - Moscow State University, Russia
- **ZSMC** Zoologische Staatssammlung München, Germany

### DNA barcodes and molecular species delimitation

For molecular species delimitation, the mitochondrial gene, cytochrome oxidase subunit 1 (CO1), was used as the DNA barcode. Legs from selected individuals were sent to the Canadian Center for DNA Barcoding (CCDB) in Guelph for subsequent DNA extraction, amplification and sequencing. DNA amplification was performed with the primer pair C_LepFoIF and C_LepFoIR (Hernández‐Triana et al. 2014). Specimens older than 1990 were not sequenced. In some cases, the full length regions were not obtained, possibly due to fragmentation caused by aging or poor preservation. Material retrieved from CBG had previously been sequenced through different projects and the DNA barcodes were already accessible in BOLD Systems (Ratnasingham and Hebert 2007). The dataset, consisting of 124 DNA barcodes, are available in BOLD under the dataset DS-REPGR (dx.doi.org/10.5883/DS-REPGR).

Molecular species delimitation was mainly based on the Barcode Index Number (BIN) system (Ratnasingham and Hebert 2013), dividing DNA barcodes into BINs. Intraspecific and interspecific genetic distances were calculated in R (R Core Team 2020) with the packages ape (Paradis and Schliep 2019) and vegan (Oksanen et al. 2019). Additionally, a Bayesian analysis of the CO1 sequence data was carried out in BEAST v.2.5.1 (Bouckaert et al. 2019). Sequences representing six outgroup taxa were downloaded from BOLD Systems and added to the dataset. These taxa include four species within *Exechia, E. cornuta* Lundström, 1914, *E.cincta* Winnertz, 1863, *E.fusca* (Meigen, 1804) and *E.separata* Lundström, 1912 and two within Exechiini, *Allodialugens* (Wiedemann, 1817) and *Exechiopsissubulata* (Winnertz, 1863). Sequence alignment was performed in MAFFT v.7.310 with "linsi" settings (Katoh et al. 2005). The BEAST input xml-file was generated in BEAUTI v.2.5.1 (Bouckaert et al. 2019) with a HKY substitution model, Yule tree prior and a strict clock. Two chains were run for 10 mil. MCMC generations, sampling trees every 5000 generations. The convergence of the runs were evaluated by examining the effective sample size (ESS) values in Tracer v.1.7.1 (Rambaut et al. 2018). The two chains were combined in LogCombiner with a 10% burn-in (Bouckaert et al. 2019). A maximum clade credibility tree was generated from the sampled trees using Tree Annotator v.2.5.1 (Bouckaert et al. 2019) and visualised in R with the ggtree package (Yu et al. 2017). The R scripts are available from GitHub (github.com) under the repository, The-Exechia-Parva-Group (zenodo.org/badge/latestdoi/321617679) and in Suppl. material 3.

### Morphological examination

Male and female terminalia were dissected from specimens and heated in lactic acid to remove soft tissue. Temporary slide mounts were made using glycerol as medium, so that the terminalia later could be stored on microvials in glycerol, together with the pinned specimens. For most male specimens, the gonostyli were dissected. Slides were photographed using a Zeiss Axio imager M2 microscope, together with an Axiocam 506 colour camera. Stacked images were rendered in the image-stacking software Helicon Focus 7, sharpened in Topaz Sharpen AI and post-edited in Adobe Photoshop CS6. Illustrations were made in Adobe Illustrator.

### Terminology

The morphological terminology follows Søli (1997) and Søli et al. (2000). In addition, we have adopted the terminology of Kjærandsen (2006) regarding the division of the male gonostylus into a "dorsal branch", "dorsointernal branch", "medial branch", "ventral branch", "anterior branch" and "internal branch". In the *Exexhiaparva* group, the dorso-internal branch and the anterior branch are reduced or inconspicuous and these terms have thus been omitted in the descriptions.

The following abbreviations will be used throughout the text:

- Dorsal gonostylus branch = **DB**
- Gonocoxal lobe = **GL**
- Internal gonostylus branch = **IB**
- Medial gonostylus branch = **MB**
- Ventral gonostylus branch = **VB**

Terminology used to describe male and female terminalia is shown in Figs 2, 3.

### The *Exechiaparva* group

**Description**:

**Colouration**. Pale to dark brown individuals. Head usually darker than scutum. Palpus, scape, pedicel and basal part of first flagellomere usually paler than rest of head and antennae. Scutum uniformly coloured, sometimes with pale lateral and frontal margins; mesonotal stripes rarely present. Legs usually whitish-yellow. Halteres usually whitish-yellow, apically sometimes slightly darker. Wings usually hyaline with yellow or pale brown tint, rarely with weak dark marks. Abdomen pale brown to dark brown, sometimes tergites I-III slightly paler or with lateral pale area. Female tergites II-VI usually with lateral and/or dorsolateral pale areas, at the anterior margin of the tergites. Terminalia yellow to brown. **Head**. Two ocelli, touching margins of compound eyes. Vertex with row of 6 orbital bristles along each eye margin; 12 frontal bristles. Frons and vertex covered with short, usually pale setae, extending from level of frontal bristles to occiput. Clypeus covered with thin pale setae, usually most dense on ventral part. Antennae with 14 flagellomeres, covered with fine setae. **Thorax**. Scutum with strong pre-alar and postalar bristles and three rows of discal bristles. Scutellum with one pair of strong bristles, usually curving anteriorly. Bristles on scutum and scutellum slightly forked apically, with one branch shorter than the other (as illustrated in Magnussen 2020). Scutum and scutellum covered with short setae, usually pale, but sometimes darker. Antepronotum with three bristles, the dorsal-most usually shorter and sometimes pale, the remaining two stronger, dark, curved anteriorly; anterodorsally with few short setae. Proepisternum with two strong bristles. Laterotergite centrally with several elongate setae. Other lateral tergites bare. **Legs**. Tibiae covered with setae arranged in rows. Mid-tibia with rows of posterodorsal, anterodorsal, anterior and anteroventral bristles. Hind tibia with rows of posterodorsal and anterodorsal bristles; apical sixth with row of anterior bristles. **Abdomen**. Covered with pale to dark brown setae. **Male terminalia.** Tergite IX medially divided, covered with microtrichia; each part tapered towards more or less pointed apex, with 2-12 setae, 1-2 most apical setae elongate. Cerci usually not reaching longer than level of gonostylus; each part more or less kidney-shaped; evenly covered with microtrichia; covered with setae mainly on the dorsal and internal surface. Gonocoxites, evenly covered with setae, except on basoventral half or two thirds, with more elongate setae towards apicoventral margins; apicoventrally with pair of usually elongated gonocoxal lobes, each usually with tapered basal part and apical part with lateral margins extended more or less parallel; each GL apically with 2-5 usually elongate setae. One pair of aedaegal guides, usually visible as transparent, more or less elongate structures, protruding from below apicoventral margins of gonocoxites, between the hypandrium and the GL. Hypandrium (= sternite IX) present as weakly-delimited sclerite at the apicoventral gonocoxal margin, fused with the gonocoxites, small and rounded, covered with 5-30 smaller setae, apically with two strong elongated setae. Hypandrial lobe extending from below or adjacent to the hypandrium; deeply bifurcated with branches diverging in 180-140 degree angle; each branch elongated and usually slender, extending exteriorly and usually curving strongly distally. Gonostylus with DB of variable size and shape; more or less covered with setae on dorsal side. VB usually short and rounded, hardly noticeable in ventral view; with 1-4 setae, one or more setae is usually located at or close to the apex. IB with most basal part wide and rounded consisting of two hyaline lobes, the largest wrinkled with small process or flap with 2 small setae; the remaining part elongate and slender with 3-6 setae, one located close to the base, and at least one located at or close to the apex, while remaining setae usually arranged in pair or row somewhere on apical half. MB of variable size and shape, usually with 1 seta located on basal half. **Female terminalia**: Tergite VIII in dorsal view with apical margin more or less emarginate; evenly covered with setae. Tergite IX short, mostly hidden within tergite VIII; apical margin distinctly emarginate, medial part fused with gonocoxite IX; without setae. Tergite X reduced. Cerci two-segmented; apical segment two-thirds to half as long as basal segment; in lateral view, basal segment at most 1.7 times broader than apical segment; evenly covered with small setae, except on ventral side of basal segments. Sternite VIII about 1.2 times longer than broad, usually with lateral margins distinctly rounded; evenly covered with setae on apical half; hypogynal valves broad, apicoventrally emarginate, truncate or obtuse, apicolateral corner forming short lobe apically with large, somewhat curved setae; hypogynal valves separated by more or less deep cleft, one-third to one-seventh as deep as entire segment length. Labia hyaline; apical part exceeding beyond apex of hypogynium, in ventral view about as broad as long, slightly acuminate or evenly tapered, acute apex. Gonapophysis VIII reduced or fused with gonapophysis IX. Gonapophysis IX elongate, extended beyond apex of cerci; apical part narrow, in ventral and dorsal view usually slightly spathulate; apically with 2-8 minute setae; ventral side sometimes with distinct sclerotisation around opening of spermathecal duct. Sternite X reduced or fused with gonapophysis IX. Spermathecal duct extended half to three-fourths of gonapophysis IX length.

**Diagnosis**:

**Males**. Distinguished from species in the *E.cincta* group in having the hypandrial lobe with branches diverging in a wide angle (Fig. 2a); from other species in the genus, in having the gonocoxites apicoventrally with pair of usually elongated lobes (gonocoxal lobes), each lobe apically with 2-5, usually elongate setae (Fig. 2a); in addition, the hypandrium usually covered with 5-30 smaller setae, apically with two stronger and more elongate setae (Fig. 2a).

**Females**. Distinguished from species in the *E.fusca* group in having the hypogynal valves apicoventrally emarginate, truncate or obtuse (Fig. 3b), not evenly tapered towards an acute apex as in *E.confinis*; however, with apicolateral margin forming a short lobe (Fig. 3), in combination with gonapophysis IX reaching beyond cerci (Fig. 3a); from *E.dorsalis* (Staeger, 1840) in having the tergite VIII with apicolateral corner evenly rounded and inconspicuous (Fig. 3a); from other species in the genus by having the cerci two segmented, apical segment two-thirds to half as long as basal segment, in lateral view basal segment at most 1.7 times broader than apical segment (Fig. 3a).

## Taxon treatments

### Exechia adenaparva

Chandler, 2000

9D488837-6572-56B1-B528-8C41052A4A57

Exechia adenaparva Chandler, 2000: 282 ♂ (Chandler 2000)

#### Distribution

**Figure 4.**
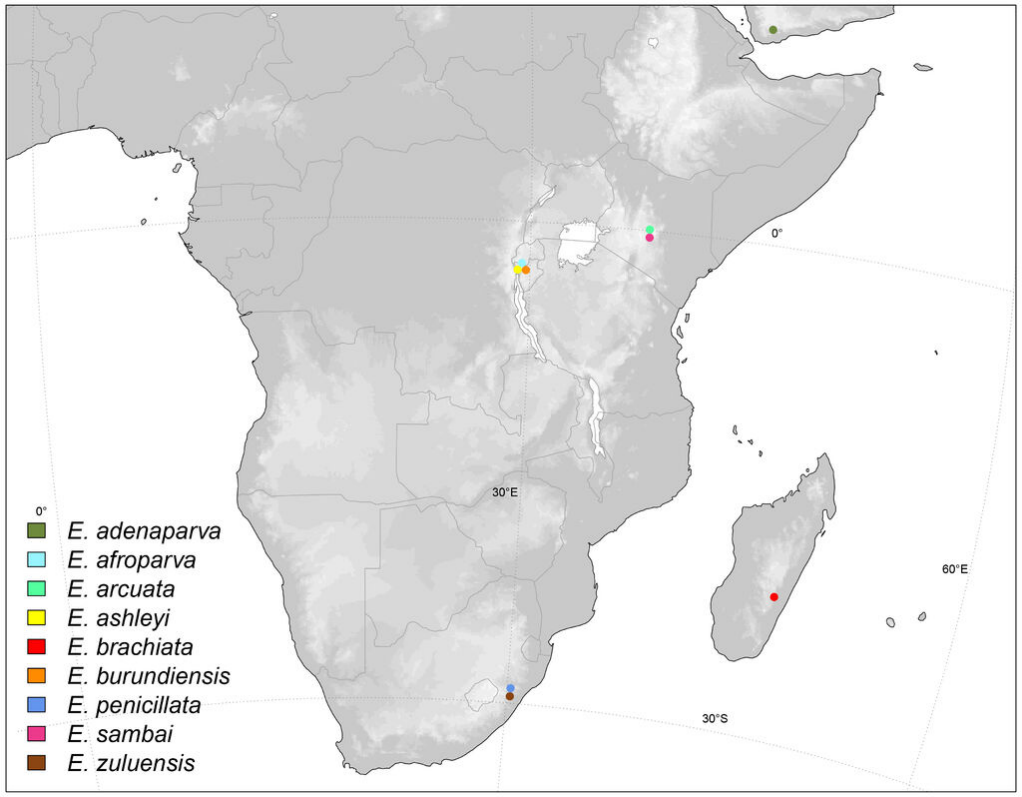
Distribution map of species in the *E.parva* group occurring in the Afrotropical Region, based on localities from studied material, type material and DNA barcoded material. Different species are represented by individual colours. Altitudes are indicated in shades from grey (lowland) to white (mountains).

Afrotropical, Yemen (Fig. 4).

#### Notes

We did not have the opportunity to re-describe this species, but the male terminalia are well illustrated in Chandler (2000) (Fig. 5a, b). Dr. Erica McAlister, NHM, provided us with images of the holotype (Fig. 5c, d), which seems to be in good condition. The holotype is pinned with the abdomen and terminalia dissected and slide-mounted, with five labels: "W. ADEN. PROT. / Wadi Dareija, / S.W of Dahla, / ea. 4500 ft. / 6-9.xi.1937", "B.M. Exp. to / S.W. Arabia. / H. Scott & / E.B. Britton. / B.M. 1938-246", "Exechia / ♂ adenaparva spn / det. P. J. Chandler / Holotype", "BMNH(E) # / 254408", "NHMUK 012804625".

### Exechia afroparva

Lindemann sp. n.

91F7E7D4-5DC3-5D7F-8BA0-FA3AB2228861

urn:lsid:zoobank.org:act:061df718-a50b-418c-9566-58d26ebe4c73

#### Materials

- **Type status:**Holotype. **Occurrence:** catalogNumber: TSZD-JKJ-107225; occurrenceRemarks: BMSA(D): 26650; recordedBy: A.H. Kirk-Spriggs; individualCount: 1; sex: male; lifeStage: adult; preparations: Pinned, with genitalia in glycerine in separate microvial; **Location:** country: Burundi; stateProvince: Kayanza prov.; municipality: Rwegara; locality: Parc National de la Kibira; verbatimElevation: 2237 m; decimalLatitude: -2.922000; decimalLongitude: 29.501117; **Event:** samplingProtocol: Malaise trap; eventDate: 2010-11-25/2010-11-26; habitat: Indigenous Afromontane forest; **Record Level:** institutionCode: BMSA

#### Description

Male: Body length 5.2 mm. Wing length 4.0 mm. **Colouration** (Dry specimen). Head dark brown; eye margin, face and clypeus yellow; labellum yellow; palpus yellow-brown, segments 4-5 dark brown. Antennae with scape and pedicel yellow; flagellum brown, first flagellomere with yellow base. Thorax with scutum brown, pale mesonotal stripes present and anterior and lateral margins broadly yellow; lateral sclerites pale brown to dark brown; propleura brown; halteres whitish-yellow, apically slightly darker. Legs whitish-yellow. Abdomen dark brown, tergites I-III with yellow lateral area. Terminalia pale brown. **Head**. Frons and vertex covered with short black setae. Clypeus covered with short black setae, evenly distributed; flagellomeres longer than broad, with sixth flagellomere 1.2 times as long as wide. **Thorax**. Scutum covered with short black setae. **Legs**. Mid-tibia with 23 anterior, 9 posterodorsal, 10 posterior and 6 posteroventral bristles. Hind tibia with 14 anterodorsal, 8 posterodorsal and 8 posterior bristles. **Abdomen.** Tergites covered with black setae. **Terminalia** (Fig. 6). Each part of divided tergite IX apically with about 7 setae, apical seta stout. Gonocoxites evenly covered with setae, except on basoventral third (Fig. 6a, b). Each GL evenly tapered towards apex, entirely covered with setae, apex with 2 setae (Fig. 6a, b). Aedaegal guides short, evenly tapered towards rounded apex (Fig. 6a, b). Hypandrium covered with about 11 setae, apical pair of setae reaching about level of GL apex (Fig. 6a, b). Hypandrial lobe elongate, each branch basally with large hyaline flap, apical part tapering towards narrow rounded apex (Fig. 6a). Gonostylus (Fig. 6c) with DB elongate, apically spathulate with apicoexternal margin slightly expanded, dorsally evenly covered with setae, except on apical third. VB with apical half acuminate, apex acute; with 3 setae, apical seta on apex. IB elongate, expanded area one-third from apex, apically capitate, apex acute, row of 4 setae close to apex. MB elongate, apex rounded, apically with 2 small setae, basal seta close to centre.

Female unknown.

#### Diagnosis

Distinguished from all species in the *E.parva* group in having the gonocoxal lobes entirely covered with setae and entire length tapering (Fig. 6a), in combination with the internal gonostylus branch with 4 setae close to the apex (Fig. 6c).

#### Etymology

From *afro*, relating to the Afrotropical Region and *parva*, relating to the resemblance to *E.parva*.

#### Distribution

Afrotropical, Burundi (Fig. 4).

#### Biology

Adult collected in afromontane forest (2237 m a.s.l.).

### Exechia arcuata

Lindemann sp. n.

640977E0-9902-5931-9423-CF5FE3AD0129

urn:lsid:zoobank.org:act:3d22f6c7-e233-48d1-9da3-19a0af7f6add

http://dx.doi.org/10.5883/BOLD:AEA5210

#### Materials

- **Type status:**Holotype. **Occurrence:** catalogNumber: TSZD-JKJ-107175; recordedBy: J. Kjærandsen; individualCount: 1; sex: male; lifeStage: adult; preparations: Pinned, with genitalia in glycerine in separate microvial; **Location:** country: Kenya; stateProvince: Nyeri county; locality: Mt. Kenya, Northern Naro Moru, Base camp at Naro Moru River Lodge; verbatimElevation: 3050 m; decimalLatitude: -0.17028; decimalLongitude: 37.215; **Event:** samplingProtocol: sweep net; eventDate: 2008-08-19; habitat: bamboo forest; **Record Level:** institutionCode: TMU

#### Description

Male: Wing length 3.2 mm. **Colouration** (Dry specimen). Head dark brown; face and clypeus brown; labellum and palpus yellow. Antenna with scape and pedicel yellow; flagellum brown, first flagellomere with yellow base. Thorax with scutum brown, lateral margin broadly yellow; lateral sclerites pale brown; propleura yellow; halteres whitish-yellow. Legs whitish-yellow. Abdomen dark brown, tergites II-III with lateral yellow area. Terminalia. **Head**. Frons and vertex covered with pale setae. Clypeus covered with pale brown setae. Antenna long, 2.3 times as long as length from vertex to ventral margin of clypeus; flagellomeres longer than broad, with sixth flagellomere 1.4 times as long as wide. **Thorax**. Scutum covered with pale setae. **Legs**. Fore leg with tibia 0.93 times as long as first tarsomere. Mid-tibia with 20 anterior, 4 posterodorsal, 8 posterior and 4 posteroventral bristles. **Wings**. Vein r-m 3 times longer than stem of M-fork. **Abdomen.** Tergites covered with pale brown setae. **Terminalia** (Fig. 7). Each part of divided tergite IX apically with about 7 setae, apical seta stout. Gonocoxites evenly covered with setae on apicoventral half (Fig. 7a, b). Gonocoxal apicoventral margin, between GL and hypandrium, forming distinct protrusion with 2-3 elongate setae reaching far beyond GL apex (Fig. 7a, b). Each GL small and distinctly curved interiorly, apex with row of 4 short setae somewhat extended down exterior margin (Fig. 7a, b). Aedaegal guides indistinct or reduced. Hypandrium covered with about 6 setae, apical pair elongate, reaching far beyond GL apex (Fig. 7a, b). Hypandrial lobe broad, apically tapering towards rounded apex (Fig. 7a). Gonostylus (Fig. 7c) with DB elongate, about 4 times longer than broad, apex distinctly spathulate; evenly covered with setae on dorsal side, except on apical fourth. VB ovate, apically acute, with 2 setae, apical seta on apex. IB with apex membranous rounded, apical part with row of 3 setae, apical seta close to apex. MB forming small apicointernal extension bearing 2 small setae, 1 basal seta located about the middle.

Female: Unknown.

#### Diagnosis

Distinguished from all species in the *E.parva* group in having the gonocoxal lobes distinctly curved interiorly (Fig. 7a, b).

#### Etymology

From Latin *arcus*, bow, relating to the shape of the gonocoxal lobe.

#### Distribution

Afrotropical, Kenya (Fig. 4).

#### Biology

Adult collected in bamboo forest (3050 m a.s.l.).

### Exechia ashleyi

Lindemann sp. n.

890CB2DD-53CA-5568-8867-F7F09942A52B

urn:lsid:zoobank.org:act:1db2fea8-351d-4fc7-b241-bbbb043edf47

#### Materials

- **Type status:**Holotype. **Occurrence:** catalogNumber: TSZD-JKJ-107227; occurrenceRemarks: BMSA(D): 26355.; recordedBy: A.H. Kirk-Spriggs; individualCount: 1; sex: male; lifeStage: adult; preparations: Pinned, with genitalia in glycerine in separate microvial; **Location:** country: Burundi; stateProvince: Kayanza prov.; municipality: Rwegura; locality: Parc National de la Kibira; verbatimElevation: 2237 m; decimalLatitude: -2.92194; decimalLongitude: 29.5011; **Event:** samplingProtocol: Malaise trap; eventDate: 2010-11-24; habitat: Indigenous Afromontane forest; **Record Level:** institutionCode: BMSA

#### Description

Male: Body length 3.2 mm. Wing length 2.5 mm. **Colouration** (Dry specimen). Head face and clypeus dark brown, almost black; labellum brown; palpus yellow. Antenna with scape and pedicel yellow; flagellum brown, basal half of first flagellomere yellow. Thorax with scutum dark brown, lateral margin pale brown; lateral sclerites and propleura brown; halteres whitish-yellow, apically slightly darker. Legs whitish-yellow. Abdomen dark brown, tergites II-III with slightly paler lateral area. Terminalia yellow. **Head**. Frons and vertex covered with long pale brown setae. Clypeus covered with pale setae, evenly distributed; flagellomeres quadrate, with sixth flagellomere as long as broad. **Thorax**. Scutum covered with long, pale brown setae. **Legs**. Fore leg with tibia 0.76 times as long as first tarsomere. Mid-tibia with 17 anterior, 4 posterodorsal, 9 posterior and no posteroventral bristles. Hind tibia with 7-8 anterodorsal, 5 posterodorsal and 5 posterior bristles. **Abdomen.** Tergites covered with long, dark brown setae. **Terminalia** (Fig. 8). Each part of divided tergite IX apically with about 9 setae, apical stout. Gonocoxites (Fig. 8a, b) evenly covered with setae, except on basoventral third; setae on apicoventral margin reaching as far as GL apex. Each GL apex with 3 setae (Fig. 8a, b). Aedaegal guides short, evenly tapered towards acute apex (Fig. 8a). Hypandrium covered with about 12 setae, apical pair elongate, reaching beyond the GL apex (Fig. 8a, b). Hypandrial lobe with each branch abruptly curved interiorly, apically narrow, apex rounded (Fig. 8a). Gonostylus (Fig. 8c) with DB short, squared, about 2 times longer than broad, apically truncate with apical corners virtually right-angled, apical margin emarginate, dorsal side evenly covered with setae. VB lanceolate, apex with 2 setae, apical seta on apex. IB with apex membranous, acute, apical part with row of 3 setae close to apex. MB short, slender, apex rounded, apically with row of 3 small setae.

Female: Unknown.

#### Diagnosis

Distinguished from *E.penicillata* and *E.sambai* in having the dorsal gonostylus branch short and squared with apico-internal corner right-angled (Fig. 8c), in combination with setae on apicoventral margin of gonocoxites not reaching beyond the gonocoxal lobe apex (Fig. 8a) and by the shape of the hypandrial lobe (Fig. 8a); from *E.burundiensis* in having the dorsal gonostylus branch apically emarginate (Fig. 8c) and the gonocoxal lobe with apical setae parallel, not splaying (Fig. 8a); from *E.afrorepanda* in having the gonocoxal lobe mostly bare, with parallel ventral margins (Fig. 8a); from other species in the *E.parva* group in having the internal gonostylus branch with 3 setae close to the apex (Fig. 8c).

#### Etymology

Named in honour of Dr. Ashley Kirk-Spriggs, the collector of the holotype, who also collected other invaluable material of several species described in this revision.

#### Distribution

Afrotropical, Burundi (Fig. 4).

#### Biology

Adult collected in afromontane forest (2237 m a.s.l.).

### Exechia bifasciata

Lindemann sp. n.

3C953E28-3E88-5C67-9017-A26230E283A4

urn:lsid:zoobank.org:act:2ae21a90-ec81-4425-9af5-00b1bdad220a

#### Materials

- **Type status:**Holotype. **Occurrence:** catalogNumber: TSZD-JKJ-111548; recordedBy: T. Saigusa; individualCount: 1; sex: male; lifeStage: adult; preparations: Pinned, with genitalia in glycerine in separate microvial; **Location:** island: Kyushu; country: Japan; stateProvince: Miyazaki prefecture; locality: Kobayashi-shi, Inokodanibashi 2-5; verbatimElevation: 365-450 m; **Event:** eventDate: 2004-04-17; **Record Level:** collectionCode: KUEC

#### Description

Male: Body length 3.7 mm. Wing length 3.2 mm. **Colouration** (Dry specimen). Head, face and clypeus dark brown; labellum brown; palpus dark brown. Antenna with scape and pedicel yellow; flagellum dark brown, basal half of first flagellomere pale. Scutum and lateral sclerites dark brown; propleura brown; halteres whitish-yellow. Wings hyaline with two weak dark marks, one reaching from apical part of costal cell to middle of cell r4+5 and the other covering area posterior to cubital fork (Fig. 9e). Legs yellow. Abdomen dark brown. Terminalia pale brown. **Head**. Frons and vertex covered with pale setae. Clypeus densely covered with pale setae; flagellomeres quadrate, with sixth flagellomere as long as broad. **Thorax**. Scutum covered with short pale setae. **Legs**. Fore leg with tibia length 0.93 times length of first tarsomere. Mid-tibia with 21 anterior, 5 posterodorsal, 8 posterior and 6 posteroventral bristles. Hind tibia with 11 anterodorsal, 4 posterodorsal and 5 posterior bristles. **Wings** (Fig. 9e). Vein r-m 2 times longer than stem of M-fork. **Abdomen.** Tergites covered with short, pale setae. **Terminalia** (Fig. 9). Each part of divided tergite IX apically with about 20 setae, most apical setae stout. Each cercus triangular with apex truncate and base acute (Fig. 9d). Gonocoxites evenly covered with setae on apicoventral half (Fig. 9a, b). GL very short, length about 0.17 of gonocoxite width, entirely covered with setae, apex with 3 short setae (Fig. 9a, b). Aedaegal guides elongate, curved interiorly, basal part wide, abruptly tapered, apical two-thirds slender, apex rounded (Fig. 9a, b). Hypandrium covered with about 13 setae with apical pair reaching slightly beyond level of GL apex (Fig. 9a, b). Hypandrial lobe with each branch lanceolate, apically rounded (Fig. 9a). Gonostylus (Fig. 9c) with DB large, wide, spathulate, apical half almost as broad as basal half, dorsal side with baso-internal part densly covered with setae, baso-external margin with three small setae. VB ovate, apex rounded, 2 setae close to apex. IB with apex expanded into round membranous area, apically with 1 seta located slightly below apex, pair of setae located one-third from apex. MB large, wide, almost as long as DB, geniculate, apically with 2 very small setae close to apex, medio-internally with 1 seta.

Female: Unknown.

#### Diagnosis

Distinguished from all species in the *E.parva* group in having the wings with dark marks (Fig. 9e), the gonocoxal lobes very short with length only about 0.17 of gonocoxite width (Fig. 9a), each cercus triangular and apically truncate (Fig. 9d), the aedaegal guides very distinctive (Fig. 9a, b) and by the shape of the dorsal and medial branch of the gonostylus (Fig. 9c).

#### Etymology

From Latin *fascia*, band, with the Latin prefix *bi*-, two, relating to the specific wing pattern, forming two dark bands.

#### Distribution

**Figure 10.**
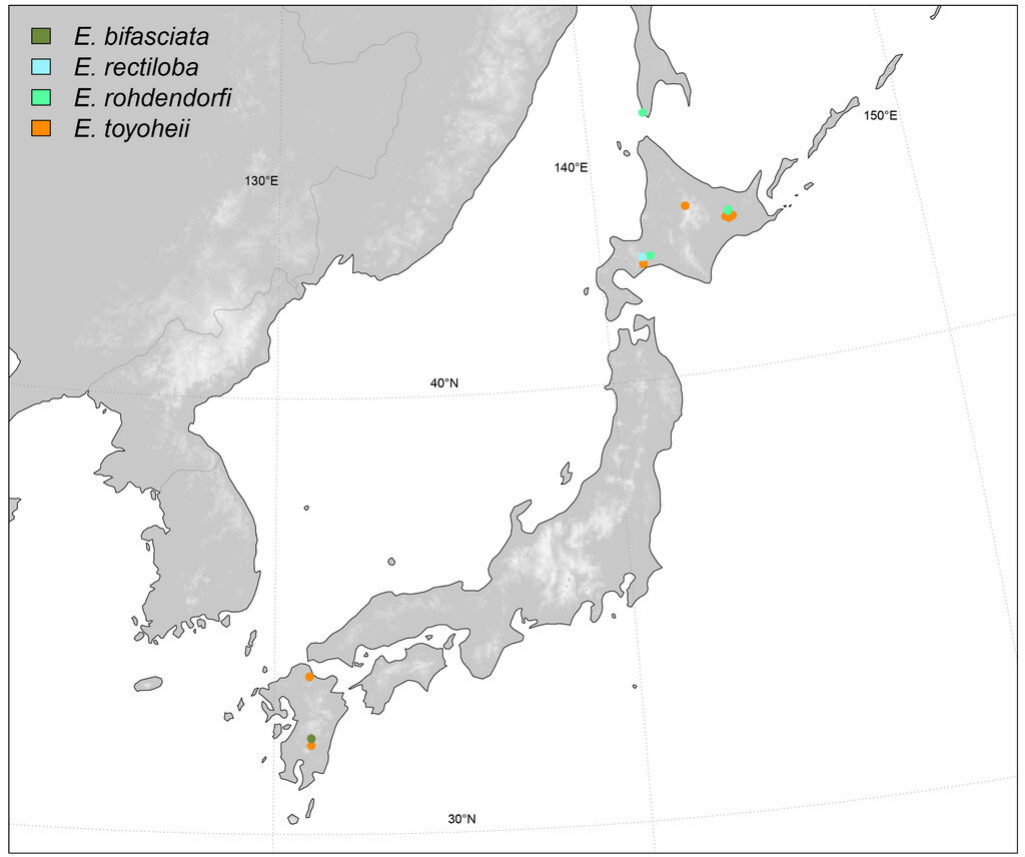
Distribution map of species in the *E.parva* group occurring in the East Palaearctic Region, based on localities from studied material, type material and DNA-barcoded material. Different species are represented by individual colours. Altitudes are indicated in shades from grey (lowland) to white (mountains).

East Palaearctic, Japan (Fig. 10).

#### Biology

Unknown.

### Exechia brachiata

Lindemann sp. n.

CD9A9320-0991-51EF-A4BC-02928D2E96AF

urn:lsid:zoobank.org:act:7181740d-9624-4cff-bf74-8374358fe1c3

#### Materials

- **Type status:**Holotype. **Occurrence:** catalogNumber: TSZD-JKJ-107226; recordedBy: A.H. Kirk-Spriggs; individualCount: 1; sex: male; lifeStage: adult; preparations: Pinned, with genitalia in glycerine in separate microvial; **Location:** country: Madagascar; stateProvince: Fianarantsoa prov.; locality: Ranomafana NP; decimalLatitude: -21.2603; decimalLongitude: 47.4186; **Event:** eventDate: 2007-01-16; **Record Level:** institutionCode: BMSA

#### Description

Male: Body length 3.2 mm. Wing length 2.5 mm. **Colouration** (Dry specimen). Head and face dark brown; clypeus brown; labellum and palpus yellow. Antenna with scape and pedicel yellow; flagellum brown with first flagellomere pale brown. Thorax with scutum and lateral sclerites brown; propleura pale brown; halteres whitish-yellow, apically slightly darker. Legs whitish-yellow. Abdomen dark brown, tergites II-III with lateral yellow area. Terminalia pale brown. **Head**. Frons and vertex covered with long dark brown setae. Clypeus covered with brown setae, more dense towards ventral side; flagellomeres quadrate, with sixth flagellomere as long as broad. **Thorax**. Scutum covered with long dark brown setae. **Legs**. Fore leg with tibia 0.87 times as long as first tarsomere. Mid-tibia with 20 anterior, 5 posterodorsal, 8 posterior and no posteroventral bristles. Hind tibia with 7-8 anterodorsal, 8-9 posterodorsal and 4 posterior bristles. **Abdomen.** Tergites covered with long dark brown to black setae. **Terminalia** (Fig. 11). Each part of divided tergite IX apically with about 6 setae, most apical seta stout. Gonocoxites evenly covered with setae, except on basoventral three-fourths (Fig. 11a, b). Each GL entirely covered with setae, apically with 2 short stout setae (Fig. 11a, b). Aedaegal guides elongate, basal part wide, evenly tapered, apex acute (Fig. 11a, b). Hypandrium with row of 4 large setae reaching about half of GL, otherwise bare (Fig. 11a, b). Hypandrial lobe with basal part large, medial area hyaline or hollow, each branch relatively short, apex rounded (Fig. 11a, b). Gonostylus (Fig. 11c) with DB medially broad, tapered towards narrow apical part, apex rounded, base acuminate, baso-internally forming elongated narrow branch about as long as half DB length, extending in almost straight angle and curving distally; apex with three short stout setae, dorsal side evenly covered with setae, except on apical fourth, on baso-internal branch and on baso-external corner. VB ovate, apically as broad as basally, apex rounded, with 2 setae on apical half. IB short; apex trifurcated with middle branch broad and truncated and lateral branches short and acute; apical part with 2 small setae, one seta located on apical side of each furcation and with 1 seta located further down. MB large, curved interiorly, at about half length bifurcated with each branch apically acute; the internal branch straight and about as long as stem, with two small setae located adjacent to apex; external branch shorter, bent interiorly at a right angle.

Female: Unknown.

#### Diagnosis

Distinguished from all species in the *E.parva* group in having the dorsal branch of the gonostylus with an elongated and narrow baso-internal lobe extending in an almost straight angle and curving distally (Fig. 11c), the internal branch of the gonostylus apically trilobed (Fig. 11c), the medial branches of the gonostylus bilobed (Fig. 11c) and the hypandrium with a row of 4 large setae (Fig. 11a, b).

#### Etymology

From Latin, *brachiatus*, branched, relating to the shape of the gonostylus with bilobed dorsal and medial branches and trilobed internal branch.

#### Distribution

Afrotropical, Madagascar (Fig. 4)

#### Biology

Unknown.

### Exechia breviflagellata

Lindemann sp. n.

392331B5-C101-5BB1-B99E-C0EFE79A4E3C

urn:lsid:zoobank.org:act:952b7008-5f0f-4182-bd34-b96083ea4ace

http://dx.doi.org/10.5883/BOLD:ACI6985

#### Materials

- **Type status:**Holotype. **Occurrence:** catalogNumber: BIOUG27487-H10; recordedBy: BIObus 2014; individualCount: 1; sex: male; lifeStage: adult; preparations: Pinned, with genitalia in glycerine in separate microvial; **Location:** country: Canada; stateProvince: Yukon Territory; locality: Kluane National Park and Reserve, Dezadeash River Trail; verbatimElevation: 582 m; decimalLatitude: 60.748; decimalLongitude: -137.513; **Event:** samplingProtocol: Intercept trap; eventDate: 2014-07-24; habitat: Wetland; fieldNotes: 1 Intercept Trap|cold and overcast on day of collection|Wetland with grasses and shrubs; **Record Level:** institutionCode: CBG
- **Type status:**Paratype. **Occurrence:** catalogNumber: BIOUG09240-B08; recordedBy: Chris Johnstone; individualCount: 1; sex: Female; lifeStage: adult; preparations: Pinned, with genitalia in glycerine in separate microvial; **Location:** country: Canada; stateProvince: Ontario; locality: Georgian Bay Islands National Park, Administration Office, 901 Wye Valley Rd.; verbatimElevation: 190 m; decimalLatitude: 44.7418; decimalLongitude: -79.8501; **Event:** samplingProtocol: Malaise Trap; eventDate: 2013-04-28; habitat: Wetland; fieldNotes: Marsh; **Record Level:** institutionCode: CBG
- **Type status:**Paratype. **Occurrence:** catalogNumber: BIOUG10611-B11; recordedBy: Chris Johnstone; individualCount: 1; sex: male; lifeStage: adult; preparations: Pinned, with genitalia in glycerine in separate microvial; **Location:** country: Canada; stateProvince: Ontario; locality: Georgian Bay Islands National Park, Administration Office, 901 Wye Valley Rd.; verbatimElevation: 190 m; decimalLatitude: 44.7418; decimalLongitude: -79.8501; **Event:** samplingProtocol: Malaise Trap; eventDate: 2013-05-23; habitat: Wetland; fieldNotes: 2 Malaise traps|Marsh; **Record Level:** institutionCode: CBG

#### Description

Male (n = 2): Body length 2.9-3.2 mm. Wing length 2.3-2.5 mm. **Colouration** (Dry specimen). Head dark brown; face and clypeus brown; labellum pale brown; palpus whitish-yellow. Antenna with scape and pedicel brown; flagellum brown. Scutum, lateral sclerites and propleura brown; halteres whitish-yellow. Legs whitish-yellow. Abdomen brown. Terminalia yellow. **Head**. Frons and vertex covered with pale setae. Clypeus covered with few (15-17) pale setae, evenly distributed. Antenna short, 1.45-1.5 times as long as length from vertex to ventral margin of clypeus; flagellomeres quadrate, with sixth flagellomere 0.8-0.9 times as long as wide. **Thorax**. Scutum covered with short pale brown setae. **Legs**. Fore leg with tibia 0.96-1.0 times as long as first tarsomere. Mid-tibia with 20-21 anterior, 3-4 posterodorsal, 7-9 posterior and (n = 1) 2 posteroventral bristles. Hind tibia with 5 anterodorsal, 4-5 posterodorsal and 4-5 posterior bristles. **Wings**. Vein r-m 2.46-2.6 times longer than stem of M-fork. **Abdomen.** Tergites covered with pale brown setae. **Terminalia** (Fig. 12a, b, c). Each part of divided tergite IX with about 4-5 setae, apical seta stout. Gonocoxites evenly covered with setae, except on basoventral third (Fig. 12a, b). GL with length 0.55-0.61 of gonocoxite width, apico-internal margin slightly angled exteriorly, basal third or fourth covered with setae, apex with 3-4 setae (Fig. 12a, b). Aedaegal guides short with acute apex (Fig. 12a, b). Hypandrium covered with 16-19 setae, apical pair reaching about half of the GL (Fig. 12a, b). Hypandrial lobe with each branch slender, evenly tapering. Gonostylus (Fig. 12c) with DB 1.37-1.42 times longer than broad, short and round; apical lobe well defined, short and broad, 0.18-0.2 times as long as the total DB length, apex rounded; evenly covered with setae on dorsal side, except on most apical part; external margin evenly rounded, with row of 4-5 elongate setae. VB round, with 2 small setae. IB apically with 1 seta close to apex and pair of setae one-third from apex. MB with apex acute, 1 seta close to base.

Female (n = 1): Body length 3.1 mm. Wing length 2.7 mm. **Colouration** (Dry specimen). Head, face and clypeus dark brown; labellum pale brown; palpus yellow. Antenna with scape and pedicel yellow; flagellum pale brown. Scutum, lateral sclerites and propleura brown; halteres whitish-yellow. Legs whitish-yellow. Abdomen brown, tergites II-VI with paler lateral areas, not extending notably dorsally. Terminalia yellow. **Head**. Frons and vertex and clypeus covered with pale setae. Antenna short, 1.4 times as long as length from vertex to ventral margin of clypeus; flagellomeres broader than long, with sixth flagellomere 0.7 as long as wide. **Thorax**. Scutum covered with pale brown setae. **Legs**. Fore leg with tibia as long as first tarsomere. Hind tibia with 6 anterodorsal and 6 posterodorsal bristles. **Wings**. Vein r-m 2.8 times longer than stem of M-fork. **Abdomen.** Tergites covered with pale brown setae. **Terminalia** (Fig. 12d, e). Cerci with apical segment 0.7 as long as basal segment. Tergite VIII with apicolateral margin virtually straight (Fig. 12d). Sternite VII with apicoventral margin acuminate. Sternite VIII (Fig. 12e) with hypogynal valves separated by wide v-shaped cleft with depth about one-sixth of sternite VIII and hypogynium length; apical seta about 0.54 times as long as sternite VIII length. Gonapophysis IX with basolateral part expanding in relatively obtuse angle; spermathecal eminence in ventral view appears cross-shaped, but with lateral branches slightly curved distally; gonapophysis IX apically with about 4 small setae.

#### Diagnosis

Distinguished from *E.brevilobata* in having the gonostylus with the dorsal branch only 1.37-1.42 times longer than broad, with its apical lobe broader and shorter, 0.18-0.2 of the total dorsal branch length (Fig. 12c); from *E.sphaerata* and *E.repandoides* in having shorter antennae, only 1.4-1.5 times as long as length from vertex to ventral margin of clypeus, in combination with the gonostylus with the apical lobe of the dorsal branch well defined (Fig. 12c); from other species in the *E.parva* group in having shorter antennae, in combination with the apico-internal margin of the gonocoxal lobe slightly angled exteriorly (Fig. 12a, b).

#### Etymology

From Latin *brevis*, short and *flagellum*, whip, relating to the short antennae of the species.

#### Distribution

**Figure 13.**
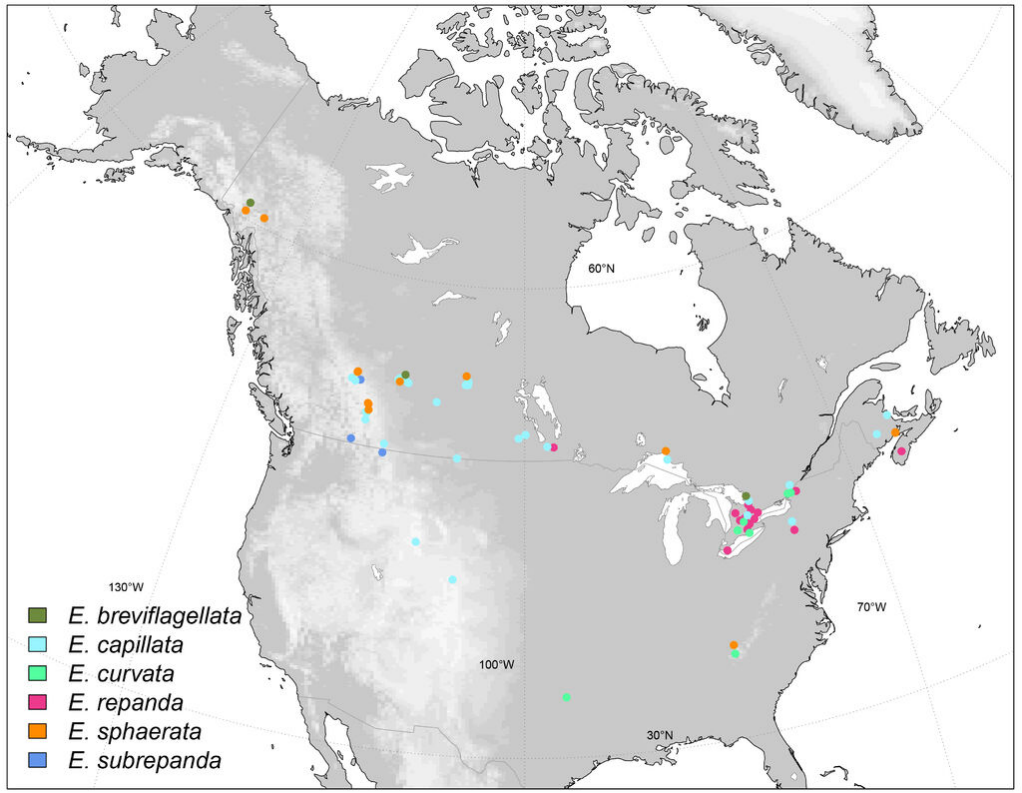
Distribution map of species in the *E.parva* group occurring in the Nearctic Region, based on localities from studied material, type material and DNA-barcoded material. Different species are represented by individual colours. Altitudes are indicated in shades from grey (lowland) to white (mountains).

Nearctic, Canada (Fig. 13).

#### Biology

Adults collected in wetland habitats.

### Exechia brevilobata

Lindemann sp. n.

FD1C5196-A34B-5402-8EB0-B78FB3573517

urn:lsid:zoobank.org:act:7250235c-0edb-4ee2-b56c-1ebec3d5fb66

http://dx.doi.org/10.5883/BOLD:ACI6985

#### Materials

- **Type status:**Holotype. **Occurrence:** catalogNumber: TSZD-JKJ-102846; recordedBy: Rikmyrsprosjektet; individualCount: 1; sex: male; lifeStage: adult; preparations: Pinned (HMDS-dried from ethanol); **Location:** country: Norway; county: HEN; municipality: Engerdal; locality: Åsen; decimalLatitude: 61.88586; decimalLongitude: 11.78283; **Event:** samplingProtocol: window trap; eventDate: 2016-08-18/2016-10-27; **Record Level:** institutionCode: TMU
- **Type status:**Paratype. **Occurrence:** catalogNumber: TSZD-JKJ-215077; recordedBy: K. Müller; individualCount: 1; sex: male; lifeStage: adult; preparations: Pinned (HMDS-dried from ethanol); **Location:** country: Sweden; county: LU; municipality: Jokkmokk; locality: Messaure; verbatimElevation: 175; decimalLatitude: 66.68262; decimalLongitude: 20.36322; **Event:** samplingProtocol: pitfall trap; eventDate: 1972-10-04/1973-05-10; **Record Level:** institutionCode: TMU
- **Type status:**Paratype. **Occurrence:** catalogNumber: TSZD-JKJ-209230; recordedBy: M. Karström; individualCount: 1; sex: male; lifeStage: adult; preparations: 80% alc.; **Location:** country: Sweden; county: LU; municipality: Jokkmokk; locality: Porsitjärn/Porsi VVO, 1.5 km SE Vuollerim; verbatimElevation: 60; decimalLatitude: 66.42444; decimalLongitude: 20.67139; **Event:** samplingProtocol: Window trap; eventDate: 2004-05-06/2004-08-13; **Record Level:** institutionCode: TMU
- **Type status:**Paratype. **Occurrence:** catalogNumber: TSZD-JKJ-209181; recordedBy: M. Karström; individualCount: 1; sex: male; lifeStage: adult; preparations: Pinned (HMDS-dried from ethanol); **Location:** country: Sweden; county: LU; municipality: Jokkmokk; locality: Porsitjärn/Porsi VVO, 1.5 km SE Vuollerim; verbatimElevation: 60; decimalLatitude: 66.42444; decimalLongitude: 20.67139; **Event:** samplingProtocol: Window trap; eventDate: 2004-05-06/2004-08-13; **Record Level:** institutionCode: TMU
- **Type status:**Paratype. **Occurrence:** catalogNumber: TSZD-JKJ-205876; recordedBy: M. Karström; individualCount: 1; sex: male; lifeStage: adult; preparations: Slide mounted in Canada Balsam; **Location:** country: Sweden; county: LU; municipality: Jokkmokk; locality: Porsitjärn/Porsi VVO, 1.5 km SE Vuollerim (Tussilago-lunden); verbatimElevation: 60; decimalLatitude: 66.42444; decimalLongitude: 20.67139; **Event:** samplingProtocol: window trap; eventDate: 2003-04-14/2003-10-18; **Record Level:** institutionCode: TMU
- **Type status:**Paratype. **Occurrence:** catalogNumber: TSZD-JKJ-112073; recordedBy: M. Karström; individualCount: 1; sex: male; lifeStage: adult; preparations: Pinned (HMDS-dried from ethanol); **Location:** country: Sweden; county: LU; municipality: Jokkmokk; locality: Porsitjärn/Porsi VVO, 1.5 km SE Vuollerim; verbatimElevation: 60; decimalLatitude: 66.42444; decimalLongitude: 20.67139; **Event:** samplingProtocol: Window trap; eventDate: 2004-08-13/2004-10-13; **Record Level:** institutionCode: TMU
- **Type status:**Paratype. **Occurrence:** catalogNumber: TSZD-JKJ-112074; recordedBy: M. Karström; individualCount: 1; sex: male; lifeStage: adult; preparations: Pinned (HMDS-dried from ethanol); **Location:** country: Sweden; county: LU; municipality: Jokkmokk; locality: Porsitjärn/Porsi VVO, 1.5 km SE Vuollerim; verbatimElevation: 60; decimalLatitude: 66.42444; decimalLongitude: 20.67139; **Event:** samplingProtocol: Window trap; eventDate: 2004-08-13/2004-10-13; **Record Level:** institutionCode: TMU
- **Type status:**Paratype. **Occurrence:** catalogNumber: TSZD-JKJ-112075; recordedBy: M. Karström; individualCount: 1; sex: male; lifeStage: adult; preparations: Pinned (HMDS-dried from ethanol); **Location:** country: Sweden; county: LU; municipality: Jokkmokk; locality: Porsitjärn/Porsi VVO, 1.5 km SE Vuollerim; verbatimElevation: 60; decimalLatitude: 66.42444; decimalLongitude: 20.67139; **Event:** samplingProtocol: Window trap; eventDate: 2004-08-13/2004-10-13; **Record Level:** institutionCode: TMU
- **Type status:**Paratype. **Occurrence:** catalogNumber: TSZD-JKJ-112076; recordedBy: M. Karström; individualCount: 1; sex: male; lifeStage: adult; preparations: Pinned (HMDS-dried from ethanol); **Location:** country: Sweden; county: LU; municipality: Jokkmokk; locality: Porsitjärn/Porsi VVO, 1.5 km SE Vuollerim; verbatimElevation: 60; decimalLatitude: 66.42444; decimalLongitude: 20.67139; **Event:** samplingProtocol: Window trap; eventDate: 2004-08-13/2004-10-13; **Record Level:** institutionCode: TMU
- **Type status:**Paratype. **Occurrence:** catalogNumber: TSZD-JKJ-112077; recordedBy: M. Karström; individualCount: 1; sex: male; lifeStage: adult; preparations: Pinned (HMDS-dried from ethanol); **Location:** country: Sweden; county: LU; municipality: Jokkmokk; locality: Porsitjärn/Porsi VVO, 1.5 km SE Vuollerim; verbatimElevation: 60; decimalLatitude: 66.42444; decimalLongitude: 20.67139; **Event:** samplingProtocol: Window trap; eventDate: 2004-08-13/2004-10-13; **Record Level:** institutionCode: TMU
- **Type status:**Paratype. **Occurrence:** catalogNumber: TSZD-JKJ-112078; recordedBy: M. Karström; individualCount: 1; sex: male; lifeStage: adult; preparations: Pinned (HMDS-dried from ethanol); **Location:** country: Sweden; county: LU; municipality: Jokkmokk; locality: Porsitjärn/Porsi VVO, 1.5 km SE Vuollerim; verbatimElevation: 60; decimalLatitude: 66.42444; decimalLongitude: 20.67139; **Event:** samplingProtocol: Window trap; eventDate: 2004-08-13/2004-10-13; **Record Level:** institutionCode: TMU
- **Type status:**Paratype. **Occurrence:** catalogNumber: TSZD-JKJ-112079; recordedBy: M. Karström; individualCount: 1; sex: male; lifeStage: adult; preparations: Pinned (HMDS-dried from ethanol); **Location:** country: Sweden; county: LU; municipality: Jokkmokk; locality: Porsitjärn/Porsi VVO, 1.5 km SE Vuollerim; verbatimElevation: 60; decimalLatitude: 66.42444; decimalLongitude: 20.67139; **Event:** samplingProtocol: Window trap; eventDate: 2004-08-13/2004-10-13; **Record Level:** institutionCode: TMU
- **Type status:**Paratype. **Occurrence:** catalogNumber: TSZD-JKJ-112080; recordedBy: M. Karström; individualCount: 1; sex: male; lifeStage: adult; preparations: Pinned (HMDS-dried from ethanol); **Location:** country: Sweden; county: LU; municipality: Jokkmokk; locality: Porsitjärn/Porsi VVO, 1.5 km SE Vuollerim; verbatimElevation: 60; decimalLatitude: 66.42444; decimalLongitude: 20.67139; **Event:** samplingProtocol: Window trap; eventDate: 2004-08-13/2004-10-13; **Record Level:** institutionCode: TMU
- **Type status:**Paratype. **Occurrence:** catalogNumber: TSZD-JKJ-112081; recordedBy: M. Karström; individualCount: 1; sex: male; lifeStage: adult; preparations: Pinned (HMDS-dried from ethanol); **Location:** country: Sweden; county: LU; municipality: Jokkmokk; locality: Porsitjärn/Porsi VVO, 1.5 km SE Vuollerim; verbatimElevation: 60; decimalLatitude: 66.42444; decimalLongitude: 20.67139; **Event:** samplingProtocol: Window trap; eventDate: 2004-08-13/2004-10-13; **Record Level:** institutionCode: MZLU
- **Type status:**Paratype. **Occurrence:** catalogNumber: TSZD-JKJ-112082; recordedBy: M. Karström; individualCount: 1; sex: male; lifeStage: adult; preparations: Pinned (HMDS-dried from ethanol); **Location:** country: Sweden; county: LU; municipality: Jokkmokk; locality: Porsitjärn/Porsi VVO, 1.5 km SE Vuollerim; verbatimElevation: 60; decimalLatitude: 66.42444; decimalLongitude: 20.67139; **Event:** samplingProtocol: Window trap; eventDate: 2004-05-06/2004-08-13; **Record Level:** institutionCode: TMU
- **Type status:**Paratype. **Occurrence:** catalogNumber: TSZD-JKJ-112083; recordedBy: M. Karström; individualCount: 1; sex: male; lifeStage: adult; preparations: Pinned (HMDS-dried from ethanol); **Location:** country: Sweden; county: LU; municipality: Jokkmokk; locality: Porsitjärn/Porsi VVO, 1.5 km SE Vuollerim; verbatimElevation: 60; decimalLatitude: 66.42444; decimalLongitude: 20.67139; **Event:** samplingProtocol: Window trap; eventDate: 2004-05-06/2004-08-13; **Record Level:** institutionCode: MZLU

#### Description

Male (n = 9): Body length (n = 8) 3.5-3.8 mm. Wing length 2.7-3.2 mm. **Colouration** (Dry specimen; n = 2). Head brown to dark brown, face and clypeus pale brown to dark brown; labellum yellow to brown; palpus yellow. Antenna with scape and pedicel yellow; flagellum yellow to brown with basal part of first segment pale. Scutum brown to dark brown, anterolateral areas distinctly paler; lateral sclerites pale brown to dark brown, propleura yellow to dark brown; halteres whitish-yellow. Legs yellow. Abdomen brown to dark brown. Terminalia yellow. **Head**. Frons and vertex covered with whitish to pale brown setae. Clypeus covered with thin pale setae, evenly distributed. Antenna short, 1.5-1.65 times as long as length from vertex to ventral margin of clypeus; flagellomeres quadrate, with sixth flagellomere as long as broad. **Thorax**. Scutum covered with pale brown setae. **Legs**. Fore leg with tibia 1.0 times as long as first tarsomere. Mid-tibia with 20-22 anterior, (n = 3) 4 posterodorsal, 8 posterior and 1-2 posteroventral bristles. Hind tibia (n = 3) with 7-8 anterodorsal, 4 posterodorsal and 4-5 posterior bristles. **Wings**. Vein r-m 2.3-2.5 times longer than stem of M-fork. **Abdomen.** Tergites covered with pale brown setae. **Terminalia** (Fig. 14). Each part of divided tergite IX with 5-6 setae, 1-2 most apical stout. Gonocoxites with GL length 0.58-0.63 of gonocoxite width, apico-internal margin slightly angled exteriorly, basal fifth covered with setae, apex with 3-4 setae (Fig. 14a, b). Aedaegal guides short with acute apex. Hypandrium covered with 10-13 setae, apical pair reaching about half of GL (Fig. 14a). Hypandrial lobe with each branch slender, evenly tapering. Gonostylus (Fig. 14c) with DB short, round, 1.8-2.0 times longer than broad, slightly curved interiorly; apical lobe well defined, short and narrow, 0.21-0.25 times as long as the total DB length, apex rounded; evenly covered with setae on dorsal side, except on most apical part; external margin evenly rounded, with row of 4-5 elongate setae. VB round, with 2 small setae. IB apically with 1 seta close to apex and pair of setae one-third from apex. MB apex acute, with 1 seta close to the base.

Female: Unknown.

#### Diagnosis

Distinguished from *E.breviflagellata* in having the gonostylus with the dorsal branch 1.8-2 times longer than broad, with its apical lobe narrower and longer, 0.21-0.25 of the total dorsal branch length (Fig. 14c); from *E.sphaerata* and *E.repandoides* in having the antennae only 1.5-1.65 times as long as length from vertex to ventral margin of clypeus, in combination with the apical lobe of the dorsal gonostylus branch well defined (Fig. 14c); from other species in the *E.parva* group in having shorter antennae, in combination with apico-internal margin of gonocoxal lobe slightly angled exteriorly.

#### Etymology

From Latin *brevis*, short and *lobatus*, with lobes, relating to the short apical lobe of the dorsal branch of the gonostylus.

#### Distribution

**Figure 15.**
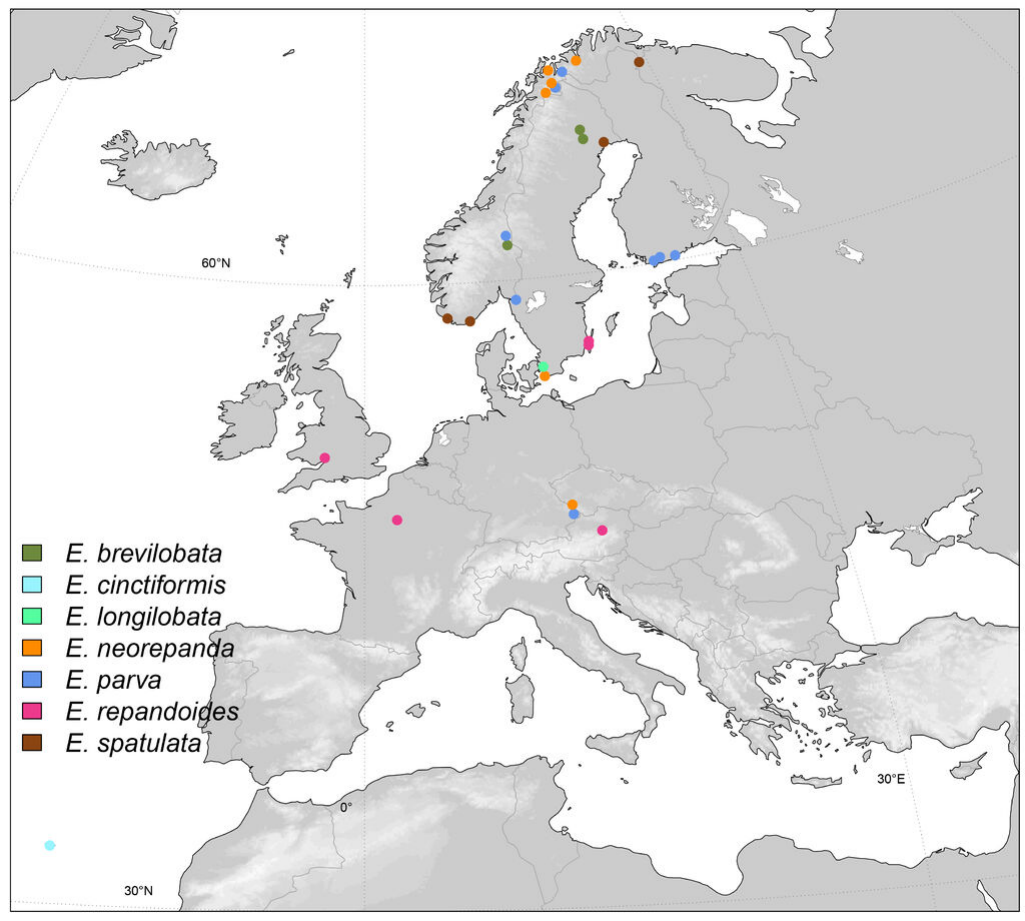
Distribution map of species in the *E.parva* group occurring in the West Palaearctic Region, based on localities from studied material, type material and DNA-barcoded material. Different species are represented by individual colours. Altitudes are indicated in shades from grey (lowland) to white (mountains).

East Palaearctic, Norway, Sweden (Fig. 15).

#### Biology

Unknown.

#### Taxon discussion

Material of this species from Jokkmokk, Sweden, have earlier been identified as *E.repandoides* (Kjærandsen et al. 2007). The two species may have non-overlapping distributions, with *E.brevilobata* only recorded from boreal areas and *E repandoides* only recorded from nemoral areas.

### Exechia burundiensis

Lindemann sp. n.

F9FAA639-7946-5D93-A960-547F0FE34A07

urn:lsid:zoobank.org:act:30637427-b5a9-4104-a6cd-a25cd52aca25

#### Materials

- **Type status:**Holotype. **Occurrence:** catalogNumber: TSZD-JKJ-107224; occurrenceRemarks: BMSA(D): 24977; recordedBy: A.H. Kirk-Spriggs; individualCount: 1; sex: male; lifeStage: adult; preparations: Pinned, with genitalia in glycerine in separate microvial; **Location:** country: Burundi; stateProvince: Kayanza prov.; municipality: Rwegura; locality: Parc National de la Kibira; verbatimElevation: 2237 m; decimalLatitude: -2.92194; decimalLongitude: 29.5011; **Event:** samplingProtocol: Malaise trap; eventDate: 2010-11-24; habitat: Indigenous Afromontane forest; **Record Level:** institutionCode: BMSA

#### Description

Adult male: Body length 2.6 mm. Wing length 2.1 mm. **Colouration** (Dry specimen). Head dark brown; face and clypeus yellow; labellum yellow; palpus yellow, with segments 4-5 yellow to brown. Antenna with scape and pedicel yellow; flagellum brown. Thorax with scutum brown; lateral sclerites pale brown; propleura yellow; halteres whitish-yellow. Legs whitish-yellow. Abdomen dark brown, tergites I-III with slightly paler lateroventral area. Terminalia pale brown. **Head**. Frons and vertex covered with short pale brown setae. Clypeus covered with only a few (about 11) black setae, mostly on ventral side; flagellomeres quadrate, with sixth flagellomere as long as broad. **Thorax**. Scutum covered with short pale brown setae. **Legs**. Fore leg with tibia 0.95 times as long as first tarsomere. Mid-tibia with 16 anterior, 4 posterodorsal, 7 posterior and 2 posteroventral bristles. Hind tibia with 11 anterodorsal, 4 posterodorsal and 3 posterior bristles. **Abdomen.** Tergites covered with long pale brown to yellow setae. **Terminalia** (Fig. 16). Each part of divided tergite IX apically with about 6 setae, most apical seta elongate. Gonocoxites with apicoventral margin with 2 setae reaching beyond GL apex (Fig. 16a, b). GL apex with 3 setae distinctly splaying (Fig. 16a, b). Aedaegal guides short and acuminate (Fig. 16a). Hypandrium covered with about 10 setae, apical pair elongate, reaching beyond GL apex (Fig. 16a, b). Hypandrial lobe with each branch relatively wide, widening somewhat towards the middle, apex narrow, acute (Fig. 16a, b). Gonostylus with DB (Fig. 16c) elongate, spathulate, apico-external corner virtually right-angled, apex rounded, forming acute angle pointing apico-interiorly; dorsal side evenly covered with setae, except on apical fourth. VB ovate, apex acute, with 2 setae, most apical seta on apex. IB apically rounded; distal part with 4 apical setae, otherwise bare. MB short, slightly curved interiorly towards rounded apex, with 3 setae, 2 most apical setae very small, close to apex, basal seta one third from apex.

Female: Unknown.

#### Diagnosis

Distinguished from all species in the *E.parva* group in having the gonocoxal lobe with apical setae distinctly splaying (Fig. 16a, b), the internal gonostylus branch with 4 setae close to the apex (Fig. 16c), by the shape of the hypandrial lobe (Fig. 16a) and the dorsal gonostylus branch (Fig. 16c).

#### Etymology

From *Burundi*, the country where the holotype was collected, with Latin ending -*ensis*, belonging to.

#### Distribution

Afrotropical, Burundi (Fig. 4).

#### Biology

Adult collected in afromontane forest (2237 m a.s.l.).

### Exechia capillata

Johannsen, 1912

AC115234-7745-5E2F-B574-4F3556682C3F

http://dx.doi.org/10.5883/BOLD:AAP2525

Exechia capillata Johannsen, 1912:73 ♂♀ (Johannsen 1912)

#### Materials

- **Type status:**Holotype. **Occurrence:** catalogNumber: TTG-TBB-2046; recordedBy: O. A. Johannsen; individualCount: 1; sex: male; lifeStage: adult; preparations: Slide mounted in canada balsam, terminalia in glycerine in micro-vial; **Record Level:** institutionCode: CUIC
- **Type status:**Other material. **Occurrence:** catalogNumber: BIOUG04555-G02; recordedBy: Brett Sarchuk; individualCount: 1; sex: female; lifeStage: adult; preparations: Pinned, with genitalia in glycerine in separate microvial; **Location:** country: Canada; stateProvince: Alberta; locality: Elk Island National Park, Astotin Lake, The Point, near administration/warden office; verbatimElevation: 719 m; decimalLatitude: 53.685; decimalLongitude: -112.86; **Event:** samplingProtocol: Malaise Trap; eventDate: 2012-09-14; habitat: Forest; fieldNotes: peninsula, emergent white birch/willow/trembling aspen; **Record Level:** institutionCode: CBG
- **Type status:**Other material. **Occurrence:** catalogNumber: BIOUG11733-G07; recordedBy: Bernard Martin; individualCount: 1; sex: female; lifeStage: adult; preparations: Pinned, with genitalia in glycerine in separate microvial; **Location:** country: Canada; stateProvince: New Brunswick; locality: Kouchibouguac National Park, Near Park Compound, behind Research House; verbatimElevation: 61 m; decimalLatitude: 46.7707; decimalLongitude: -65.0064; **Event:** samplingProtocol: Malaise Trap; eventDate: 2013-09-27; habitat: Forest; fieldNotes: 2 Malaise Traps|mixed forest; **Record Level:** institutionCode: CBG
- **Type status:**Other material. **Occurrence:** catalogNumber: 10BBCDIP-0557; recordedBy: BIObus 2010; individualCount: 1; sex: male; lifeStage: adult; preparations: Pinned, with genitalia in glycerine in separate microvial; **Location:** country: Canada; stateProvince: British Columbia; locality: Kootenay NP, Redstreak Campground; verbatimElevation: 1018 m; decimalLatitude: 50.627; decimalLongitude: -116.055; **Event:** samplingProtocol: Pan Trap; eventDate: 2010-07-30; habitat: Mixed Habitat; fieldNotes: 10 Yellow Pans||Mixed coniferous and grasses; **Record Level:** institutionCode: CBG
- **Type status:**Other material. **Occurrence:** catalogNumber: BIOUG21428-G08; recordedBy: M.Otway; individualCount: 1; sex: male; lifeStage: adult; preparations: Pinned, with genitalia in glycerine in separate microvial; **Location:** country: Canada; stateProvince: Saskatchewan; locality: Grasslands National Park, East Block; verbatimElevation: 889 m; decimalLatitude: 49.001; decimalLongitude: -106.557; **Event:** samplingProtocol: Malaise Trap; eventDate: 2014-10-01; habitat: Grassland; fieldNotes: 2 Malaise traps|In shrubland habitat, along a corridor of shrubs in a coulee; **Record Level:** institutionCode: CBG
- **Type status:**Other material. **Occurrence:** catalogNumber: BIOUG31068-C05; recordedBy: Amy Audoux; individualCount: 1; sex: male; lifeStage: adult; preparations: Pinned, with genitalia in glycerine in separate microvial; **Location:** country: Canada; stateProvince: New Brunswick; municipality: Fredericton; locality: Devon Middle School; verbatimElevation: 13 m; locationRemarks: EQP-CLL-911; decimalLatitude: 45.9686; decimalLongitude: -66.624; **Event:** samplingProtocol: Malaise Trap; eventDate: 2016-09-30; **Record Level:** institutionCode: CBG
- **Type status:**Other material. **Occurrence:** catalogNumber: BIOUG31108-D02; recordedBy: Derek Lindskoog; individualCount: 1; sex: male; lifeStage: adult; preparations: Pinned, with genitalia in glycerine in separate microvial; **Location:** country: Canada; stateProvince: Alberta; municipality: Edmonton; locality: Highlands School; verbatimElevation: 658 m; locationRemarks: EQP-CLL-905; decimalLatitude: 53.5707; decimalLongitude: -113.433; **Event:** samplingProtocol: Malaise Trap; eventDate: 2016-09-30; **Record Level:** institutionCode: CBG
- **Type status:**Other material. **Occurrence:** catalogNumber: BIOUG05792-H02; recordedBy: BIOBus 2012; individualCount: 1; sex: female; lifeStage: adult; preparations: Pinned, with genitalia in glycerine in separate microvial; **Location:** country: Canada; stateProvince: Alberta; locality: Jasper National Park, Pocahontas Campground; verbatimElevation: 1131 m; locationRemarks: Site C21; decimalLatitude: 53.195; decimalLongitude: -117.914; **Event:** samplingProtocol: Pitfall Trap; eventDate: 06/14/2012; habitat: Forest; fieldNotes: 10 pitfall traps|birch and spruce forest on a slope, lots of fallen logs; **Record Level:** institutionCode: CBG
- **Type status:**Other material. **Occurrence:** catalogNumber: BIOUG06581-D12; recordedBy: BIOBus 2012; individualCount: 1; sex: female; lifeStage: adult; preparations: Pinned, with genitalia in glycerine in separate microvial; **Location:** country: Canada; stateProvince: Saskatchewan; locality: Prince Albert National Park, Narrows Peninsula Trail; verbatimElevation: 530 m; decimalLatitude: 53.9872; decimalLongitude: -106.282; **Event:** samplingProtocol: Intercept Trap; eventDate: 06/14/2012; habitat: Forest; fieldNotes: 1 intercept trap|partly cloudy|24C|white spruce and poplar forest

#### Description

Male (n = 4): Body length 2.7-3.5 mm. Wing length 2.4-3.0 mm. **Colouration** (Dry specimen). Head dark brown; face and clypeus brown to dark brown; labellum brown; palpus yellow. Antenna with scape and pedicel yellow; flagellum brown to dark brown, basal part of first segment pale. Scutum and lateral sclerites brown to dark brown; propleura yellow to dark brown; halteres whitish-yellow; wings hyaline tinged brown. Legs whitish-yellow. Abdomen dark brown, tergites I-III slightly paler, sometimes tergite II with distinct pale lateral area covering ventral half. Terminalia yellow. **Head**. Frons and vertex covered with pale short setae. Clypeus covered with pale setae, evenly distributed. Antenna (n = 2) 2.0-2.1 times as long as length from vertex to ventral margin of clypeus; flagellomeres quadrate or slightly longer than broad, sixth flagellomere 1.0-1.15 times as long as wide. **Thorax**. Scutum covered with pale setae. **Legs**. Fore leg with tibia (n = 2) 0.9-0.94 times as long as first tarsomere. Mid-tibia with 15-19 anterior, 4-5 posterodorsal, 5-7 posterior and 2-4 posteroventral bristles. Hind tibia with (n = 3) 5-7 anterodorsal, (n = 3) 3-5 posterodorsal and (n = 3) 4 posterior bristles. **Wings**. Vein r-m 2.5-2.9 times longer than stem of M-fork. **Abdomen.** Tergites covered with pale setae. **Terminalia** (n =2, Fig. 17a, b, c). Each part of divided tergite IX with about 3-4 setae, most apical seta stout. Gonocoxites with GL length 0.46-0.48 of gonocoxite width, apico-internal margin slightly angled exteriorly, basal half or basal two thirds covered with setae, apex with 3-4 setae (Fig. 17a, b). Aedaegal guides short and round (Fig. 17a, b). Hypandrium covered with 12-13 setae, apical pair reaching about half of GL (Fig. 17a, b). Hypandrial lobe with each branch slender, evenly tapering. Gonostylus (Fig. 17c) with DB 2.3-2.45 times longer than broad, basal part square-shaped; apical lobe well defined, elongate, curving ventrally, apex rounded; evenly covered with setae on dorsal side, except on apical lobe; medio-external margin expanded distally, forming short process with row of 4-5 setae. VB with 2 small setae. IB apically with 1 seta close to apex and row of 2-3 setae one-third from apex. MB apex acute, with 1 seta close to base.

Female (n = 4): Body length 2.6-2.7 mm. Wing length 2.2-2.3 mm. **Colouration** (Dry specimen). Head dark brown; face and clypeus brown to dark brown; labellum brown; palpus yellow. Antenna with scape and pedicel yellow; flagellum brown, basal part of first flagellomere pale. Thorax with scutum brown to dark brown; lateral sclerites brown; propleura pale brown; halteres whitish-yellow; wings hyaline tinged with brown. Legs whitish-yellow. Abdomen pale brown to dark brown, tergites II-VI with lateral pale areas extending somewhat dorsally along anterior margin of tergites III and VI, but not forming any complete band. Terminalia pale brown. **Head**. Vertex, frons and clypeus covered with pale setae. Antenna (n = 3) 1.8-1.9 times as long as length from vertex to ventral margin of clypeus; flagellomeres quadrate, with sixth flagellomere as long as broad. **Thorax**. Scutum covered with pale setae. **Legs**. Fore leg with tibia (n = 3) 0.95-1.0 times as long as first tarsomere. Mid-tibia with (n = 3) 17-19 anterior, 3-4 posterodorsal, (n = 3) 5-6 posterior and (n = 3) 2-3 posteroventral bristles. Hind tibia with 4-5 anterodorsal, 5-6 posterodorsal and 4 posterior bristles. **Wings**. Vein r-m (n = 1) 2.4-2.6 times longer than stem of M-fork. **Abdomen.** Tergites covered with brown setae. **Terminalia** (Fig. 17d, e, f). Cerci with apical segment half as long as basal segment. Tergite VIII with apicolateral margin slightly angular or virtually straight (Fig. 17d). Sternite VII apicoventral margin evenly rounded. Sternite VIII (Fig. 17e) with hypogynal valves separated by narrow cleft with depth about one-fourth of sternite VIII length; apical seta length 0.6-0.65 of sternite VIII medial length. Gonapophysis IX (Fig. 17f) with basolateral part expanding in relatively obtuse angle; spermathecal eminence in ventral view appearing simple, unbranched; apically with about 8 small setae.

#### Diagnosis

Distinguished from all species in the *E.parva* group in having the dorsal gonostylus branch with the medio-external margin forming a short distally projected process (Fig. 17c), in combination with the gonocoxal lobe with apico-internal margin slightly angled exteriorly (Fig. 17a).

#### Distribution

Nearctic, Canada, USA (Fig. 13).

#### Biology

Reared from fruitbody of *Collybiadryophila* (Bull. : Fr.) P. Kumm. = *Gymnopusdryophilus* (Bull. : Fr.) Murrill (Johannsen 1912). Adults collected in different types of forest, grassland and wetland habitats.

#### Notes

Holotype with whole specimen, except terminalia mounted on slide in Canada balsam with one wing detached. Terminalia in alcohol, transferred to glycerine and associated with empty pin with labels in type collection. A second tube with terminalia of E.cf.dorsalis misplaced in same vial - this was also transferred to glycerine in a microvial and pinned separately.

### Exechia chirotheca

Lindemann sp. n.

A1BB2947-FC76-5A03-BEA7-75730CD425A2

urn:lsid:zoobank.org:act:b9d1788f-c210-48b8-92b3-2c691e25a2f2

#### Materials

- **Type status:**Holotype. **Occurrence:** catalogNumber: TSZD-JKJ-111552; recordedBy: J. Emoto; individualCount: 1; sex: male; lifeStage: adult; preparations: Pinned, with genitalia in glycerine in separate microvial; **Location:** country: Nepal; stateProvince: Province No. 1; county: Terhathum District; locality: Basantapur; verbatimElevation: 2300 m; decimalLatitude: 27.1333; decimalLongitude: 87.4333; **Event:** eventDate: 1972-05-06; **Record Level:** institutionCode: KUEC

#### Description

Male: Body length 3.3 mm. Wing length 2.8 mm. **Colouration** (Dry specimen). Head, face dark and clypeus dark brown; labellum pale brown; palpus yellow. Antenna with scape and pedicel yellow; flagellum brown, basal half of first flagellomere pale. Scutum, lateral sclerites and propleura brown; halteres yellow. Legs yellow. Abdomen dark brown. Terminalia yellow. **Head**. Frons and vertex covered with pale setae. Clypeus covered with pale setae, evenly distributed. Antenna long, 2.2 times as long as length from vertex to ventral margin of clypeus; flagellomeres longer than broad, with sixth flagellomere 1.5 as long as wide. **Thorax**. Scutum covered with pale setae. **Legs**. Fore leg with tibia 0.83 times as long as first tarsomere. Mid-tibia with 21 anterior, 3 posterodorsal, 10 posterior and 3 posteroventral bristles. Hind tibia with 9 anterodorsal, 4 posterodorsal and 6 posterior bristles. **Wings**. Vein r-m 2.9 times longer than stem of M-fork. **Abdomen.** Tergites covered with short, pale setae. **Terminalia** (Fig. 18). Each part of divided tergite IX apically with about 6 setae, the 2-3 most apical setae stout. Gonocoxites evenly covered with setae, except on basoventral half (Fig. 18a, b); GL apex with 3 stout short setae (Fig. 18a, b). Aedaegal guides elongate with parallel margins, apex acute (Fig. 18a, b). Hypandrium with about 6 setae, apical pair relatively short, reaching about the GL basal fourth (Fig. 18a, b). Hypandrial lobe with each branch basally wide, apically narrow, evenly tapered (Fig. 18a). Gonostylus (Fig. 18c) with DB large, elongate, apex rounded, apicoexternal margin forming a short rounded lobe extending distally; dorsal side evenly covered with setae. VB apically acuminate, apex acute, with 2 setae, most apical seta on apex. IB apically with 1 seta on apex and pair of setae on elevated area one-third from apex. MB short, slightly curved interiorly towards acute apex; apically forming a small finger-like process, with 2 setae on the apex.

#### Diagnosis

Distinguished from all species in the *E.parva* group in having the medial gonostylus branch with an apical finger-like process with 2 setae at the apex (Fig. 18c), the gonocoxal lobe apex with relatively short and stout setae (Fig. 18a, b) and by the shape of the dorsal gonostylus branch (Fig. 18c).

#### Etymology

From Latin *chirotheca*, mitten, relating to the shape of the dorsal and medial lobes of the gonostylus, resembling mittens.

#### Distribution

**Figure 19.**
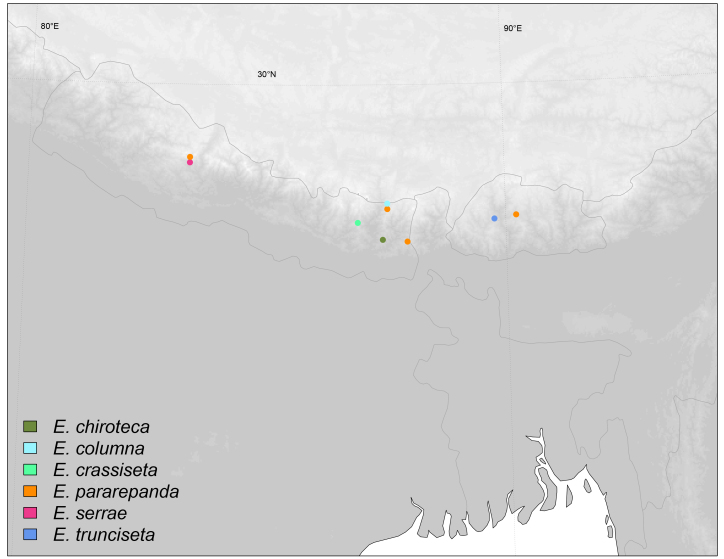
Distribution map of species in the *E.parva* group occurring in the eastern Himalayas (Oriental Region), based on localities from studied material, type material and DNA-barcoded material. Different species are represented by individual colours. Altitudes are indicated in shades from grey (lowland) to white (mountains).

Oriental, Nepal (Fig. 19).

#### Biology

Adult collected in the eastern Himalayas (2300 m a.s.l.).

### Exechia cinctiformis

Storå, 1941

DE409AAE-F291-5610-8FF3-830388D73DD4

Exechia cinctiformis Storå, 1941: 2 ♂♀ (Storå 1941)
Exechia sp. Storå, 1941:3 ♀ (Storå 1941)
Exechia dahli Nielsen, 1966: 8 ♂♀ (Nielsen 1966)

#### Distribution

West Palaearctic, Madeira (Fig. 15)

#### Notes

We did not have the opportunity to re-describe this species, but the male terminalia are well illustrated in Chandler and Ribeiro (1995) who also noted that the male terminalia resemble *E.parva* on the apicolateral margin and *E.repanda* on the gonostylus.

#### Figures

Male: Fig. 20. Map: Fig. 15.

### Exechia columna

Lindemann sp. n.

B1B0383C-A9D0-5C64-B455-ED53BCC0EAAC

urn:lsid:zoobank.org:act:01744a34-c29f-41c8-8bdc-f629b4600422

#### Materials

- **Type status:**Holotype. **Occurrence:** catalogNumber: TSZD-JKJ-111553; recordedBy: H. Shima; individualCount: 1; sex: male; lifeStage: adult; preparations: Pinned, with genitalia in glycerine in separate microvial; **Location:** country: Nepal; stateProvince: Province no. 1 (Kosi Zone); county: Sankhuwasabha District; locality: Thudam; verbatimElevation: 3500 m; decimalLatitude: 27.7500; decimalLongitude: 87.5333; **Event:** samplingProtocol: Malaise Trap; eventDate: 1972-07-02; **Record Level:** institutionCode: KUEC

#### Description

Male: Body length 3.9 mm. Wing length 3.2 mm. **Colouration** (Dry specimen). Head, face and clypeus dark brown; labellum dark brown; palpus whitish-yellow. Antenna with scape and pedicel yellow; flagellum pale brown, basal half of first flagellomere pale. Thorax with scutum pale brown, lateral margin paler; lateral sclerites brown; propleura brown; halteres whitish-yellow. Legs whitish-yellow. Abdomen dark brown, tergites II-III with a lateral pale area. Terminalia brown. **Head**. Frons and vertex covered with pale setae. Clypeus covered with only few (about 17) pale setae. Antenna long, 2.1 times as long as length from vertex to ventral margin of clypeus; flagellomeres longer than broad, sixth flagellomere 1.5 times as long as wide. **Thorax**. Scutum covered with pale setae. **Legs**. Fore leg with tibia 0.91 times as long as first tarsomere. Mid-tibia with 19 anterior, 4 posterodorsal, 7 posterior and 3 posteroventral bristles. **Wings**. Vein r-m 2.14 times longer than stem of M-fork. **Abdomen.** Tergites covered with pale brown setae. **Terminalia** (Fig. 21). Each part of divided tergite IX apically with about 4 setae, most apical seta stout. Gonocoxites with GL elongate, entirely covered with setae, apex with 2 short setae (Fig. 21a, b). Aedaegal guides elongate and spathulate (Fig. 21a). Hypandrium strongly extended distally, forming a large lobe that reaches far beyond apicoventral gonocoxal margin, covered with about 10 setae, with apical pair stout, reaching almost level of GL apex (Fig. 21a, b). Hypandrial lobe with each branch elongate, narrow, apically rounded (Fig. 21a). Gonostylus (Fig. 21c) with DB forming a large distally projecting baso-external lobe; basal part rounded, abruptly tapered, apical part slender elongate, apex rounded; basodorsal part evenly covered with setae; baso-external lobe with 2 short stout setae on apex. VB small and apically rounded, with 2 setae, most apical seta on apex. IB with apical part slender and elongate; 1 seta on apex and row of 4 setae on elevated area close to middle. MB elongate; internal margin smooth; apically somewhat hollowed with small seta within hollow area close to apex.

Female: Unknown.

#### Diagnosis

Distinguished from *E.serrae* in having the gonocoxal lobe extended beyond the apex of the apical hypandrial setae (Fig. 21a, b) and the medial gonostylus branch with internal margin smooth (Fig. 21c); from other species in the *E.parva* group in having the hypandrium forming a distally-extended lobe reaching far beyond the apicoventral gonocoxal margin (Fig. 21a, b) and the dorsal gonostylus branch with a large baso-external lobe with two apical setae (Fig. 21c).

#### Etymology

From Latin *columna*, column, relating to the shape of the hypandrium forming a large distally-extended lobe.

#### Distribution

Oriental, Nepal (Fig. 19)

#### Biology

Adult collected in the eastern Himalayas (3500 m a.s.l.).

### Exechia crassiseta

Lindemann sp. n.

37C20DEC-E3FA-5637-BE19-AA911CC23187

urn:lsid:zoobank.org:act:dd60a76e-48a1-4991-8cc6-20c9f368603a

#### Materials

- **Type status:**Holotype. **Occurrence:** catalogNumber: TSZD-JKJ-111554; recordedBy: J. Emoto; individualCount: 1; sex: male; lifeStage: adult; preparations: Pinned, with genitalia in glycerine in separate microvial; **Location:** country: Nepal; stateProvince: Province no. 1 (Kosi Zone); county: Sankhuwasabha District; locality: Salpa La; verbatimElevation: 2900-3000 m; decimalLatitude: 27.450000; decimalLongitude: 86.916667; **Event:** eventDate: 1981-07-29; **Record Level:** institutionCode: KUEC

#### Description

Male: Body length 3.6 mm. Wing length 3.2 mm. **Colouration** (Dry specimen). Head, face and clypeus dark brown; labellum pale brown; palpus yellow to pale brown. Antenna with scape and pedicel yellow; flagellum dark brown, basal half of first flagellomere yellow. Thorax with scutum dark brown, except narrow yellow anterolateral margin; lateral sclerites dark brown; propleura brown; halteres whitish-yellow. Legs yellow. Abdomen dark brown, tergites II-III with yellow laterodorsal area. Terminalia pale brown with MB dark brown. **Head**. Frons and vertex covered with brown setae. Clypeus covered with pale setae, most dense towards ventral side; flagellomeres quadrate, with sixth flagellomere as long as broad. **Thorax**. Scutum covered with brown setae. **Legs**. Fore leg with tibia 0.7 times length of first tarsomere. Mid-tibia with 23 anterior, 5 posterodorsal, 10 posterior and 5 posteroventral bristles. Hind tibia with 10 anterodorsal, 6 posterodorsal and 4 posterior bristles. **Wings**. Vein r-m 3.3 times longer than stem of M-fork. **Abdomen.** Tergites covered with long brown setae. **Terminalia** (Fig. 22). Each part of divided tergite IX apically with about 4 setae, most apical seta stout. Gonocoxites with apicoventral margin between GL and hypandrium forming short protrusion, each with 2 very stout and apically truncated setae (Fig. 22a, b); GL apex with 2-3 relatively short and stout setae (Fig. 22a, b). Aedaegal guides elongate, converging, apically acute (Fig. 22a). Hypandrium with about 8 setae, apical pair not reaching longer than half the GL length (Fig. 22a, b). Hypandrial lobe with each branch narrow, evenly tapering, apex acute. Gonostylus (Fig. 22c) with DB elongate, apex rounded, baso-internally forming small lobe, extending interiorly; dorsal side evenly covered with relatively stout setae, except on most basal part and internal lobe; apically with about 4 very stout and apically truncated setae. VB ovate, apex acute, with 1 elongate seta on apex and 3 smaller setae further down, one distinctly wider than others. Apical part of IB with 1 seta on apex and row of 4 setae on elevation one-fifth from the apex. MB short, apex acute, internal margin forming curved acute process, external margin forming short acute process, apex with row of 4 elongate setae, all longer than MB length.

Female: Unknown.

#### Diagnosis

Distinguished from *E.trunciseta* by the shape of the medial gonostylus branch (Fig. 22c); from other species in the *E.parva* group in having the dorsal gonostylus branch apically with a row of 4 stout truncate setae, baso-internally with a short lobe (Fig. 22c) and in having the medial gonostylus branch darkened, apically with a row of 4 setae, all of which are longer than the medial gonostylus branch length (Fig. 22c).

#### Etymology

From Latin *crassus*, stout and *seta*, bristle, relating to the shape of the seta on apicoventral margin of the gonocoxites and apically on the dorsal branch of the gonostylus.

#### Distribution

Oriental, Nepal (2900-3000 m a.s.l., Fig. 19).

#### Biology

Unknown

### Exechia curvata

Lindemann sp. n.

4C1393AF-9BC7-58C7-B636-E48F7F1D1E14

urn:lsid:zoobank.org:act:f73e7d88-14b3-4e50-8ca2-cdc3cbe12988

http://dx.doi.org/10.5883/BOLD:AAN8586

#### Materials

- **Type status:**Holotype. **Occurrence:** catalogNumber: BIOUG05015-F10; recordedBy: Becky Nichols; individualCount: 1; sex: male; lifeStage: adult; preparations: Pinned (Dried from HMDS); **Location:** country: United States; stateProvince: Tennessee; locality: Great Smoky Mountains National Park, Twin Creeks Science and Education Center; verbatimElevation: 559 m; decimalLatitude: 35.6859; decimalLongitude: -83.4986; **Event:** samplingProtocol: Malaise Trap; eventDate: 2012-10-30; **Record Level:** institutionCode: CBG
- **Type status:**Paratype. **Occurrence:** catalogNumber: BIOUG00965-E09; recordedBy: James Sones; individualCount: 1; sex: female; lifeStage: adult; preparations: Pinned (Dried from HMDS); **Location:** country: Canada; stateProvince: Ontario; locality: Leeds and Grenville, Elizabethtown-Kitley, 4452 Rowsome Rd., Elizabethtown; verbatimElevation: 112 m; locationRemarks: MT-2; decimalLatitude: 44.621; decimalLongitude: -75.773; **Event:** samplingProtocol: Malaise Trap; eventDate: 2010-10-14; habitat: Temperate mixed forest; **Record Level:** institutionCode: CBG
- **Type status:**Paratype. **Occurrence:** catalogNumber: BIOUG05033-B01; recordedBy: Becky Nichols; individualCount: 1; sex: female; lifeStage: adult; preparations: Pinned (Dried from HMDS); **Location:** country: United States; stateProvince: Tennessee; locality: Great Smoky Mountains National Park, Twin Creeks Science and Education Center; verbatimElevation: 559 m; decimalLatitude: 35.6859; decimalLongitude: -83.4986; **Event:** samplingProtocol: Malaise Trap; eventDate: 2012-12-06; **Record Level:** institutionCode: CBG
- **Type status:**Paratype. **Occurrence:** catalogNumber: BIOUG21858-G11; recordedBy: CSC; individualCount: 1; sex: female; lifeStage: adult; preparations: Pinned (Dried from HMDS); **Location:** country: Canada; stateProvince: Ontario; locality: Thousand Islands National Park; verbatimElevation: 91 m; decimalLatitude: 44.453; decimalLongitude: -75.865; **Event:** samplingProtocol: Malaise Trap; eventDate: 29-Sep-2014; habitat: Mixed habitat; fieldNotes: Shoreline transition area (from emergent typha marsh to mixed forest edge); T1; **Record Level:** institutionCode: CBG
- **Type status:**Paratype. **Occurrence:** catalogNumber: BIOUG16127-C09; recordedBy: Claire Gulliver; individualCount: 1; sex: female; lifeStage: adult; preparations: Pinned (Dried from HMDS); **Location:** country: Canada; stateProvince: Ontario; locality: London; verbatimElevation: 259 m; locationRemarks: EQP-CLL-589; decimalLatitude: 43.03; decimalLongitude: -81.271; **Event:** samplingProtocol: Malaise Trap; eventDate: 03-Oct-2014; **Record Level:** institutionCode: CBG

#### Description

Male (n = 1): Body length 3.2 mm. Wing length 2.8 mm. **Colouration** (Dry specimen). Head, face and clypeus dark brown; labellum dark brown; palpus yellow. Antenna with scape and pedicel yellow; flagellum pale brown, basal half of first flagellomere pale. Thorax with scutum dark brown, anterior and lateral margin paler; lateral sclerites and propleura brown; halteres whitish-yellow. Legs whitish-yellow. Abdomen dark brown with pale lateral area confined to ventral margin of tergite II and basolateral part of tergite III. Terminalia yellow. **Head**. Vertex, frons and clypeus covered with pale setae. Antenna 1.9 times as long as length from vertex to ventral margin of clypeus; flagellomeres longer than broad, with sixth flagellomere 1.2 times as long as wide. **Thorax**. Scutum covered with pale setae. **Legs**. Fore leg with tibia 1.05 times as long as first tarsomere. Mid-tibia with 20 anterior, 4 posterodorsal, 10 posterior and 3 posteroventral bristles. Hind tibia with 6 anterodorsal, 5 posterodorsal and 3 posterior bristles. **Wings**. Vein r-m 2.22 times longer than stem of M-fork. **Abdomen.** Tergites covered with pale setae. **Terminalia** (n = 2, Fig. 23a, b, c). Each part of divided tergite IX with about 7-8 setae, most apical seta stout. GL with length 0.42-0.48 gonocoxite width, apico-internal margin slightly angled exteriorly, basal third covered with setae, apex with 3 setae (Fig. 23a, b). Aedaegal guides short with acute apex. Hypandrium covered with about 12 setae, with the apical pair reaching about half of the GL (Fig. 23a, b). Hypandrial lobe with each part apically slightly widened. Gonostylus (Fig. 23c) with DB about 2.4 times longer than broad, distinctly curved interiorly, apical lobe well defined, apex rounded; evenly covered with setae on dorsal side, except on the most apical part; external margin forming a distinct and slightly protruding angle, with row of 4 elongate setae. VB round, with 2 small setae. IB apically with 1 seta close to apex and pair of setae one-third from apex. MB apex acute, with 1 seta close to the base.

Female (n = 4): Body length 2.6-2.8 mm. Wing length 2.4-2.5 mm. **Colouration** (Dry specimen). Head, face and clypeus dark brown; labellum and palpus yellow. Antenna with scape and pedicel yellow; flagellum pale brown, basal half of first flagellomere pale. Thorax with scutum brown to dark brown; lateral sclerites brown; propleura brown; halteres whitish-yellow. Legs whitish-yellow. Abdomen brown to dark brown, tergites II-VI with pale lateral areas extending dorsally at anterior fourth to third of tergite III and anterior third to half of tergite IV, forming two distinct pale bands. Terminalia pale brown. **Head**. Vertex, frons and clypeus covered with pale setae. Antenna 1.75-1.9 times as long as length from vertex to ventral margin of clypeus; flagellomeres slightly longer than broad, with sixth flagellomere (n = 2) 1.0-1.1 times as long as wide. **Thorax**. Scutum covered with pale setae. **Legs**. Fore leg with tibia as long as first tarsomere. Mid-tibia with 20-24 anterior, (n = 3) 3 posterodorsal, (n = 3) 6-8 posterior and 1-3 posteroventral bristles. Hind tibia with (n = 3) 6-7 anterodorsal, 4 posterodorsal and 3-4 posterior bristles. **Wings**. Vein r-m 2.0-2.2 times longer than stem of M-fork. **Abdomen.** Tergites covered with brown setae on dark areas and pale setae on pale areas. **Terminalia** (Fig. 23d, e). Cerci with basal segment 0.6 times as long as basal segment. Tergite VIII with apicolateral margin forming distinctly protruding angle (Fig. 23d). Sternite VIII (Fig. 23e) with hypogynal valves separated by narrow cleft with depth about one-sixth of sternite VIII length; apical seta 0.6-0.67 of sternite VIII length. Gonapophysis IX with basolateral part expanding in relatively obtuse angle; spermathecal eminence trilobed, in ventral view appearing cross-shaped; apically with about 8 small setae.

#### Diagnosis

Distinguished from *E.repanda*, *E.subrepanda* and *E.neorepanda* in having the dorsal gonostylus branch more curved and with external margin forming a distinct protruding angle, with the external row of setae covering only about one sixth of the total dorsal gonostylus branch length (Fig. 23c), in combination with the gonocoxal lobe length only 0.42-0.48 times the gonocoxite width (Fig. 23a, b); from other species in the *E.parva* group in having the dorsal branch about 2.4 times longer than broad (Fig. 23c), in combination with the gonocoxal lobe covered with setae on about the basal third, with the apico-internal margin slightly angled exteriorly (Fig. 23a, b).

#### Etymology

From Latin *curvus*, curved, relating to the shape of the dorsal branch of the gonostylus.

#### Distribution

Nearctic, Canada, USA (Fig. 13).

#### Biology

Adult collected in temperate mixed forest.

### Exechia longichaeta

Wu, Xu & Yu, 2004

9CD41099-2E07-5C9E-97BB-0CA8715A9CBF

Exechia longichaeta Wu, Xu & Yu, 2004: 555 ♂ (Wu et al. 2004)

#### Distribution

Palaearctic, Oriental, China

#### Taxon discussion

Wu et al. (2004) suggested that the species was similar to *E.pollex* Shaw, 1935, which is closely related to *E.spinigera* Winnertz, 1863 in the *E.fusca* group. However, based on the shape of the deeply biforked hypandrial lobe, as well as the elongate GLs with apical setae (Fig. 24a), we consider it more appopriate to place the species within the *E.parva* group. Comparison of illustrations of male terminalia in ventral view indicates that *E.longichaeta* is likely a close ally of, or possibly even a junior synonym of *E.pararepanda.* This is evident when considering that, in both species, the apical part of the hypandrium is not distinctly fused with the gonocoxites, as well as the shape and apical setae of the GLs and the profile of the DB of the gonostylus. It is possible that the two species are sympatric, as *E.longichaeta* is distributed as far south-west as Kunming, Yunnan (Wu et al. 2004).

#### Notes

We did not have the opportunity to re-describe this species; however, the male terminalia are well illustrated in Wu et al. (2004).

### Exechia longilobata

Lindemann sp. n.

38689566-07C8-59A4-BCD1-884912FBF000

urn:lsid:zoobank.org:act:f8d0b8b2-d202-4232-b897-f3bf8d8c42d0

http://dx.doi.org/10.5883/BOLD:ADD3869

#### Materials

- **Type status:**Holotype. **Occurrence:** catalogNumber: TSZD-JKJ-102235; recordedBy: Bo W. Svensson et al.; individualCount: 1; sex: male; lifeStage: adult; preparations: Pinned, with genitalia in glycerine in separate microvial; **Location:** country: Sweden; county: Skåne; municipality: Malmö; locality: Limhamns kalkbrott; verbatimElevation: -40 m; decimalLatitude: 55.5681; decimalLongitude: 12.9241; **Event:** samplingProtocol: Malaise Trap; eventDate: 2011-11-15; habitat: Limestone quarry; **Record Level:** institutionCode: TMU
- **Type status:**Paratype. **Occurrence:** catalogNumber: TSZD-JKJ-259573; recordedBy: B. W. Svensson & Co.; individualCount: 1; sex: male; lifeStage: adult; preparations: Pinned, with genitalia in glycerine in separate microvial; **Location:** country: Sweden; county: Skåne; municipality: Malmö; locality: Limhamns kalkbrott; verbatimElevation: -40 m; locationRemarks: MT 1 - "grafitti"; decimalLatitude: 55.56826; decimalLongitude: 12.92408; **Event:** samplingProtocol: Malaise trap; eventDate: 2011-11-01/2011-11-30; habitat: Limestone quarry; **Record Level:** institutionCode: TMU
- **Type status:**Paratype. **Occurrence:** catalogNumber: SPM-054527; recordedBy: Bo W. Svensson et al.; individualCount: 1; sex: male; lifeStage: adult; preparations: Pinned, with genitalia in glycerine in separate microvial; **Location:** country: Sweden; county: Skåne; municipality: Malmö; locality: Limhamns kalkbrott; verbatimElevation: -40 m; locationRemarks: MT2 - "planen"; decimalLatitude: 55.5681; decimalLongitude: 12.9241; **Event:** samplingProtocol: Malaise Trap; eventDate: 2009-04-23/2009-05-10; habitat: Limestone quarry; **Record Level:** institutionCode: TMU
- **Type status:**Paratype. **Occurrence:** catalogNumber: SPM-058935; recordedBy: Bo W. Svensson et al.; individualCount: 1; sex: male; lifeStage: adult; preparations: Pinned, with genitalia in glycerine in separate microvial; **Location:** country: Sweden; county: Skåne; municipality: Malmö; locality: Limhamns kalkbrott; verbatimElevation: -40 m; decimalLatitude: 55.56826; decimalLongitude: 12.92408; **Event:** samplingProtocol: Malaise trap; eventDate: 2012-02-29/2012-03-30; habitat: Limestone quarry; **Record Level:** institutionCode: TMU

#### Description

Male (n = 4): Body length 3.0-3.1 mm. Wing length 2.5 mm. **Colouration** (Dry specimen). Head, face and clypeus dark brown; labellum yellow; palpus yellow. Antenna with scape and pedicel yellow; flagellum yellow. Thorax with scutum and lateral sclerites brown; propleura pale brown; halteres whitish-yellow, apically slightly darker. Legs yellow. Abdomen brown with tergite II slightly paler or with pale area covering its ventral half. Terminalia yellow. **Head**. Frons, vertex and clypeus covered with pale setae. Antenna short, 1.53-1.64 times as long as length from vertex to ventral margin of clypeus; flagellomeres quadrate, with sixth flagellomere 0.9-0.95 times as long as wide. **Thorax**. Scutum covered with pale setae. **Legs**. Fore leg with tibia 0.96-1.1 times as long as first tarsomere. Mid-tibia (n = 1) with 19 anterior, 4 posterodorsal, 7 posterior and 2 posteroventral bristles. Hind tibia (n = 1) with 7 anterodorsal and 2 posterodorsal bristles. **Wings**. Vein r-m 2.5-3.1 times longer than stem of M-fork. **Abdomen.** Tergites covered with pale setae. **Terminalia** (Fig. 25). Each part of divided tergite IX with about 7-9 setae, most apical seta stout. GL with length 0.4-0.54 of gonocoxite width, apico-internal margin slightly angled exteriorly, basal third covered with setae, apex with 3 setae (Fig. 25a, b). Aedaegal guides somewhat elongated, evenly tapered, acute apex (Fig. 25a, b). Hypandrium covered with 25-31 setae, with the apical pair reaching about two-thirds of GL (Fig. 25a, b). Hypandrial lobe with each branch with apical half distinctly widened with apex somewhat acute (Fig. 25a, b). Gonostylus with DB (Fig. 25c) 3.0-3.2 times longer than broad, slightly curved interiorly; apical lobe well defined, elongated, narrow, apex rounded; evenly covered with setae on dorsal side, except on most apical part; medio-external margin forming distinct angle, with row of 4 elongate setae. VB apically rounded with 2 small setae. IB apically with 1 seta close to apex and pair of setae one-third from apex. MB apex acute with 1 seta close to the base.

Female: Unknown.

#### Diagnosis

Distinguished from *E.spatulata* in having the dorsal gonostylus branch more narrow, not spathulate, only 3-3.2 times longer than broad (Fig. 25c), the hypandrium more setose with 25-31 setae (Fig. 25a), each part of the hypandrial lobe with apical half expanded to a wide disc (Fig. 25a), the aedaegal guides evenly tapered (Fig. 25a) and the gonocoxal lobe length only 0.4-0.52 times the gonocoxite width (Fig. 25a, b); from other species in the *E.parva* group in having the dorsal gonostylus branch more elongate (Fig. 25c), the hypandrial lobe with apical half of each branch widened (Fig. 25a) and the gonocoxal lobes with apico-internal margin slightly angled exteriorly (Fig. 25a).

#### Etymology

From Latin *longus*, long and *lobatus*, with lobes, relating to the long apical part of the dorsal branch of the gonostylus.

#### Distribution

West Palaearctic, Sweden (Fig. 15)

#### Biology

Adults collected in limestone quarry.

### Exechia neorepanda

Lindemann sp. n.

1D7CF3D3-8301-556D-AB50-E1E7356D3BD7

urn:lsid:zoobank.org:act:fb17387f-c74d-45de-94ec-33380499362e

http://dx.doi.org/10.5883/BOLD:ACJ3411

#### Materials

- **Type status:**Holotype. **Occurrence:** catalogNumber: TSZD-JKJ-103405; recordedBy: Jostein Kjærandsen & Martin T. Dahl; individualCount: 1; sex: male; lifeStage: adult; preparations: Pinned, with genitalia in glycerine in separate microvial; **Location:** country: Norway; county: Finnmark; municipality: Alta; locality: Mattisdalen indre V; locationRemarks: WT-6; decimalLatitude: 69.8267; decimalLongitude: 22.8493; **Event:** samplingProtocol: Window trap; eventDate: 2017-07-10; **Record Level:** institutionCode: TMU
- **Type status:**Paratype. **Occurrence:** catalogNumber: TSZD-JKJ-102234; recordedBy: Bo W. Svensson et al.; individualCount: 1; sex: male; lifeStage: adult; preparations: Pinned, with genitalia in glycerine in separate microvial; **Location:** country: Sweden; county: Skåne; municipality: Malmö; locality: Limhamns kalkbrott; verbatimElevation: -40 m; decimalLatitude: 55.5681; decimalLongitude: 12.9241; **Event:** samplingProtocol: Malaise Trap; eventDate: 2011-12-16; habitat: Limestone quarry; **Record Level:** institutionCode: TMU
- **Type status:**Paratype. **Occurrence:** catalogNumber: TSZD-JKJ-101379; recordedBy: J. Kjærandsen & M. Torp; individualCount: 1; sex: male; lifeStage: adult; preparations: Pinned, with genitalia in glycerine in separate microvial; **Location:** country: Norway; county: Troms; municipality: Bardu; locality: Kjeleelvdalen, Rohkunborri NP; locationRemarks: MT-2; decimalLatitude: 68.588; decimalLongitude: 18.584; **Event:** samplingProtocol: Malaise Trap; eventDate: 2015-10-08; **Record Level:** institutionCode: TMU
- **Type status:**Paratype. **Occurrence:** catalogNumber: TSZD-JKJ-101398; recordedBy: J. Kjærandsen & M. Torp; individualCount: 1; sex: male; lifeStage: adult; preparations: Pinned, with genitalia in glycerine in separate microvial; **Location:** country: Norway; county: Troms; municipality: Bardu; locality: Kjeleelvdalen, Rohkunborri NP; locationRemarks: MT-2; decimalLatitude: 68.588; decimalLongitude: 18.584; **Event:** samplingProtocol: Malaise Trap; eventDate: 2015-10-08; **Record Level:** institutionCode: TMU
- **Type status:**Paratype. **Occurrence:** catalogNumber: TSZD-JKJ-101840; recordedBy: Jostein Kjærandsen & Martin T. Dahl; individualCount: 1; sex: male; lifeStage: adult; preparations: Pinned, with genitalia in glycerine in separate microvial; **Location:** country: Norway; county: Troms; municipality: Bardu; locality: Kjeleelvdalen, Rohkunborri NP; locationRemarks: MT-2; decimalLatitude: 68.588; decimalLongitude: 18.584; **Event:** samplingProtocol: Malaise Trap; eventDate: 2015-09-04; **Record Level:** institutionCode: TMU
- **Type status:**Paratype. **Occurrence:** catalogNumber: TSZD-JKJ-100510; recordedBy: J. Kjærandsen; individualCount: 1; sex: male; lifeStage: adult; preparations: Pinned, with genitalia in glycerine in separate microvial; **Location:** country: Norway; county: Troms; municipality: Målselv; locality: Dividalen, Velstjonna SE; decimalLatitude: 68.996; decimalLongitude: 19.489; **Event:** samplingProtocol: Interception trap; eventDate: 2014-11-04; **Record Level:** institutionCode: TMU
- **Type status:**Paratype. **Occurrence:** catalogNumber: TSZD-JKJ-102591; recordedBy: J. Kjærandsen; individualCount: 1; sex: male; lifeStage: adult; preparations: Pinned, with genitalia in glycerine in separate microvial; **Location:** country: Norway; county: Troms; municipality: Tromsø; locality: Nakkemyren at Nakkeelva; verbatimElevation: 154; decimalLatitude: 69.6022; decimalLongitude: 19.5847; **Event:** samplingProtocol: Malaise trap; eventDate: 2016-09-03; **Record Level:** collectionID: TMU-JKJ-COL-000432; institutionCode: TMU
- **Type status:**Paratype. **Occurrence:** catalogNumber: TSZD-JKJ-111221; recordedBy: J. Kjærandsen; individualCount: 1; sex: male; lifeStage: adult; preparations: Pinned, with genitalia in glycerine in separate microvial; **Location:** country: Norway; county: Sør-Trøndelag; municipality: Oppdal; locality: Dovre, Grønbekken; verbatimElevation: 920; decimalLatitude: 62.279; decimalLongitude: 9.598; **Event:** samplingProtocol: Sweep net; eventDate: 2020-07-07; **Record Level:** collectionID: TMU-JKJ-COL-001481; institutionCode: TMU
- **Type status:**Paratype. **Occurrence:** catalogNumber: TSZD-JPL-100101; recordedBy: Jon Peder Lindemann; individualCount: 1; sex: male; lifeStage: adult; preparations: Pinned; **Location:** country: Norway; county: Troms; municipality: Tromsø; locality: Tromsøya, Folkeparken; decimalLatitude: 69.634975; decimalLongitude: 18.903577; **Event:** samplingProtocol: hand picked; eventDate: 2021-04-04; habitat: in stems of *Heracleumpersicum*; **Record Level:** institutionCode: TMU
- **Type status:**Paratype. **Occurrence:** catalogNumber: TSZD-JPL-100103; recordedBy: Jon Peder Lindemann; individualCount: 1; sex: male; lifeStage: adult; preparations: Pinned; **Location:** country: Norway; county: Troms; municipality: Tromsø; locality: Tromsøya, Folkeparken; decimalLatitude: 69.634975; decimalLongitude: 18.903577; **Event:** samplingProtocol: hand picked; eventDate: 2021-04-04; habitat: in stems of *Heracleumpersicum*; **Record Level:** institutionCode: TMU
- **Type status:**Paratype. **Occurrence:** catalogNumber: TSZD-JPL-100105; recordedBy: Jon Peder Lindemann; individualCount: 1; sex: male; lifeStage: adult; preparations: Pinned; **Location:** country: Norway; county: Troms; municipality: Tromsø; locality: Tromsøya, Folkeparken; decimalLatitude: 69.634975; decimalLongitude: 18.903577; **Event:** samplingProtocol: hand picked; eventDate: 2021-04-04; habitat: in stems of *Heracleumpersicum*; **Record Level:** institutionCode: TMU

#### Description

Male (n = 11, Fig. 1): Body length (n = 7) 3.2-3.6 mm. Wing length 2.7-3.0 mm. **Colouration** (Dry specimen). Head, face and clypeus dark brown; labellum yellow to brown; palpus yellow. Antenna with scape and pedicel yellow; flagellum pale brown to dark brown, basal half of first flagellomere pale. Thorax and lateral sclerites dark brown; propleura brown to dark brown; halteres yellow to whitish-yellow. Legs yellow. Abdomen dark brown. Terminalia yellow to brown. **Head**. Vertex, frons and clypeus covered with pale setae. Antenna (n = 6) 1.7-1.95 times as long as length from vertex to ventral margin of clypeus; flagellomeres quadrate or longer than broad, with sixth flagellomere 1.0-1.3 times as long as wide. **Thorax**. Scutum covered with pale setae. **Legs**. Fore leg with tibia (n = 6) 0.9-1.0 times as long as first tarsomere. Mid-tibia with 18-22 anterior, 3-4 posterodorsal, (n = 6) 4-6 posterior and (n = 6) 2-3 posteroventral bristles. Hind tibia with (n = 5) 6-7 anterodorsal, (n = 5) 4-5 posterodorsal and 2-4 posterior bristles. **Wings**. Vein r-m (n = 6) 1.83-2.8 times longer than stem of M-fork. **Abdomen.** Tergites covered with pale setae. **Terminalia** (n = 7, Fig. 26). Each part of divided tergite IX with 3-4 setae, most apical seta stout. GL with length 0.53-0.63 of gonocoxite width, apico-internal margin slightly angled exteriorly, basal third covered with setae, apex with 4-5 setae (Fig. 26a, b). Aedaegal guides short with acute apex (Fig. 26a, b). Hypandrium covered with 11-13 setae, with the apical pair reaching about two-thirds of the GL (Fig. 26a, b). Each part of hypandrial lobe with apical half slightly widened. Gonostylus (Fig. 26c) with DB 2.3-2.5 times longer than broad, slightly curved interiorly, apical lobe well defined, apex rounded; dorsal side evenly covered with setae, except on most apical part; external margin evenly rounded, with row of 4-5 elongate setae. VB apically acute, with 2 small setae. IB apically with 1 seta close to apex and pair of setae one-third from apex. MB apex acute, with 1 seta close to the base.

Female: Unknown.

#### Diagnosis

Distinguished from *E.curvata*, *E.repanda* and *E.subrepanda* in having the dorsal gonostylus branch only slightly curved, the external margin without a distinct angle and with the external row of setae covering one-third to half of the total dorsal gonostylus branch length (Fig. 26c); from other species in the *E.parva* group in having the dorsal branch 2.3-2.5 times longer than broad (Fig. 26c), in combination with the gonocoxal lobe covered with setae on basal half or less (Fig. 26a, b), with the apico-internal margin slightly angled exteriorly (Fig. 26a, b).

#### Etymology

From Greek *neos*, new and *repanda*, relating to the resemblance of the species to *E.repanda*. The name indicates that the species has commonly been identified as *E.repanda*.

#### Distribution

West Palaearctic, Austria, Belgium, Britain, Corsica, Czech Republic, Denmark, Estonia, Finland, France, Germany, Hungary, Italy, Norway, Poland, Russia, Sweden, Switzerland, The Netherlands (Fig. 15, Chandler 2005)

#### Biology

Reared from fruitbody of *Lyophyllumloricatum* (Fr.) Kühner, *Mycenagalericulata* (Scop.) Gray, *Kuehneromycesmutabilis* (Schaeff.) Singer & A.H.Sm., *Gyromitraesculenta* (Pers.) Fr., *Inocybegoodeyi* Gillet, and *Calocybegambosa* (Fr. : Fr.) Donk (Dely-Draskovits 1974, Ševčík 2006, Chandler 2010, Jakovlev 1994).

Adults have been reported hibernating in hollow, usually broken umbelliferous stems (Väisänen 1981, Kurina 1997) and we have also collected adults from broken stems of *Heracleumpersicum* Desf. ex Fisch. in Tromsø, Norway during the winter season (Fig. 1c, d).

#### Taxon discussion

This Palaearctic species is morphologically very close to the two Nearctic species, *E.repanda* and *E.subrepanda* and species determination should be conducted with care or confirmed by their distinctly-separated DNA barcodes and BINs. We assume that previous records of *E.repanda* in the West-Palaearctic Region constitute what we define as *E.neorepanda* sp. n.

### Exechia pararepanda

Kallweit, 1995

BCD2B67C-52D8-507F-8037-D9E5E1D521A0

Exechia pararepanda Kallweit in Kallweit & Martens, 1995: 243 ♂ (Kallweit and Martens 1995)

#### Materials

- **Type status:**Other material. **Occurrence:** catalogNumber: TSZD-JKJ-111555; recordedBy: A. Nakanishi; individualCount: 1; sex: male; lifeStage: adult; preparations: Pinned, with genitalia in glycerine in separate microvial; **Location:** country: Nepal; stateProvince: Gandaki Pradesh; county: Myagdi District; locality: Mudi, Dobang Kharka; verbatimElevation: 2400 m; decimalLatitude: 28.6000; decimalLongitude: 83.4000; **Event:** eventDate: 1971-10-20; **Record Level:** institutionCode: KUEC
- **Type status:**Other material. **Occurrence:** catalogNumber: TSZD-JKJ-111560; recordedBy: A. Nakanishi; individualCount: 1; sex: male; lifeStage: adult; preparations: Pinned, with genitalia in glycerine in separate microvial; **Location:** country: Nepal; stateProvince: Gandaki Pradesh; county: Myagdi District; locality: Mudi, Dobang Kharka; verbatimElevation: 2400 m; decimalLatitude: 28.6000; decimalLongitude: 83.4000; **Event:** eventDate: 1971-10-25/1971-10-26; **Record Level:** institutionCode: KUEC
- **Type status:**Other material. **Occurrence:** catalogNumber: TSZD-JKJ-111558; recordedBy: J. Emoto; individualCount: 1; sex: male; lifeStage: adult; preparations: Pinned, with genitalia in glycerine in separate microvial; **Location:** country: Nepal; stateProvince: Province No. 1; county: Taplejung District; locality: Topke Gola; verbatimElevation: 3700 m; decimalLatitude: 27.6333; decimalLongitude: 87.5833; **Event:** eventDate: 1972-07-08; **Record Level:** institutionCode: KUEC
- **Type status:**Other material. **Occurrence:** catalogNumber: TSZD-JKJ-111559; recordedBy: J. Emoto; individualCount: 1; sex: male; lifeStage: adult; preparations: Pinned, with genitalia in glycerine in separate microvial; **Location:** country: Nepal; stateProvince: Province No. 1; county: Taplejung District; locality: Thurukpa - Topke Gola; verbatimElevation: 2600 m; decimalLatitude: 27.6333; decimalLongitude: 87.5833; **Event:** eventDate: 1972-06-12; **Record Level:** institutionCode: TMU
- **Type status:**Other material. **Occurrence:** catalogNumber: TSZD-JKJ-111557; individualCount: 1; sex: male; lifeStage: adult; preparations: Pinned, with genitalia in glycerine in separate microvial; **Location:** country: Nepal; stateProvince: Province No. 1; county: Sankhuwasabha District; locality: Thudam; verbatimElevation: 3500 m; decimalLatitude: 27.7500; decimalLongitude: 87.5333; **Event:** samplingProtocol: Malaise Trap; eventDate: 1974-07-01; **Record Level:** institutionCode: KUEC
- **Type status:**Other material. **Occurrence:** catalogNumber: TSZD-JKJ-111556; recordedBy: T. Saigusa; individualCount: 1; sex: male; lifeStage: adult; preparations: Pinned, with genitalia in glycerine in separate microvial; **Location:** country: Bhutan; locality: Pele La; verbatimElevation: 3400 m; **Event:** eventDate: 1993-08-06; **Record Level:** institutionCode: KUEC

#### Description

Male (n = 6): Body length (n = 3) 2.5-3.2 mm. Wing length (n = 3) 2.1-2.7 mm. **Colouration** (Dry specimen). Head, face and clypeus dark brown; labellum and palpus yellow. Antenna with scape and pedicel yellow; flagellum brown to dark brown, basal half of first flagellomere yellow. Scutum brown to dark brown, with anterolateral margin yellow; lateral sclerites brown to dark brown; propleura pale brown to brown; halteres whitish-yellow to yellow. Legs whitish-yellow to yellow. Abdomen brown to dark brown. Terminalia yellow to pale brown. **Head**. Frons and vertex covered with short, pale setae. Clypeus covered with few (7-15) thin pale setae, mainly on ventral half. Antenna long, (n = 2) 2.0-2.1 times as long as length from vertex to ventral margin of clypeus ; flagellomeres longer than broad, with sixth flagellomere (n = 5) 1.2-1.4 times as long as wide. **Thorax**. Scutum covered with short pale setae. **Legs**. Fore leg with tibia (n = 3) 0.88-0.91 times as long as first tarsomere. Mid-tibia with (n = 5) 15-19 anterior, (n = 5) 2-4 posterodorsal, (n = 5) 6-8 posterior and (n = 5) 1-4 posteroventral bristles. Hind tibia with 4-8 anterodorsal, 4 posterodorsal and 2-3 posterior bristles. **Wings**. Vein r-m (n = 3) 1.81-2.2 times longer than stem of M-fork. **Abdomen.** Tergites covered with pale brown setae. **Terminalia** (Fig. 27). Each part of divided tergite IX apically with 4-7 setae, most apical seta stout. Gonocoxal apicoventral margin, between hypandrium and GL with 2-3 long and stout setae, usually reaching beyond GL apex (Fig. 27a, b). Each GL apically with row of 5 setae, extending from apex along apical third or fourth of the external margin (Fig. 27a, b). Aedaegal guides short, evenly tapering towards acute apex (Fig. 27a). Hypandrium with apical part not distinctly fused with gonocoxites (Fig. 27b), sometimes slightly protruding, covered with 7-12 setae, with apical pair stout and elongate, reaching beyond GL apex (Fig. 27a, b). Hypandrial lobe with each branch elongated, narrow, evenly tapering. Gonostylus (Fig. 27c) with DB slightly curved interiorly; apical lobe well defined, elongated, apex rounded; baso-internal and basodorsal part covered with several setae; apicodorsally with row of 2-3 elongate setae. VB round, with 2 setae, most apical seta on apex. IB apically rounded, apically with 1 seta on apex and pair of setae one-third from apex. MB short, curved interiorly towards acute, sometimes somewhat bifurcated apex; apically with 2 small setae.

Female: Unknown.

#### Diagnosis

Distinguished from all species in the *E.parva* group in having the gonocoxal lobe apex with a row of 5 setae extending down apico-exterior margin (Fig. 27a, b), the hypandrium with apical part not distinctly fused with gonocoxites (Fig. 27b) and the apicoventral gonocoxal setae and apical hypandrial setae stout reaching beyond apex of the gonocoxal lobes (Fig. 27a, b).

#### Distribution

Oriental, Bhutan, Nepal (Fig. 19)

#### Biology

Adults collected in the eastern Himalayas (2400-3400 m a.s.l.). Kallweit and Martens (1995) collected adults in a forest of *Lithocarpus*, *Quercus*, *Magnolia*, and *Rhododendron* species, trapped in inflorescences of the pitcher plant *Arisaemagriffithii*.

### Exechia parva

Lundström, 1909

7537C99A-75D1-53F6-A887-9B1AA60C3424

http://dx.doi.org/10.5883/BOLD:ACJ4957

Exechia parva Lundström, 1909: 50 ♂ (Lundström 1909)

#### Materials

- **Type status:**Lectotype. **Occurrence:** recordedBy: R. Frey; individualCount: 1; sex: male; lifeStage: adult; preparations: pinned; **Location:** country: Finland; locality: Karislojo; **Record Level:** institutionCode: MZHF
- **Type status:**Other material. **Occurrence:** catalogNumber: TSZD-JKJ-101428; recordedBy: J. Kjærandsen & M. Torp; individualCount: 1; sex: male; lifeStage: adult; preparations: Pinned, with genitalia in glycerine in separate microvial; **Location:** country: Norway; county: Troms; municipality: Målselv; locality: Skaktardalen N, Øvre Dividal; locationRemarks: LVN, WT-2; decimalLatitude: 68.763; decimalLongitude: 19.724; **Event:** samplingProtocol: window trap; eventDate: 2015-10-07; **Record Level:** institutionCode: TMU
- **Type status:**Other material. **Occurrence:** catalogNumber: TSZD-JKJ-101682; recordedBy: J. Kjærandsen & M. Torp; individualCount: 1; sex: male; lifeStage: adult; preparations: Pinned, with genitalia in glycerine in separate microvial; **Location:** country: Norway; county: Troms; municipality: Målselv; locality: Skaktarelvmoen, Øvre Dividal; locationRemarks: LVN, WT-1; decimalLatitude: 68.767; decimalLongitude: 19.708; **Event:** eventDate: 2015-07-27; **Record Level:** institutionCode: TMU
- **Type status:**Other material. **Occurrence:** catalogNumber: TSZD-JKJ-102584; recordedBy: J. Kjærandsen; individualCount: 1; sex: male; lifeStage: adult; preparations: Pinned, with genitalia in glycerine in separate microvial; **Location:** country: Norway; county: Troms; municipality: Kåfjord; locality: Kåfjorddalen; verbatimElevation: 118 m; decimalLatitude: 69.4327; decimalLongitude: 20.9674; **Event:** samplingProtocol: sweep net; eventDate: 2016-09-22; **Record Level:** institutionCode: TMU
- **Type status:**Other material. **Occurrence:** catalogNumber: TSZD-JKJ-102825; recordedBy: Rikmyrsprosjektet; individualCount: 1; sex: male; lifeStage: adult; preparations: Pinned, with genitalia in glycerine in separate microvial; **Location:** country: Norway; county: Hedmark; municipality: Engerdal; locality: Åsen; locationRemarks: Site 6; decimalLatitude: 61.8859; decimalLongitude: 11.7828; **Event:** samplingProtocol: Malaise trap - 6; eventDate: 2016-07-16; **Record Level:** institutionCode: TMU
- **Type status:**Other material. **Occurrence:** catalogNumber: TSZD-JKJ-107532; recordedBy: J. Kjærandsen; individualCount: 1; sex: female; lifeStage: adult; preparations: Pinned, with genitalia in glycerine in separate microvial; **Location:** country: Norway; county: Hordaland; municipality: Bømlo; locality: Vorland, Langevag; decimalLatitude: 59.6074; decimalLongitude: 5.21117; **Event:** eventDate: 2003-01-26; habitat: in umbelliferous stems; **Record Level:** collectionID: COL-008481; institutionCode: TMU
- **Type status:**Other material. **Occurrence:** catalogNumber: TSZD-JKJ-107531; recordedBy: J. Kjærandsen; individualCount: 1; sex: female; lifeStage: adult; preparations: Pinned, with genitalia in glycerine in separate microvial; **Location:** country: Norway; county: Hordaland; municipality: Bømlo; locality: Vorland, Langevag; decimalLatitude: 59.6074; decimalLongitude: 5.21117; **Event:** eventDate: 2003-01-26; habitat: in umbelliferous stems; **Record Level:** collectionID: COL-008481; institutionCode: TMU
- **Type status:**Other material. **Occurrence:** catalogNumber: TSZD-JKJ-107530; recordedBy: J. Kjærandsen; individualCount: 1; sex: female; lifeStage: adult; preparations: Pinned, with genitalia in glycerine in separate microvial; **Location:** country: Norway; county: Hordaland; municipality: Sveio; locality: Førde; decimalLatitude: 59.614; decimalLongitude: 5.47238; **Event:** eventDate: 2003-11-16; habitat: in umbelliferous stems; **Record Level:** collectionID: COL-008478; institutionCode: TMU
- **Type status:**Other material. **Occurrence:** catalogNumber: TSZD-JKJ-107529; recordedBy: J. Kjærandsen; individualCount: 1; sex: female; lifeStage: adult; preparations: Pinned, with genitalia in glycerine in separate microvial; **Location:** country: Norway; county: Hordaland; municipality: Sveio; locality: Førde; decimalLatitude: 59.614; decimalLongitude: 5.47238; **Event:** eventDate: 2003-11-16; habitat: in umbelliferous stems; **Record Level:** collectionID: COL-008478; institutionCode: TMU

#### Description

Male (n = 4): Body length 3.0-3.8 mm. Wing length 2.5-3.2 mm. **Colouration** (Dry specimen). Head, face and clypeus dark brown; labellum and palpus yellow to pale brown. Antenna with scape and pedicel yellow; flagellum dark brown to pale brown, basal half of first flagellomere pale. Scutum dark brown; lateral sclerites and propleura brown to dark brown; halteres whitish-yellow. Legs yellow to whitish-yellow. Abdomen brown to dark brown, tergites I-III slightly paler, sometimes tergite II with pale area confined to a narrow stripe along ventral margin. Terminalia yellow. **Head**. Vertex, frons and clypeus covered with pale setae. Antenna (n = 2) 1.8-2 times as long as length from vertex to ventral margin of clypeus ; flagellomeres longer than broad, with sixth flagellomere 1.1-1.3 times as long as wide. **Thorax**. Scutum covered with pale to pale brown setae. **Legs**. Fore leg with tibia (n = 1) 0.95 times as long as first tarsomere. Mid-tibia with 18-22 anterior, 3-4 posterodorsal, 4-8 posterior and (n = 2) 2-3 posteroventral bristles. Hind tibia with (n = 3) 6-8 anterodorsal, (n = 2) 5 posterodorsal and (n = 2) 4 posterior bristles. **Wings**. Vein r-m (n = 2) 2.45-2.5 times longer than stem of M-fork. **Abdomen.** Tergites covered with pale to pale brown setae. **Terminalia** (Fig. 28a, b, c). Each part of divided tergite IX with 6-8 setae, 2 most apical setae stout. GL with length 0.33-0.4 of gonocoxite width, evenly tapered, apico-internal margin slightly angled exteriorly, entire length covered with setae, apex with 3-4 setae (Fig. 28a, b). Aedaegal guides short and round (Fig. 28a). Hypandrium covered with 10-12 setae, with apical pair reaching about half of the GL (Fig. 28a). Hypandrial lobe with each branch basally wide, apical half narrow, apex rounded. Gonostylus (Fig. 28c) with DB 1.4-1.6 times longer than borad, short and round, slightly curved interiorly; apical lobe well defined, short and narrow, apex rounded; dorsal side evenly covered with setae, except on most apical part; medio-external margin angled, with row of 4-5 short setae. VB with 2 small setae. IB apically with 1 seta close to the apex and pair of setae one-fourth from apex. MB short, curved interiorly, apex acute, with 1 seta close to the base.

Female (n = 4): Body length 3.3-3.9 mm. Wing length 2.9-3.2 mm. **Colouration** (Dry specimen). Head, face and clypeus brown to dark brown; labellum and palpus yellow. Antenna with scape and pedicel yellow; flagellum pale brown to brown, basal part of first flagellomere pale. Thorax with scutum brown to dark brown; lateral sclerites pale brown to brown; halteres whitish-yellow; wings hyaline tinged with brown. Legs whitish-yellow. Abdomen brown to dark brown, tergites III-VI with lateral pale areas, extending from lateral margin to about half tergal height. Terminalia pale brown. **Head**. Vertex, frons and clypeus covered with pale setae. Antenna (n = 2) 1.7-1.8 times as long as length from vertex to ventral margin of clypeus; flagellomeres quadrate, with sixth flagellomere as long as broad. **Thorax**. Scutum covered with pale brown setae. **Legs**. Fore leg with tibia 0.93-1.0 times as long as first tarsomere. Mid-tibia with 23-26 anterior, 3-4 posterodorsal, 6-8 posterior and 2-3 posteroventral bristles. Hind tibia with 5-6 anterodorsal, 5 posterodorsal and 3-5 posterior bristles. **Wings**. Vein r-m (n = 2) 2.7-2.8 times longer than stem of M-fork. **Abdomen.** Tergites covered with pale brown setae. **Terminalia** (n = 3, Fig. 28d, e). Cerci with apical segment 0.57-0.65 times as long as basal segment. Tergite VIII with apicolateral margin slightly angular or virtually straight. Sternite VII with apicoventral margin angular or slightly acuminate, apex truncate. Sternite VIII (Fig. 28d) with hypogynal valves separated by a wide cleft with depth about one-seventh of sternite VIII length; apical lobes almost as long as breadth of hypogynial valve in ventral view; apical seta length 0.67-0.73 of sternite VIII medial length. Gonapophysis IX (Fig. 28e) with basolateral part expanding in a relatively obtuse angle; spermathecal eminence in ventral view trifurcate with middle branch large and round and lateral branches short.

#### Diagnosis

Distinguished from all species in the *E.parva* group in having the gonocoxal lobe evenly tapered and entirely covered with setae (Fig. 28a, b), in combination with the gonocoxal lobes with apico-internal margin slightly angled exteriorly (Fig. 28a, b) and the internal branch of the gonostylus with only one seta close to the apex (Fig. 28c).

#### Distribution

East Palaearctic, West Palaearctic, Austria, Belgium, Britain, Bulgaria, Croatia, Czech Republic, Estonia, Finland, France, Germany, Ireland, Latvia, Norway, Poland, Russia, Slovenia, Sweden, Switzerland, The Netherlands (Polevoi and Barkalov 2017, Chandler 2005, Fig. 15).

#### Biology

Reared from fruitbody of *Nematoloma* sp. [? *udum* (Pers. : Fr.) Karst.] = *Hypholoma* sp. [? *udum* (Pers. : Fr.) Kühner], *Ptychoverpabohemica* (Krombh.) Boud. = *Verpabohemica* (Krombh.) J. Schröt, and *Russulaochroleuca* (Pers.) Fr. (Plassmann 1971, Jakovlev 1994, Hackman and Meinander 1979). Larvae, determined with DNA barcodes, have been collected from fruitbody of *Cortinarius* sp.

Adults have frequently been reported hibernating in hollow, usually broken, umbelliferous stems (Plassmann 1971, Väisänen 1981, Kurina 1997) and we have also collected adults from broken stems of *Anthriscussylvestris* (L.) Hoffm. and *Heracleumpersicum* Desf. ex Fisch. at different localities in Norway during the winter season.

#### Taxon discussion

Chandler (1977) noticed two types of abdominal pattern in females matching the illustration of female terminalia of *E.parva* in Plassmann (1970). In our material, all female specimens of *E.parva* match the first type mentioned by Chandler (1977), with "[...] small yellow patches on tergites 3-6 not more than half tergal height in lateral view".

### Exechia penicillata

Lindemann sp. n.

BFAFEB6D-B3FE-581D-B2EA-70BDA14663C9

urn:lsid:zoobank.org:act:dbe1bdf0-280d-46eb-848d-db9e45a5e9e0

http://dx.doi.org/10.5883/BOLD:AEA7288

#### Materials

- **Type status:**Holotype. **Occurrence:** catalogNumber: TSZD-JKJ-107187; recordedBy: M. Mostovski; individualCount: 1; sex: male; lifeStage: adult; preparations: Pinned, with genitalia in glycerine in separate microvial; **Location:** country: South Africa; stateProvince: KwaZulu-Natal; municipality: Pietermaritzburg; locality: Karkloof Nat. Res.; verbatimElevation: 1325 m; decimalLatitude: -29.3169; decimalLongitude: 30.2514; **Event:** samplingProtocol: Malaise trap; eventDate: 2005-08-27; habitat: mistbelt forest; **Record Level:** institutionCode: TMU
- **Type status:**Paratype. **Occurrence:** catalogNumber: TSZD-JKJ-107188; recordedBy: M. Mostovski; individualCount: 1; sex: female; lifeStage: adult; preparations: Pinned, with genitalia in glycerine in separate microvial; **Location:** country: South Africa; stateProvince: KwaZulu-Natal; municipality: Pietermaritzburg; locality: Karkloof Nat. Res.; verbatimElevation: 1325 m; decimalLatitude: -29.3169; decimalLongitude: 30.2514; **Event:** samplingProtocol: Malaise trap; eventDate: 2005-08-27; habitat: mistbelt forest; **Record Level:** institutionCode: TMU

#### Description

Male: Body length 3.5 mm. Wing length 2.7 mm. **Colouration** (Dry specimen). Head and face dark brown; clypeus, labellum and palpus brown. Antenna with scape and pedicel yellow; flagellum brown. Thorax with scutum brown; lateral sclerites pale brown; propleura yellow; halteres whitish-yellow. Legs whitish-yellow. Abdomen dark brown, tergites II-IV with a lateroventral yellow area. Terminalia pale brown. **Head**. Frons and vertex covered with short, brown setae. Clypeus covered with dense thin pale setae. Antennae elongate, 2.3 times as long as length from vertex to ventral margin of clypeus ; flagellomeres slightly longer than broad, with sixth flagellomere 1.1 times as long as wide. **Thorax**. Scutum covered with short, brown setae. **Legs**. Fore leg with tibia and first tarsomere equally long. Mid-tibia with 20 anterior, 4 posterodorsal, 7 posterior and 3 posteroventral bristles. Hind tibia with 8 anterodorsal, 6 posterodorsal and 4 posterior bristles. **Wings**. Vein r-m 2.1 times longer than stem of M-fork. **Abdomen.** Tergites covered with pale brown setae. **Terminalia** (Fig. 29a, b, c). Each part of divided tergite IX apically with about 3 setae, most apical seta stout. Gonocoxites with setae on apicoventral margin elongate, reaching as far as, or beyond GL apex (Fig. 29a, b). GL apex with 2 setae (Fig. 29a). Aedaegal guides round (Fig. 29a). Hypandrium covered with about 9 setae, with apical pair elongate, reaching beyond GL apex (Fig. 29a). Hypandrial lobe with each branch wide medially, with narrow rounded apex (Fig. 29a). Gonostylus (Fig. 29c) with DB about 2.8 times longer than broad; apico-internal corner forming tapering acute apex extended apico-interiorly; apico-exterior corner right-angled, rounded; apical margin emarginate; dorsal side evenly covered with setae, except on most apical parts; VB oblong, with 2 small setae. IB apically rounded, apical part with 3 setae close to the apex. MB evenly tapered towards acute apex; bare.

Female: Body length 3 mm. Wing length 2.5 mm. **Colouration** (Dry specimen). Head with vertex dark brown; face and clypeus brown; labellum and palpus yellow. Antenna with scape and pedicel yellow; flagellum brown, basal part of first segment yellow. Thorax with scutum brown; lateral sclerites brown to pale brown; propleura yellow; halteres whitish-yellow. Legs whitish-yellow. Abdomen dark brown, tergites II-VI with lateral yellow areas. Terminalia brown. **Head**. Frons and vertex and clypeus covered with brown setae. Antenna 1.7 times as long as length from vertex to ventral margin of clypeus ; flagellomeres quadrate, with sixth flagellomere as long as broad. **Thorax**. Scutum covered with short dark brown setae. **Legs**. Mid-tibia with 22 anterior, 5 posterodorsal, 5 posterior and 2 posteroventral bristles. Hind tibia with 8 anterodorsal, 5 posterodorsal and 4 posterior bristles. **Wings**. Vein r-m 2.5 times longer than stem of M-fork. **Abdomen.** Tergites covered with brown setae. **Terminalia** (Fig. 29d, e). Cerci with apical segment length about 0.5 of basal segment length. Tergite VIII with apicolateral margin slightly angular. Sternite VII with apicoventral margin evenly rounded. Sternite VIII (Fig. 29d) with hypogynal valves separated by a cleft with depth about one-fifth of Sternite VIII length; apical setae about 0.7 of sternite VIII length. Gonapophysis IX (Fig. 29e) basolaterally expanding in abrupt almost right angle; spermathecal eminence distinctly trilobed with lateral branches curved distally.

#### Diagnosis

Distinguished from *E.sambai* and *E.ashleyi* in having the dorsal gonostylus branch 2.8 times longer than broad, with apico-internal corner acuminate and apico-external corner right-angled (Fig. 29c); from *E.burundiensis* in having the dorsal gonostylus branch apically emarginate (Fig. 29c) and the gonocoxal lobes with apical setae not splaying (Fig. 29a); from *E.afroparva* in having the gonocoxal lobes with parallel ventral margins (Fig. 29a); from other species in the *E.parva* group in having the internal branch with 3 setae close to the apex (Fig. 29c).

#### Etymology

From Latin *penicillus*, paint brush, relating to the shape and placement of setae on the internal lobe of the gonostylus, resembling a brush.

#### Distribution

Afrotropical, South Africa (Fig. 4)

#### Biology

Collected in mist-belt forest (1325 m a.s.l.).

### Exechia rectiloba

Lindemann sp. n.

1475EEDF-E52A-5CD4-B70F-4E0CE17D431C

urn:lsid:zoobank.org:act:faa157e1-c2c7-4018-a02e-6214fede532a

http://dx.doi.org/10.5883/BOLD:ADB8703

#### Materials

- **Type status:**Holotype. **Occurrence:** catalogNumber: TSZD-JKJ-102067; recordedBy: Jostein Kjærandsen; individualCount: 1; sex: male; lifeStage: adult; preparations: Pinned, with genitalia in glycerine in separate microvial; **Location:** island: Hokkaido; country: Japan; stateProvince: Hokkaido Prefecture; locality: Chitose-shi, Kokeno-domon Gallary beside Lake Shikotsu; verbatimElevation: 279 m; decimalLatitude: 42.712; decimalLongitude: 141.321; **Event:** samplingProtocol: sweep net; eventDate: 2006-10-02; **Record Level:** institutionCode: TMU

#### Description

Male: Body length 3.2 mm. Wing length 2.8 mm. **Colouration** (Dry specimen). Head, face and clypeus brown; labellum and palpus yellow. Antenna with scape and pedicel yellow; flagellum pale brown, basal half of first flagellomere pale. Thorax with scutum brown, anterior and lateral margin paler; lateral sclerites and propleura brown; halteres whitish-yellow. Legs whitish-yellow. Abdomen brown with a pale area covering ventral and apicolateral margins of tergite II and ventral and basolateral margins of tergite III, in dorsal view appearing as a pale band narrowly broken medially. Terminalia yellow. **Head**. Frons, vertex and clypeus covered with pale setae. Antenna 1.8 times as long as length from vertex to ventral margin of clypeus; flagellomeres quadrate, with sixth flagellomere as long as wide. **Thorax**. Scutum covered with pale setae. **Legs**. Fore leg with tibia 1.05 times as long as first tarsomere. Mid-tibia with 26 anterior, 3 posterodorsal, 7 posterior and 2 posteroventral bristles. Hind tibia with 7 anterodorsal, 4 posterodorsal and 6 posterior bristles. **Wings**. Vein r-m 2.7 times longer than stem of M-fork. **Abdomen.** Tergites covered with long, pale setae. **Terminalia** (Fig. 30). Each part of divided tergite IX with about 6-8 setae, most apical seta stout. GL (Fig. 30a, b) with length about 0.52 of gonocoxite width; apico-internal margin slightly angled exteriorly; basal tenth covered with setae; apex with 3 setae. Aedaegal guides short with acute apex. Hypandrium covered with about 13 setae, with apical pair reaching about half of the GL (Fig. 30a, b). Hypandrial lobe with each branch slender, apical half somewhat widened. Gonostylus (Fig. 30c) with DB about 2.2 times longer than broad, slightly curved interiorly; apical lobe well defined, about 0.22 of total DB length, apex acute; evenly covered with setae on dorsal side, except on the most apical part; external margin forming indistinct angle with row of 4 elongated setae. VB with 2 small setae. IB apically with 1 seta close to apex and pair of setae one-third from apex. MB apex acute, with 1 seta close to the base.

Female: Unknown.

#### Diagnosis

Distinguished from other species in the *E.parva* group in having the gonocoxal lobes extending almost without tapering, with only the basal tenth covered with setae (Fig. 30a, b), in combination with the dorsal gonostylus branch only about 2.2 times longer than broad (Fig. 30c) and the gonocoxal lobes with apico-internal margin slightly angled exteriorly (Fig. 30a, b).

#### Etymology

From Latin *rectus*, straight and *loba*, lobe, relating to the shape of the gonocoxal lobes.

#### Distribution

East Palaearctic, Japan (Fig. 10)

#### Biology

Unknown.

### Exechia repanda

Johannsen, 1912

6E0F1E50-1A27-5305-B5A4-65B750C1B19B

http://dx.doi.org/10.5883/BOLD:ABA6848

Exechia repanda Johannsen, 1912: 73 ♂♀ (Johannsen 1912)

#### Materials

- **Type status:**Holotype. **Occurrence:** catalogNumber: TTG-TBB-2048; recordedBy: O. A. Johannsen; individualCount: 1; sex: Male; lifeStage: adult; preparations: Pinned, with genitalia in glycerine in separate microvial; **Location:** country: USA; stateProvince: New York; municipality: Ithaca; **Event:** eventDate: 1894-08-28; **Record Level:** institutionCode: CUIC
- **Type status:**Other material. **Occurrence:** catalogNumber: BIOUG17656-D06; recordedBy: BIObus 2013; individualCount: 1; sex: female; lifeStage: adult; preparations: Pinned, with genitalia in glycerine in separate microvial; **Location:** country: Canada; stateProvince: Nova Scotia; locality: Kejimkujik National Park, Eel Weir Road; verbatimElevation: 96 m; decimalLatitude: 44.3482; decimalLongitude: -65.189; **Event:** samplingProtocol: Malaise Trap; eventDate: 2013-06-22; habitat: Wetland; fieldNotes: 2 malaise traps|mostly sunny|bog; **Record Level:** institutionCode: CBG
- **Type status:**Other material. **Occurrence:** catalogNumber: BIOUG31032-H11; recordedBy: Mary-Ann Mitchler; individualCount: 1; sex: female; lifeStage: adult; preparations: Pinned, with genitalia in glycerine in separate microvial; **Location:** country: Canada; stateProvince: Manitoba; municipality: Winnipeg; locality: Oakenwald School; verbatimElevation: 232 m; locationRemarks: EQP-CLL-551; decimalLatitude: 49.8459; decimalLongitude: -97.139; **Event:** samplingProtocol: Malaise Trap; eventDate: 2016-09-30; **Record Level:** institutionCode: CBG
- **Type status:**Other material. **Occurrence:** catalogNumber: BIOUG03446-H05; recordedBy: Tyler Peters; individualCount: 1; sex: male; lifeStage: adult; preparations: Pinned, with genitalia in glycerine in separate microvial; **Location:** country: Canada; stateProvince: Ontario; locality: Point Pelee National Park, Cactus Field; verbatimElevation: 168 m; decimalLatitude: 41.939; decimalLongitude: -82.516; **Event:** samplingProtocol: Malaise Trap; eventDate: 2012-07-04; habitat: Mixed Habitat; fieldNotes: Cedar / Savannah; **Record Level:** institutionCode: CBG
- **Type status:**Other material. **Occurrence:** catalogNumber: BIOUG27568-B05; recordedBy: BIO Collections Staff; individualCount: 1; sex: male; lifeStage: adult; preparations: Pinned, with genitalia in glycerine in separate microvial; **Location:** country: Canada; stateProvince: Ontario; municipality: Mississauga; locality: CRH Canada, 2391 Lakeshore Rd W; verbatimElevation: 87 m; locationRemarks: Site 3; decimalLatitude: 43.491; decimalLongitude: -79.618; **Event:** samplingProtocol: Intercept Trap; eventDate: 2015-10-15; habitat: Grassland; fieldNotes: 1 Intercept Trap|windy, sunny|grass, thistle; **Record Level:** institutionCode: CBG
- **Type status:**Other material. **Occurrence:** catalogNumber: BIOUG08568-B05; recordedBy: Barb Maschke; individualCount: 1; sex: Male; lifeStage: adult; preparations: Pinned, with genitalia in glycerine in separate microvial; **Location:** country: Canada; stateProvince: Ontario; municipality: Perth East; locality: Mornington Central School; verbatimElevation: 380 m; locationRemarks: EQP-CLL-556; decimalLatitude: 43.614; decimalLongitude: -80.885; **Event:** samplingProtocol: Malaise Trap; eventDate: 2013-10-04; **Record Level:** institutionCode: CBG
- **Type status:**Other material. **Occurrence:** catalogNumber: BIOUG05522-E07; recordedBy: Stacey Fraser; individualCount: 1; sex: Male; lifeStage: adult; preparations: Pinned, with genitalia in glycerine in separate microvial; **Location:** country: Canada; stateProvince: Ontario; municipality: Guelph; locality: St. James Catholic School; locationRemarks: EQP-CLL-592; decimalLatitude: 43.5596; decimalLongitude: -80.234; **Event:** samplingProtocol: Malaise Trap; eventDate: 2013-04-03; **Record Level:** institutionCode: CBG

#### Description

Male (n = 3): Body length 3.0-3.2 mm. Wing length 2.4-2.6 mm. **Colouration** (Dry specimen). Head, face and clypeus dark brown; labellum pale brown to yellow; palpus yellow. Antenna with scape and pedicel yellow; flagellum pale brown, basal half of first flagellomere pale. Thorax with scutum brown, sometimes anterior and lateral margin paler; lateral sclerites pale brown to brown; propleura yellow to pale brown; halteres whitish-yellow. Legs whitish-yellow. Abdomen brown, tergites II-III slightly paler than the rest or sometimes a distinct pale area covering lateral part of tergite II and anterolateral part of tergite III. Terminalia yellow to pale brown. **Head**. Frons, vertex and clypeus covered with pale setae. Antenna 1.83-1.93 times as long as length from vertex to ventral margin of clypeus; flagellomeres slightly longer than broad, with sixth flagellomere 1.0-1.1 times as long as wide. **Thorax**. Scutum covered with pale setae. **Legs**. Fore leg with tibia as long as first tarsomere. Mid-tibia with 20-22 anterior, 2-3 posterodorsal, 7 posterior and (n = 1) 1 posteroventral bristles. Hind tibia with 8-7 anterodorsal, 5 posterodorsal and 4 posterior bristles. **Wings**. Vein r-m 2.7-3 times longer than stem of M-fork. **Abdomen.** Tergites covered with pale setae. **Terminalia** (Figs 2b, 31a, b, c). Each part of divided tergite IX with about 7-9 setae, most apical seta stout. GL (Fig. 31a, b) with length 0.5-0.54 of gonocoxite width; apico-internal margin slightly angled exteriorly; basal half or third covered with setae; apex with 3-4 setae. Aedaegal guides short with acute apex. Hypandrium covered with 9-13 setae, with apical pair reaching about half of GL (Fig. 31a). Each part of hypandrial lobe narrow, apical half somewhat widened. Gonostylus (Fig. 31c) with DB 2.3-2.4 times longer than broad, curved interiorly; apical lobe well defined, apex rounded; evenly covered with setae on dorsal side, except on the most apical part; external margin forming a distinct angle, with row of 4-5 elongate setae. VB round, with 2 small setae. IB apically with 1 seta close to apex and pair of setae one-third from apex. MB apex acute, with 1 seta close to the base.

Female (n = 3): Body length 3.1-3.3 mm. Wing length 2.4-2.8 mm. **Colouration** (Dry specimen). Head, face and clypeus dark brown; labellum yellow to pale brown; palpus yellow. Antenna with scape and pedicel yellow; flagellum brown, basal part of first segment pale. Thorax with scutum dark brown, sometimes anterior and lateral margin paler; lateral sclerites brown; propleura yellow to brown; halteres whitish-yellow. Legs whitish-yellow. Abdomen brown to dark brown, tergites II-VI with pale lateral areas extending dorsally at anterior fifth to third of tergites III and IV, forming two more or less distinct pale bands. Terminalia pale brown. **Head**. Frons, vertex and clypeus covered with pale setae. Antenna 1.7-1.8 times as long as length from vertex to ventral margin of clypeus; flagellomeres quadrate or longer than broad, with sixth flagellomere 1.0-1.2 times as long as wide. **Thorax**. Scutum covered with pale setae. **Legs**. Fore leg with tibia (n = 2) 1.1-1.13 as long as first tarsomere. Mid-tibia with (n = 1) 20 anterior, (n = 1) 3 posterodorsal and (n = 1) 5 posterior bristles. Hind tibia with (n = 1) 6 anterodorsal, (n = 1) 4 posterodorsal and (n = 1) 4 posterior bristles. **Wings**. Vein r-m 1.5-2.8 times longer than stem of M-fork. **Abdomen.** Tergites covered with pale to pale brown setae. **Terminalia** (Fig. 31d, e). Cerci with apical segment half as long as basal segment. Tergite VIII with apicolateral margin slightly angular, not distinctly protruding (Fig. 31d). Sternite VII with apicoventral margin evenly rounded, sometimes with medial part very slightly protruding. Sternite VIII (Fig. 31e) with hypogynal valves separated by narrow cleft with depth about one-fifth of sternite VIII length; apical seta about 0.6-0.65 of sternite VIII length. Gonapophysis IX with basolateral part expanding in a relatively obtuse angle; spermathecal eminence trilobed, in ventral view appearing cross-shaped; apex with 8 small setae.

#### Diagnosis

Distinguished from *E.subrepanda* in having the dorsal gonostylus branch only 2.3-2.4 times longer than broad and with the external row of setae covering one-third to half of the total dorsal gonostylus branch length (Fig. 31c); from *E.neorepanda* in having the dorsal gonostylus branch more curved interiorly and with the external margin forming a more distinct angle (Fig. 31c); from *E.curvata* in having the dorsal gonostylus branch with exterior margin forming a less protruding angle, with external row of setae covering one-third to half of the total dorsal gonostylus branch length (Fig. 31c), in combination with the gonocoxal lobes more elongate; from other species in the *E.parva* group by the dorsal gonostylus branch 2.3-2.4 times longer than broad (Fig. 31c), in combination with the gonocoxal lobes covered with setae on basal half or less and with the apico-internal margin slightly angled exteriorly (Fig. 31a).

#### Distribution

Nearctic, Canada, USA (Fig. 13)

#### Biology

Adults collected in grassland, savannah and wetland habitats.

#### Taxon discussion

The species is very close to *E.neorepanda* and *E.subrepanda* and species determination should be conducted with care or by use of DNA barcoding. It has also been reported from Chokotka, East Russia (Polevoi and Barkalov 2017), but we have not considered these occurrences due to the risk of confusion with *E.neorepanda* or *E.subrepanda*. We assume that previous records of *E.repanda* in the West-Palaearctic Region constitute records of *E.neorepanda*.

#### Notes

Holotype in rather poor condition - both wings and left legs mounted on slide. Antennae almost intact (one tip lost). Terminalia in fairly good condition - transferred to glycerine in microvial and associated with pinned specimen.

### Exechia repandoides

Caspers, 1984

0BEFF25D-06E7-5861-B145-8F11ADE9E95C

Exechia repandoides Caspers, 1984: 180 ♂ (Caspers 1984)

#### Materials

- **Type status:**Holotype. **Occurrence:** recordedBy: H. Malicky; individualCount: 1; sex: male; lifeStage: adult; preparations: 80% Alcohol; **Location:** country: Austria; locality: Teichbach/Lunz; **Event:** samplingProtocol: "Emergenzfalle"; eventDate: 1972-09-22; **Record Level:** institutionCode: ZSMC
- **Type status:**Other material. **Occurrence:** catalogNumber: TSZD-JKJ-211754; recordedBy: G. Couturier; individualCount: 1; sex: male; lifeStage: adult; preparations: Pinned, with genitalia in glycerine in separate micro-vial; **Location:** country: France; county: Île-de-France; municipality: Versaille; locality: Val d'Or, La Miniére; decimalLatitude: 48.78; decimalLongitude: 2.08; **Event:** eventDate: 1970-06-05; habitat: Garden; fieldNotes: "au pied d'une haie"; **Record Level:** institutionCode: TMU
- **Type status:**Other material. **Occurrence:** catalogNumber: TSZD-JKJ-232387; recordedBy: Swedish Malaise Trap Project; individualCount: 1; sex: male; lifeStage: adult; preparations: Pinned, with genitalia in glycerine in separate microvial; **Location:** island: Öland; country: Sweden; county: Kalmar; municipality: Morbylonga; locality: Gamla Skogsby (Kallstad); decimalLatitude: 56.616700; decimalLongitude: 16.507617; **Event:** samplingProtocol: malaise trap; eventDate: 2003-09-12/2003-10-08; habitat: Meadow with bushes; fieldNotes: "diversitets-ängen" / trapID 22; **Record Level:** institutionCode: TMU
- **Type status:**Other material. **Occurrence:** catalogNumber: TSZD-JKJ-207679; recordedBy: H. Andersson & R. Danielsson; individualCount: 1; sex: male; lifeStage: adult; preparations: Slide; **Location:** island: Öland; country: Sweden; county: Kalmar; municipality: Borgholm; locality: Halltorps Hage; verbatimElevation: 25m; decimalLatitude: 56.800278; decimalLongitude: 16.576667; **Event:** samplingProtocol: window trap; eventDate: 1976-08-3/1976-08-6; habitat: Oak forest; **Record Level:** institutionCode: TMU

#### Description

Male (n = 3): Body length (n = 2) 3.0-3.3 mm. Wing length 2.8 mm. **Colouration** (Dry specimen; n = 2). Head pale brown to brown; labellum and palpus yellow. Antenna yellow. Scutum pale brown to brown, anterolaterally distinctly paler; lateral sclerites yellow to pale brown; halteres yellow. Legs yellow to whitish-yellow. Abdomen pale brown to brown, tergite II slightly paler. Terminalia pale brown. **Head**. Frons and vertex and clypeus covered with pale setae. Antenna 1.8-1.95 times as long as length from vertex to ventral margin of clypeus; flagellomeres elongate, with sixth flagellomere 1.25 times longer than broad. **Thorax**. Scutum covered with pale setae. **Legs**. Fore leg with tibia 0.96-1.0 times as long as first tarsomere. Mid-tibia with 20-24 anterior, 3-4 posterodorsal, 7-8 posterior and 2-3 posteroventral bristles. Hind tibia (n = 2) with 7-8 anterodorsal, 4-6 posterodorsal and 3-5 posterior bristles. **Wings**. Vein r-m 2.0-2.3 times longer than stem of M-fork. **Abdomen.** Tergites covered with pale setae. **Terminalia** (Fig. 32). Each part of divided tergite IX with 5 setae, most apical setae stout. GL (Fig. 32a, b) with length 0.54-0.58 of gonocoxite width; apico-internal margin slightly angled exteriorly; basal fourth covered with setae; apex with 3-4 setae. Aedaegal guides short, rounded. Hypandrium covered with 17-21 setae, with apical pair reaching about half of the GL (Fig. 32a, b). Hypandrial lobe with each branch slender, basal half somewhat widened, apical half narrow. Gonostylus (Fig. 32c) with DB 2.0-2.4 times longer than broad, distinctly curved interiorly, apical part broad, apex acute, apico-internally with short angular projection; evenly covered with setae on dorsal side, except on most apical part; apico-external margin slightly angled with row of 4-5 elongate setae. VB round, with 2 small setae. IB apically with 1 seta close to apex and pair of setae one-third from apex. MB apex acute, with 1 seta close to the base.

Female: Unknown.

#### Diagnosis

Distinguished from *E.sphaerata* in having the dorsal gonostylus branch apico-internally forming a short angular projection (Fig. 32d) and with the apex acute (Fig. 32c), in combination with the hypandrium more setose, covered with 17-21 setae (Fig. 32a, b); from *E.brevilobata* and *E.breviflagellata* in having the antennae 1.8-1.95 times as long as length from vertex to ventral margin of clypeus, in combination with the dorsal gonostylus branch without a distinct apical lobe (Fig. 32c), and the hypandrium more setose (Fig. 32a, b); from other species in the *E.parva* group in having a short dorsal gonostylus branch, 2.0-2.4 times longer than broad (Fig. 32c), in combination with the gonocoxal lobes with apico-internal margin angled exteriorly (Fig. 32a, b).

#### Distribution

West Palaearctic, Austria, Britain, Corsica, Czech Republic, Estonia, France, Germany, Hungary, Italy, Sweden, Switzerland (Fig. 15, Chandler 2005).

#### Biology

Reared from fruitbody of *Tricholomasejunctum* (Sowerby) Quél. and *Cortinarius* sp. (Šedivý and Ševčík 2003, Ševčík 2006). Adults collected in old growth deciduous forest.

#### Taxon discussion

The earlier records of this species from Jokkmokk, Sweden (Kjærandsen et al. 2007), have here been re-identified to *E.brevilobata.* The two species may have non-overlapping distributions, with *E.brevilobata* only recorded from boreal areas and *E.repandoides* only recorded from nemoral areas.

#### Notes

The pinned specimens have been preserved in alcohol for some years before being dry-mounted and may, therefore, be considerably paler compared to fresh material.

Holotype compared with and found to be conspecific with TSZD-JKJ-207679.

### Exechia rohdendorfi

Zaitzev, 1996

5ACF9945-B07B-5658-9F7A-C8A3B67AE5E2

http://dx.doi.org/10.5883/BOLD:AEA3741

Exechia rohdendorfi Zaitzev, 1996: 68 ♂ (Zaitzev 1996)

#### Materials

- **Type status:**Holotype. **Occurrence:** catalogNumber: Di0525; occurrenceRemarks: Two labels: Label 1 in transliteration:"Sakhalin, Neversk Distr., 20.IX.1986, A. Zaitzev" [in work with original description Neversk Distr. change on more exact place - Kuznetzov Cape]. Label 2: "Holotypus ? Exechiarohdendorfi Holotypus det. Zaitzev"; recordedBy: A. Zaitzev; individualCount: 1; sex: male; lifeStage: adult; preparations: Pinned; **Location:** island: Sakhalin; country: Russia; stateProvince: Sakhalin Oblast; locality: Neversk Distr., Kuznetzov Cape; **Event:** eventDate: 1986-09-20; **Record Level:** institutionCode: ZMM
- **Type status:**Other material. **Occurrence:** catalogNumber: TSZD-JKJ-109246; recordedBy: J. Kjærandsen; individualCount: 1; sex: male; lifeStage: adult; preparations: Pinned, with genitalia in glycerine in separate microvial; **Location:** island: Hokkaido; country: Japan; stateProvince: Hokkaido Prefecture; locality: Kushiro-shi, Middle-lower reach of Ibeshibetsu River near Lake Akan, Akan-cho; verbatimElevation: 427 m; locationRemarks: Site 2; decimalLatitude: 43.48083; decimalLongitude: 144.13917; **Event:** samplingProtocol: sweep net; eventDate: 2006-10-04; **Record Level:** institutionCode: TMU
- **Type status:**Other material. **Occurrence:** catalogNumber: TSZD-JKJ-233424; recordedBy: J. Kjærandsen; individualCount: 1; sex: male; lifeStage: adult; preparations: Pinned, with genitalia in glycerine in separate microvial; **Location:** island: Hokkaido; country: Japan; stateProvince: Hokkaido Prefecture; locality: Kushiro-shi, Middle reach of Ibeshibetsu River near Lake Akan, Akan-cho; verbatimElevation: 448 m; locationRemarks: Site 3; decimalLatitude: 43.48806; decimalLongitude: 144.14778; **Event:** samplingProtocol: sweep net; eventDate: 2006-10-04; **Record Level:** institutionCode: TMU
- **Type status:**Other material. **Occurrence:** catalogNumber: TSZD-JKJ-107245; recordedBy: J. Kjærandsen; individualCount: 1; sex: male; lifeStage: adult; preparations: Pinned, with genitalia in glycerine in separate microvial; **Location:** island: Hokkaido; country: Japan; stateProvince: Hokkaido Prefecture; locality: Chitose-shi, Chitose-gawa at Rankoshi; verbatimElevation: 45 m; decimalLatitude: 42.805; decimalLongitude: 141.567; **Event:** samplingProtocol: sweep net; eventDate: 2006-10-02; **Record Level:** institutionCode: TMU
- **Type status:**Other material. **Occurrence:** catalogNumber: TSZD-JKJ-232387; recordedBy: J. Kjærandsen; individualCount: 1; sex: male; lifeStage: adult; preparations: Pinned, with genitalia in glycerine in separate microvial; **Location:** island: Hokkaido; country: Japan; stateProvince: Hokkaido Prefecture; locality: Chitose-shi, Chitose-gawa at Rankoshi; verbatimElevation: 45 m; decimalLatitude: 42.805; decimalLongitude: 141.567; **Event:** samplingProtocol: sweep net; eventDate: 2006-10-02; **Record Level:** institutionCode: TMU

#### Description

Male (n = 4): Body length 3.4-3.6 mm. Wing length 3-3.1 mm. **Colouration** (Dry specimen). Head brown to dark brown; face yellow to brown; clypeus pale brown; labellum yellow; palpus yellow. Antenna with scape and pedicel yellow; flagellum yellow to brown, first segment with yellow base. Scutum pale brown with a narrow yellow anterolateral margin; lateral sclerites pale brown; propleura whitish-yellow; halteres whitish-yellow, apically slightly darker. Legs whitish-yellow. Abdomen pale brown to brown, tergite III sometimes with a small yellow lateral spot. Terminalia pale brown with MD and apico-internal lobe of DB dark brown (Fig. 33b). **Head**. Vertex, frons and clypeus covered with brown setae. Antenna (n = 2) 1.9-2 times as long as length from vertex to ventral margin of clypeus ; flagellomeres slightly longer than broad, with sixth flagellomere 1.1 times as long as wide. **Thorax**. Scutum covered with pale brown setae. **Legs**. Fore leg with tibia 0.77-0.92 times length of first tarsomere. Mid-tibia with 24 anterior, 4-5 posterodorsal, 9-11 posterior and 6-7 posteroventral bristles. Hind tibia with 14-10 anterodorsal, 6-5 posterodorsal and 6-5 posterior bristles. **Wings**. Vein r-m (n = 2) 2.7-3.3 times longer than stem of M-fork. **Abdomen.** Tergites covered with brown setae. **Terminalia** (Fig. 33). Each part of divided tergite IX with about 2-5 setae, most apical seta stout. GL relatively narrow, length about one-third of gonocoxite width, apex with 2-3 elongate setae, otherwise bare (Fig. 33a, b). Aedaegal guides narrow, elongate, apex acute (Fig. 33a). Hypandrium covered with about 6-12 setae, with apical pair reaching about level of GL apex (Fig. 33a). Hypandrial lobe with each branch elongate, narrow, apically abruptly curved interiorly, apex rounded. Gonostylus (Fig. 33c) with DB short, wide, forming a large, darkened apico-internal lobe; dorsal side evenly covered with setae, except on apico-internal lobe; apico-externally somewhat acute with 1-2 long setae. VB large, apically acute, with 1 long seta on apex and 3-4 shorter setae further down, one somewhat wider than others. Apical part of IB with 1 seta on the apex and row of 4 setae on shelf close to apex. MB short, wide, apex narrow and truncate, internally forming elongate, more or less acute process extending interiorly; apical margin with 6-7 long setae, all longer than MB length.

Female: Unknown.

#### Diagnosis

Distinguished from *E.toyoheii* in having the hypandrium with apical pair of setae elongate (Fig. 33a), the internal gonostylus branch with apical seta and subapical row of setae close together (Fig. 33c) and the gonocoxal apicoventral margin without distinct protrusions (Fig. 33a); from other species in the *E.parva* group by the dorsal gonostylus branch short with a large darkened internal lobe (Fig. 33c), in combination with the medial gonostylus branch darkened (Fig. 33b), apically with a row of 3-4 setae, all of which are longer than the medial gonocoxal branch length (Fig. 33c).

#### Distribution

East Palaearctic, Japan, Russia (Fig. 10)

#### Biology

Unknown

#### Taxon discussion

We have recognised one specimen (TSZD-JKJ-108464) with slightly deviating shape of the medial and internal gonostylus branch (Fig. 33c, d). Taking into consideration the similarity in the CO1 sequence between the two forms, as well as being sympatric, they probably belong to the same species.

#### Notes

We have studied the holotype, based on images of the terminalia, provided by courtesy of Dr. Andrey Ozerov, Moscow State University. The holotype is in good condition and terminalia not dissected.

### Exechia sambai

Lindemann sp. n.

B94D46A7-572D-50FE-8121-DDE0CCAE4D6F

urn:lsid:zoobank.org:act:dad1c233-a38a-48f5-9bed-fcecc64710dd

http://dx.doi.org/10.5883/BOLD:AEA7289

#### Materials

- **Type status:**Holotype. **Occurrence:** catalogNumber: TSZD-JKJ-107177; recordedBy: J. Kjærandsen; individualCount: 1; sex: male; lifeStage: adult; preparations: Pinned, with genitalia in glycerine in separate microvial; **Location:** country: Kenya; stateProvince: Nyeri county; locality: Mt. Kenya, Northern Naro Moru, Base camp at Naro Moru River Lodge; verbatimElevation: 3050 m; decimalLatitude: -0.17028; decimalLongitude: 37.215; **Event:** samplingProtocol: sweep net; eventDate: 2008-08-19; habitat: bamboo forest; **Record Level:** institutionCode: TMU
- **Type status:**Paratype. **Occurrence:** catalogNumber: TSZD-JKJ-107178; recordedBy: J. Kjærandsen; individualCount: 1; sex: female; lifeStage: adult; preparations: Pinned, with genitalia in glycerine in separate microvial; **Location:** country: Kenya; stateProvince: Nyeri county; locality: Mt. Kenya, Northern Naro Moru, Base camp at Naro Moru River Lodge; verbatimElevation: 3050 m; decimalLatitude: -0.17028; decimalLongitude: 37.215; **Event:** samplingProtocol: sweep net; eventDate: 2008-08-19; habitat: bamboo forest; **Record Level:** institutionCode: TMU
- **Type status:**Paratype. **Occurrence:** catalogNumber: TSZD-JKJ-107176; recordedBy: J. Kjærandsen; individualCount: 1; sex: male; lifeStage: adult; preparations: Pinned, with genitalia in glycerine in separate microvial; **Location:** country: Kenya; stateProvince: Nyeri county; locality: Mt. Kenya, Northern Naro Moru, Base camp at Naro Moru River Lodge; verbatimElevation: 3050 m; decimalLatitude: -0.17028; decimalLongitude: 37.215; **Event:** samplingProtocol: sweep net; eventDate: 2008-08-19; habitat: bamboo forest; **Record Level:** institutionCode: TMU

#### Description

Male (n = 2): Body length 3.3-3.4 mm. Wing length 2.8-2.9 mm. **Colouration** (Dry specimen). Head and face brown; clypeus pale brown; labellum yellow; palpus yellow to whitish-yellow. Antenna with scape and pedicel yellow; flagellum pale brown to brown, basal half of first flagellomere pale. Thorax with scutum pale brown, anterior and lateral margin slightly paler; lateral sclerites and propleura pale brown; halteres whitish-yellow. Legs whitish-yellow. Abdomen brown. Terminalia yellow. **Head**. Frons and vertex covered with pale setae. Clypeus covered with 13-16 pale setae, evenly distributed. Antenna long, 2.0-2.25 times as long as length from vertex to ventral margin of clypeus; flagellomeres longer than broad, with sixth flagellomere 1.2-1.3 times as long as wide. **Thorax**. Scutum covered with short, pale setae. **Legs**. Fore leg with tibia 0.91-0.95 times as long as first tarsomere. Mid-tibia with 22-23 anterior, 3-5 posterodorsal, 5-7 posterior and 2-4 posteroventral bristles. Hind tibia with 6-8 anterodorsal, 4 posterodorsal and 3-5 posterior bristles. **Wings**. Vein r-m 2.75-3 times longer than stem of M-fork. **Abdomen.** Tergites covered with pale setae. **Terminalia** (Fig. 34a, b, c). Each part of divided tergite IX apically with about 6 setae, most apical seta stout. Gonocoxites (Fig. 34a, b) with setae on apicoventral margin elongate, reaching far beyond GL apex. GL apex with 2 setae (Fig. 34a, b). Aedaegal guides acuminate (Fig. 34a). Hypandrium covered with about 11 setae, with apical pair elongate, reaching far beyond GL apex (Fig. 34a, b). Hypandrial lobe with each branch short, evenly tapered, apex acute (Fig. 34a). Gonostylus (Fig. 34c) with DB short and squared; about 2 times longer than broad; apico-internal corner acuminate, extended apico-interiorly; apico-exterior corner forming rounded lobe, extended apico-exteriorly; apical margin emarginate; dorsal side evenly covered with setae, except on apical third; VB apically acuminate, with 2 setae, most apical seta on apex. IB apically rounded; apically with 3 setae close to apex. MB apically curved interiorly with apex acute; apically with 2 small setae.

Female: Body length 3.5 mm. Wing length 3.2 mm. **Colouration** (Dry specimen). Head, face and clypeus brown; labellum and palpus yellow. Antenna with scape and pedicel yellow; flagellum pale brown, basal part of first flagellomere pale. Thorax with scutum, lateral sclerites and propleura pale brown; halteres whitish-yellow. Legs whitish-yellow. Abdomen brown, tergites I-IV with pale lateral areas. Terminalia yellow. **Head**. Frons and vertex covered with short pale setae. Clypeus covered with pale setae, evenly distributed. Antenna 2 times as long as length from vertex to ventral margin of clypeus. **Thorax**. Scutum covered with short pale setae. **Legs**. Fore leg with tibia 0.92 times as long as first tarsomere. Mid-tibia with 24 anterior, 4 posterodorsal, 8 posterior and 3 posteroventral bristles. Hind tibia with 8 anterodorsal, 5 posterodorsal and 4 posterior bristles. **Wings**. Vein r-m 2.9 times longer than stem of M-fork. **Abdomen.** Tergites covered with dark brown setae. **Terminalia** (Fig. 34d, e). Cerci with length of apical segment about two-thirds of basal segment. Tergite VIII with apicolateral margin weakly curved. Sternite VII with apicoventral margin acuminate. Sternite VIII (Fig. 34d) in ventral view square shaped, equally broad along most of length, except abruptly tapered apical part; two large bare areas extending into basal part of setae cover; hypogynal valves separated by wide cleft with depth about one-seventh of sternite VIII length; apical seta about 0.6 of sternite VIII length. Gonapophysis IX (Fig. 34e) with basolateral part abruptly expanded forming distally projecting lobe; spermathecal eminence weakly trilobed.

#### Diagnosis

Distinguished from *E.penicillata* in having the dorsal gonostylus branch 2 times longer than broad and with the apico-external corner extended into a rounded lobe (Fig. 34c); from *E.ashleyi* in having the dorsal gonostylus branch with apico-internal corner extended and acuminate (Fig. 34c) and the gonocoxites with setae on apicoventral margin reaching far beyond the gonocoxal lobe apex (Fig. 34a, b); from *E.burundiensis* in having the dorsal gonostylus branch apically emarginate (Fig. 34c) and the gonocoxal lobes with apical setae parallel, not splaying (Fig. 34a, b); from *E.afroparva* in having the gonocoxal lobes with parallel ventral margins (Fig. 34a, b); from other species in the *E.parva* group in having the internal branch of the gonostylus with 3 setae close to the apex (Fig. 34c).

#### Etymology

Named in honour of the contemporary artist and painter Chéri Samba, relating to the shape of the internal lobe of the gonostylus, resembling a paintbrush.

#### Distribution

Afrotropical, Kenya (Fig. 4)

#### Biology

Adult collected in bamboo forest (3050 m a.s.l.)

### Exechia serrae

Lindemann sp. n.

C2B55C9C-16E2-5669-959F-877443CDBF62

urn:lsid:zoobank.org:act:2e0af56c-4573-474e-aa12-f18ba98c3ea9

#### Materials

- **Type status:**Holotype. **Occurrence:** catalogNumber: TSZD-JKJ-111561; recordedBy: A. Nakanishi; individualCount: 1; sex: male; lifeStage: adult; preparations: Pinned, with genitalia in glycerine in separate microvial; **Location:** country: Nepal; stateProvince: Gandaki Pradesh; county: Myagdi District; locality: Mudi, Dobang Kharka; verbatimElevation: 2400 m; decimalLatitude: 28.6000; decimalLongitude: 83.4000; **Event:** eventDate: 1971-10-23; **Record Level:** institutionCode: KUEC

#### Description

Male: Body length 3.5 mm. Wing length 3.2 mm. **Colouration** (Dry specimen). Head, face and clypeus brown; labellum and palpus yellow. Antenna with scape and pedicel yellow; flagellum brown. Scutum and lateral sclerites pale brown; propleura yellow; halteres whitish-yellow. Legs whitish-yellow. Abdomen dark brown, tergites II-III with a lateral pale area. Terminalia yellow. **Head**. Frons and vertex covered with short pale setae. Clypeus covered with pale setae. Antenna 2 times as long as length from vertex to ventral margin of clypeus; flagellomeres longer than broad, with sixth flagellomere 1.4 times as long as wide. **Thorax**. Scutum covered with short, pale setae. **Legs**. Fore leg with tibia 0.78 times as long as first tarsomere. Mid-tibia with 18 anterior, 4 posterodorsal, 10 posterior and 4 posteroventral bristles. Hind tibia with 8 anterodorsal, 3 posterodorsal and 4 posterior bristles. **Wings**. Vein r-m 3.7 times longer than stem of M-fork. **Abdomen.** Tergites covered with pale setae. **Terminalia** (Fig. 35). Each part of divided tergite IX apically with about 3 setae, 2 most apical setae stout. GL (Fig. 35a, b) with entire length covered with setae; apex with 2 setae. Aedaegal guides elongate and spathulate (Fig. 35a). Hypandrium (Fig. 35a, b) strongly extended distally, forming large lobe that reaches far beyond apicoventral gonocoxal margin; covered with about 12 setae, with apical pair stout and reaching far beyond the level of the GL apex. Hypandrial lobe with each branch elongate, narrow, evenly tapered, apex acute (Fig. 35a). Gonostylus (Fig. 35c) with DB forming slender distally projecting baso-external lobe; basal part squared, abruptly tapered distally, apical part slender elongate, apex somewhat spathulate; basodorsal part evenly covered with setae; baso-external lobe with 2 apical setae, one on apex. VB small, apex rounded, with 3 setae, 2 most apical setae close to apex. IB with apical half slender, apex forming hyaline membranous area; apically with 1 seta close to apex and row of 3 setae on shelf one-third from apex. MB large, elongate; internal margin with few large teeth; apically hollowed with small seta within hollow area close to apex.

Female: Unknown.

#### Diagnosis

Distinguished from *E.columna* in having the gonocoxal lobes not reaching beyond the apical hypandrial setae (Fig. 35a, b) and the medial gonostylus branch with internal margin forming several large teeth (Fig. 35c); from other species in the *E.parva* group by the hypandrium forming a distally-extended lobe that reaches far beyond apicoventral gonocoxal margin (Fig. 35a, b) and the dorsal gonostylus branch forming a baso-external lobe with two apical setae (Fig. 35c).

#### Etymology

From Latin *serra*, saw, relating to the shape of the medial lobe of the gonostylus.

#### Distribution

Oriental, Nepal (Fig. 19).

#### Biology

Adult collected in the eastern Himalayas (2500 m a.s.l.).

### Exechia spatulata

Lindemann sp. n.

5105096D-7D9A-596D-8944-A7FF635C58EA

urn:lsid:zoobank.org:act:05e60b65-31c0-4583-8580-ff712f5aa0a3

http://dx.doi.org/10.5883/BOLD:ACC3725

#### Materials

- **Type status:**Holotype. **Occurrence:** catalogNumber: TSZD-JKJ-111444; recordedBy: Jarl Birkeland; individualCount: 1; sex: male; lifeStage: adult; preparations: Pinned; **Location:** country: Norway; county: Rogaland; municipality: Sokndal; locality: Skittmyr; decimalLatitude: 58.350488; decimalLongitude: 6.3054945; **Event:** samplingProtocol: Malaise trap; eventDate: 2020-05-21/2020-06-11; **Record Level:** collectionID: TMU-JKJ-COL-001514; institutionCode: TMU
- **Type status:**Paratype. **Occurrence:** catalogNumber: TSZD-JKJ-103590; occurrenceRemarks: TMU-JKJ fieldwork; recordedBy: Jostein Kjærandsen & Martin T. Dahl; individualCount: 1; sex: male; lifeStage: adult; preparations: Pinned, with genitalia in glycerine in separate microvial; **Location:** country: Norway; county: Finnmark; municipality: Sør-Varanger; locality: Gjøkbekken, Pasvik; verbatimElevation: 72 m; locationRemarks: SLAM 1; decimalLatitude: 69.1522; decimalLongitude: 29.1516; **Event:** samplingProtocol: SLAM trap; eventDate: 2017-08-25; **Record Level:** collectionID: TMU-JKJ-COL-000538; institutionCode: TMU
- **Type status:**Paratype. **Occurrence:** catalogNumber: TSZD-JKJ-107937; occurrenceRemarks: ADB - M&M project; recordedBy: Kai Berggren; individualCount: 1; sex: male; lifeStage: adult; preparations: Pinned, with genitalia in glycerine in separate microvial; **Location:** country: Norway; county: Vest-Agder; municipality: Kristiansand; locality: Nedre Jegersbergvann; verbatimElevation: 21 m; locationRemarks: MT 3, at lake; decimalLatitude: 58.169; decimalLongitude: 8; **Event:** samplingProtocol: Malaise trap; eventDate: 2019-06-29; **Record Level:** collectionID: TMU-JKJ-COL-001099; institutionCode: TMU
- **Type status:**Paratype. **Occurrence:** catalogNumber: TSZD-JKJ-107987; occurrenceRemarks: Gift from collector; recordedBy: J. Birkeland; individualCount: 1; sex: male; lifeStage: adult; preparations: Pinned, with genitalia in glycerine in separate microvial; **Location:** country: Norway; county: Rogaland; municipality: Sokndal; locality: Skittmyr; verbatimElevation: 22 m; decimalLatitude: 58.351; decimalLongitude: 6.306; **Event:** samplingProtocol: Malaise trap; eventDate: 2019-10-02; **Record Level:** collectionID: TMU-JKJ-COL-001079; institutionCode: TMU
- **Type status:**Paratype. **Occurrence:** catalogNumber: TSZD-JKJ-107932; occurrenceRemarks: Depth: 7.5 | ADB - M&M project; recordedBy: K. Berggren; individualCount: 1; sex: male; lifeStage: adult; preparations: Pinned, with genitalia in glycerine in separate microvial; **Location:** country: Norway; county: Vest-Agder; municipality: Kristiansand; locality: Nedre Jegersbergvann; verbatimElevation: 21 m; locationRemarks: MT 3, at lake; decimalLatitude: 58.169; decimalLongitude: 8; **Event:** samplingProtocol: Malaise trap; eventDate: 2019-08-28; **Record Level:** collectionID: TMU-JKJ-COL-001101; institutionCode: TMU
- **Type status:**Paratype. **Occurrence:** catalogNumber: TSZD-JKJ-111547; recordedBy: H. Andersson; individualCount: 1; sex: male; lifeStage: adult; preparations: Slide; **Location:** country: Sweden; county: Norrbotn; municipality: Luleå; locality: Råneå, Högsön, Högsöfjärden; **Event:** eventDate: 1965-09-06; **Record Level:** institutionCode: TMU
- **Type status:**Paratype. **Occurrence:** catalogNumber: SPM-060717; recordedBy: M. Söderlund; individualCount: 1; sex: male; lifeStage: adult; preparations: Pinned, with genitalia in glycerine in separate microvial; **Location:** country: Sweden; county: Västra Götaland; municipality: Ale; locality: Kollanda mosse; locationRemarks: MT 2; decimalLatitude: 57.940833; decimalLongitude: 12.249444; **Event:** samplingProtocol: Malaise trap; eventDate: 1995-09-11/1995-09-20; **Record Level:** institutionCode: TMU
- **Type status:**Paratype. **Occurrence:** catalogNumber: SPM-060720; recordedBy: M. Söderlund; individualCount: 1; sex: male; lifeStage: adult; preparations: Pinned, with genitalia in glycerine in separate microvial; **Location:** country: Sweden; county: Västra Götaland; municipality: Ale; locality: Kollanda mosse; locationRemarks: MT 2; decimalLatitude: 57.940833; decimalLongitude: 12.249444; **Event:** samplingProtocol: Malaise trap; eventDate: 1995-10-01/1995-10-10; **Record Level:** collectionID: COL-009616; institutionCode: TMU
- **Type status:**Paratype. **Occurrence:** catalogNumber: SPM-036986; recordedBy: G. Nilsson; individualCount: 1; sex: male; lifeStage: adult; preparations: Pinned, with genitalia in glycerine in separate microvial; **Location:** country: Sweden; county: Västmanland; municipality: Västerås; locality: Solbacken; decimalLatitude: 59.517222; decimalLongitude: 16.740000; **Event:** samplingProtocol: Malaise trap; eventDate: 1990-08-25/1990-09-09; **Record Level:** collectionID: COL-008186; institutionCode: TMU
- **Type status:**Paratype. **Occurrence:** catalogNumber: TSZD-JKJ-111490; recordedBy: J. Birkeland; individualCount: 1; sex: male; lifeStage: adult; preparations: Pinned; **Location:** country: Norway; county: Rogaland; municipality: Sokndal; locality: Skittmyr 2; decimalLatitude: 58.3501773; decimalLongitude: 6.3055563; **Event:** samplingProtocol: Malaise trap; eventDate: 2020-08-08/2020-09-05; **Record Level:** collectionID: TMU-JKJ-COL-001525; institutionCode: TMU
- **Type status:**Paratype. **Occurrence:** catalogNumber: TSZD-JKJ-111221; recordedBy: J. Kjærandsen; individualCount: 1; sex: male; lifeStage: adult; preparations: Pinned; **Location:** country: Norway; county: Trřndelag; municipality: Oppdal; locality: Dovre, Grønbakken; decimalLatitude: 62.278890; decimalLongitude: 9.59833; **Event:** samplingProtocol: Sweep net; eventDate: 2020-07-07; **Record Level:** collectionID: TMU-JKJ-COL-001481; institutionCode: TMU
- **Type status:**Paratype. **Occurrence:** catalogNumber: TSZD-JKJ-111439; recordedBy: J. Birkeland; individualCount: 1; sex: male; lifeStage: adult; preparations: Pinned; **Location:** country: Norway; county: Rogaland; municipality: Sokndal; locality: Skittmyr 2; decimalLatitude: 58.3501773; decimalLongitude: 6.3055563; **Event:** samplingProtocol: Malaise trap; eventDate: 2020-07-10/2020-08-08; **Record Level:** collectionID: TMU-JKJ-COL-001524; institutionCode: TMU

#### Description

Male (n = 12): Body length 2.9-3.2 mm. Wing length 2.4-2-5 mm. **Colouration** (Dry specimen). Head, face and clypeus dark brown; labellum dark brown; palpus yellow. Antenna with scape and pedicel yellow; flagellum dark brown, basal part of first flagellomere pale. Thorax with scutum, lateral sclerites and propleura dark brown; halteres whitish-yellow. Legs yellow. Abdomen dark brown. Terminalia brown. **Head**. Vertex, frons and clypeus covered with pale setae. Antenna 1.78 times as long as length from vertex to ventral margin of clypeus; flagellomeres quadrate, with sixth flagellomere as long as broad. **Thorax**. Scutum covered with pale setae. **Legs** (n = 3). Fore leg with tibia as long as first tarsomere. Mid-tibia with 20 anterior, 3 posterodorsal, 4 posterior and 2 posteroventral bristles. Hind tibia with 6 anterodorsal, 5 posterodorsal and 3 posterior bristles. **Wings**. Vein r-m 2.8 times longer than stem of M-fork. **Abdomen.** Tergites covered with pale setae. **Terminalia** (n = 4, Fig. 36a, b). Each part of divided tergite IX with about 3-4 setae, most apical seta stout. GL with length 0.6-0.75 of gonocoxite width (Fig. 36a, b); apico-internal margin slightly angled exteriorly; basal third covered with setae; apex with 3 setae. Aedaegal guides short and acute. Hypandrium covered with 10-12 setae, with apical pair reaching two thirds to three-fourths of GL (Fig. 36a, b). Hypandrial lobe with each part slender, apical half slightly widened. Gonostylus (Fig. 36c) with DB elongate, about 3.52 times longer than broad, virtually straight or very slightly curved interiorly, apical lobe distinctly spathulate, apex rounded; dorsal side evenly covered with setae, except on most apical part; external margin almost straight, not forming any angle, with row of 4 elongate setae. VB with 2 small setae. IB apically with 1 seta close to apex and pair of setae one-third from apex. MB apex acute, with 1 seta close to the base.

Female: Unknown.

#### Diagnosis

Distinguished from *E.longilobata* in having the dorsal gonostylus branch apically spathulate and about 3.5 times longer than broad (Fig. 36c), the hypandrium less setose with 10-12 setae (Fig. 36a, b), each part of the hypandrial lobe with apical half only slightly widened and the gonocoxal lobe length 0.6-0.75 the gonocoxite width (Fig. 36c); from other species in the *E.parva* group in having the dorsal branch spathulate and more elongated (Fig. 36c), in combination with the gonocoxal lobes with apico-internal margin slightly angled exteriorly (Fig. 36a).

#### Etymology

From Latin *spatula*, flat piece, relating to the the apical shape of the dorsal branch of the gonostylus.

#### Distribution

West Palaearctic, Norway, Sweden (Fig. 15)

#### Biology

Unknown.

### Exechia sphaerata

Lindemann sp. n.

223CFFF4-3F74-592C-9284-3C492E29B2BB

urn:lsid:zoobank.org:act:4e118a36-2b68-4f95-9e97-e71c913f1efa

http://dx.doi.org/10.5883/BOLD:ACC3725

#### Materials

- **Type status:**Holotype. **Occurrence:** catalogNumber: BIOUG03526-D01; recordedBy: BIOBus 2012; individualCount: 1; sex: male; lifeStage: adult; preparations: Pinned; **Location:** country: Canada; stateProvince: Alberta; locality: Banff National Park, Storm Mountain, Adjacent to train track and Bow River; verbatimElevation: 1462 m; decimalLatitude: 51.282; decimalLongitude: -115.944; **Event:** samplingProtocol: Intercept Trap; eventDate: 2012-06-20; habitat: Forest; fieldNotes: 1 Intercept Trap|mostly cloudy and 16C when site was dismantled|low alpine dry slope, temperate forest; **Record Level:** institutionCode: CBG
- **Type status:**Paratype. **Occurrence:** catalogNumber: BIOUG09297-H08; recordedBy: BIOBus 2012; individualCount: 1; sex: female; lifeStage: adult; preparations: Pinned; **Location:** country: Canada; stateProvince: Alberta; locality: Jasper National Park, Pocahontas Campground; verbatimElevation: 1131 m; locationRemarks: Site C21; decimalLatitude: 53.195; decimalLongitude: -117.914; **Event:** samplingProtocol: Pan Trap; eventDate: 2012-07-21; habitat: Forest; fieldNotes: 10 pan traps|overcast|16C|birch and spruce forest on a slope, lots of fallen logs; **Record Level:** institutionCode: CBG
- **Type status:**Paratype. **Occurrence:** catalogNumber: BIOUG06332-G11; recordedBy: BIOBus 2012; individualCount: 1; sex: male; lifeStage: adult; preparations: Pinned; **Location:** country: Canada; stateProvince: Alberta; locality: Banff National Park, Baker Creek picnic area, Adjacent to hydro line; verbatimElevation: 1500 m; decimalLatitude: 51.35; decimalLongitude: -116.063; **Event:** samplingProtocol: Sweep Net; eventDate: 2012-07-27; habitat: Forest; fieldNotes: standard 5 min sweeping (3), 4 sweepers|partly cloudy|Mature forest, lodgepole pine, temperate forest; **Record Level:** institutionCode: CBG
- **Type status:**Paratype. **Occurrence:** catalogNumber: BIOUG13692-D07; recordedBy: BIObus 2013; individualCount: 1; sex: male; lifeStage: adult; preparations: Pinned; **Location:** country: Canada; stateProvince: New Brunswick; locality: Fundy National Park, Maple Grove Trail; verbatimElevation: 212 m; decimalLatitude: 45.59; decimalLongitude: -64.985; **Event:** samplingProtocol: Intercept Trap; eventDate: 2013-05-30; habitat: Forest; fieldNotes: 1 Intercept Trap|overcast|hardwood forest, maple stand; **Record Level:** institutionCode: CBG
- **Type status:**Paratype. **Occurrence:** catalogNumber: BIOUG27489-F12; recordedBy: BIObus 2014; individualCount: 1; sex: male; lifeStage: adult; preparations: Pinned; **Location:** country: Canada; stateProvince: Yukon Territory; locality: Kluane National Park and Reserve, Dezadeash River Trail; verbatimElevation: 582 m; decimalLatitude: 60.748; decimalLongitude: -137.513; **Event:** samplingProtocol: Intercept trap; eventDate: 2014-07-24; habitat: Wetland; fieldNotes: 1 Intercept Trap|cold and overcast on day of collection|Wetland with grasses and shrubs; **Record Level:** institutionCode: CBG
- **Type status:**Paratype. **Occurrence:** catalogNumber: BIOUG13825-B01; recordedBy: BIObus 2013; individualCount: 1; sex: female; lifeStage: adult; preparations: Pinned; **Location:** country: Canada; stateProvince: New Brunswick; locality: Fundy National Park, Maple Grove Trail; verbatimElevation: 212 m; decimalLatitude: 45.59; decimalLongitude: -64.985; **Event:** samplingProtocol: Sweep Net; eventDate: 2013-05-28; habitat: Forest; fieldNotes: 5 min sweep x4 collectors (3)|sunny and clear skies|hardwood forest, maple stand; **Record Level:** institutionCode: CBG
- **Type status:**Paratype. **Occurrence:** catalogNumber: BIOUG09168-H01; recordedBy: BIOBus 2012; individualCount: 1; sex: male; lifeStage: adult; preparations: Pinned; **Location:** country: Canada; stateProvince: Alberta; locality: Jasper National Park, Pocahontas Campground; verbatimElevation: 1131 m; locationRemarks: Site C21; decimalLatitude: 53.195; decimalLongitude: -117.914; **Event:** samplingProtocol: Intercept Trap; eventDate: 21-Jul-2012; habitat: Forest; fieldNotes: 1 intercept trap|overcast|16C|birch and spruce forest on a slope, lots of fallen logs; **Record Level:** institutionCode: CBG
- **Type status:**Paratype. **Occurrence:** catalogNumber: BIOUG09164-G05; recordedBy: BIOBus 2012; individualCount: 1; sex: male; lifeStage: adult; preparations: Pinned; **Location:** country: Canada; stateProvince: Alberta; locality: Jasper National Park, Pocahontas Campground; verbatimElevation: 1131 m; locationRemarks: Site C21; decimalLatitude: 53.195; decimalLongitude: -117.914; **Event:** samplingProtocol: Intercept Trap; eventDate: 21-Jul-2012; habitat: Forest; fieldNotes: 1 intercept trap|overcast|16C|birch and spruce forest on a slope, lots of fallen logs; **Record Level:** institutionCode: CBG
- **Type status:**Paratype. **Occurrence:** catalogNumber: BIOUG06581-B12; recordedBy: BIOBus 2012; individualCount: 1; sex: female; lifeStage: adult; preparations: Pinned; **Location:** country: Canada; stateProvince: Saskatchewan; locality: Prince Albert National Park, Narrows Peninsula Trail; verbatimElevation: 530 m; decimalLatitude: 53.9872; decimalLongitude: -106.282; **Event:** samplingProtocol: Intercept Trap; eventDate: 14-Jul-2012; habitat: Forest; fieldNotes: 1 intercept trap|partly cloudy|24C|white spruce and poplar forest; **Record Level:** institutionCode: CBG
- **Type status:**Paratype. **Occurrence:** catalogNumber: BIOUG06898-F06; recordedBy: BIOBus 2012; individualCount: 1; sex: male; lifeStage: adult; preparations: Pinned; **Location:** country: Canada; stateProvince: Alberta; locality: Elk Island National Park, Elk Island Parkway; verbatimElevation: 729 m; decimalLatitude: 53.663; decimalLongitude: -112.823; **Event:** samplingProtocol: Intercept Trap; eventDate: 03-Jul-2012; habitat: Forest; fieldNotes: 1 intercept trap|overcast|14C on end date|black spruce stand; **Record Level:** institutionCode: CBG
- **Type status:**Paratype. **Occurrence:** catalogNumber: BIOUG09340-D09; recordedBy: BIOBus 2012; individualCount: 1; sex: male; lifeStage: adult; preparations: Pinned; **Location:** country: Canada; stateProvince: Alberta; locality: Jasper National Park, Pocahontas Campground; verbatimElevation: 1131 m; locationRemarks: Site C21; decimalLatitude: 53.195; decimalLongitude: -117.914; **Event:** samplingProtocol: Pan Trap; eventDate: 21-Jul-2012; habitat: Forest; fieldNotes: 10 pan traps|overcast|16C|birch and spruce forest on a slope, lots of fallen logs; **Record Level:** institutionCode: CBG
- **Type status:**Paratype. **Occurrence:** catalogNumber: BIOUG09340-B10; recordedBy: BIOBus 2012; individualCount: 1; sex: female; lifeStage: adult; preparations: Pinned; **Location:** country: Canada; stateProvince: Alberta; locality: Jasper National Park, Pocahontas Campground; verbatimElevation: 1131 m; locationRemarks: Site C21; decimalLatitude: 53.195; decimalLongitude: -117.914; **Event:** samplingProtocol: Pan Trap; eventDate: 21-Jul-2012; habitat: Forest; fieldNotes: 10 pan traps|overcast|16C|birch and spruce forest on a slope, lots of fallen logs; **Record Level:** institutionCode: CBG
- **Type status:**Paratype. **Occurrence:** catalogNumber: BIOUG13692-A05; recordedBy: BIObus 2013; individualCount: 1; sex: female; lifeStage: adult; preparations: Pinned; **Location:** country: Canada; stateProvince: New Brunswick; locality: Fundy National Park, Maple Grove Trail; verbatimElevation: 212 m; decimalLatitude: 45.59; decimalLongitude: -64.985; **Event:** samplingProtocol: Intercept Trap; eventDate: 30-May-2013; habitat: Forest; fieldNotes: 1 Intercept Trap|overcast|hardwood forest, maple stand; **Record Level:** institutionCode: CBG

#### Description

Male (n = 8): Body length 2.7-3.6 mm. Wing length 2.3-2.9 mm. **Colouration** (Dry specimen). Head brown to dark brown; face and clypeus dark brown; labellum yellow to pale brown; palpus yellow. Antenna with scape and pedicel yellow; flagellum pale brown to dark brown. Thorax with scutum and lateral sclerites brown; propleura brown to dark brown; halteres whitish-yellow to yellow. Legs whitish-yellow to yellow. Abdomen dark brown, sometimes with a pale area covering basolateral part of tergite III. Terminalia yellow. **Head**. Frons, vertex and clypeus covered with pale setae. Antenna (n = 6) 1.8-2.0 times as long as length from vertex to ventral margin of clypeus; flagellomeres slightly longer than broad, with sixth flagellomere (n = 6) 1.0-1.1 times as long as wide. **Thorax**. Scutum covered with pale setae. **Legs**. Fore leg with tibia (n = 5) 1.0-1.05 times as long as first tarsomere. Mid-tibia with (n = 5) 16-19 anterior, (n = 5) 3-4 posterodorsal, (n = 5) 5-7 posterior and (n = 5) 1-3 posteroventral bristles. Hind tibia with 7-9 anterodorsal, 4 posterodorsal and 3-6 posterior bristles. **Wings**. Vein r-m (n = 6) 2.1-2.4 times longer than stem of M-fork. **Abdomen.** Tergites covered with pale setae. **Terminalia** (Fig. 37a, b, c). Each part of divided tergite IX with about 7-8 setae, most apical seta stout. GL with length 0.51-53 of gonocoxite width; apico-internal margin slightly angled exteriorly; basal third covered with setae; apex with 4 setae. Aedaegal guides short with acute apex. Hypandrium covered with 9-12 setae, with apical pair reaching two-thirds to three-fourths of GL (Fig. 37a, b). Hypandrial lobe with each branch slender, evenly tapered. Gonostylus (Fig. 37c) with DB 1.9-2.1 times longer than broad, distinctly curved interiorly, apical part broad, apex rounded; evenly covered with setae on dorsal side, except on most apical part; external margin evenly rounded with row of 5 elongated setae. VB round, with 2 small setae. IB apically with 1 seta close to apex and pair of setae one-third from apex. MB apically acute, with 1 seta close to base.

Female (n =) : Body length 2.9-3.7 mm. (n = 3) Wing length 2.5-2.9 mm. **Colouration** (Dry specimen). Head, face and clypeus dark brown; labellum and palpus yellow. Antenna with scape and pedicel yellow; flagellum brown. Thorax with scutum and propleura brown; lateral sclerites dark brown; halteres whitish-yellow. Legs yellow. Abdomen brown, tergites III-VI with lateral pale areas. Terminalia yellow. **Head**. Frons, vertex and clypeus covered with pale setae. Antenna (n = 3) 1.75-1.9 times as long as length from vertex to ventral margin of clypeus; flagellomeres quadrate, with sixth flagellomere as long as broad. **Thorax**. Scutum covered with pale setae. **Legs**. Fore leg with tibia (n = 4) 1.0-1.05 times as long as first tarsomere. Mid-tibia with (n = 3) 16-19 anterior, (n = 1) 4 posterodorsal, (n = 1) 5 posterior and (n = 1) 2 posteroventral bristles. Hind tibia with (n = 3) 7-9 anterodorsal, (n = 3) 4-5 posterodorsal and (n = 3) 4-5 posterior bristles. **Wings**. Vein r-m (n = 3) 2.1-2.4 times longer than stem of M-fork. **Abdomen.** Tergites covered with pale setae. **Terminalia** (Fig. 37d, e, f). Cerci with apical segment 0.6 times as long as basal segment. Tergite VIII with apicolateral margin forming distinctly protruding angle (Fig. 37d). Sternite VII with apex very slightly protruding. Sternite VIII (Fig. 37e) with hypogynal valves separated by narrow cleft with depth about one-third of sternite VIII length; apical seta 0.55-0.6 of sternite VIII length. Gonapophysis IX (Fig. 37f) with basolateral part expanding in obtuse angle; spermathecal eminence trilobed, in ventral view appearing cross-shaped; apically with 4 small setae.

#### Diagnosis

Distinguished from *E.repandoides* in having the dorsal branch of the gonostylus with apex rounded (Fig. 37c), apicoventrally without a short angular projection, in combination with the hypandrium with 9-12 setae (Fig. 37a, b); from *E.brevilobata* and *E.breviflagellata* in having the antennae 1.8-2.0 times as long as length from vertex to ventral margin of clypeus, in combination with the dorsal gonostylus branch without a distinct apical lobe (Fig. 37c); from other species in the *E.parva* group in having the dorsal gonostylus branch short and rounded (Fig. 37c), in combination with apico-internal margin of the gonocoxal lobe slightly angled exteriorly (Fig. 37a, b).

#### Etymology

From Latin *sphaera*, sphere, relating to the shape of the dorsal branch of the gonostylus.

#### Distribution

Nearctic, Canada (Fig. 13)

#### Biology

Adults collected in different types of forest and wetland habitats.

### Exechia subrepanda

Lindemann sp. n.

986465A3-78A1-5A15-8609-071C1A2CD3C2

urn:lsid:zoobank.org:act:1307676e-7a4e-449a-a1f6-a2265c90d807

http://dx.doi.org/10.5883/BOLD:ACI6494

#### Materials

- **Type status:**Holotype. **Occurrence:** catalogNumber: BIOUG22795-B10; recordedBy: BIObus 2014; individualCount: 1; sex: male; lifeStage: adult; preparations: Pinned, with genitalia in glycerine in separate microvial; **Location:** country: Canada; stateProvince: British Columbia; locality: Darkwoods Conservation Area, Grassy Lake; verbatimElevation: 1750 m; decimalLatitude: 49.281; decimalLongitude: -117.043; **Event:** samplingProtocol: Pan trap; eventDate: 2014-08-25; habitat: Wetland; fieldNotes: 10 Pan Traps|Mostly overcast and rainy with wet vegetation|open marsh with willows, grass and reeds; **Record Level:** institutionCode: CBG
- **Type status:**Paratype. **Occurrence:** catalogNumber: BIOUG06394-C12; recordedBy: BIOBus 2012; individualCount: 1; sex: male; lifeStage: adult; preparations: Pinned, with genitalia in glycerine in separate microvial; **Location:** country: Canada; stateProvince: Alberta; locality: Waterton Lakes National Park, East of 2 Flags Lookout, Highway 6 pulloff; verbatimElevation: 1562 m; decimalLatitude: 49.065; decimalLongitude: -113.778; **Event:** samplingProtocol: Pitfall Trap; eventDate: 2012-08-11; habitat: Forest; fieldNotes: 10 Pitfall Traps|Overcast, 19C|montane forest, douglas fir and lodgepole pine stand with aspen and birch understory; **Record Level:** institutionCode: CBG
- **Type status:**Paratype. **Occurrence:** catalogNumber: BIOUG09205-C08; recordedBy: BIOBus 2012; individualCount: 1; sex: male; lifeStage: adult; preparations: Pinned, with genitalia in glycerine in separate microvial; **Location:** country: Canada; stateProvince: Alberta; locality: Jasper National Park, Pocahontas Campground; verbatimElevation: 1131 m; locationRemarks: Site C21; decimalLatitude: 53.195; decimalLongitude: -117.914; **Event:** samplingProtocol: Intercept Trap; eventDate: 2012-07-21; habitat: Forest; fieldNotes: 1 intercept trap|overcast|16C|birch and spruce forest on a slope, lots of fallen logs; **Record Level:** institutionCode: CBG
- **Type status:**Paratype. **Occurrence:** catalogNumber: BIOUG06132-H10; recordedBy: BIOBus 2012; individualCount: 1; sex: female; lifeStage: adult; preparations: Pinned, with genitalia in glycerine in separate microvial; **Location:** country: Canada; stateProvince: Alberta; locality: Banff National Park, Baker Creek picnic area, Adjacent to hydro line; verbatimElevation: 1500 m; decimalLatitude: 51.35; decimalLongitude: -116.063; **Event:** samplingProtocol: Intercept Trap; eventDate: 2012-07-28; habitat: Forest; fieldNotes: 1 Intercept Trap|mostly sunny with a few cloudy on end date|mature forest, lodgepole pine, temperate forest; **Record Level:** institutionCode: CBG
- **Type status:**Paratype. **Occurrence:** catalogNumber: BIOUG09204-G02; recordedBy: BIOBus 2012; individualCount: 1; sex: female; lifeStage: adult; preparations: Pinned, with genitalia in glycerine in separate microvial; **Location:** country: Canada; stateProvince: Alberta; locality: Jasper National Park, Pocahontas Campground; verbatimElevation: 1131 m; locationRemarks: Site C21; decimalLatitude: 53.195; decimalLongitude: -117.914; **Event:** samplingProtocol: Intercept Trap; eventDate: 2012-07-21; habitat: Forest; fieldNotes: 1 intercept trap|overcast|16C|birch and spruce forest on a slope, lots of fallen logs; **Record Level:** institutionCode: CBG

#### Description

Male (n = 3): Body length 3.0-3.2 mm. Wing length 2.3-2.6 mm. **Colouration** (Dry specimen). Head brown to dark brown; face and clypeus dark brown; labellum and palpus yellow. Antenna with scape and pedicel yellow; flagellum pale brown to brown, basal part of first flagellomere pale. Scutum and lateral sclerites brown; propleura pale brown; halteres whitish-yellow. Legs whitish-yellow. Abdomen brown. Terminalia yellow to pale brown. **Head**. Vertex, frons and clypeus covered with pale setae. Antenna 1.55-1.7 times as long as length from vertex to ventral margin of clypeus; flagellomeres quadrate or longer than broad, with sixth flagellomere 1.0-1.2 times as long as wide. **Thorax**. Scutum covered with pale setae. **Legs**. Fore leg with tibia (n = 1) as long as first tarsomere. Mid-tibia with (n = 2) 15-20 anterior, (n = 2) 3-4 posterodorsal, (n = 2) 6 posterior and (n = 1) 1 posteroventral bristles. Hind tibia with (n = 2) 5-7 anterodorsal, 4 posterodorsal and 3 posterior bristles. **Wings**. Vein r-m (n = 2) 2.2-2.7 times longer than stem of M-fork. **Abdomen.** Tergites covered with pale setae. **Terminalia** (Fig. 38a, b, c). Each part of divided tergite IX with 8-9 setae, most apical seta stout. GL (Fig. 38a, b) with length 0.53-0.60 of gonocoxite width; apico-internal margin slightly angled exteriorly; basal half or basal third covered with setae; apex with 3-4 setae. Aedaegal guides short with rounded or slightly angled apex (Fig. 38a, b). Hypandrium covered with 8-12 setae with apical pair reaching about three-fourths of GL (Fig. 38a, b). Hypandrial lobe with each branch slender, evenly tapered, apex acute. Gonostylus (Fig. 38c) with DB 2.4-2.7 times longer than broad, curved interiorly, apical lobe well defined, apex acute or somewhat rounded; evenly covered with setae on dorsal side, except on most apical part; external margin forming distinct angle, with row of 4-5 elongate setae. VB with 2 small setae. IB apically with 1 seta close to apex and pair of setae one-third from apex. MB apex acute, with 1 seta close to the base.

Female (n = 2): Body length 2.6-3.2 mm. Wing length 2.2-2.6 mm. **Colouration** (Dry specimen). Head, face and clypeus dark brown; labellum and palpus yellow. Antenna with scape and pedicel yellow; flagellum pale brown, basal part of first segment pale. Scutum and lateral sclerites brown; propleura pale brown; halteres whitish-yellow. Legs whitish-yellow. Abdomen pale brown to brown, tergites III-VI with yellow lateral areas extending dorsally at anterior tenth to eighth of tergites III and IV, forming two more or less distinct pale bands. Terminalia yellow. **Head**. Vertex, frons and clypeus covered with pale setae. Antenna 1.65-1.67 times as long as length from vertex to ventral margin of clypeus; flagellomeres slightly longer than broad, with sixth flagellomere 1.1-1.2 times as long as wide. **Thorax**. Scutum covered with pale setae. **Legs**. Fore leg with tibia (n = 1) as long as first tarsomere. Mid-tibia with (n = 1) 16 anterior, (n = 1) 3 posterodorsal and 4-5 posterior bristles. Hind tibia with (n = 1) 7 anterodorsal, (n = 1) 5 posterodorsal and (n = 1) 4 posterior bristles. **Wings**. Vein r-m 2-2.7 times longer than stem of M-fork. **Abdomen.** Tergites covered with pale brown setae. **Terminalia** (Fig. 38d, e, f). Cerci with apical segment half as long as basal segment. Tergite VIII with apicolateral margin slightly angular, not distinctly protruding (Fig. 38d). Sternite VII with apicoventral margin evenly rounded, sometimes with medial part very slightly protruding. Sternite VIII (Fig. 38e) with hypogynal valves separated by narrow cleft with depth about one fourth of sternite VIII length; apical seta 0.55-0.6 of sternite VIII length. Gonapophysis IX (Fig. 38f) with basolateral part expanding in relatively obtuse angle; spermathecal eminence trilobed, in ventral view appearing cross-shaped.

#### Diagnosis

Distinguished from *E.repanda* in having the dorsal gonostylus branch 2.4-2.7 times longer than broad and with external row of setae covering only one-fifth to one-fourth of the total dorsal gonostylus branch length (Fig. 38c); from *E.neorepanda* in having the dorsal gonostylus branch more curved, with external margin forming a distinct angle and with external row of setae covering only one-fifth to one-fourth of total dorsal gonostylus branch length (Fig. 38c); from *E.curvata* in having the dorsal gonostylus branch with external margin forming a less protruding angle (Fig. 38c), in combination with the gonocoxal lobe more elongate, its length 0.53-0.60 of the gonocoxite width (Fig. 38a, b); from other species in the *E.parva* group in having the dorsal gonostylus branch 2.4-2.7 times longer than broad (Fig. 38c), in combination with the gonocoxal lobes covered with setae on basal half or less and with apico-internal margin slightly angled exteriorly (Fig. 38a, b).

#### Etymology

Named after the species *Exechiarepanda*, with Latin prefix *sub*-, below, relating to the close resemblance to *E.repanda*.

#### Distribution

Nearctic, Canada (Fig. 13).

#### Biology

Unknown

#### Taxon discussion

The species is very close to *E.neorepanda* and *E.repanda* and species determination should be conducted with care or with aid of DNA barcoding.

### Exechia toyoheii

Lindemann sp. n.

BF42BCDF-9F28-5159-A97A-87CDD980DC46

urn:lsid:zoobank.org:act:7fd1ffd0-0ca9-420b-9036-fb9a60504d6c

http://dx.doi.org/10.5883/BOLD:AEE4580

#### Materials

- **Type status:**Holotype. **Occurrence:** catalogNumber: TSZD-JKJ-109250; recordedBy: J. Kjærandsen; individualCount: 1; sex: male; lifeStage: adult; preparations: Pinned, with genitalia in glycerine in separate microvial; **Location:** island: Hokkaido; country: Japan; stateProvince: Hokkaido Prefecture; locality: Kamikawa-cho, Hakuun-bashi, Ishikari River, Shirakawa; verbatimElevation: 398 m; locationRemarks: Site 4; decimalLatitude: 43.81722; decimalLongitude: 142.84556; **Event:** samplingProtocol: sweep net; eventDate: 2006-10-06; **Record Level:** institutionCode: TMU
- **Type status:**Paratype. **Occurrence:** catalogNumber: TSZD-JKJ-233423; recordedBy: J. Kjærandsen; individualCount: 1; sex: male; lifeStage: adult; preparations: Pinned, with genitalia in glycerine in separate microvial; **Location:** island: Hokkaido; country: Japan; stateProvince: Hokkaido Prefecture; locality: Kushiro-shi, Middle reach of Ibeshibetsu River near Lake Akan, Akan-cho; verbatimElevation: 448 m; locationRemarks: Site 3; decimalLatitude: 43.48806; decimalLongitude: 144.14778; **Event:** samplingProtocol: sweep net; eventDate: 2006-10-04; **Record Level:** institutionCode: TMU
- **Type status:**Paratype. **Occurrence:** catalogNumber: TSZD-JKJ-111551; recordedBy: O. Yata; individualCount: 1; sex: male; lifeStage: adult; preparations: Pinned, with genitalia in glycerine in separate microvial; **Location:** island: Kyushu; country: Japan; stateProvince: Fukuoka Prefecture; locality: Nogochi; **Event:** eventDate: 1975-05-13; **Record Level:** institutionCode: KUEC
- **Type status:**Paratype. **Occurrence:** catalogNumber: TSZD-JKJ-111549; recordedBy: T. Saigusa; individualCount: 1; sex: male; lifeStage: adult; preparations: Pinned, with genitalia in glycerine in separate microvial; **Location:** island: Kyushu; country: Japan; stateProvince: Miyazaki Prefecture; locality: Kobayashi, Inokodanibashi 2 , 400m upstream; verbatimElevation: 395-370 m; **Event:** eventDate: 2004-11-26; **Record Level:** institutionCode: KUEC
- **Type status:**Paratype. **Occurrence:** catalogNumber: TSZD-JKJ-111550; recordedBy: T. Saigusa; individualCount: 1; sex: male; lifeStage: adult; preparations: Pinned, with genitalia in glycerine in separate microvial; **Location:** island: Kyushu; country: Japan; stateProvince: Miyazaki prefecture; locality: Kobayashi , Suki-son, Inokodanibashi 3; verbatimElevation: 385 m; **Event:** eventDate: 2004-12-15; **Record Level:** institutionCode: KUEC
- **Type status:**Paratype. **Occurrence:** catalogNumber: TSZD-JKJ-232386; recordedBy: J. Kjærandsen; individualCount: 1; sex: male; lifeStage: adult; preparations: Pinned, with genitalia in glycerine in separate microvial; **Location:** island: Hokkaido; country: Japan; stateProvince: Hokkaido Prefecture; locality: Chitose-shi, Kokeno-domon Gallary beside Lake Shikotsu; verbatimElevation: 279 m; decimalLatitude: 42.71194; decimalLongitude: 141.32111; **Event:** samplingProtocol: sweep net; eventDate: 2006-10-02; **Record Level:** institutionCode: TMU
- **Type status:**Paratype. **Occurrence:** catalogNumber: TSZD-JKJ-109247; recordedBy: J. Kjærandsen; individualCount: 1; sex: male; lifeStage: adult; preparations: Pinned, with genitalia in glycerine in separate microvial; **Location:** island: Hokkaido; country: Japan; stateProvince: Hokkaido Prefecture; locality: Kushiro-shi, Middle-lower reach of Ibeshibetsu River near Lake Akan, Akan-cho; verbatimElevation: 427 m; locationRemarks: Site 2; decimalLatitude: 43.48083; decimalLongitude: 144.13917; **Event:** samplingProtocol: sweep net; eventDate: 2006-10-04; **Record Level:** institutionCode: TMU
- **Type status:**Paratype. **Occurrence:** catalogNumber: TSZD-JKJ-109248; recordedBy: J. Kjærandsen; individualCount: 1; sex: male; lifeStage: adult; preparations: Pinned, with genitalia in glycerine in separate microvial; **Location:** island: Hokkaido; country: Japan; stateProvince: Hokkaido Prefecture; locality: Kamikawa-cho, Hakuun-bashi, Ishikari River, Shirakawa; verbatimElevation: 398 m; locationRemarks: Site 4; decimalLatitude: 43.81722; decimalLongitude: 142.84556; **Event:** samplingProtocol: sweep net; eventDate: 2006-10-06; **Record Level:** institutionCode: TMU
- **Type status:**Paratype. **Occurrence:** catalogNumber: TSZD-JKJ-109249; recordedBy: J. Kjærandsen; individualCount: 1; sex: male; lifeStage: adult; preparations: Pinned, with genitalia in glycerine in separate microvial; **Location:** island: Hokkaido; country: Japan; stateProvince: Hokkaido Prefecture; locality: Kamikawa-cho, Hakuun-bashi, Ishikari River, Shirakawa; verbatimElevation: 398 m; locationRemarks: Site 4; decimalLatitude: 43.81722; decimalLongitude: 142.84556; **Event:** samplingProtocol: sweep net; eventDate: 2006-10-06; **Record Level:** institutionCode: TMU

#### Description

Male (n = 9): Body length (n = 8) 3.5-3.9 mm. Wing length 2.9-3.4 mm. **Colouration** (Dry specimen). Head dark brown; face and clypeus dark brown to pale brown; labellum yellow, sometimes pale brown; palpus yellow to pale brown, segments 4 and 5 usually somewhat darker. Antenna with scape and pedicel yellow; flagellum dark brown to pale brown, basal part of first flagellomere yellow. Thorax with scutum dark brown to brown, anterolateral margin paler; lateral sclerites pale brown to dark brown; propleura pale brown to brown; halteres whitish-yellow. Legs whitish-yellow to yellow, coxa sometimes slightly darker. Abdomen dark brown, tergites II-III with a yellow laterodorsal area. Terminalia brown with MB and internal lobe of DB dark brown (Fig. 39b). **Head**. Frons and vertex covered with short, dark brown to pale brown setae. Clypeus covered with pale setae. Antenna (n = 3) 1.9-2.1 times length from vertex to ventral margin of clypeus; flagellomeres quadrate or longer than broad; sixth flagellomere (n = 6) 1.0-1.2 times as long as wide. **Thorax**. Scutum covered with short, dark brown to pale brown setae. **Legs**. Fore leg with tibia (n = 8) 0.77-0.85 times length of first tarsomere. Mid-tibia with (n = 8) 22-28 anterior, (n = 8) 4-6 posterodorsal, (n = 6) 12-15 posterior and (n = 6) 4-7 posteroventral bristles. Hind tibia with 10-14 anterodorsal, 5-7 posterodorsal and 5-10 posterior bristles. **Wings**. Vein r-m (n = 3) 2.4-3.3 times longer than stem of M-fork. **Abdomen.** Tergites covered with long, pale brown to brown setae. **Terminalia** (Figs 2a, 39). Each part of divided tergite IX apically with about 4-6 setae, 1-2 most apical setae stout. Apicoventral margin of each gonocoxite forming short protrusion between GL and hypandrium, each with stout apical seta. GL (Fig. 39a, b) relatively narrow, length about one-third of gonocoxite width, apex with 3 elongate setae, otherwise bare. Aedaegal guides elongate and acute. Hypandrium covered with 11-13 setae, with apical pair very short, hardly reaching beyond apical margin of gonocoxites (Fig. 39a, b). Hypandrial lobe with each branch elongate, narrow, apically abruptly curved interiorly, apex rounded. Gonostylus (Fig. 39c) with DB short and rounded, forming large and darkened internal lobe; evenly covered with setae on dorsal side, except on the internal lobe; apically with 4-6 elongate setae. VB large, apically acute; with 1 elongate seta on the apex and 3 smaller setae further down, one distinctly wider than others. IB apically with 1 seta on apex and row of 4 setae on small elevation one-third from apex. MB with apex truncate; internal margin forming elongate acute process extending interiorly; apex with row of 3-4 elongated setae sometimes with slightly widened apex, all longer than MB length.

Female: Unknown.

#### Diagnosis

Distinguished from *E.rohdendorfi* in having the hypandrium with a short apical pair of setae (Fig. 39a, b), in combination with the apicoventral gonocoxal margin with a pair of protrusions, each with a stout apical setae (Fig. 39a, b), the internal branch of the gonostylus with subapical row of setae placed at a greater distance from the apical setae (Fig. 39c) and by the shape of the dorsal and medial branch of the gonostylus (Fig. 39c); from *E.crassiseta* and *E.trunciseta* in having a short dorsal gonostylus branch with a large darkened internal lobe (Fig. 39c); from other species in the *E.parva* group in having a darkened medial gonostylus branch with 3-4 apical setae, all of which are as long as or longer than the medial gonostylus branch length (Fig. 39c).

#### Etymology

Named in honour of Professor Emeritus Toyohei Saigusa, the collector of two of the paratypes, who also provided us with the loan of other invaluable material of several species described in this revision.

#### Distribution

East Palaearctic, Japan (Fig. 10)

#### Biology

Unknown

### Exechia trunciseta

Lindemann sp. n.

9306DCA4-2C06-5654-98A1-FDA1DA07D64E

urn:lsid:zoobank.org:act:ad92ce22-8806-45de-8bd7-94fc1ccc1a5a

#### Materials

- **Type status:**Holotype. **Occurrence:** catalogNumber: TSZD-JKJ-111562; recordedBy: T. Saigusa; individualCount: 1; sex: male; lifeStage: adult; preparations: Pinned, with genitalia in glycerine in separate microvial; **Location:** country: Bhutan; stateProvince: Thimpu District; locality: East of Dochhu La; verbatimElevation: 2700 m; decimalLatitude: 27.487778; decimalLongitude: 89.762222; **Event:** eventDate: 1993-08-16; **Record Level:** collectionCode: KUEC

#### Description

Male: Wing length 3.3 mm. **Colouration** (Dry specimen). Head, face and clypeus dark brown; labellum yellow; palpus yellow with segments 4 and 5 pale brown. Antenna with scape and pedicel yellow; flagellum dark brown, basal half of first flagellomere yellow. Scutum dark brown with yellow anterolateral margin; lateral sclerites and propleura brown; halteres whitish-yellow. Legs yellow. Abdomen dark brown, tergites II-III with a yellow laterodorsal area. Terminalia brown with MB dark brown (Fig. 40b). **Head**. Frons and vertex covered with short, brown setae. Clypeus covered with pale setae. **Thorax**. Scutum covered with short, brown setae. **Legs**. Fore leg with tibia 0.72 times length of first tarsomere. Mid-tibia with 23 anterior, 5 posterodorsal, 11 posterior and 3 posteroventral bristles. Hind tibia with 10 anterodorsal, 5 posterodorsal and 5 posterior bristles. **Abdomen.** Tergites covered with brown setae. **Terminalia** (Fig. 40). Each part of divided tergite IX with about 4 setae, most apical seta stout. Apicoventral margin of each gonocoxite forming short protrusion between the GL and hypandrium, each with 2 very stout setae, most apical seta distinctly truncated. GL (Fig. 40a, b) about one-third of gonocoxite width, apex with 2-3 relatively stout setae. Aedaegal guides elongate and apically acute, converging (Fig. 40a). Hypandrium covered with about 6 setae, with apical pair very short, length about half GL length (Fig. 40a, b). Hypandrial lobe with each branch elongate, slender, evenly tapered, apex acute. Gonostylus (Fig. 40c) with DB elongate and apically rounded; baso-internally forming small lobe, extending interiorly; evenly covered with relatively stout seta on dorsal side, except on most basal part and on internal lobe; apically with about 4 very stout and apically truncated setae. VB apically acute with 1 elongated seta on apex and 4 smaller setae further down, one distinctly wider than the others. IB apically with 1 seta on apex and row of 4 setae on elevation one-sixth from apex. MB wide, elongate, apex acute, baso-internally forming short distinctly right-angled process, apico-internally with row of 4 elongated setae, all about as long as MB length.

Female: Unknown

#### Diagnosis

Distinguished from *E.crassiseta* by the shape of the medial gonostylus branch (Fig. 40c); from other species in the *E.parva* group in having the dorsal gonostylus branch basolaterally forming a short lobe and apically with a row of 4 stout truncated setae (Fig. 40c), in combination with a dark medial gonostylus branch (Fig. 40b), apically with a row of 4 elongated setae, all about as long as the medial gonostylus branch length (Fig. 40c).

#### Etymology

From Latin *truncatus*, truncated and *seta*, bristle, relating to the shape of the seta on posteroventral margin of the gonocoxites and on apical margin of the dorsal lobe of the gonostylus.

#### Distribution

Oriental, Nepal (2700 m a.s.l., Fig. 19)

#### Biology

Unknown.

### Exechia zuluensis

Lindemann sp. n.

567006E4-7C8E-53DB-AB72-469887EB0A4E

urn:lsid:zoobank.org:act:2ff411dd-0893-4cd7-914e-935b77b47676

http://dx.doi.org/10.5883/BOLD:AEA3872

#### Materials

- **Type status:**Holotype. **Occurrence:** catalogNumber: TSZD-JKJ-107186; recordedBy: M. Mostovski; individualCount: 1; sex: male; lifeStage: adult; preparations: Pinned, with genitalia in glycerine in separate microvial; **Location:** country: South Africa; stateProvince: KwaZulu-Natal; municipality: Pietermaritzburg; locality: Karkloof Nat. Res.; verbatimElevation: 1325 m; decimalLatitude: -29.3169; decimalLongitude: 30.2514; **Event:** samplingProtocol: Malaise trap; eventDate: 2005-08-27; habitat: mistbelt forest; **Record Level:** institutionCode: TMU

#### Description

Male: Body length 4.1 mm. Wing length 3.4 mm. **Colouration** (Dry specimen). Head, face and clypeus dark brown; labellum and palpus yellow. Antenna with scape and pedicel yellow; flagellum brown, first segment with yellow base. Thorax with scutum dark brown, lateral margin broadly yellow; lateral sclerites brown; propleura pale brown; halteres whitish-yellow. Legs whitish-yellow. Abdomen dark brown, tergites II-III with lateroventral yellow area. Terminalia pale brown. **Head**. Frons and vertex covered with pale setae. Clypeus covered with pale brown setae. Antenna long, 2.3 times as long as length from vertex to ventral margin of clypeus; flagellomeres slightly longer than broad, with sixth flagellomere 1.1 times as long as wide. **Thorax**. Scutum covered with short pale brown setae. **Legs**. Fore leg with tibia 0.89 times as long as first tarsomere. Mid-tibia with 26 anterior, 4 posterodorsal, 10 posterior and 3 posteroventral bristles. Hind tibia with 12 anterodorsal, 6 posterodorsal and 5 posterior bristles. **Wings**. Vein r-m 2.8 times longer than stem of M-fork. **Abdomen.** Tergites covered with pale brown setae. **Terminalia** (Fig. 41). Each part of divided tergite IX apically with about 7 setae, apical seta stout. Gonocoxites with setae on apicoventral margin elongate, reaching as far as, or slightly shorter than level of GL apex (Fig. 41a). Each GL apex with row of 4 elongate setae, somewhat splayed out (Fig. 41a). Aedaegal guides indistinct or reduced. Hypandrium covered with about 12 setae, with apical pair elongate, almost reaching level of GL apex (Fig. 41a, b). Hypandrial lobe with each branch evenly tapered, apically narrow, apex rounded (Fig. 41a). Gonostylus (Fig. 41c) with DB elongate and acuminate, apex spathulate; about 3.3 times longer than broad; evenly covered with setae on dorsal side, except on apical sixth. VB small and round; basally with short seta, otherwise bare. IB apically acute, with 1 seta close to apex and pair of setae about one-third from apex. MB slightly curved interiorly with apex acute; small seta located one-sixth from apex.

Female: Unknown.

#### Diagnosis

Distinguished from *E.arcuata* in having the gonocoxal lobe straight (Fig. 41a, b); from other species in the *E.parva* group in having the dorsal gonostylus branch 3.3 times longer than broad with apex spathulate (Fig. 41c), in combination with the internal gonostylus branch with 1 seta close to the apex (Fig. 41c), the gonocoxal lobes with apico-internal margin straight (Fig. 41a) and the apical hypandrial setae reaching almost the level of the gonocoxal lobe apex (Fig. 41a).

#### Etymology

From *KwaZulu-Natal*, the Province where the holotype was collected, with Latin suffix -*ensis*, belonging to.

#### Distribution

Afrotropical, South Africa (Fig. 4)

#### Biology

Collected in mistbelt forest (1325 m a.s.l.).

## Identification Keys

### Males

**Table:** 

1	Wings with dark spots (Fig. 9e). Each cercus triangular, apically wide, tapering towards basal part (Fig. 9d). GL very short, length about one-sixth of the gonocoxite width (Fig. 9a, b). Aedaegal guides long and arched, apically rounded (Fig. 9a). Oriental.	* E. bifasciata *
–	Wings without spots. Each cercus kidney-shaped (Fig. 2b). GL longer, length at least one-third of the gonocoxite width. Aedaegal guides either shorter, less arched or apically pointed.	2
2	In ventral view, GLs entirely setose and not evenly tapered (Figs 11a, 21a).	3
–	In ventral view, GLs with apical part more or less bare (Fig. 2), or entirely setose and evenly tapered (Figs 5a, 6a, 28a).	5
3	Hypandrium extended distally, forming a large medial lobe that reaches far beyond apicoventral gonocoxal margin, apically with two stout setae (Fig. 21a). Aedaegal guides spathulate. Oriental.	4
–	Hypandrium not forming a lobe, with four stout setae (Fig. 11a). Gonostylus with DB, MB and IB branched (Fig. 11c). Aedaegal guides acute. Afrotropical (Madagascar).	* E. brachiata *
4	GL reaching beyond apical hypandrial setae (Fig. 21a). Internal margin of MB smooth (Fig. 21c). Oriental.	* E. columna *
–	GL shorter, not reaching beyond apical hypandrial setae (Fig. 35a). Internal margin of MB forming a few teeth (Fig. 35c). Oriental.	* E. serrae *
5	MB with a short thumb-like process with two small setae placed on the apex (Fig. 18c). DB apically bifurcate (Fig. 18c). Oriental.	* E. chirotheca *
–	MB not as described above. DB at most apically emarginate, not bifurcate.	6
6	MB relatively dark (Figs 33b, 40b), apically with at least 3 elongated setae, as long as or longer than the length of the MB (Figs 22c, 33c, 39c, 40c). VB large, the apical seta distinctly longer than the VB length (Fig. 39c). Aedaegal guides long, reaching beyond basal half of GL, apically acute (Fig. 2a).	7
–	MB not dark (Fig. 2b), apically with at most 3 short setae, shorter than half the length of the MB (Figs 8c, 28c). VB usually smaller, the apical setae usually not much longer than the VB length. Aedaegal guides shorter, not reaching longer than basal half of GL, apically acute or rounded or reduced (Figs 17a, 25a, 27a, 32b).	10
7	DB with a large and usually darkened internal lobe extending in interior direction (Fig. 33a, c). All apicoventral gonocoxal setae apically acute (Fig. 39b). Fore leg with tibia 0.8-0.9 times as long as basitarsus. East Palaearctic.	8
–	DB with a much shorter, not darkened lobe, located closer to the base (Fig. 40a, c). At least one pair of apicoventral gonocoxal setae apically truncate (Figs 22b, 40b). Fore leg with tibia only about 0.7 times as long as basitarsus. Oriental.	9
8	DB apically with several stout setae (Fig. 39c). IB with subapical row of setae placed with some distance to the apical setae (Fig. 39c). MB as in Fig. 39c. Apicoventral gonocoxal margin distinctly protruding on each side of hypandrium (Fig. 39a). Apical hypandrial setae short, not reaching further than the middle of the GLs (Fig. 39a). East Palaearctic.	* E. toyoheii *
–	DB apically with at most one or two stout setae (Fig. 33c). IB with subapical row of setae placed almost at the same level as the apical setae, creating a broom-like appearance (Fig. 33c). MB as in Fig. 33c. Apicoventral gonocoxal margin, not distinctly protruding (Fig. 33a). Apical hypandrial setae longer, reaching about the level of the tip of the GL (Fig. 33a). East Palaearctic.	* E. rohdendorfi *
9	MB as in Fig. 22c. Oriental.	* E. crassiseta *
–	MB as in Fig. 40c. Oriental.	* E. trunciseta *
10	IB with 3 or 4 apical setae gathered together on or close to the apex (Figs 6c, 8c, 16c). Afrotropical.	11
–	IB with one seta on or close to the apex and a pair or row of 2-3 setae placed some distance from the apical seta (Figs 27c, 28c, 31c). In *E.acuata*, apical seta and subapical pair of setae appear as a single row of 3 setae (Fig. 7c).	16
11	GL entirely covered with setae, with entire length tapering (Fig. 6a). Hypandrial lobe as in Fig. 6a. Clypeus covered with black short setae. Afrotropical.	* E. afroparva *
–	GL with at least some of the length bare and not tapering (Figs 8a, 16a). Hypandrial lobe different.	12
12	GL with two apical setae (Figs 29a, 34a). DB with apical margin about half of its full length or longer (Figs 29c, 34c).	13
–	GL with 3-4 apical setae (Fig. 8a). DB with apical margin at most one third of its full length (Figs 8c, 16c).	14
13	DB about 2 times longer than broad; apico-external corner distinctly protruding, forming a rounded lobe (Fig. 34c). Aedaegal guides apically narrow and acute (Fig. 34a). Afrotropical.	* E. sambai *
–	DB about 2.8 times longer than broad; apico-external corner right-angled or virtually so, not distinctly protruding (Fig. 29c). Aedaegal guides apically wide and rounded (Fig. 29a). Afrotropical.	* E. penicillata *
14	Apical hypandrial setae about as long as DB (Figs 8a, 16a). GL with three apical setae. Aedaegal guides present (Figs 8a, 16a).	15
–	DB almost twice as long as the apical hypandrial setae or longer (Figs 7a, 41a), distinctly spathulate (Figs 7c, 41c). GL with four apical setae. Aedaegal guides indistinct or reduced (Figs 7a, 41a).	21
15	GLs with apical setae splaying (Fig. 16a). DB long, apical margin convex (Fig. 16c). Afrotropical.	* E. burundiensis *
–	GLs with apical setae parallel (Fig. 8a). DB short, square-shaped, apical margin emarginate (Fig. 8c). Afrotropical.	* E. ashleyi *
16	GL entirely covered with setae, with entire length tapering (Figs 5a, 28a).	17
–	GL with at least some of the length bare and not tapering (Fig. 17a).	19
17	GL very short (Fig. 20a). DB elongated, acute (Fig. 20b). West Palaearctic (Madeira).	* E. cinctiformis *
–	GL longer (Figs 5a, 28a). DB very short or with apical margin emarginate (Figs 5b, 28c).	18
18	DB round with a short apical lobe (Fig. 28c). Palaearctic.	* E. parva *
–	DB elongate, apical margin emarginate (Fig. 5b). Afrotropical.	* E. adenaparva *
19	Apical hypandrial setae reaching about as far as or beyond GLs (Figs 7a, 27a). Each GL with at least 4 apical setae, sometimes extended down apico-exterior margin; apico-internal margin without any distinct angle (Figs 27a, 41a).	20
–	Apical hypandrial setae not reaching beyond three-fourths of the GLs (Figs 17a, 25a). Each GL with 3-4 apical setae, usually not extended down apico-external margin; apico-internal margin more or less distinctly angled exteriorly (Figs 17a, 25a). Nearctic and Palaearctic species.	22
20	DB acute, 2.2-2.5 times longer than broad (Fig. 27c). Each GL with 4-5 apical setae (Fig. 27a). Aedaegal guides present. Oriental.	* E. pararepanda *
–	DB spathulate, about 3.3 times longer than broad or longer (Fig. 7c). Each GL with 4 apical setae. Aedaegal guides indistinct or reduced. Afrotropical.	21
21	GL short and slender, distinctly curved interiorly, apical setae very short (Fig. 7a). DB about 4 times longer than broad (Fig. 7c). Afrotropical.	* E. arcuata *
–	GL stouter, more or less straight (Fig. 41a). DB about 3.3 times longer than broad (Fig. 7c). Afrotropical.	* E. zuluensis *
22	DB with a distinctly protruding external margin, forming a short process, bearing row of setae (Fig. 17c). GL with at least basal half covered with setae, non-tapered part short (Fig. 17a). Aedaegal guides rounded (Fig. 17a). Nearctic.	* E. capillata *
–	DB with external margin less protruding (Figs 23c, 28c). GL with basal part less setose, non-tapered part longer (Fig. 25a). Aedaegal guides apically pointed (Fig. 25a).	23
23	DB short, at most 2.1 times longer than broad, if apical lobe distinct, then at most 0.2 of total DB length (Figs 14c, 37c).	24
–	DB more elongate, at least 2.2 times longer than broad, always with a well defined apical lobe with length at least 0.22 of total DB length (Figs 23c, 26c).	27
24	DB strongly curved interiorly, without a distinct apical lobe (Figs 32c, 37c). Antennae 1.8-2.2 times as long as length from vertex to ventral margin of clypeus.	25
–	DB only slightly curved interiorly, with a well defined apical lobe (Figs 12c, 14c). Antennae 1.4-1.65 times as long as length from vertex to ventral margin of clypeus.	26
25	DB apically acute (Fig. 32c), apico-internally forming a short angular projection (Fig. 32d). Hypandrium with 17-21 setae (Fig. 32a, b). West Palaearctic.	* E. repandoides *
–	DB apically rounded, without apicoventral projection (Fig. 37c). Hypandrium with 9-12 setae (Fig. 37a). Nearctic.	* E. sphaerata *
26	DB short and round, 1.3-1.5 times longer than broad, apical lobe broader than long (Fig. 12c). Antennae 1.4-1.5 times as long as length from vertex to ventral margin of clypeus. Nearctic.	* E. breviflagellata *
–	DB more elongate, 1.8-2.0 times longer than broad, apical lobe longer than broad (Fig. 14c). Antennae slightly longer, 1.5-1.65 times as long as length from vertex to ventral margin of clypeus. West Palaearctic.	* E. brevilobata *
27	DB at least 3 times longer than broad (Figs 25c, 36c). West Palaearctic.	28
–	DB shorter (Figs 26c, 30c).	29
28	DB spathulate, about 3.5 times longer than broad (Fig. 36c). Hypandrium with 10-12 setae (Fig. 36a). Hypandrial lobe with each branch evenly wide or with apical half only slightly widened (Fig. 36a). Aedaegal guides as in Figs 26a, 32a. GL very long, 0.6-0.75 times as long as gonocoxite width. West Palaearctic.	* E. spatulata *
–	DB not spathulate, apex acute or somewhat rounded, about 3.1 times longer than broad (Fig. 25c). Hypandrium with 25-31 setae (Fig. 25a, b). Each part of hypandrial lobe with apical half expanded to a wide disc (Fig. 25a, b). Aedaegal guides as in Fig. 25a. GL only 0.4-0.52 times as long as gonocoxite width. West Palaearctic.	* E. longilobata *
29	GL almost without a tapered basal part, virtually bare (Fig. 30a, b). DB about 2.2 times longer than broad (Fig. 30c). Mid-tibia with about 26 posterior bristles. East Palaearctic.	* E. rectiloba *
–	GL with a distinctly tapered basal part, at least basal fourth covered with setae (as in Fig. 25a). DB more than 2.2 times as long as broad (Fig. 26c). Mid-tibia with 22 or fewer posterior bristles.	30
30	DB with external margin evenly rounded, without a distinct angle; external row of setae covering one-third to half of total DB length (Fig. 26c). Darker specimens, head not conspicuously darker than rest of body. Wing length 2.7-3.0 mm. Each part of divided tergite IX with 3-4 setae. West Palaearctic.	* E. neorepanda *
–	DB external margin with a distinct angle (Fig. 23c). Paler specimens with head usually darker than rest of body. Wing length 2.3-2.8 mm. Each part of divided tergite IX with 3-9 setae. Nearctic.	31
31	DB about 2.23 times longer than broad; external margin with somewhat protruding angle; external row of setae only about one sixth of total DB length (Fig. 23c). GL length 0.42-0.48 of gonocoxite width (Fig. 23a, b). Wing length about 2.8 mm. Each part of divided tergite IX with 7-8 setae. Nearctic.	* E. curvata *
–	DB at least 2.3 times longer than broad, external margin of DB with angle not protruding (Figs 31c, 38c). Wing length 2.3-2.6 mm. Each part of divided tergite IX with 3-9 setae. Nearctic.	32
32	DB 2.3-2.4 times longer than broad; external row of setae covering one-third to half of total DB length (Fig. 31c). Antennae 1.83-1.93 times as long as length from vertex to ventral margin of clypeus. Each part of divided tergite IX with about 7-9 setae. Mid-tibia with 20-22 posterior bristles. Nearctic.	* E. repanda *
–	DB 2.4-2.6 times longer than broad; external row of setae covering one-fifth to one-fourth of total DB length (Fig. 38c). Antennae 1.55-1.7 times as long as length from vertex to ventral margin of clypeus. Each part of divided tergite IX with about 3-4 setae. Mid-tibia with 15-20 posterior bristles. Nearctic.	* E. subrepanda *

Note that couplet 21 can be reached from both couplet 14 and 20. *E.longichaeta* has not been included in the key due our lack of material of the species, as well as our inability to distinguish this species from *E.pararepanda*, based on published illustrations.

### Females

**Table:** 

1	Sternite VIII with two large bare areas expanding into basal part of setae cover, lateral margins straight and parallel, cleft shallow and somewhat v-shaped (Fig. 34d). Gonapophysis IX with basolateral part forming a distally-projecting lobe (Fig. 34e). Spermathecal eminence in ventral view appearing somewhat trifurcate (Fig. 34e). Afrotropical.	* E. sambai *
–	Sternite VIII without bare area extending into seta cover, lateral margins rounded (e.g. Fig. 29d). Gonapophysis IX with basolateral part not forming distally-protruding lobe.	2
2	Gonapophysis IX with basolateral part expanding almost in a right angle (Fig. 29e). Spermathecal eminence in ventral view distinctly trifurcate (Fig. 29e). Sternite VIII with cleft shallow and somewhat v-shaped (Fig. 29d). Afrotropical.	* E. penicillata *
–	Gonapophysis IX with basolateral part expanding in a relatively obtuse angle (e.g. Fig. 17f).	3
3	Spermathecal eminence in ventral view trifurcate with middle branch large and round and lateral branches short (Fig. 28e). Apical lobe of sternite VIII almost as long as width of hypogynial valve in ventral view (Fig. 28d). Sternite VII with apex slightly truncate. If pale areas on abdomen distinct, then only as lateral patches extended to about half tergal height. Palaearctic.	* E. parva *
–	Spermathecal eminence in ventral view cross-shaped (Fig. 38f) or unbranched (Fig. 17f). Apical lobes of sternite VIII usually shorter. Sternite VII with apex acute or evenly rounded. If pale areas on abdomen distinct, then forming complete or dorsally broken band along anterior margin of tergite III and sometimes also tergite IV. Nearctic.	4
4	Tergite VIII with apicolateral margin forming a distinctly-protruding angle (Fig. 23d). Spermathecal eminence in ventral view cross-shaped (Fig. 37f).	5
–	Tergite VIII with apicolateral margin forming an obtuse angle or virtually straight (Fig. 12d). Spermathecal eminence in ventral view cross-shaped (Fig. 38f) or unbranched (Fig. 17f).	6
5	Sternite VIII with cleft shallow, depth about 0.25 times the length between the apical lobes (Fig. 23e). If pale areas on abdomen distinct, then pale band on tergite IV covering slightly less than anterior half in dorsal view. Nearctic.	* E. curvata *
–	Sternite VIII with cleft deeper, depth about 0.5 times the length between the apical lobes (Fig. 37e). If pale areas on abdomen distinct, then pale band on tergite IV covering slightly more than anterior half in dorsal view. Nearctic.	* E. sphaerata *
6	Sternite VIII with cleft shallow and v-shaped (Fig. 12e). Spermathecal eminence in ventral view cross-shaped (as in Fig. 38f). Antennae short, about 1.4 times as long as length from vertex to ventral clypeal margin. Nearctic.	* E. breviflagellata *
–	Sternite VIII with cleft not v-shaped, usually deeper (Figs 17e, 31e, 38e). Antennae 1.6 times as long as length from vertex to ventral clypeal margin or longer.	7
7	Spermathecal eminence in ventral view unbranched (Fig. 17f). If pale areas on abdomen distinct, then extending somewhat dorsally along anterior margin of tergites III and IV, but not forming any complete bands. Nearctic.	* E. capillata *
–	Spermathecal eminence in ventral view cross-shaped (as in Fig. 38f).	8
8	If pale areas on abdomen distinct, then pale bands of tergites III and IV covering anterior fifth to third in dorsal view. Nearctic.	* E. repanda *
–	If pale areas on abdomen distinct, then pale bands narrower, covering anterior tenth to eighth in dorsal view or broken medially. Nearctic.	* E. subrepanda *

## Analysis

### Molecular species delimitation and sequence analysis

Of the 33 species included in this study, we were able to obtain DNA barcodes for 20 species. The remaining 12 species were delimited, based on morphology alone. DNA barcodes from two species, *E.ashleyi* and *E.burundiensis*, were not evaluated for inclusion into BINs as the sequence lengths were shorter than 500 bp. DNA barcodes from the remaining 18 species were divided into 16 BINs, of which 14 were congruent with our species delimitations; two BINs, however, were incrongruent as each included two species as delimited on morphology.

Genetic distances between species in the CO1 sequence data ranged from 0.012 between *E.brevilobata* and *E.breviflagellata* and 0.148 between *E.arcuata* and *E.rohdendorfi*, with an average of 0.076 ± 2SD [0.015-0.137] (Suppl. material 1). Distances within species ranged from 0 to 0.01 in *E.curvata* with an average of 0.005 ± 2SD [0-0.009] (Suppl. material 2).

**Figure 42.**
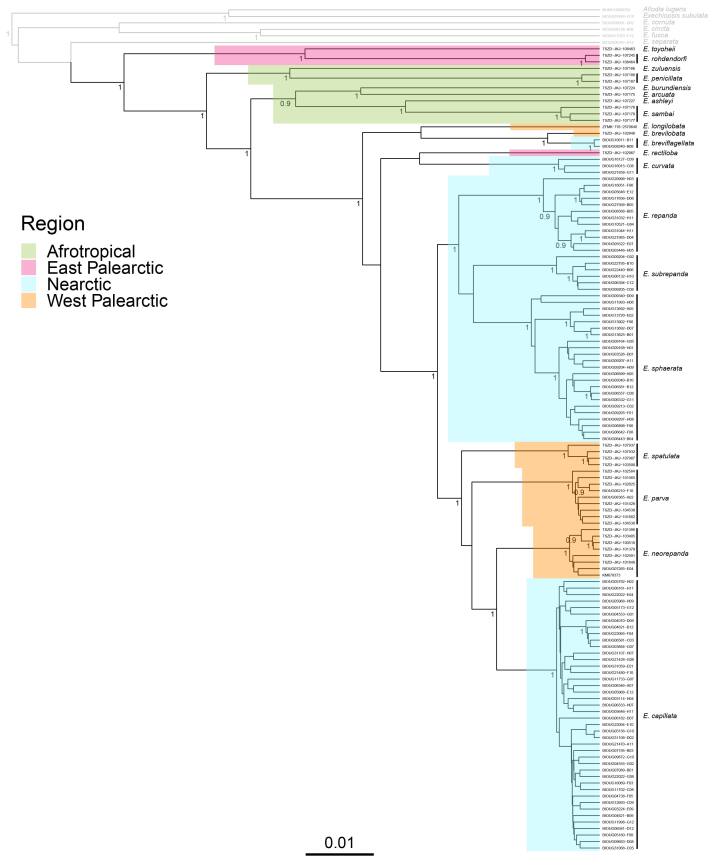
Bayesian tree of 20 species in the *E.parva* group, based on mitochondrial CO1 sequences. Ingroup clades are coloured according to zoogeographical origin of their respective terminal branches. Outgroup branches and tip labels indicated in grey. Posterior probabilities (≥ 0.95) indicated at the nodes. Terminal branches labelled with BOLD process IDs as voucher codes. Clades (indicated with bars) and singletons constituting species are labelled with species names. Scale = 0.01 divergence in the CO1 sequence.

The Bayesian tree, based on the CO1 data, indicates a monophyletic and well-supported *E.parva* group (Fig. 42), supporting the morphological delimitation of the species group. All species included in the analysis, if not singletons, constitute well-supported monophyletic clades. The species *E.rohdendorfi* and *E.toyoheii* form the most basal lineage, sister to the rest of the group.

## Discussion

### Species delimitation

The species treated in this study can mostly be delimited, based on distinct characters in the male terminalia. In some cases, however, the differences are less apparent. Most notably is the close similarities amongst the four species, *E.curvata*, *E.neorepanda*, *E.repanda* and *E.subrepanda*, which above all can be distinguished, based on slight allometric variations in the shape of the dorsal branch of the gonostylus (Figs 23c, 26c, 31c, 38c). Despite the morphological homogeneity, the species are all well supported in the Bayesian tree (Fig. 42) and with mean distances in the CO1 sequence ranging from 3-5% (Suppl. material 1).

In two cases, the molecular species delimitation, based on the BIN system, were not consistent with our delimitations. In both cases, two species ended up within a single BIN, where the BIN exhibits an Holarctic distribution, while the two included species each represent a Nearctic and a West Palaearctic distribution (Fig. 42). The first pair is *E.sphaerata* and *E.spatulata* within the BIN, BOLD:ACC3725 (dx.doi.org/10.5883/BOLD:ACC3725). These are morphologically distinct, based on the outline of the dorsal branch of the gonostylus, which, in *E.sphaerata* is short without a distinct apical lobe (Fig. 37c), closer to that of *E.repandoides*, while in *E.spatulata*, it is long and spathulate (Fig. 36c), closer to that of *E.longilobata*. The two species are also well separated in the Bayesian tree (Fig. 42). The second pair is *E.brevilobata* and *E.breviflagellata* within the BIN, BOLD:ACI6985 (dx.doi.org/10.5883/BOLD:ACI6985). These are undoubtedly very close both morphologically and genetically, forming sister lineages in the Bayesian tree (Fig. 42) and with only 0.012 mean distance in the CO1 sequence (Suppl. material 1). In this case, we have emphasised the slight variation present in the dorsal branch of the gonostylus (Figs 12c, 14c).

### Female characters

Our results fall in line with the general perception that female *Exechia* are more challenging than males to distinguish at the species level. Female terminalia have frequently been illustrated in earlier works (Lundström 1909, Chandler 1977, Matile 1978, Kurina 1999, Plassmann 1970) and, based on present knowledge, it is possible to determine most females to species group level and to some extent to species level. Within some species groups, however, species determination is limited by lack of published illustrations. Further, some cases with previously-published associations of females to males should be treated with care, although DNA barcoding will facilitate a safe quality check of these. In the *E.parva* group, previously, only the female terminalia of *E.parva* have been illustrated (Chandler 1977). We have described some female characters, based on limited material which could be associated with males, mainly through clustering of DNA barcodes. However, most females remain unknown and some of those described are more or less inseparable. Despite these difficulties, we present a key, hoping that this can encourage further work with females of this group.

### Biogeography

Intercontinental barriers appear to have a strong impact on species segregation in the *E.parva* group, as each of the species is not found in more than a single zoogeographical region. Although two BINs in the *E.parva* group were shared between the West Palaearctic and Nearctic Regions, the intergrative solution in both cases was to treat them as different species. Similar patterns are known from other genera in the family (e.g. Magnussen et al. 2018, Magnussen et al. 2019). Still, a great number of Mycetophilidae species are shared between the West and East Palaearctic Subregions and the Palaearctic and Nearctic Regions share some 9% of their common BIN pool of Mycetophilidae (Kjærandsen 2017b, fresh data pulled from BOLD). Other intercontinental species distributions are very sparse (< 1% of BINs, fresh data pulled from BOLD) and, in some of these cases, anthropogenic spreading is likely involved (e.g. Toft and Chandler 2004). Traditionally, a substantial proportion of the Scandinavian Mycetophilidae species was considered to have a Holarctic distribution, for example, 27% of the Swedish species were also reported from the Nearctic Region (Kjærandsen et al. 2007). However, DNA barcoding is now improving and narrowing the species delimitation of many Mycetophilidae species (see Kjærandsen and Søli 2020) and, hence, the proportion of Nordic species shared with the Nearctic Region is likely to decrease to around 20% (current BIN data) or lower.

We have further recognised several complexes in the group that are more or less congruent with the zoogeographic divisions. *E.rohdendorfi, E.toyoheii, E.crassiseta* and *E.trunciseta* can be clustered, based on the medial gonostylus branch darkened and with at least three elongated apical setae (from couplet 7, male key). This group can further be divided into two distinct clusters, based on the shape of the dorsal branch of the gonostylus and the setae on the ventral margin of each gonocoxite. The first cluster consisting of *E.rohdendorfi* and *E.toyoheii* (from couplet 8, male key), has an east Palaearctic distribution, with a centre in Japan where both species are collected (Fig. 10). In the Bayesian tree, *E.rohdendorfi* and *E.toyoheii* form the most basal lineage in the *E.parva* group (Fig. 42). The second cluster, consisting of *E.crassiseta* and *E.trunciseta* (from couplet 9, male key), are native to the eastern Himalayas in the Oriental Region (Fig. 19). The relationship between these two clusters is consistent with the open transition between the Oriental and the east Palaearctic Regions, where China and Japan sometimes are considered to be located in the transition zone between the two Regions (Heiser and Schmitt 2013).

The Afrotropical species, *E.afroparva*, *E.sambai*, *E.penicillata*, *E.burundiensis* and *E.ashleyi*, although highly variable in the male terminalia, can be clustered, based on the internal branch of the gonostylus apically with 3 or 4 apical setae gathered together on or close to the apex (from couplet 11, male key). The Bayesian tree suggests that also *E.arcuata* and *E.zuluensis* are part of this cluster, which is not monophyletic, but constitute two clades forming a paraphyletic assemblage (Fig. 42). This split corresponds to the geographic division between the South African species (*E.penicillata* and *E.zuluensis*) and the species from East African montane forest (*E.burundiensis*, *E.arcuata*, *E.ashleyi* and *E.sambai*, Fig. 4); however, we have not seen this pattern reflected in the morphology. The Afrotropical fauna (Fig. 4) accounts for a large part of the *E.parva* group and shows considerably higher morphological variability compared to the Holarctic species, which is consistent with the relatively large genetic distances between these species, ranging from 0.079 to 0.13 (Suppl. material 1). Following Papadopoulou et al. (2010), with an estimated divergence in CO1 of 3.54% My-1, these distances indicate speciation between 2.3 to 3.7 Ma, i.e. during late Pliocene and Pleistocene. Here, it is important to add that rates of molecular evolution may vary considerably amongst lineages and the available estimates may, therefore, not be accurate for our group of study (e.g. Wiegmann et al. 2003). Compared to the Afrotropical species of another Exechiini genus, *Allodia* Winnertz, with genetic distances in CO1 ranging from 0.056 to 0.101 (Magnussen et al. 2018), this implies a diversification of the Afrotropical *Exechia* in the same, or a slightly earlier timeframe. We can assume that similar factors have caused speciation in the two genera, possibly environmental fluctuations during Pleistocene leading to isolation in highland refuges and allopatric speciation (Magnussen et al. 2018, see Fjeldsaå and Lovett 1997 for more on Afromontane refuge theory), however, with a somewhat differing time of colonisation.

All species keying out in the male key from couplets 17 and 22, including most of the previously-described members of the *E.parva* group, occur in the Holarctic Region (Figs 10, 13, 15), except for *E.adenaparva* which is described from the Arabian Peninsula (Fig. 4). These can be clustered, based on a combination of the following characters: the internal branch of the gonostylus apically with one seta on or close to the apex, the hypandrium with apical pair of setae relatively short, the gonocoxal lobe with apico-internal margin angled exteriorly and the medial branch of the gonostylus without apical setae. In the Bayesian tree, this complex forms a large apical clade, sister to the Afrotropical lineage composed of *E.afroparva*, *E.ashleyi*, *E arcuata* and *E.burundiensis* (Fig. 42). The morphological variation in this complex is relatively homogeneous compared to the remaining species, something that is reflected by the relatively shorter branch lengths of these species, indicationg a more recent Holarctic radiation within the *E.parva* group.

Additionally, the species *E.columna* and *E.serrae*, both collected in the eastern Himalayas (Fig. 19), were not sequenced, but are morphologically very close (from couplet 4, male key) and very distinct from all other species in the group.

### Biology

The biological information about the group is very scarce for many species partly due to the limited material available for, for example, phenological studies and partly to the fact that few rearing experiments or larval studies have been carried out. The studied material is generally associated with forested, more or less damp and cool areas and, outside temperate and boreal regions (the Afrotropical and Oriental Regions), all material has been sampled from higher altitudes. As far as we know, most of the material has been collected in forest habitats, with the exception of *E.longilobata*, which is only known from a limestone quarry in Sweden. A connection to more open woodland with grasses and/or mires is noted for some of the northern species, especially late in the season when they seek umbelliferous stems for hibernation (see Introduction). What we know about host preferences, only based on records from the Holarctic species, *E.parva*, *E.neorepanda*, *E.capillata* and *E.repandoides*, indicates that the group attacks a wide range of fungi within Agaricales and occasionally also species of Russulales and Pezizales (for references, see the biology-sections below taxon treatments).

### Terminology

Some of the terms used in this article are used with a certain reservation, at least when considering homologous traits. According to Søli (1997), the aedaegal guides in the male terminalia are derived from the hypandrium and probably homologous to the hypandrial lobe; however, they are used in instances when the structures are derived from between the base of the aedaegus and the base of the gonocoxites. Since what we refer to as hypandrial lobes are clearly derived from the hypandrium, it is possible that our use of the term aedaegal guides is misapplied. In the *E.parva* group, these appear to be derived from below the ventral margin of each gonocoxite (Fig. 2a). Similar structures can be seen in a few species of the *Exechiacincta* group, although usually larger and more distinct and sometimes covered with small setae. One possibility is that they are homologous to what Søli (1997) refers to as "section 3" of the gonocoxites.

Additionally, the spermathecal eminence is here used to describe the sclerotised structure at the opening (gonopore) of the spermatheca (Figs 3a, 34e). This should be in accordance with the definition by Søli (1997) where "The spermathecal eminence [...] carries the openings of the two spermathecal ducts, [...]".

### Sampling effort

The sampling efforts in the different regions are highly divergent. Accordingly, most of the Afrotropical and Oriental species, described here, are based on singletons, i.e. only known from a single collecting event. Moreover, the geographical coverage in these Regions is very low, for example, the material from the Afrotropical Region is limited to five unique collecting events, leaving huge areas blank. Considering the low sampling effort in these Regions, one must assume that only a fraction of the total diversity has been recorded. Species representing other species groups in *Exechia* are well known from the Oriental and Afrotropical Regions (own materials, Matile 1980) and we know of several undescribed taxa in the *E.cincta* group from the Afrotropical Region. On the other hand, in the West Palaearctic and Nearctic Regions, most species are recorded multiple times at multiple locations and it is possible that we have reached a level where the main portion of the fauna have been described. New species are, however, likely to be discovered also in these Regions.

## Supplementary Material

61EF9A61-1A57-59A0-8864-17EE5A93DC2F 10.3897/BDJ.9.e67134.suppl1 Supplementary material 1 Table 1 Data type genomic Brief description Genetic distances between species File: oo_486770.pdf https://binary.pensoft.net/file/486770 Jon Peder Lindemann

A0B6FC94-740D-56DB-A640-97E27554ADB0 10.3897/BDJ.9.e67134.suppl2 Supplementary material 2 Table 2 Data type genomic Brief description Genetic distances within species File: oo_486769.pdf https://binary.pensoft.net/file/486769 Jon Peder Lindemann

5D00C45F-EB83-5486-9EF1-89C0AE22534D 10.3897/BDJ.9.e67134.suppl3 Supplementary material 3 R script Data type script Brief description R scripts for analysing CO1 sequence data File: oo_498007.zip https://binary.pensoft.net/file/498007 Jon Peder Lindemann

XML Treatment for Exechia adenaparva

XML Treatment for Exechia afroparva

XML Treatment for Exechia arcuata

XML Treatment for Exechia ashleyi

XML Treatment for Exechia bifasciata

XML Treatment for Exechia brachiata

XML Treatment for Exechia breviflagellata

XML Treatment for Exechia brevilobata

XML Treatment for Exechia burundiensis

XML Treatment for Exechia capillata

XML Treatment for Exechia chirotheca

XML Treatment for Exechia cinctiformis

XML Treatment for Exechia columna

XML Treatment for Exechia crassiseta

XML Treatment for Exechia curvata

XML Treatment for Exechia longichaeta

XML Treatment for Exechia longilobata

XML Treatment for Exechia neorepanda

XML Treatment for Exechia pararepanda

XML Treatment for Exechia parva

XML Treatment for Exechia penicillata

XML Treatment for Exechia rectiloba

XML Treatment for Exechia repanda

XML Treatment for Exechia repandoides

XML Treatment for Exechia rohdendorfi

XML Treatment for Exechia sambai

XML Treatment for Exechia serrae

XML Treatment for Exechia spatulata

XML Treatment for Exechia sphaerata

XML Treatment for Exechia subrepanda

XML Treatment for Exechia toyoheii

XML Treatment for Exechia trunciseta

XML Treatment for Exechia zuluensis

## Figures and Tables

**Figure 1a.**
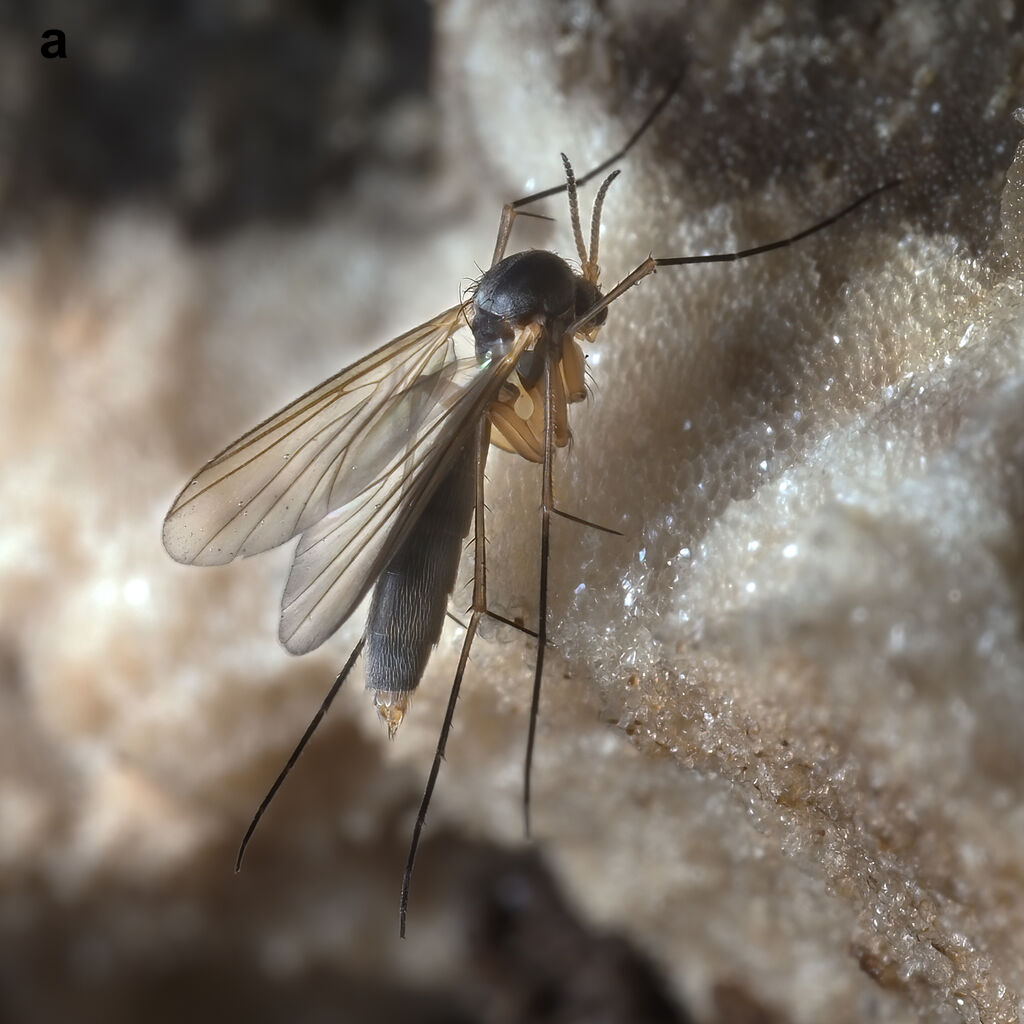
Male

**Figure 1b.**
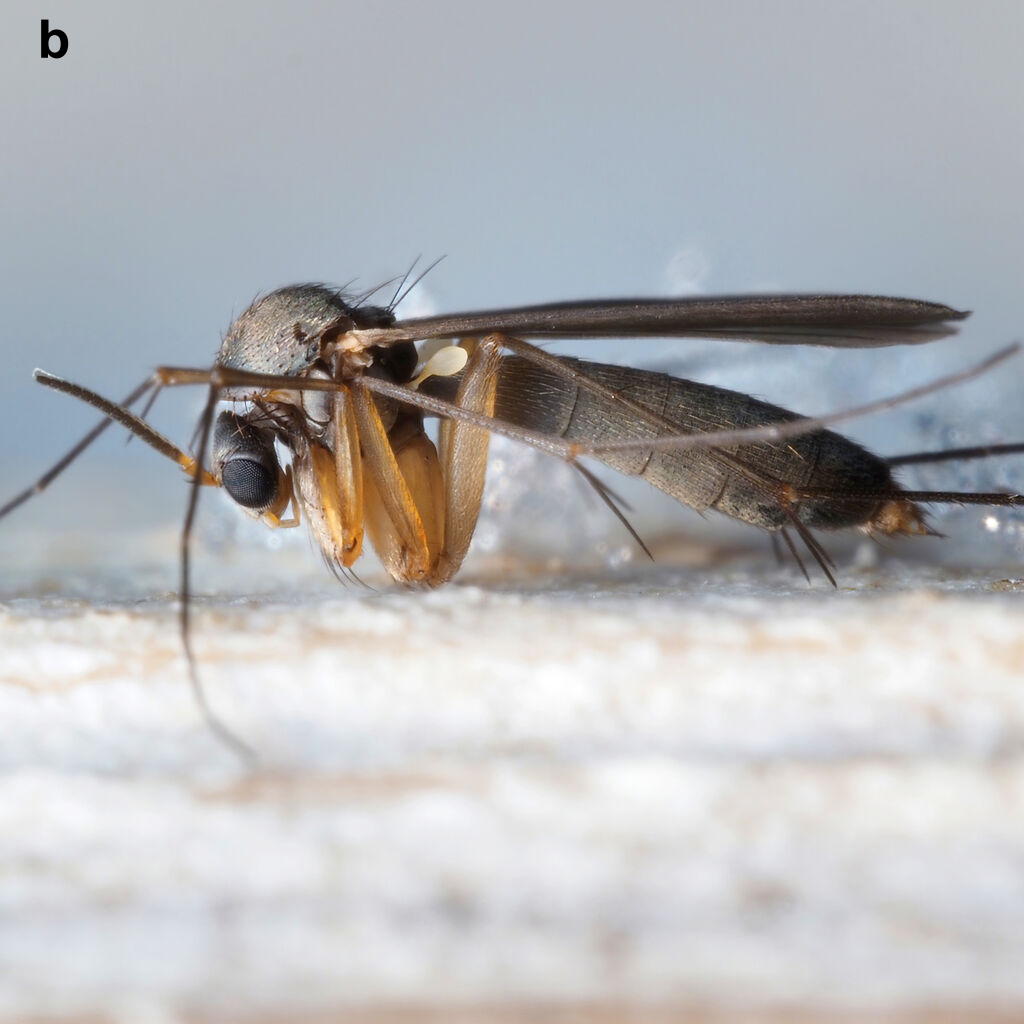
Male

**Figure 1c.**
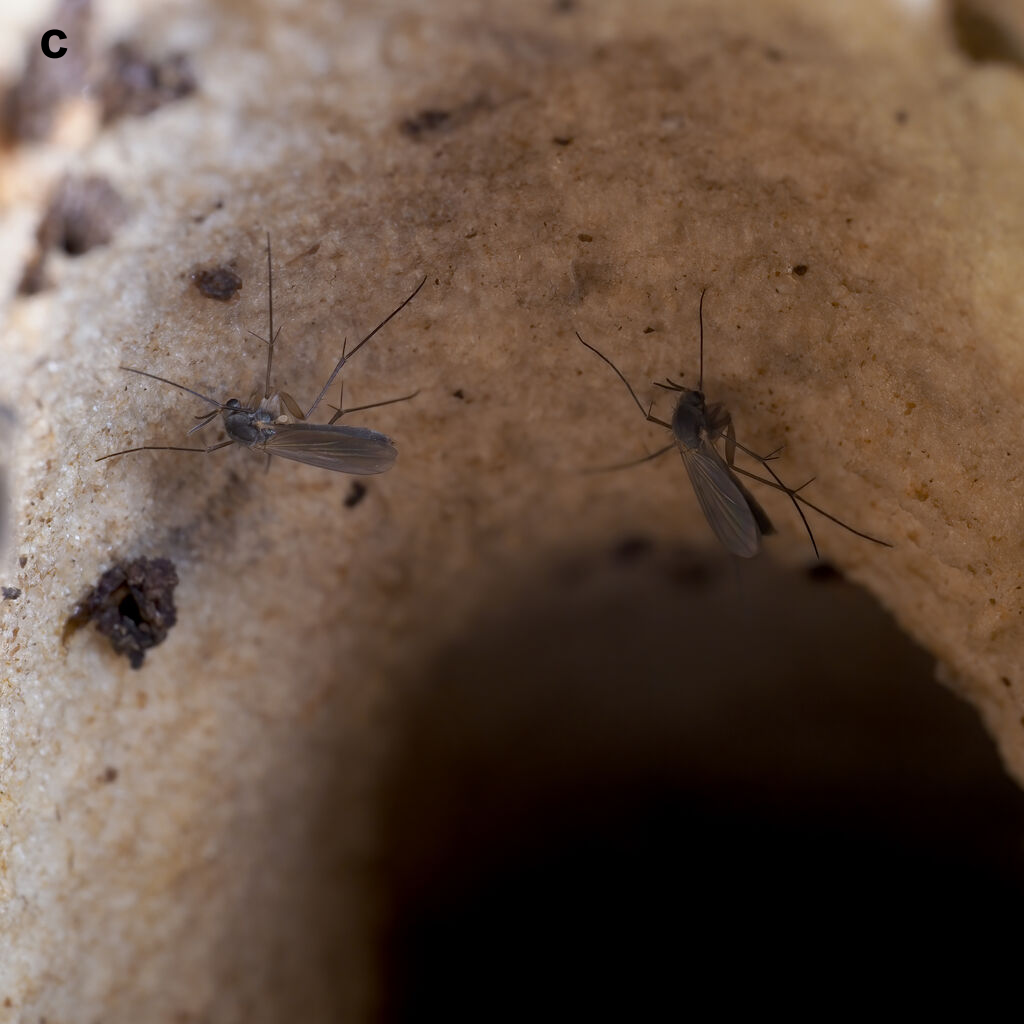
Hibernation inside a hollow stem of *Heracleumpersicum*

**Figure 1d.**
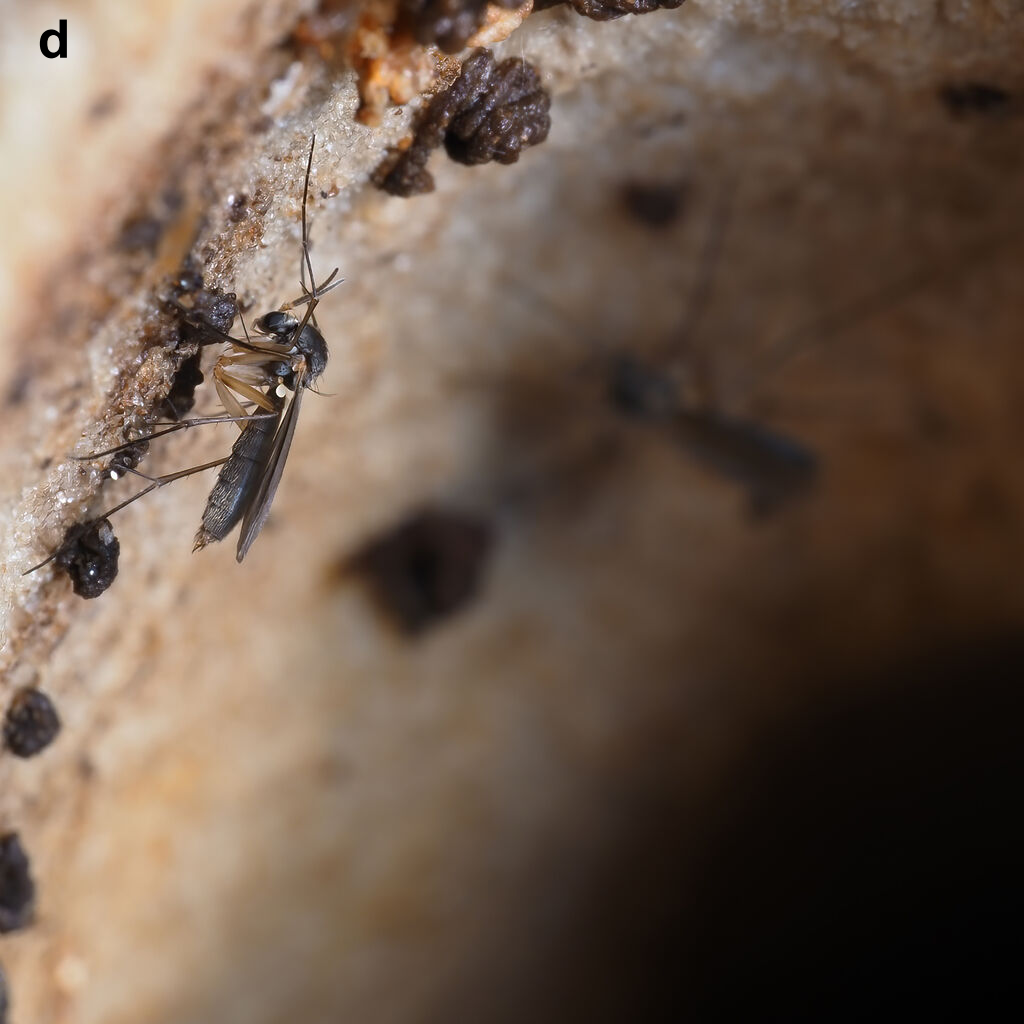
Hibernation inside a hollow stem of *Heracleumpersicum*

**Figure 2a.**
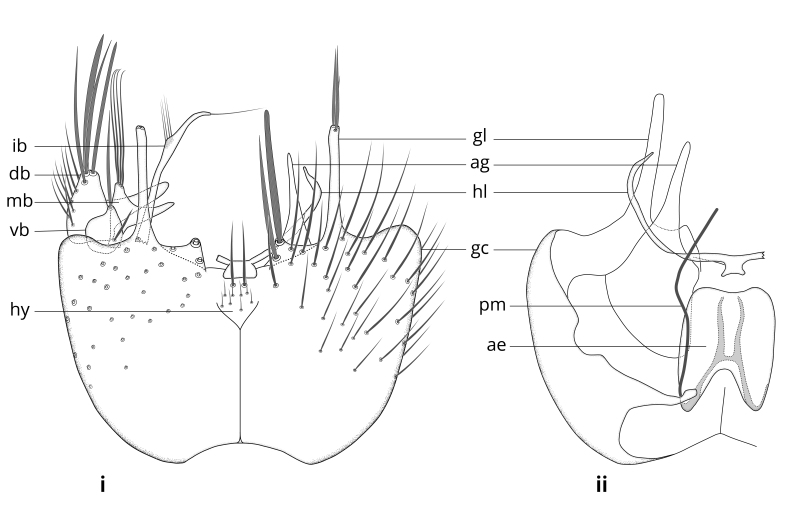
Terminalia of *E.toyoheii* sp. n. in: **i** ventral view with left gonocoxal setae and right gonostylus not drawn and **ii** dorsal view with setae, gonostyli, cerci and parts of right side not drawn.

**Figure 2b.**
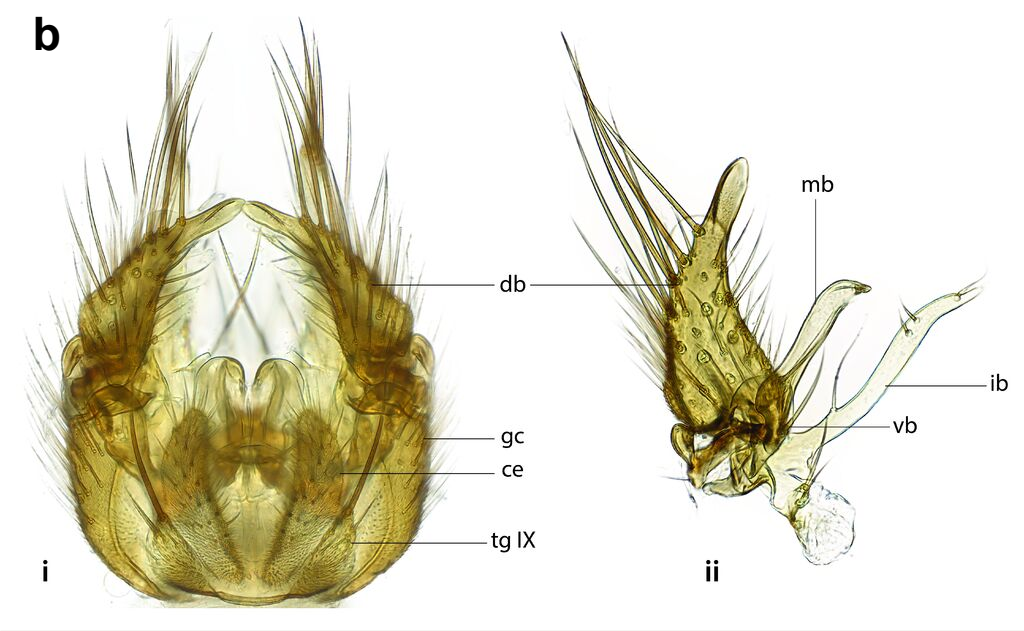
Terminalia of *E.repanda* Johannsen, 1912 in: **i** dorsal view and **ii** dissected gonostylus in dorsal view.

**Figure 3a.**
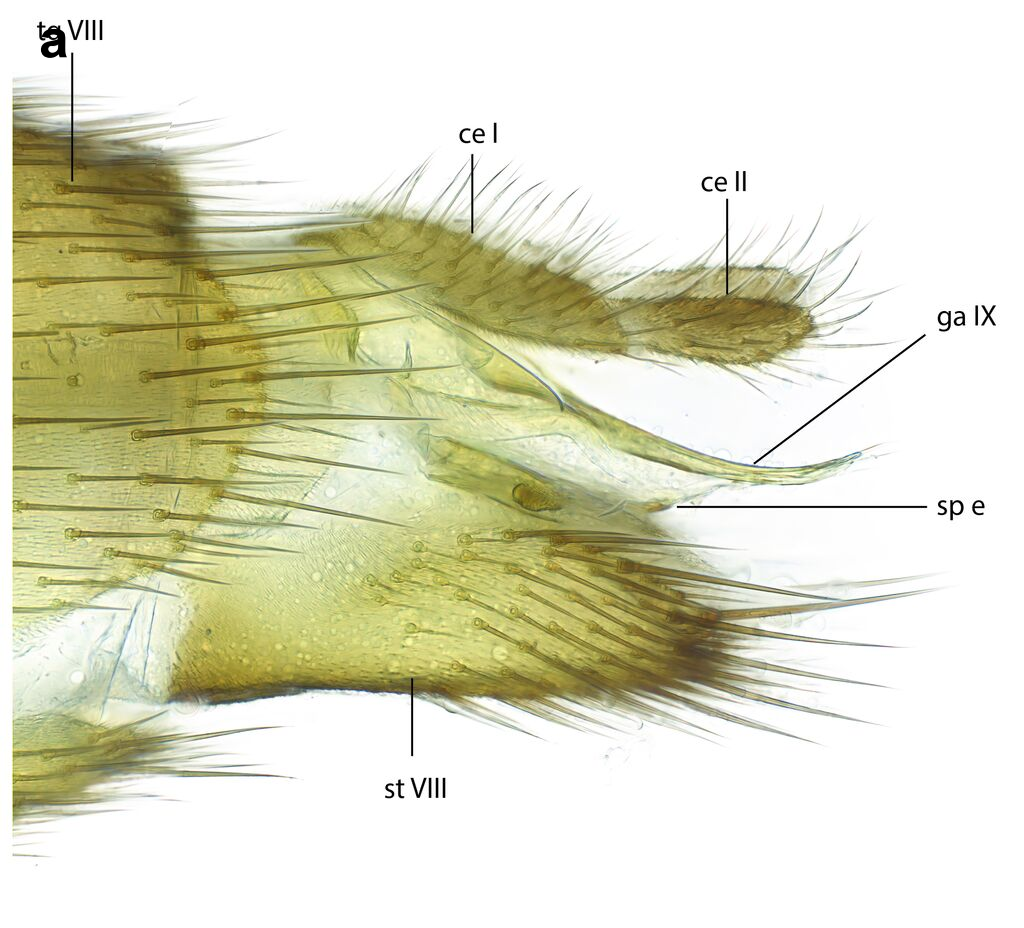
Lateral view.

**Figure 3b.**
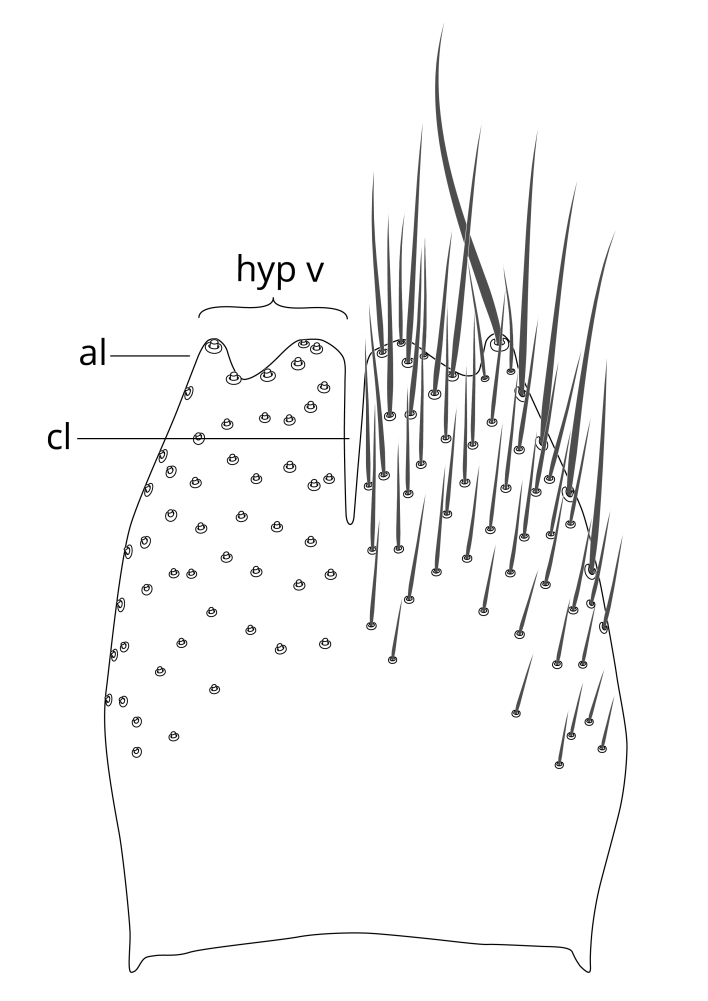
Sternite VIII, ventral view.

**Figure 5a.**
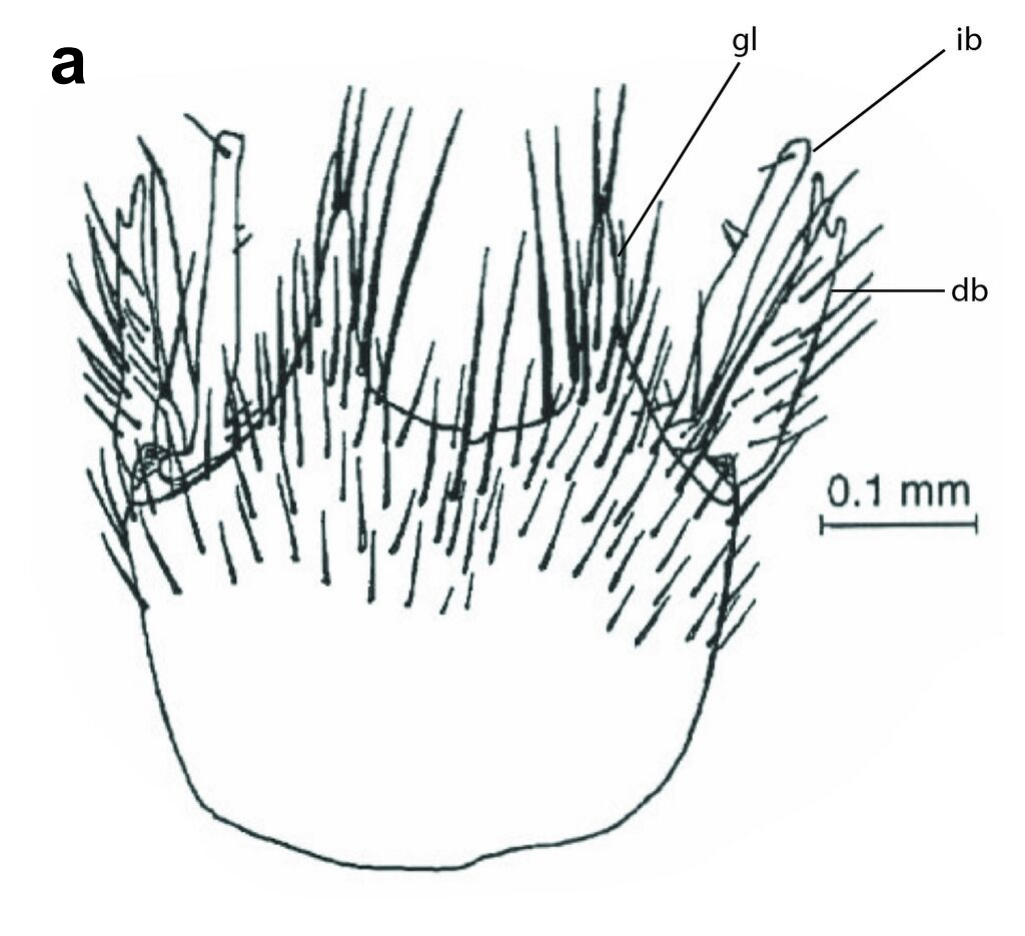
Male terminalia, ventral view. Reprinted with permission from Chandler (2000).

**Figure 5b.**
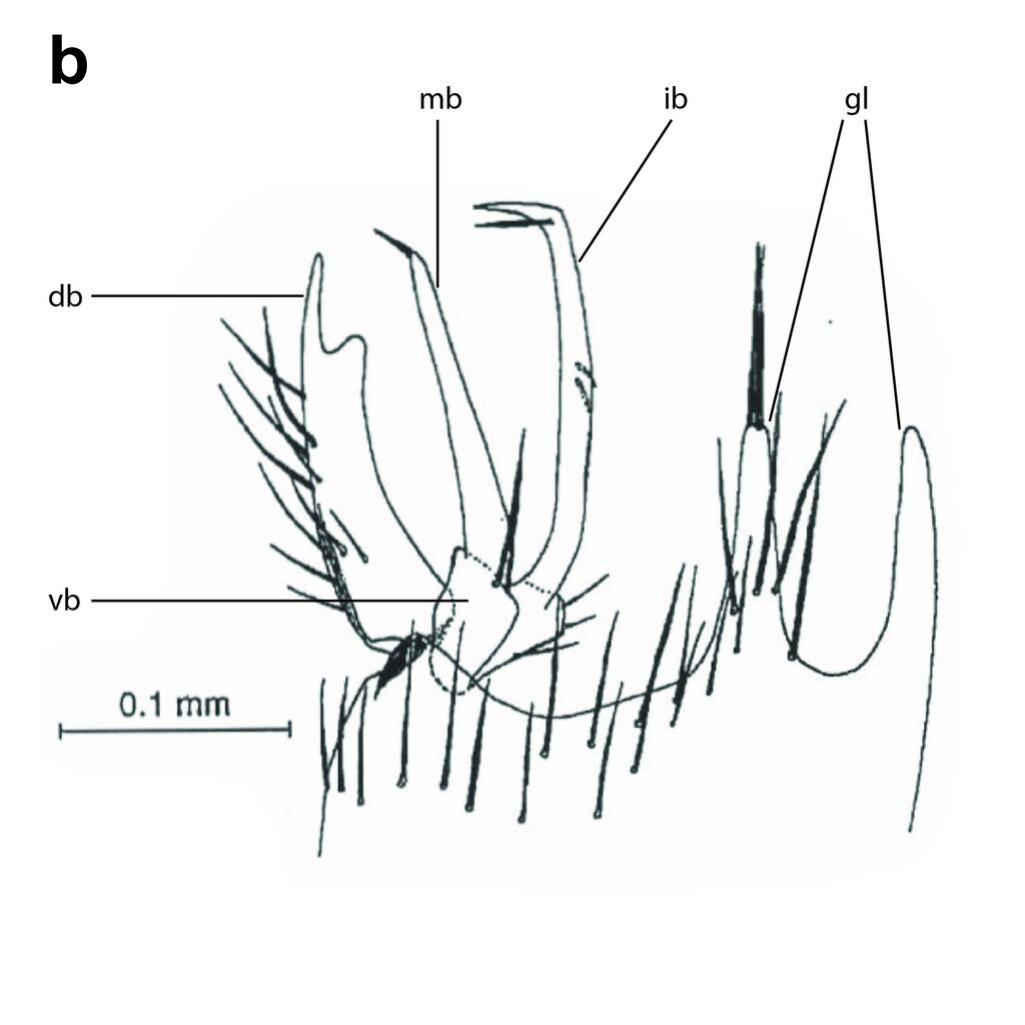
Male terminalia, lateral view. Reprinted with permission from Chandler (2000).

**Figure 5c.**
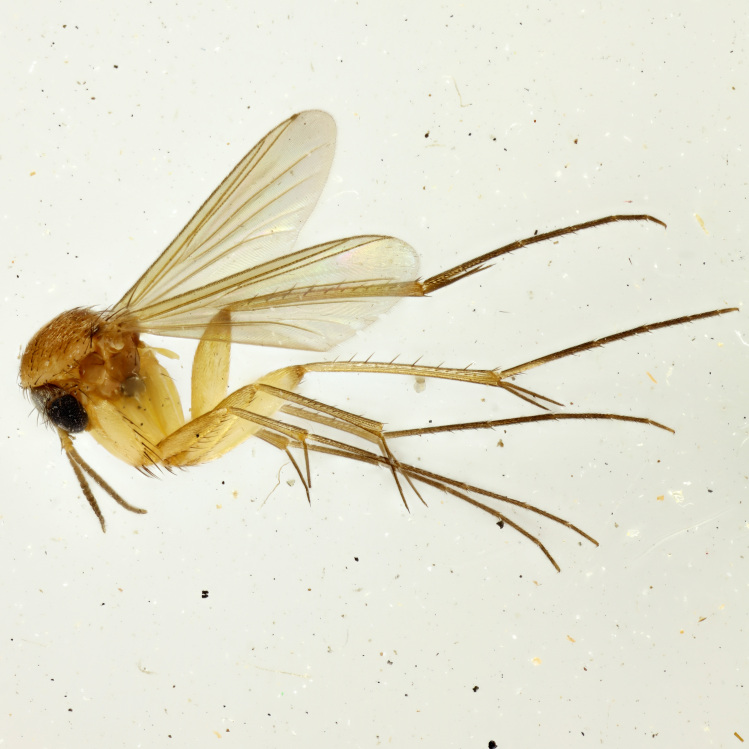
Holotype excluding abdomen. Photo by courtesy of Erica McAlister, NHM.

**Figure 5d.**
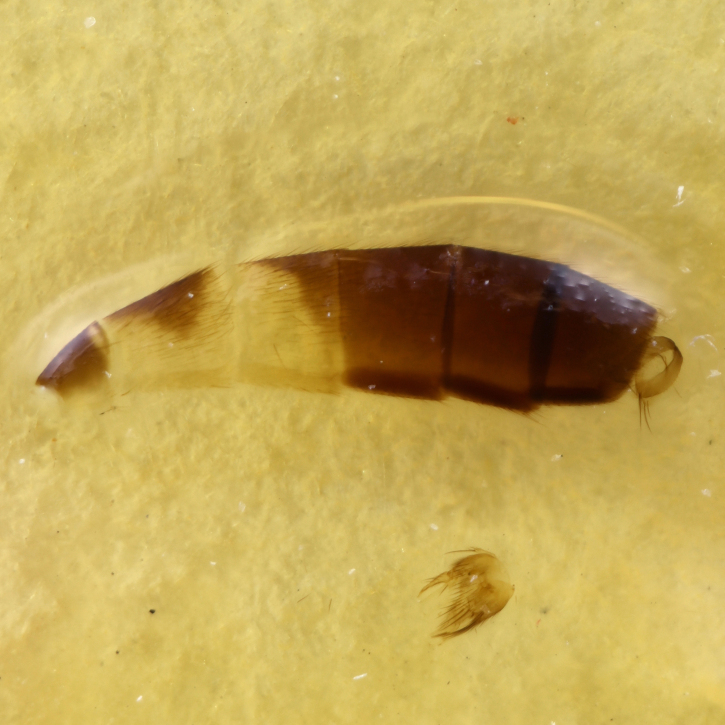
Holotype abdomen and terminalia. Photo by courtesy of Erica McAlister, NHM.

**Figure 6a.**
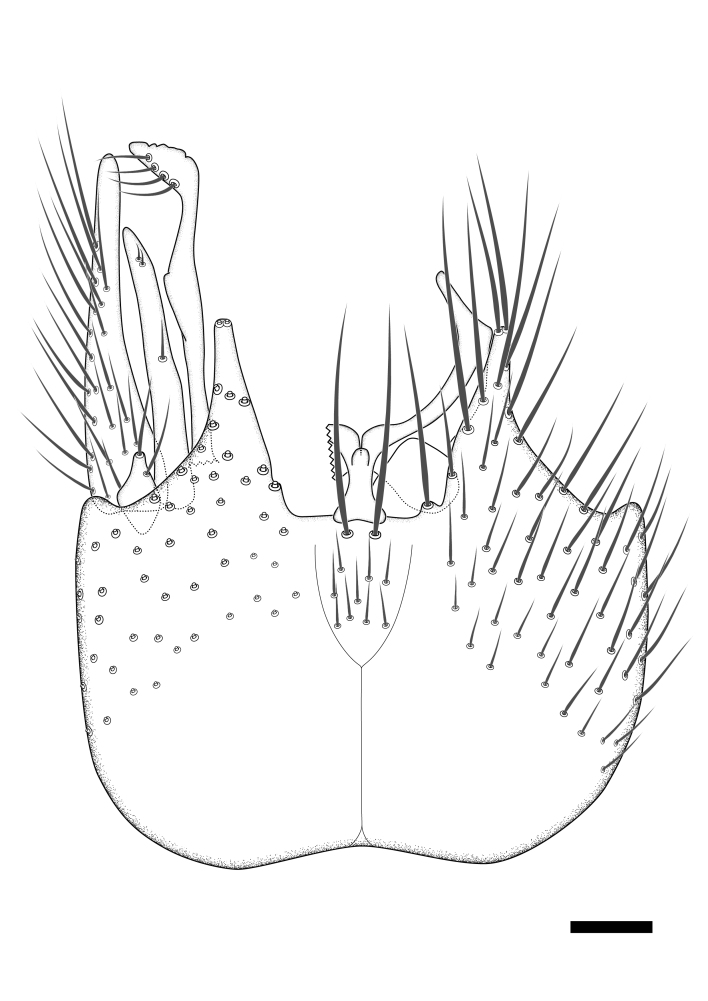
Ventral view. Right gonostylus and setae on left gonocoxite not drawn. Scale = 50 μm.

**Figure 6b.**
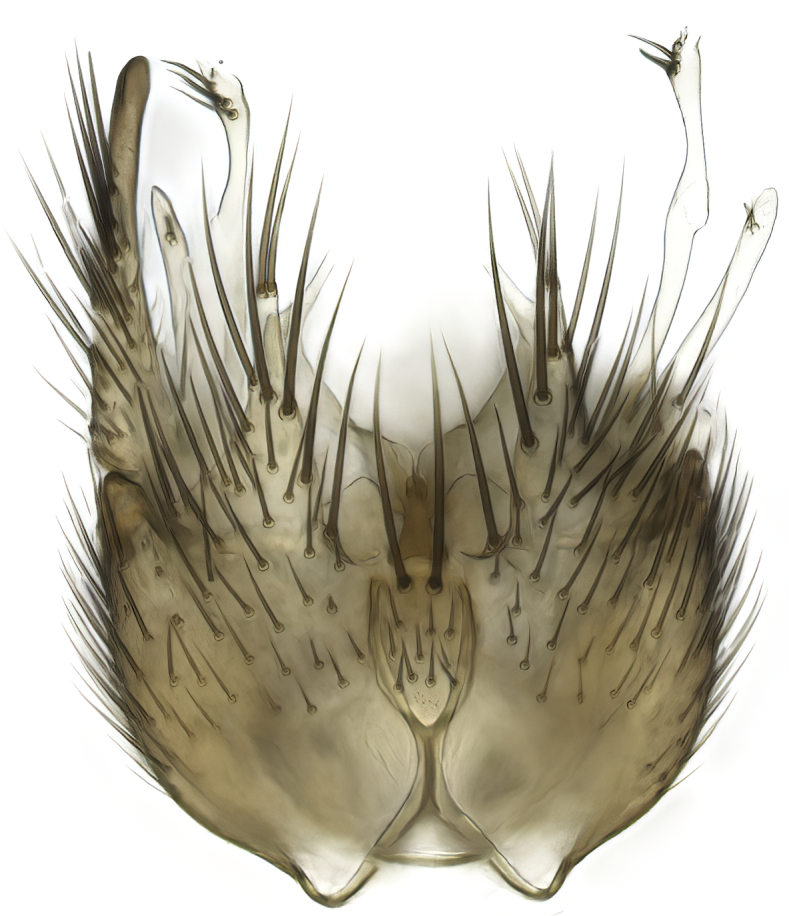
Ventral view. Photo.

**Figure 6c.**
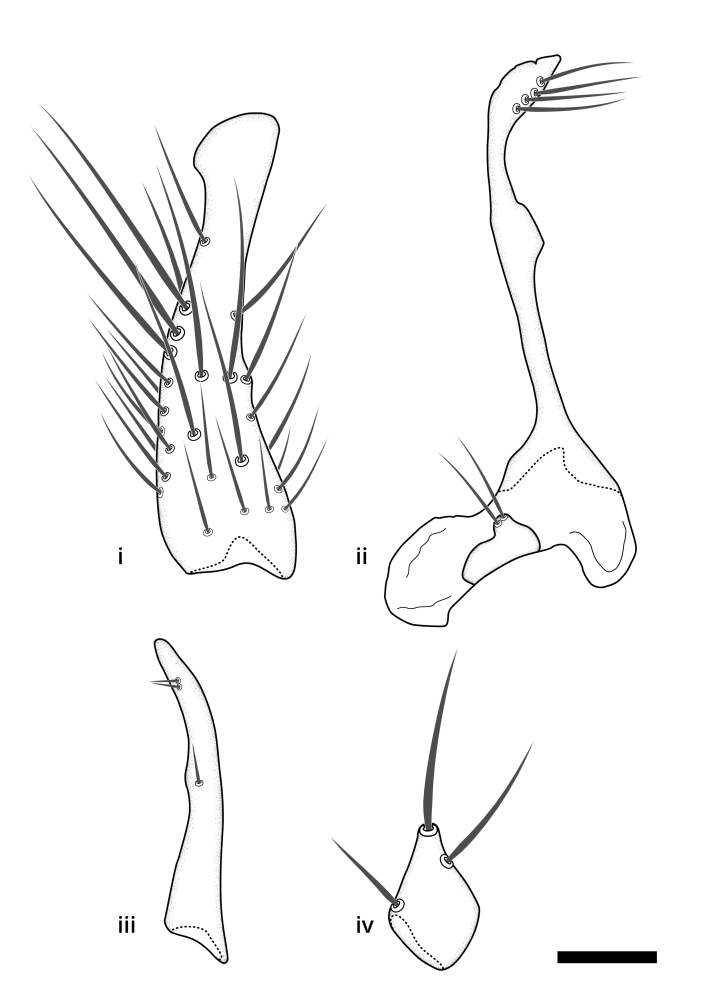
Right gonostylus with: **i** dorsal branch, **ii** internal branch, **iii** medial branch and **iv** ventral branch separated. Scale = 50 μm.

**Figure 7a.**
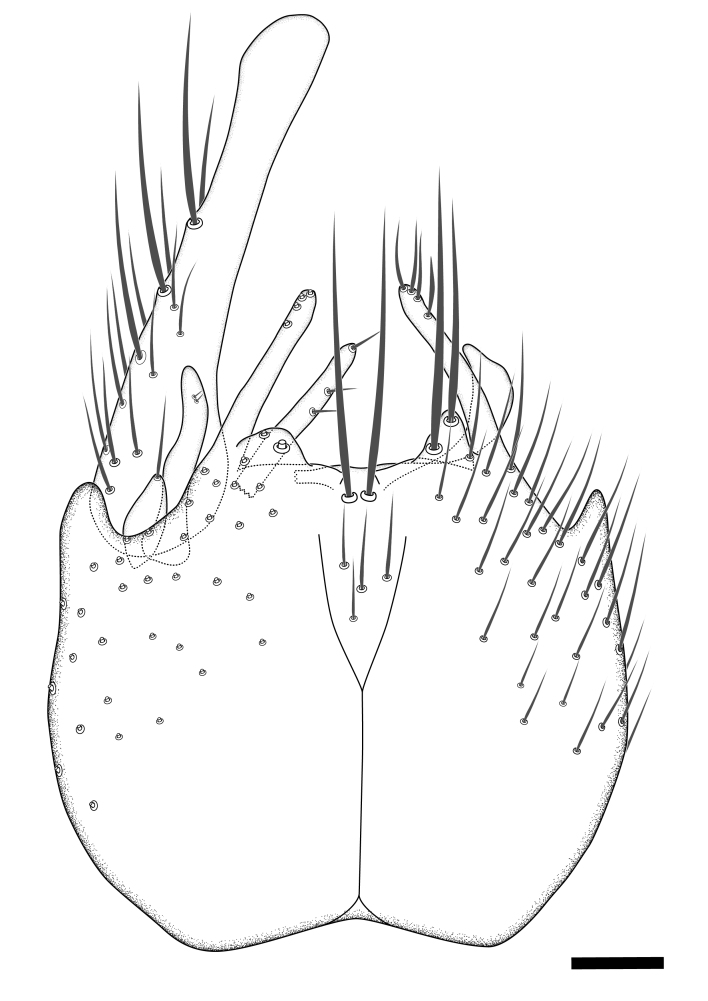
Ventral view. Right gonostylus and setae on left gonocoxite not drawn. Scale = 50 μm.

**Figure 7b.**
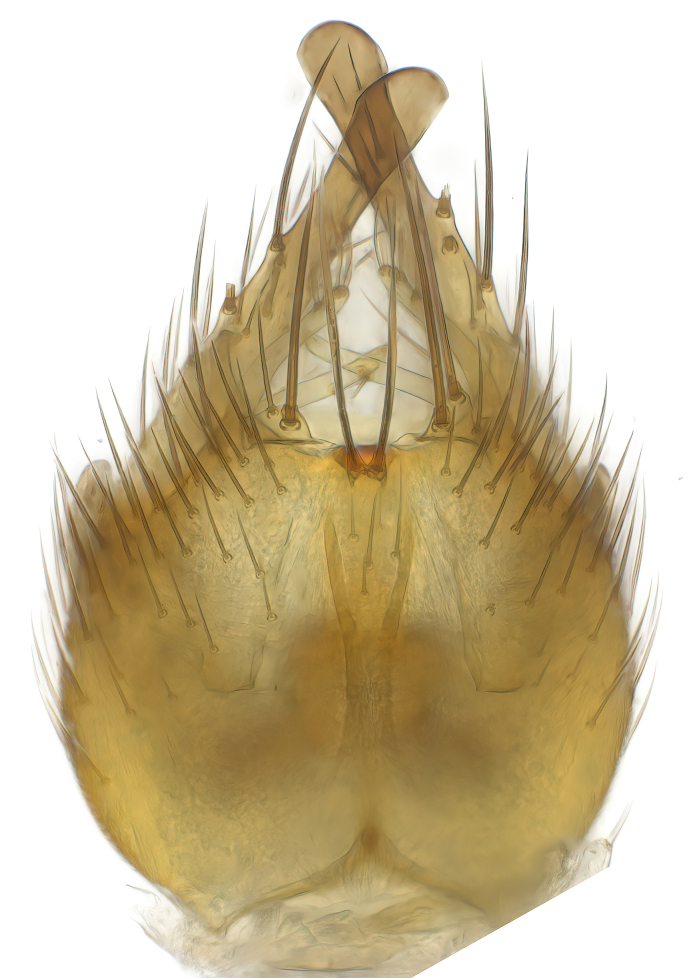
Ventral view. Photo.

**Figure 7c.**
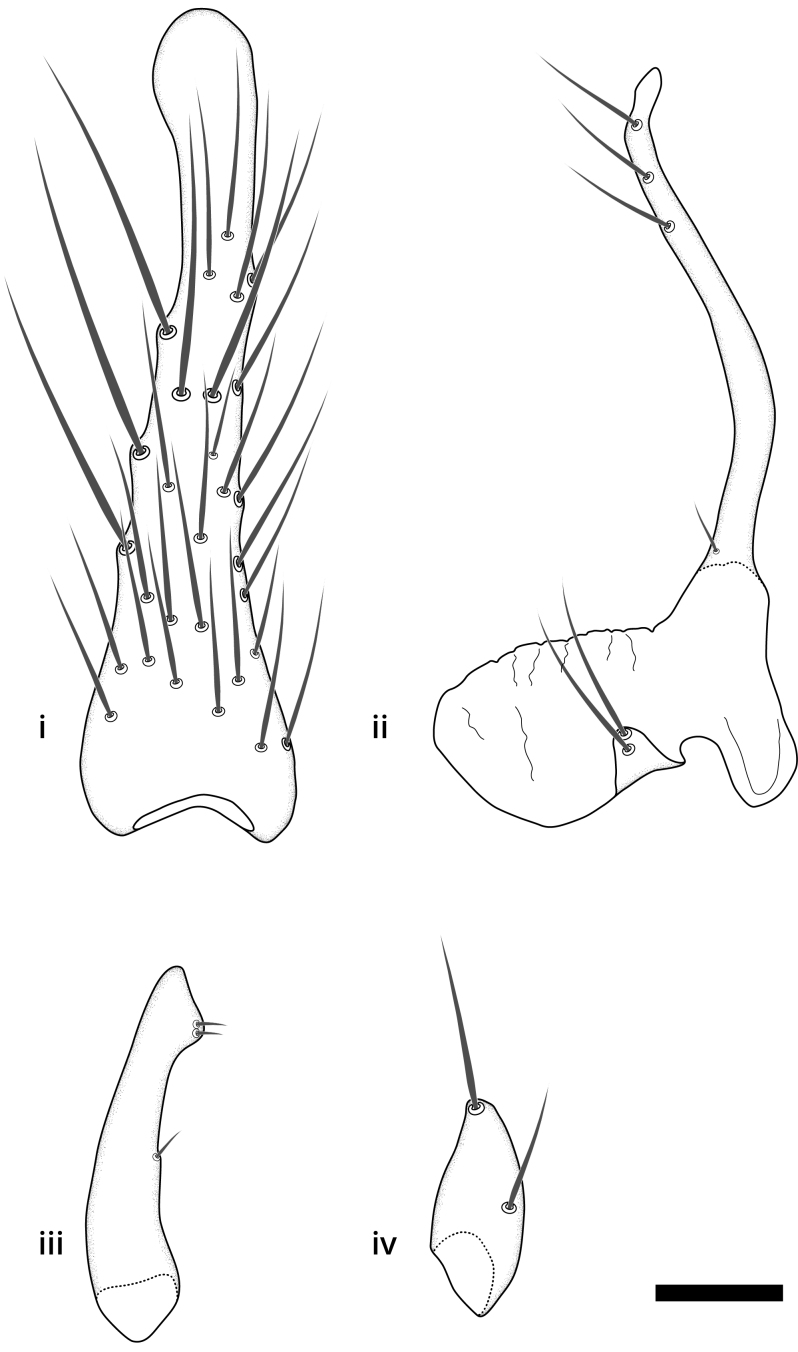
Right gonostylus with: **i** dorsal branch, **ii** internal branch, **iii** medial branch and **iv** ventral branch separated. Scale = 50 μm.

**Figure 8a.**
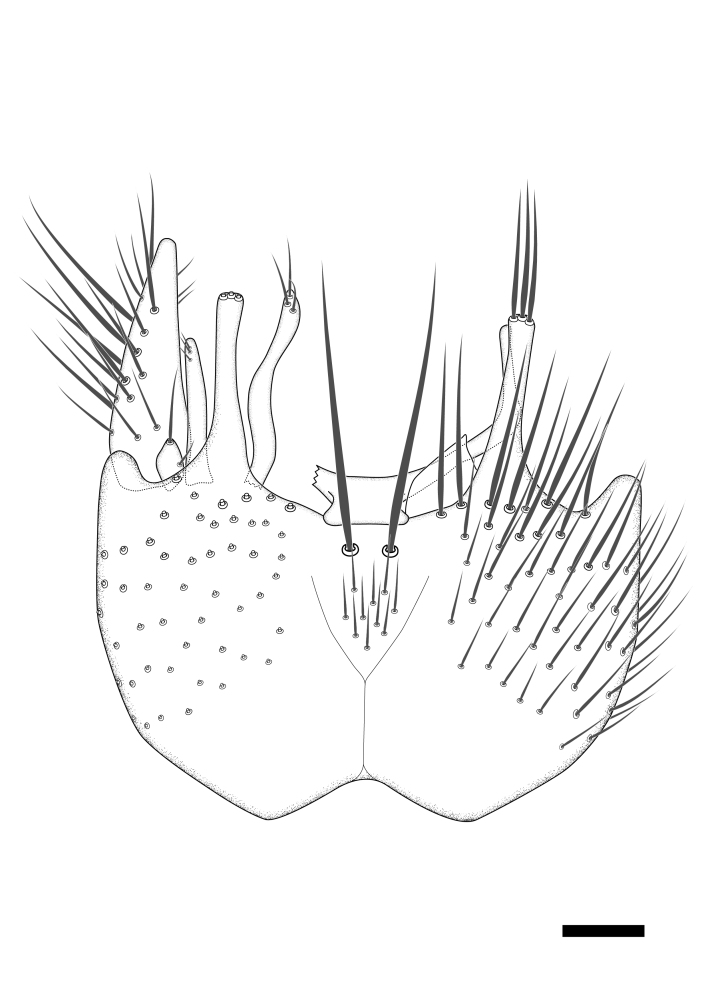
Ventral view. Right gonostylus and setae on left gonocoxite not drawn. Scale = 50 μm.

**Figure 8b.**
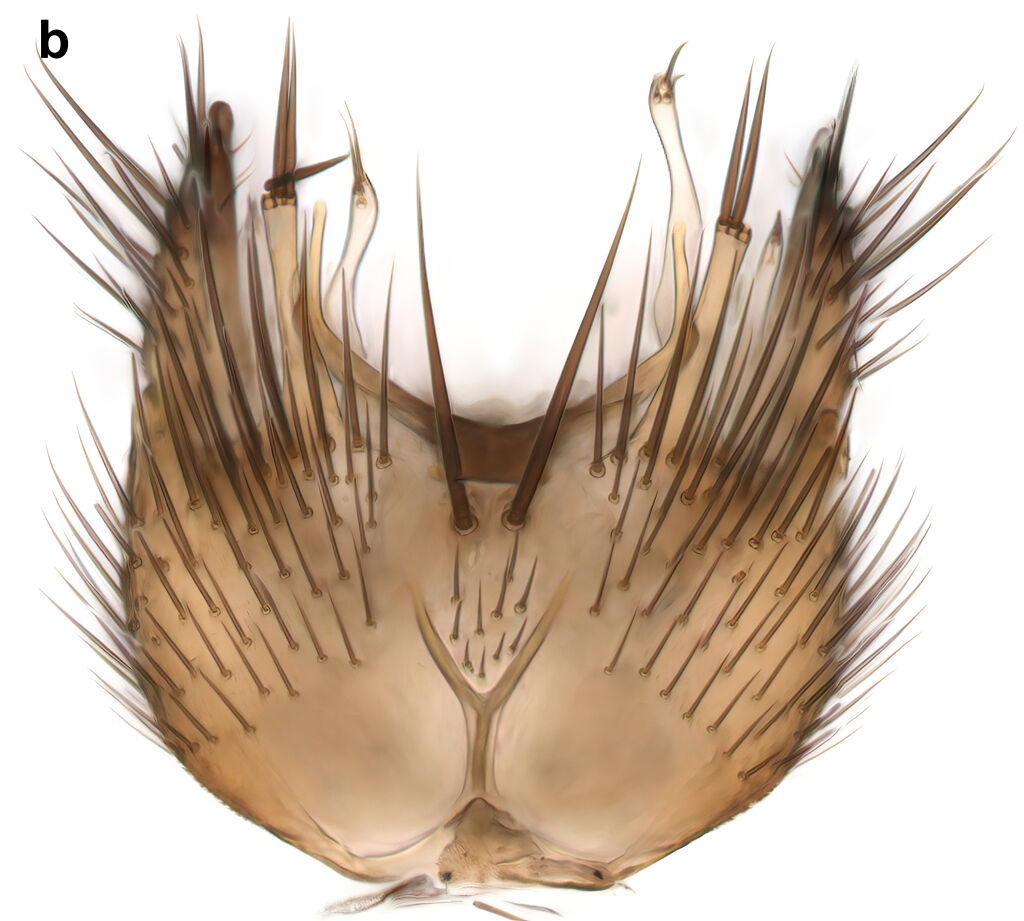
Ventral view. Photo.

**Figure 8c.**
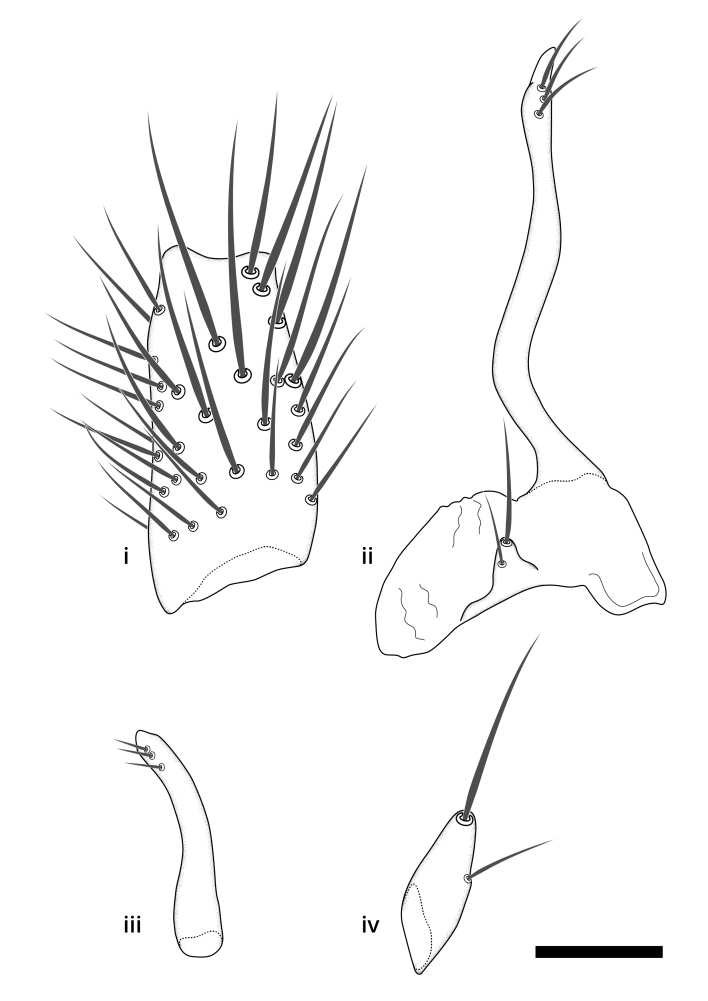
Right gonostylus with: **i** dorsal branch, **ii** internal branch, **iii** medial branch and **iv** ventral branch separated. Scale = 50 μm.

**Figure 9a.**
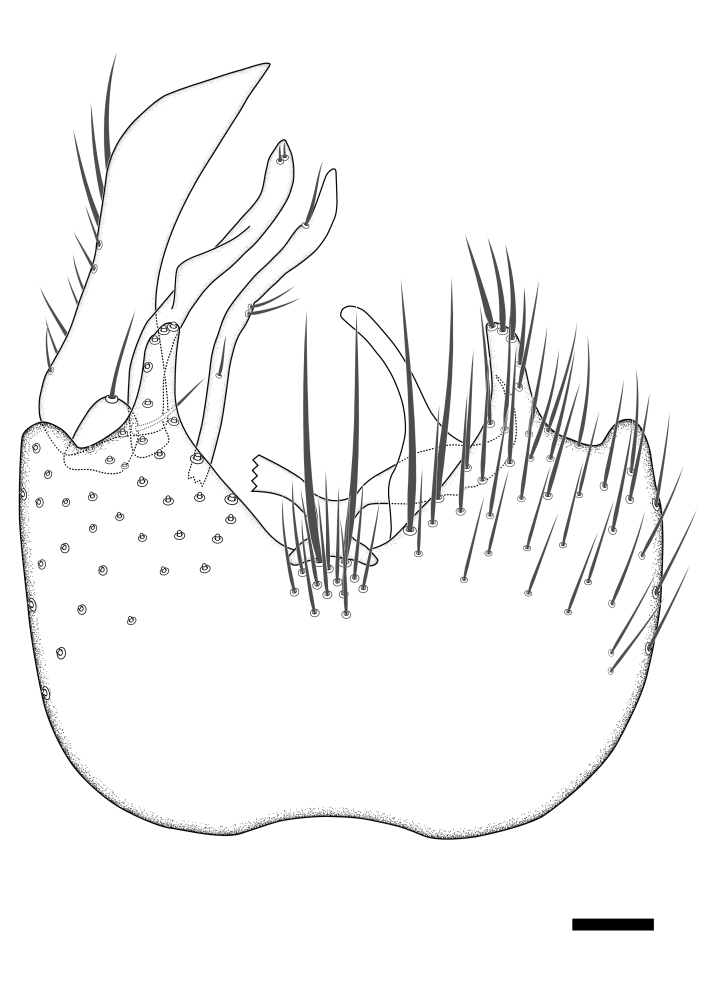
Male terminalia ventral view. Right gonostylus and setae on left gonocoxite not drawn. Scale = 50 μm.

**Figure 9b.**
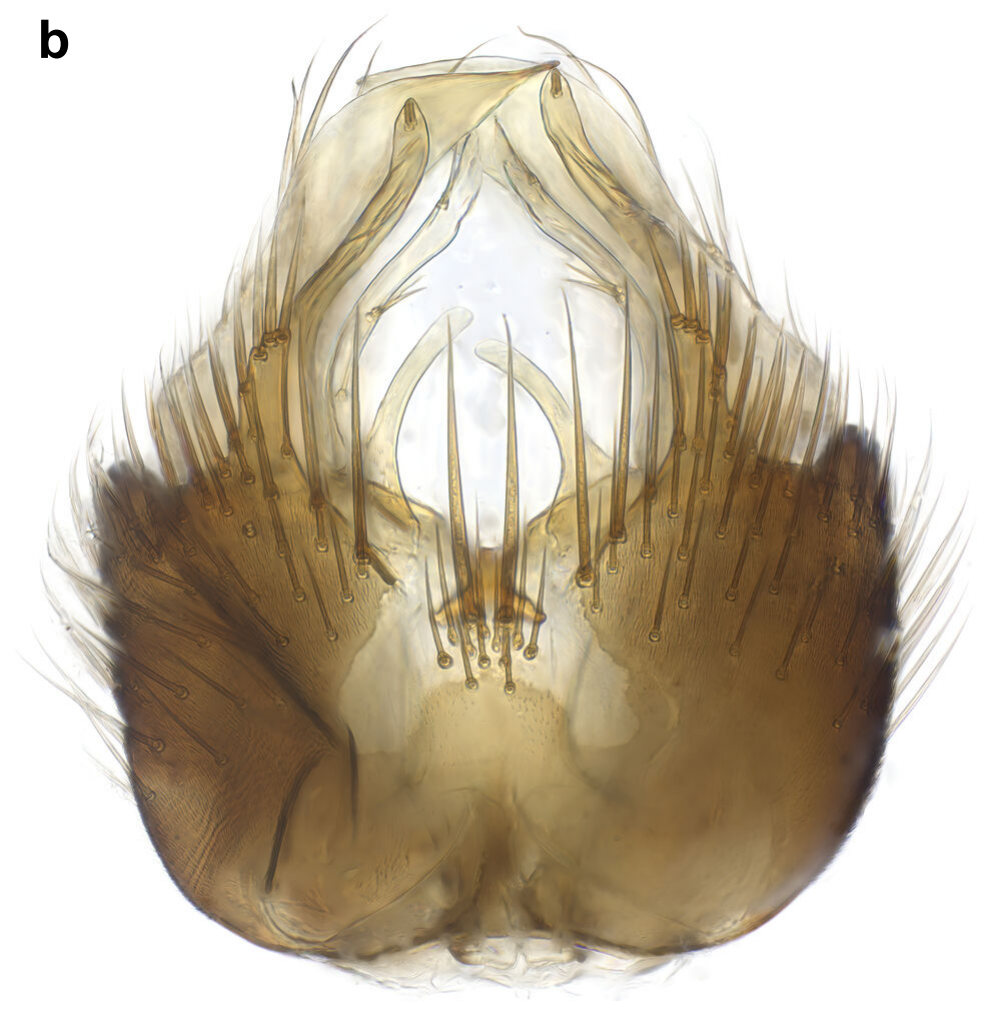
Male terminalia ventral view. Photo.

**Figure 9c.**
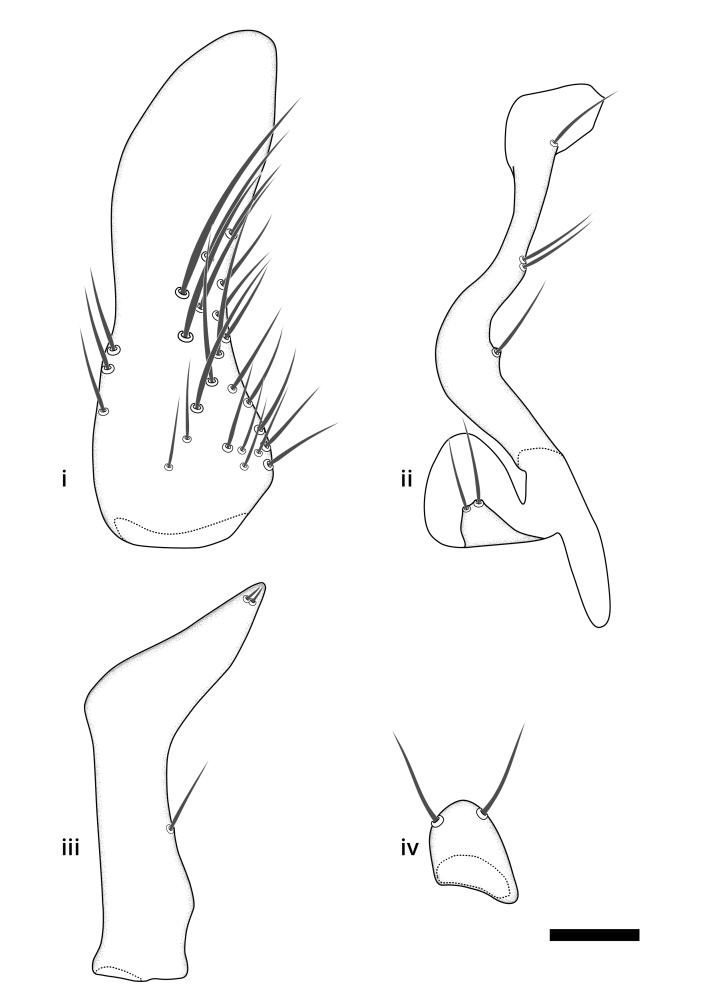
Male right gonostylus with: **i** dorsal branch, **ii** internal branch, **iii** medial branch and **iv** ventral branch separated. Scale = 50 μm.

**Figure 9d.**
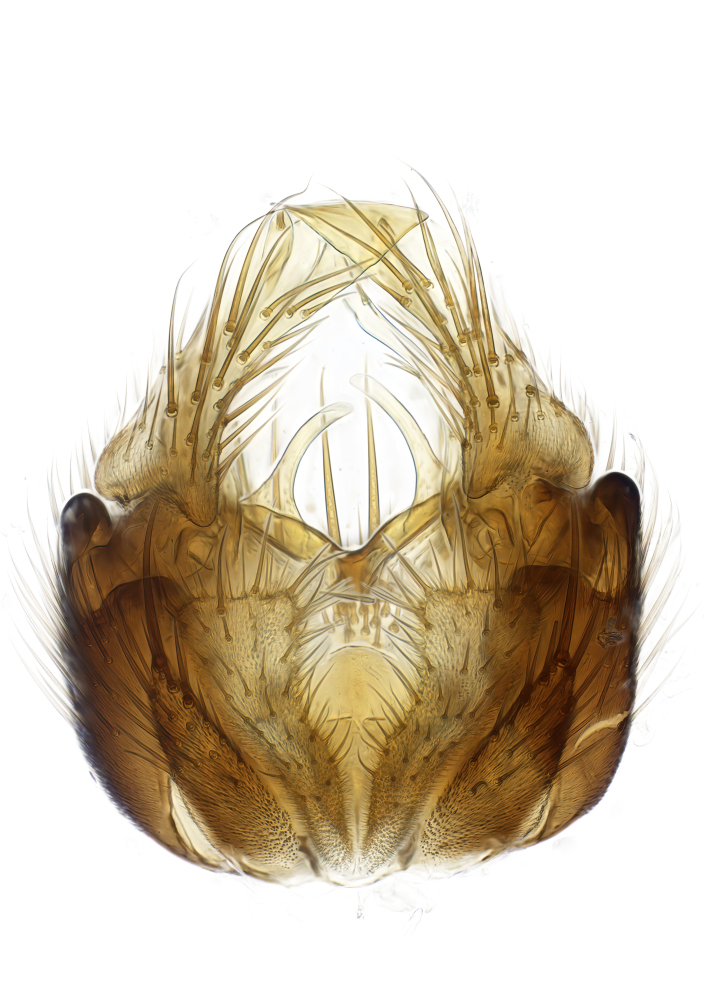
Male terminalia dorsal view. Photo.

**Figure 9e.**
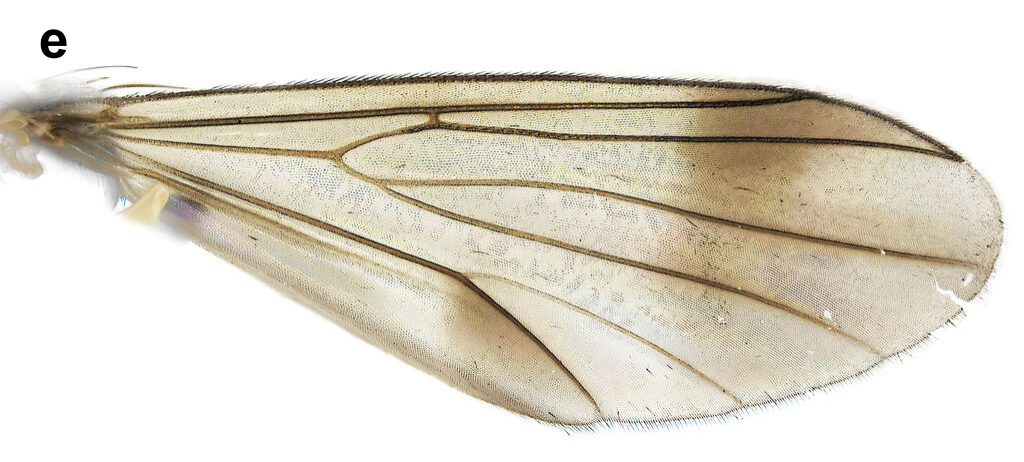
Right wing. Photo.

**Figure 11a.**
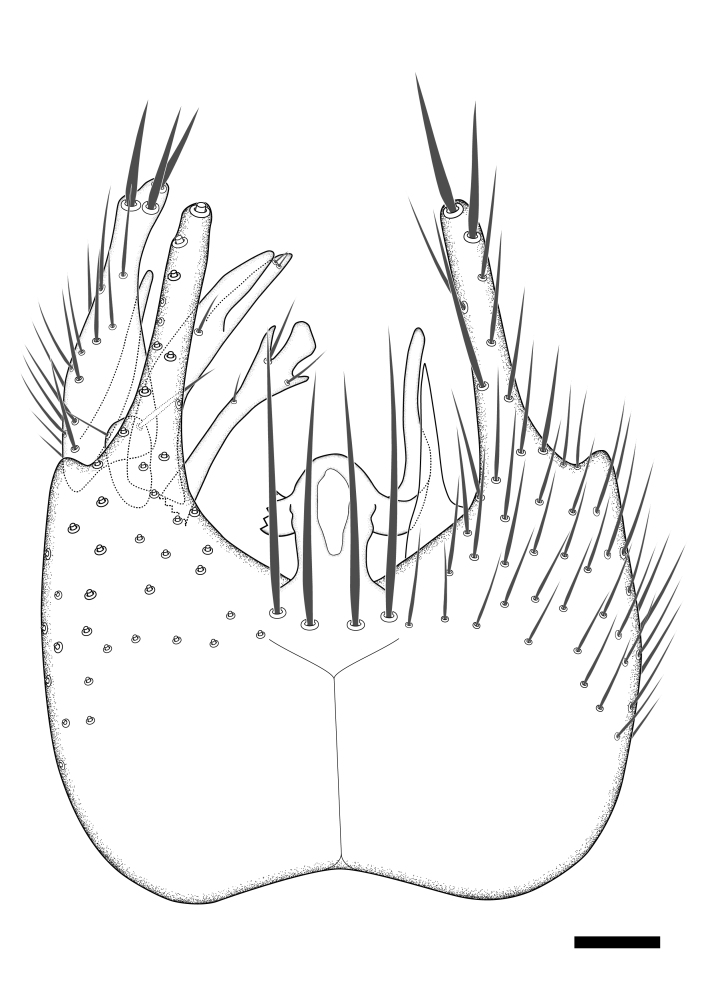
Ventral view. Right gonostylus and setae on left gonocoxite not drawn. Scale = 50 μm.

**Figure 11b.**
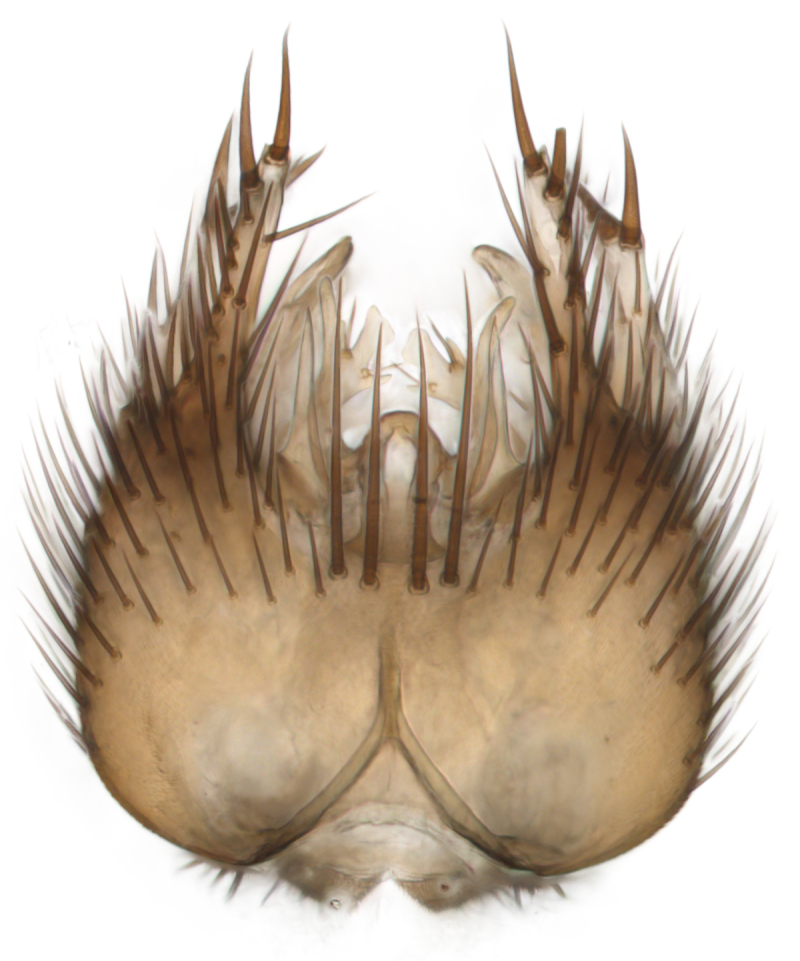
Ventral view. Photo.

**Figure 11c.**
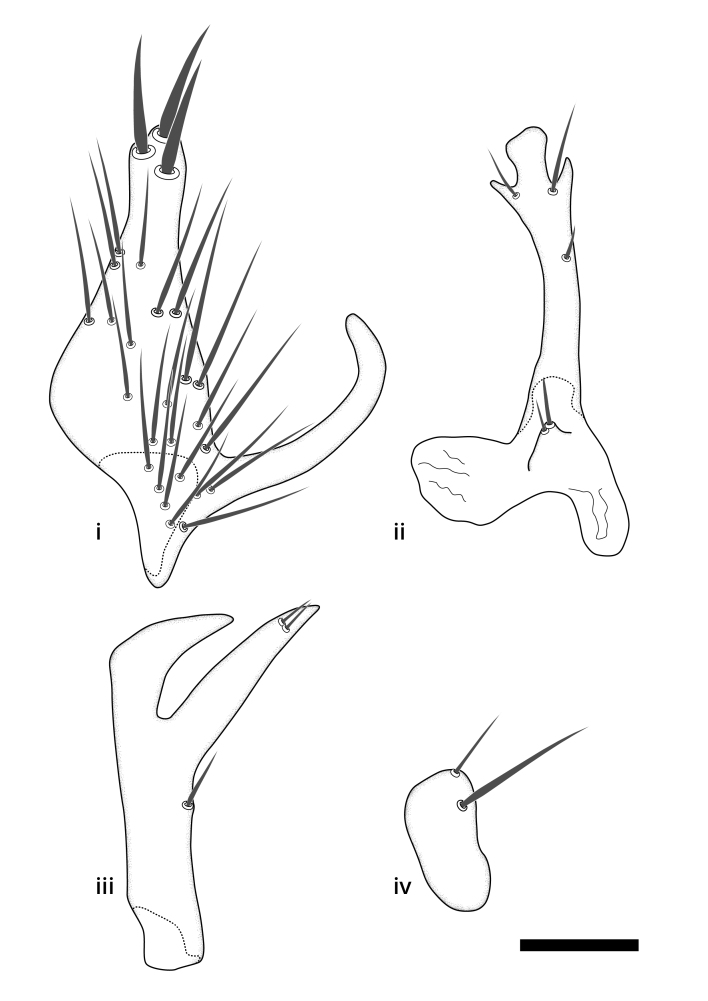
Right gonostylus with: **i** dorsal branch, **ii** internal branch, **iii** medial branch and **iv** ventral branch separated. Scale = 50 μm.

**Figure 12a.**
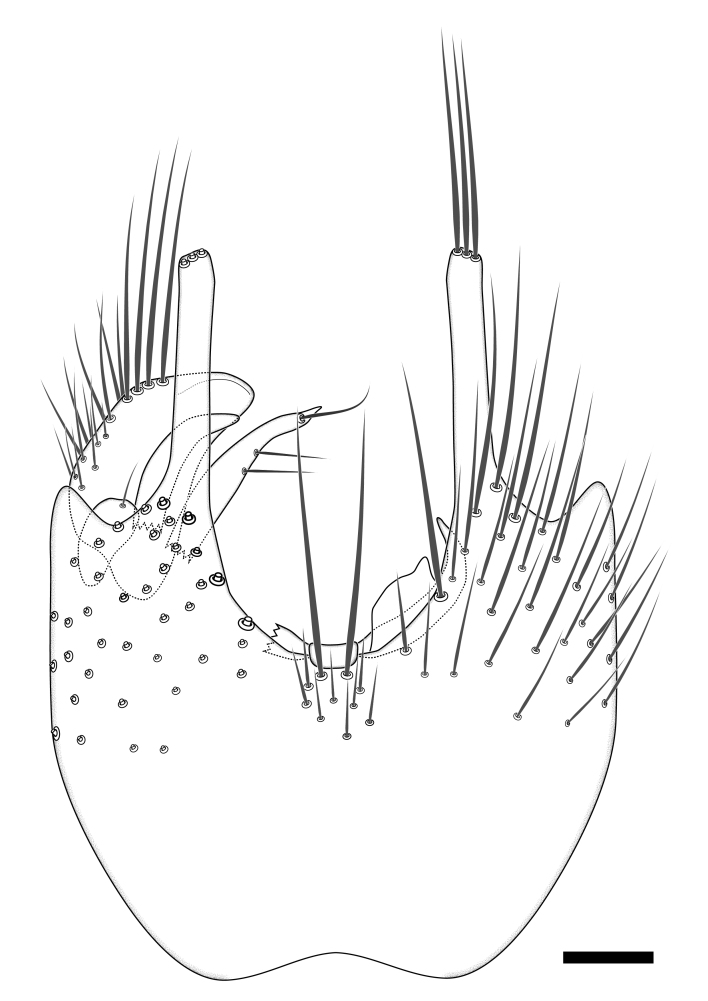
Male terminalia ventral view. Left gonocoxal setae and right gonostylus not drawn. Scale = 50 μm.

**Figure 12b.**
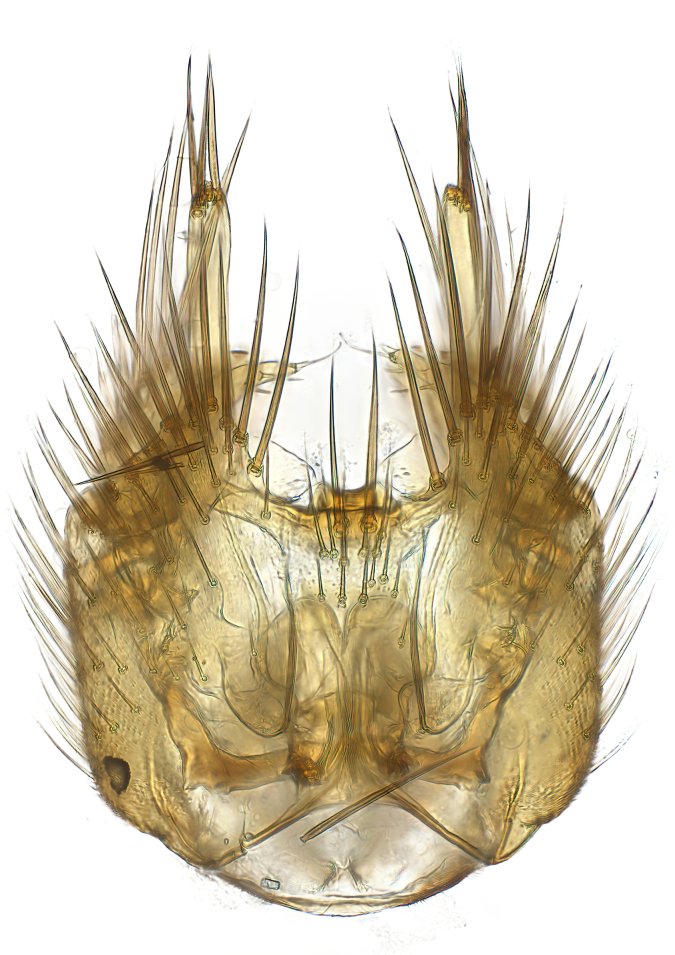
Male terminalia ventral view. Photo.

**Figure 12c.**
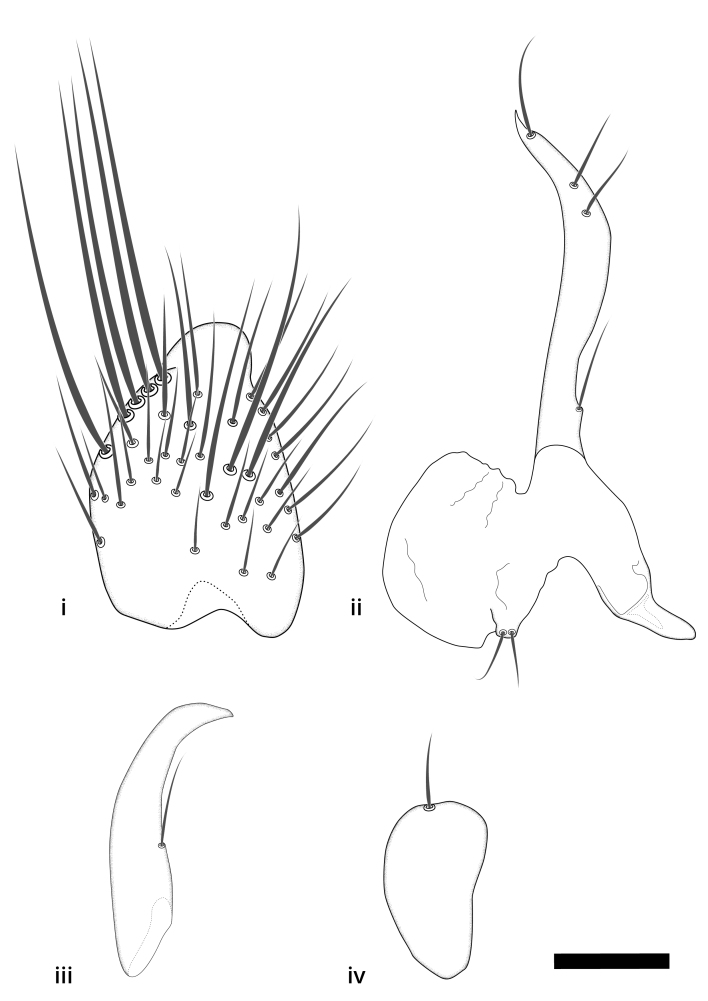
Male right gonostylus with: **i** dorsal branch, **ii** internal branch, **iii** medial branch and **iv** ventral branch separated. Scale = 50 μm.

**Figure 12d.**
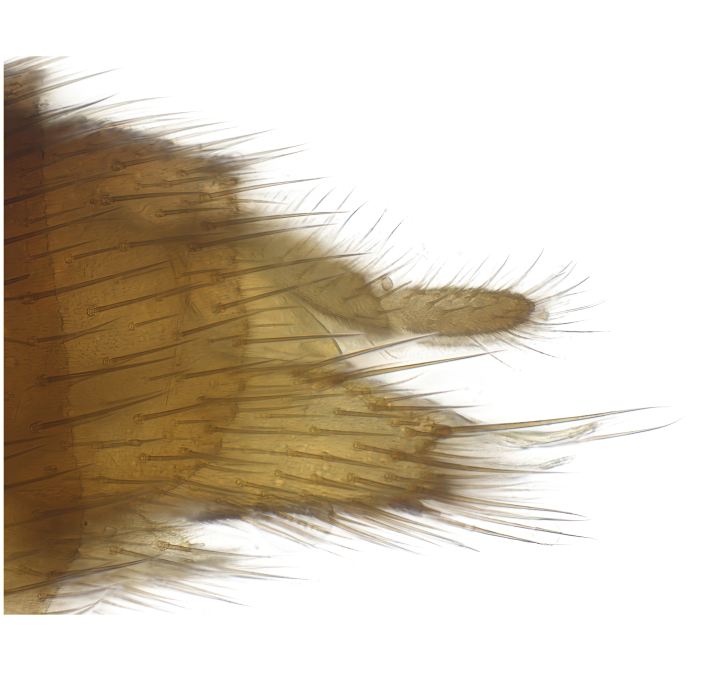
Female terminalia, lateral view. Photo.

**Figure 12e.**
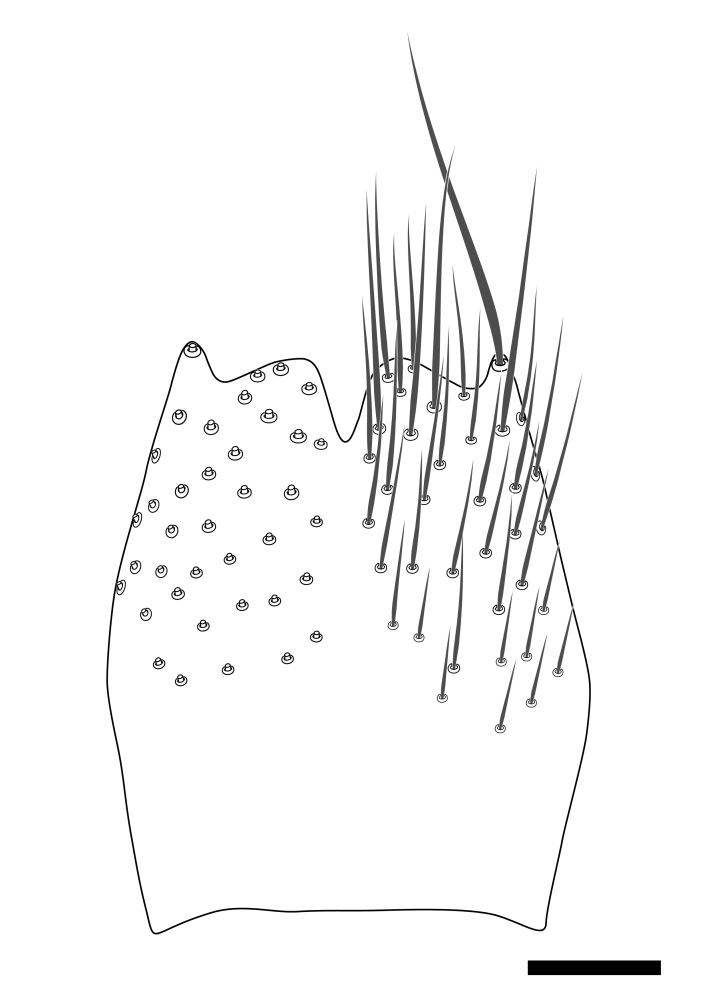
Female sternite VIII ventral view. Setae on left half not drawn. Scale = 50 μm.

**Figure 14a.**
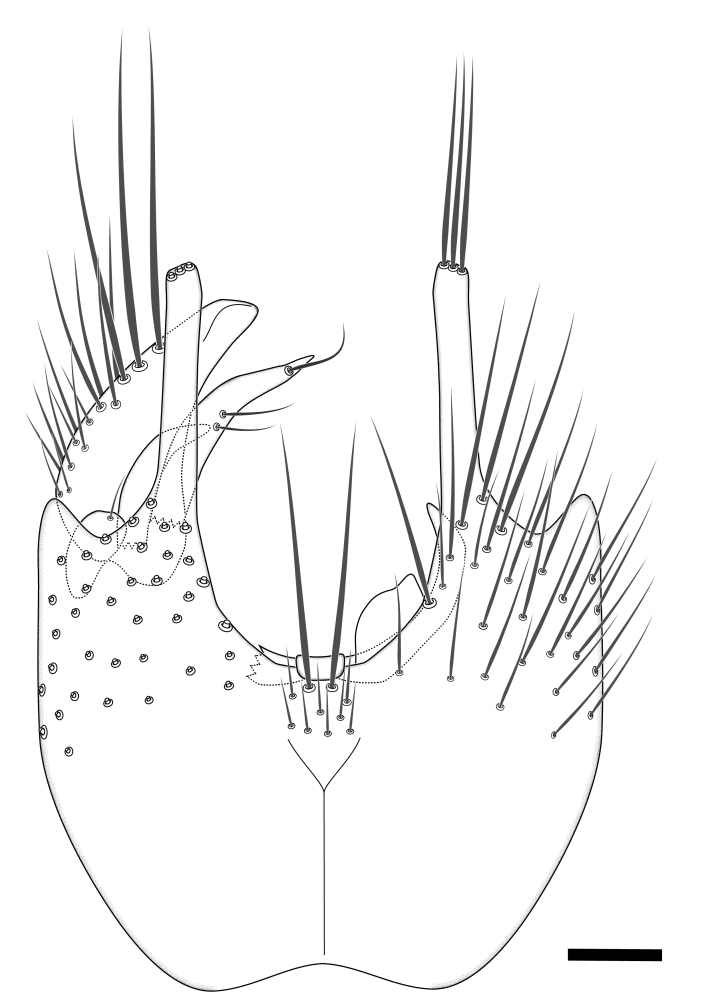
Ventral view. Right gonostylus and setae on left gonocoxite not drawn. Scale = 50 μm.

**Figure 14b.**
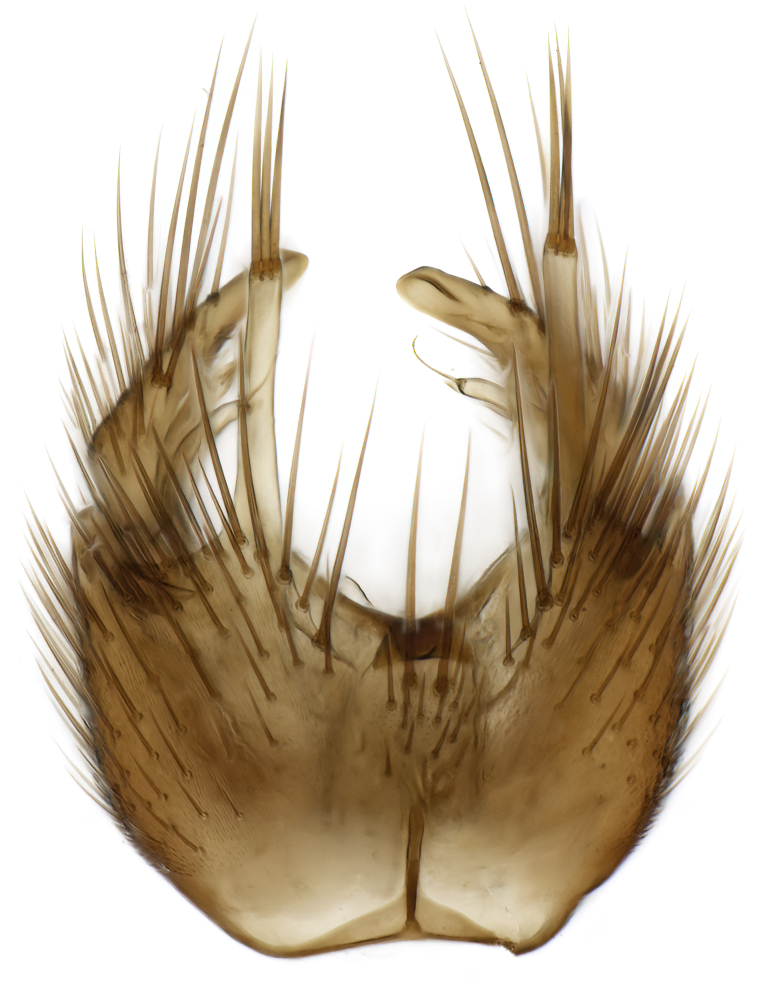
Terminalia ventral view. Photo.

**Figure 14c.**
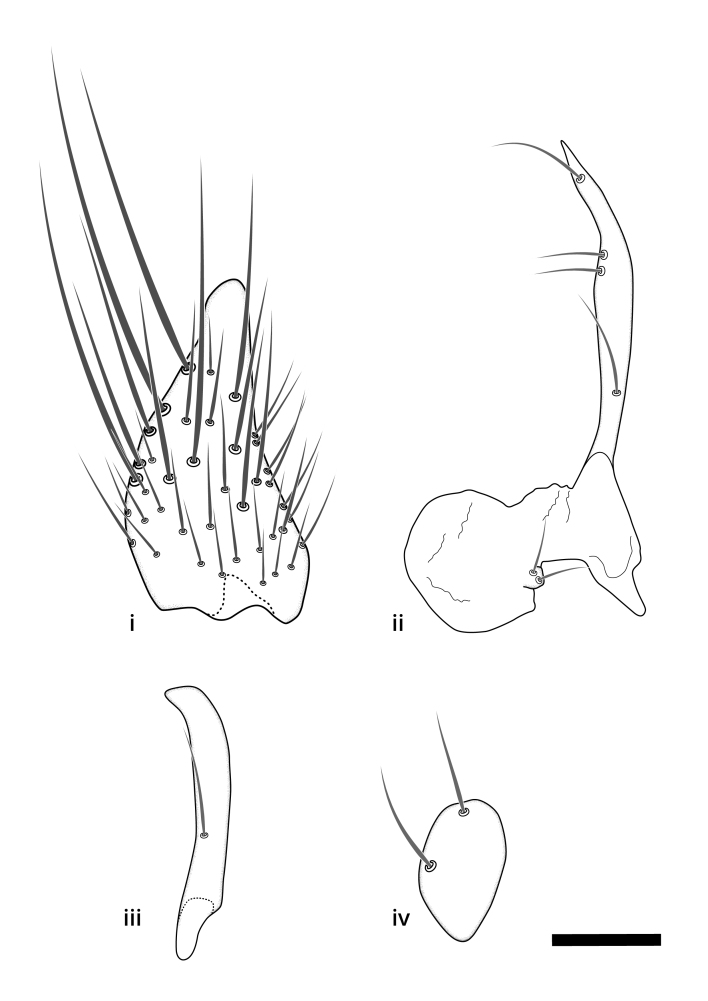
Right gonostylus with: **i** dorsal branch, **ii** internal branch, **iii** medial branch and **iv** ventral branch separated. Scale = 50 μm.

**Figure 16a.**
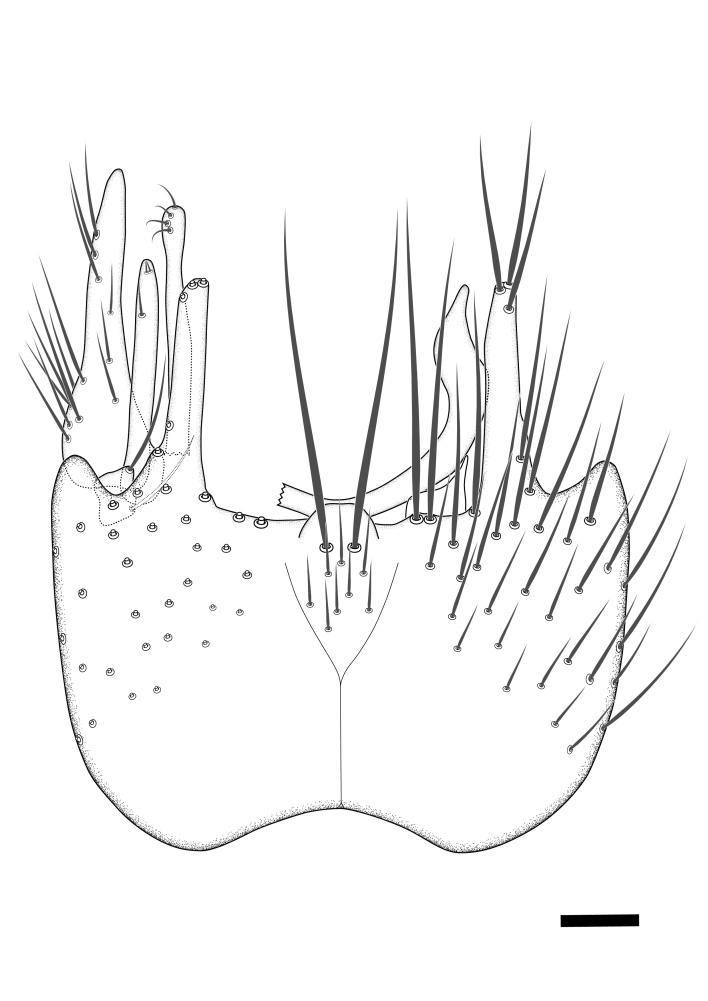
Ventral view. Right gonostylus and setae on left gonocoxite not drawn. Scale = 50 μm.

**Figure 16b.**
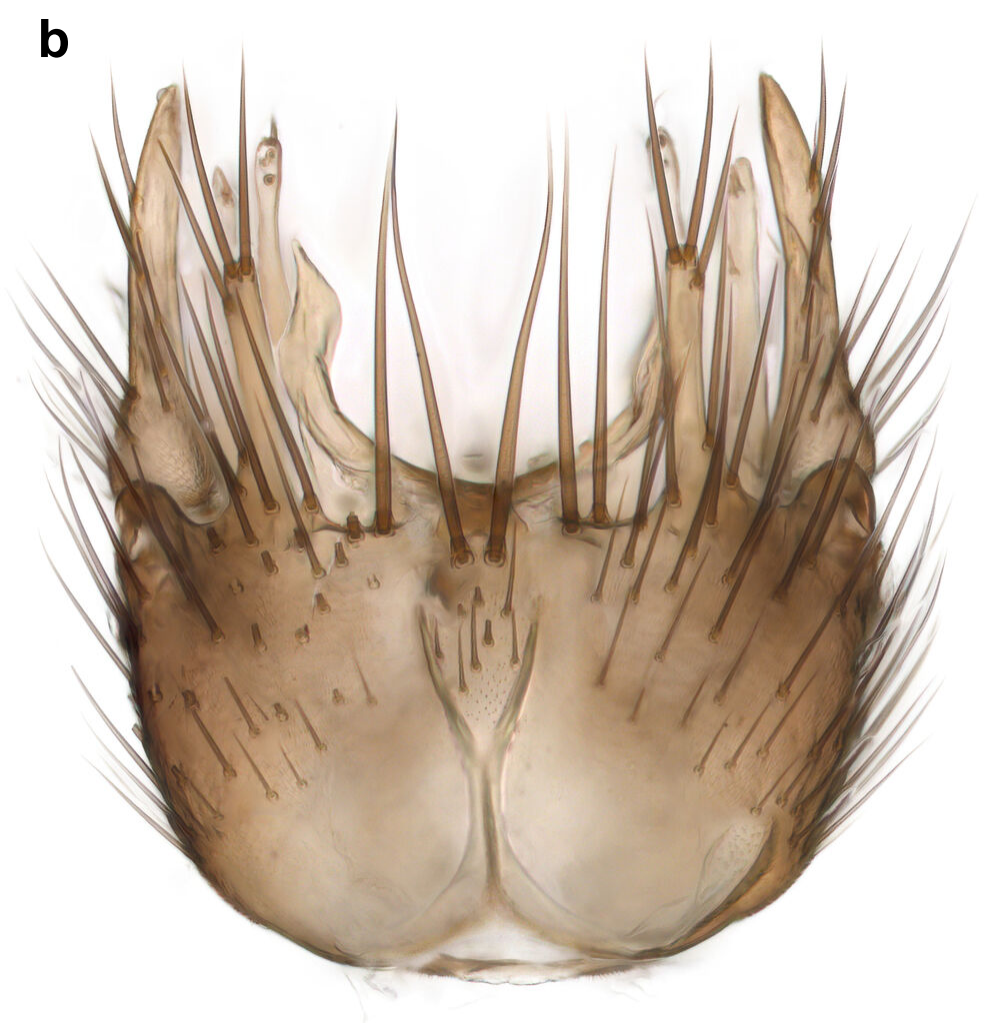
Ventral view. Photo.

**Figure 16c.**
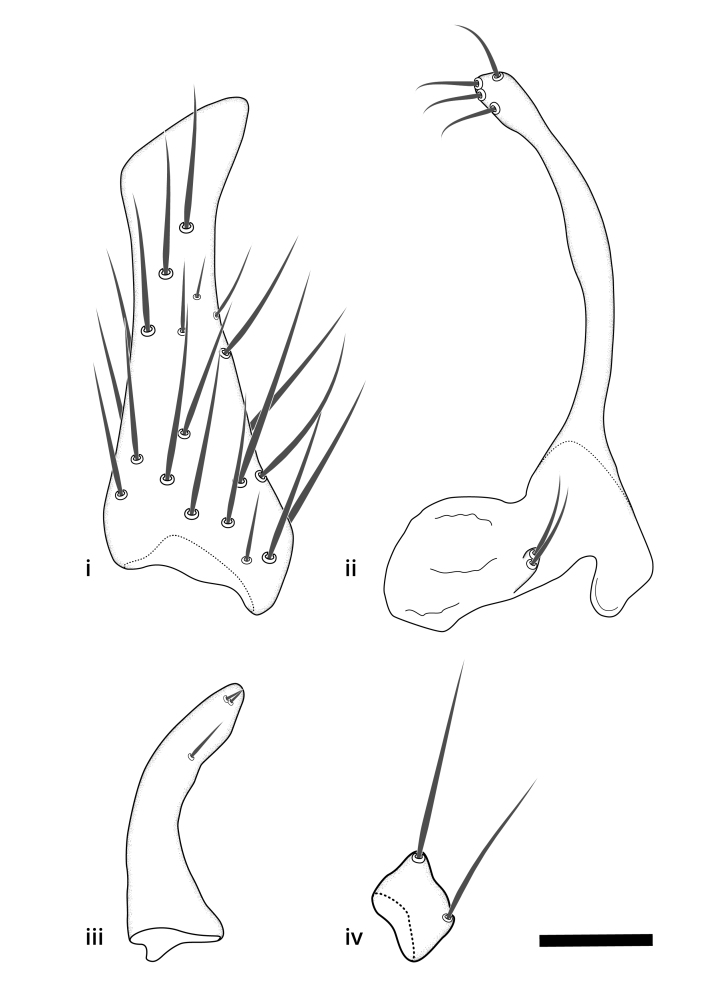
Right gonostylus with: **i** dorsal branch, **ii** internal branch, **iii** medial branch and **iv** ventral branch separated. Scale = 50 μm.

**Figure 17a.**
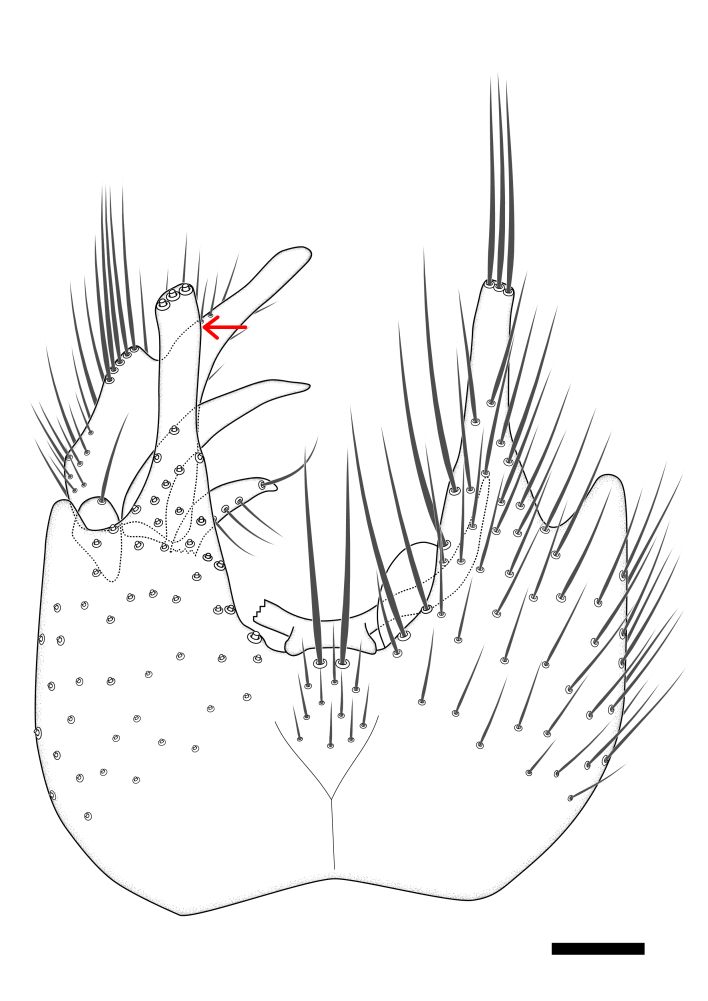
Male terminalia ventral view. Right gonostylus and setae on left gonocoxite not drawn. Red arrow indicates angled apico-internal margin of the gonocoxal lobe. Scale = 50 μm.

**Figure 17b.**
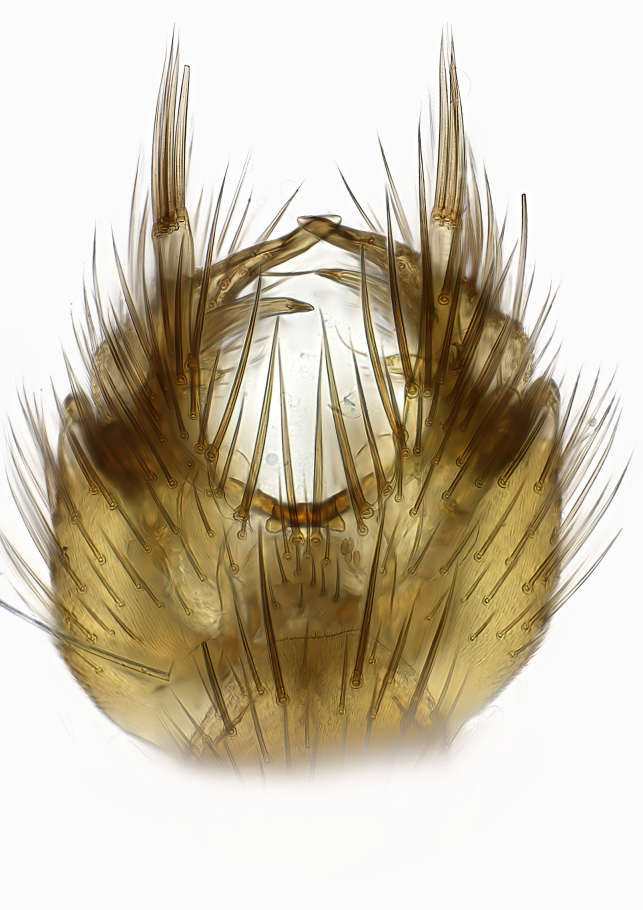
Male terminalia ventral view. Photo.

**Figure 17c.**
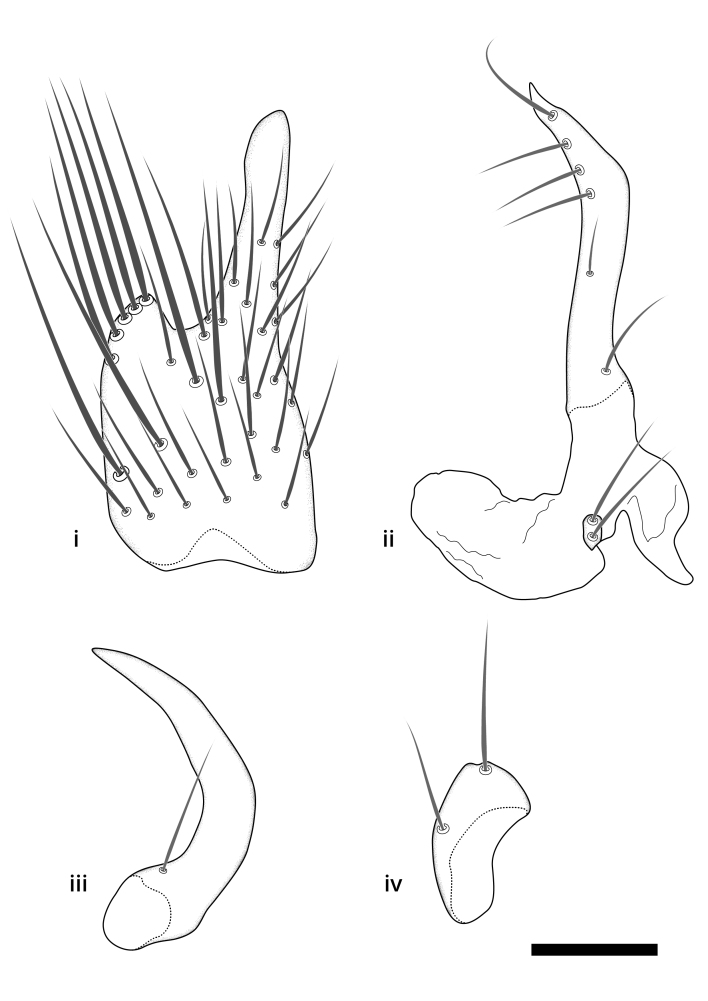
Male right gonostylus with: **i** dorsal branch, **ii** internal branch, **iii** medial branch and **iv** ventral branch separated. Scale = 50 μm.

**Figure 17d.**
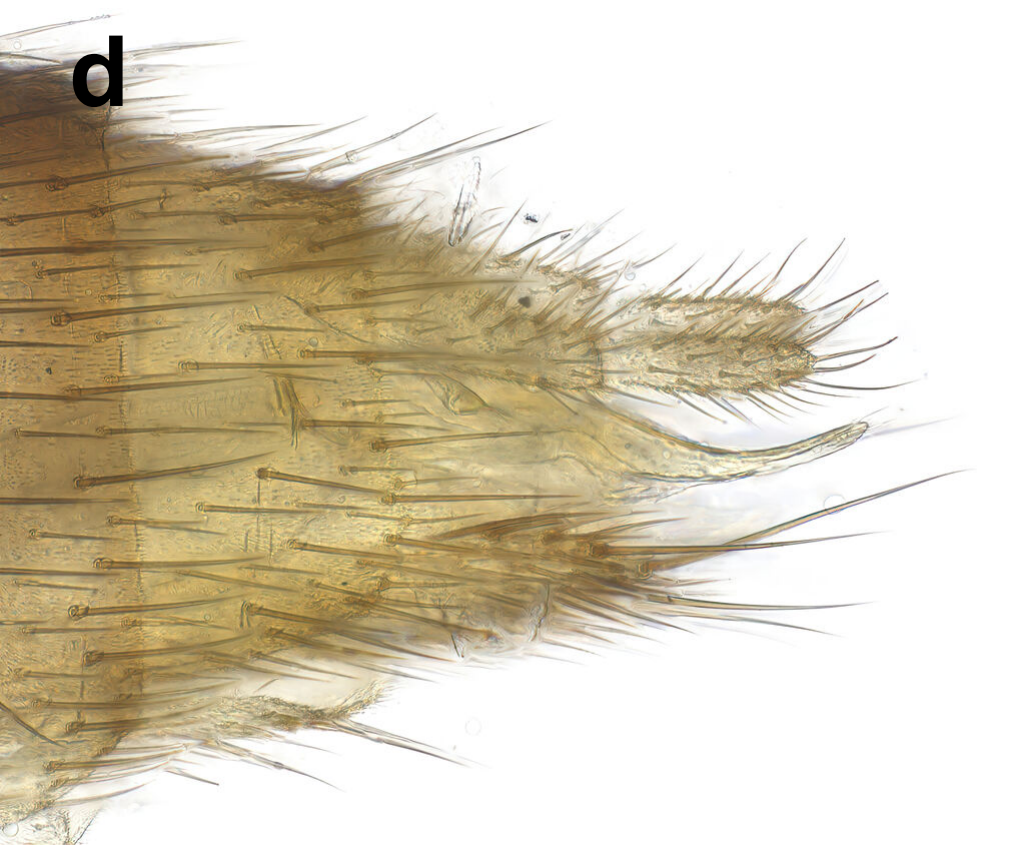
Female terminalia lateral view. Photo.

**Figure 17e.**
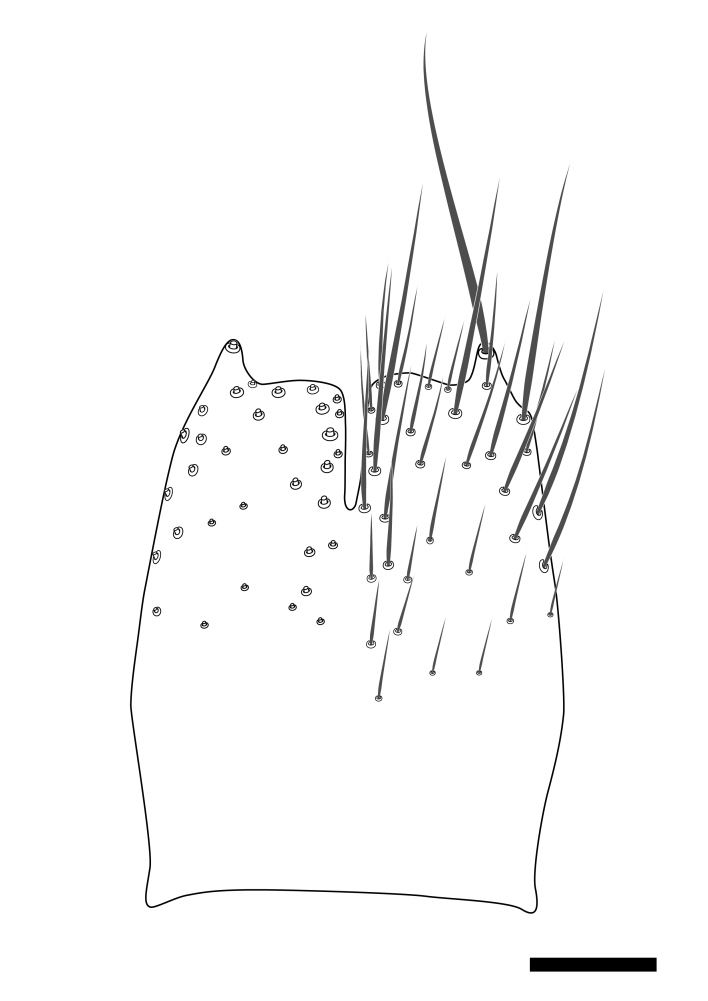
Female sternite VIII ventral view. Setae on left half not drawn. Scale = 50 μm.

**Figure 17f.**
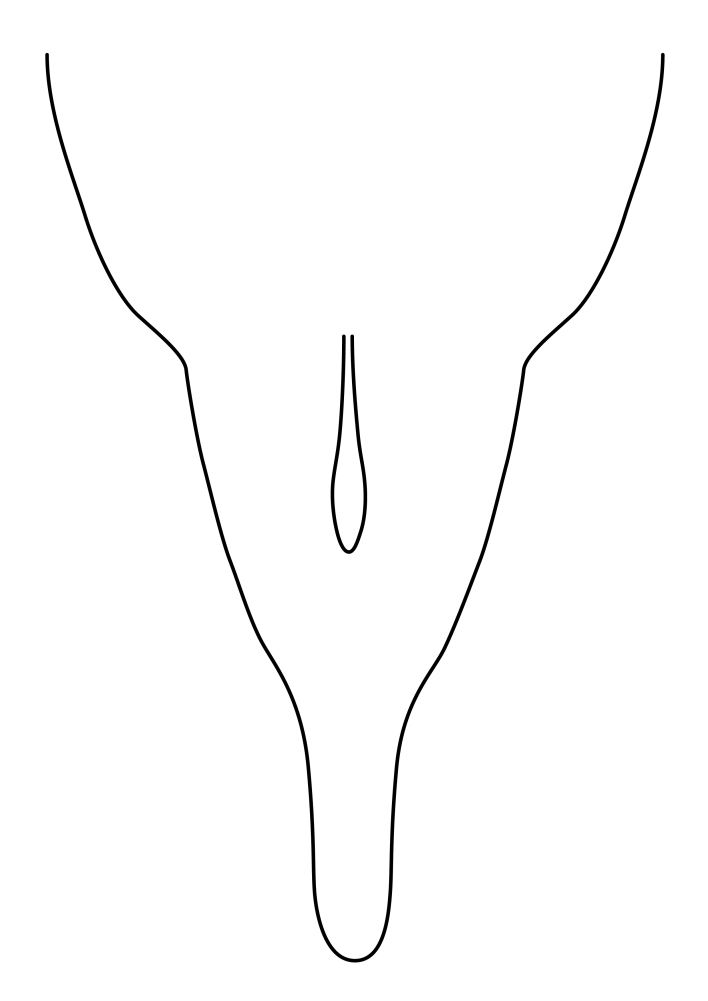
Female gonapophysis IX and spermathecal eminence in ventral view.

**Figure 18a.**
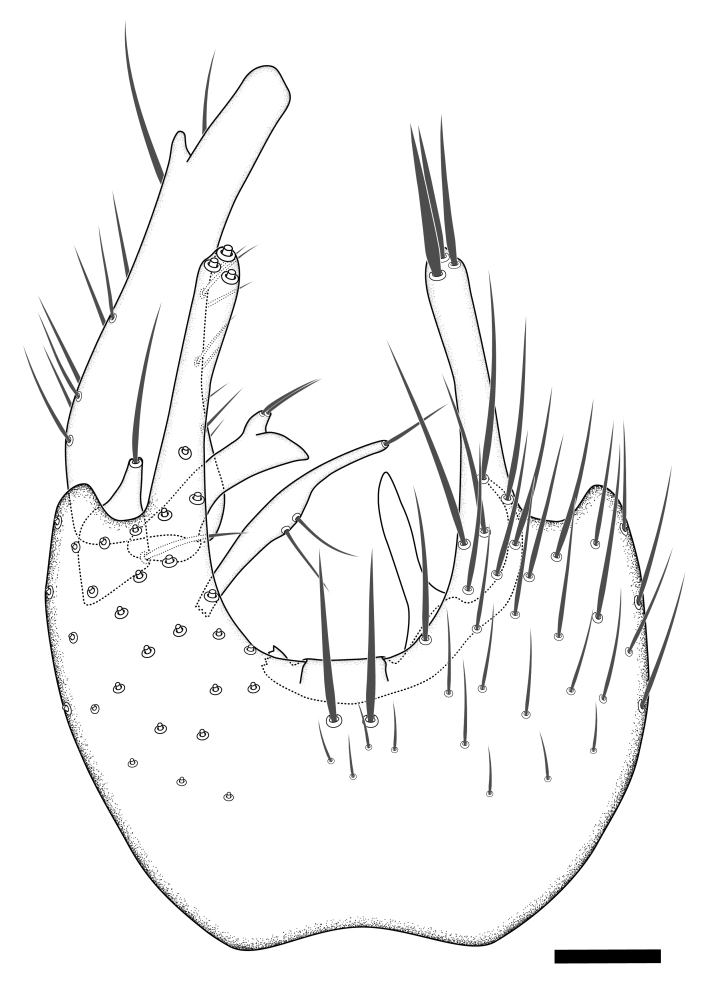
Ventral view. Right gonostylus and setae on left gonocoxite not drawn. Scale = 50 μm.

**Figure 18b.**
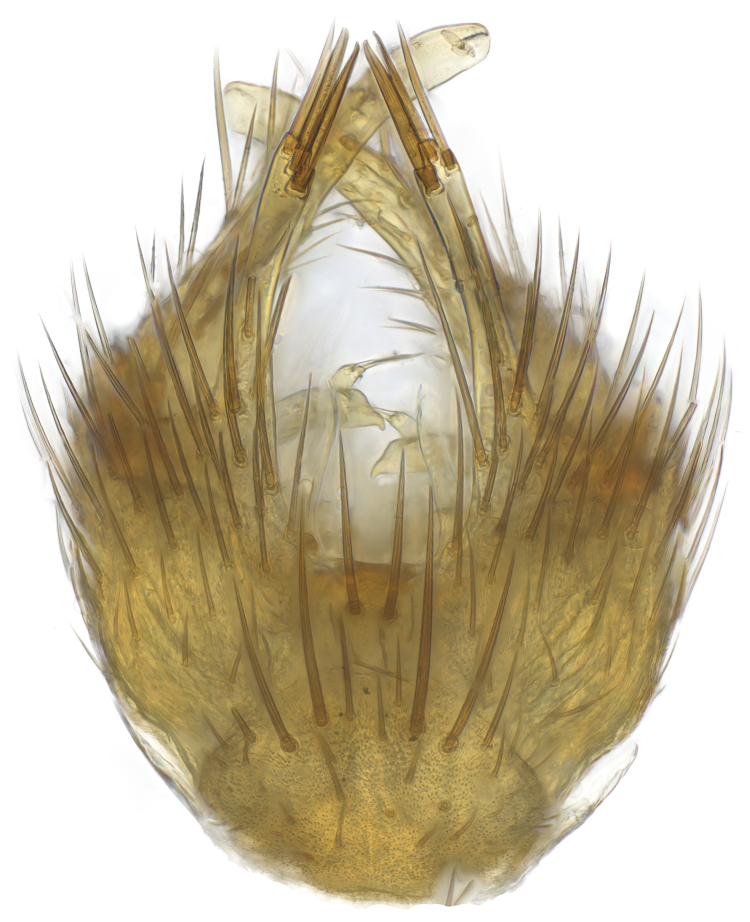
Ventral view. Photo.

**Figure 18c.**
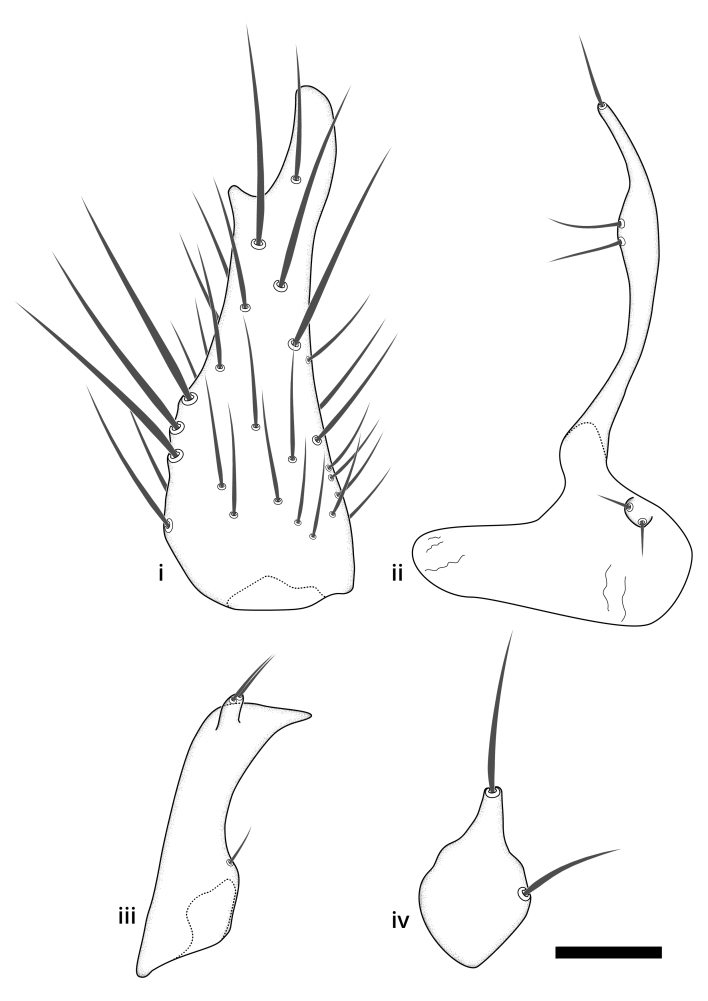
Right gonostylus with: **i** dorsal branch, **ii** internal branch, **iii** medial branch and **iv** ventral branch separated. Scale = 50 μm.

**Figure 20a.**
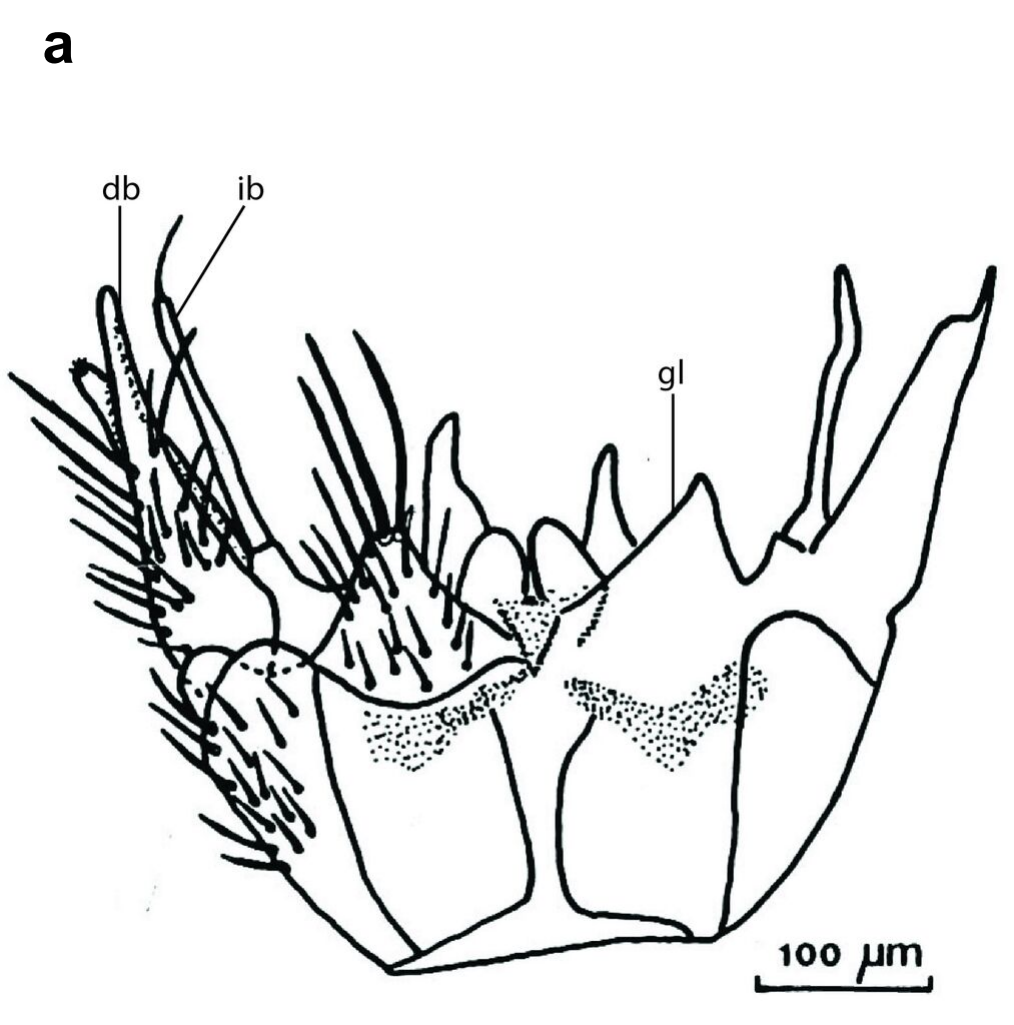
Ventral view. Reprinted with permission from Chandler and Ribeiro (1995)

**Figure 20b.**
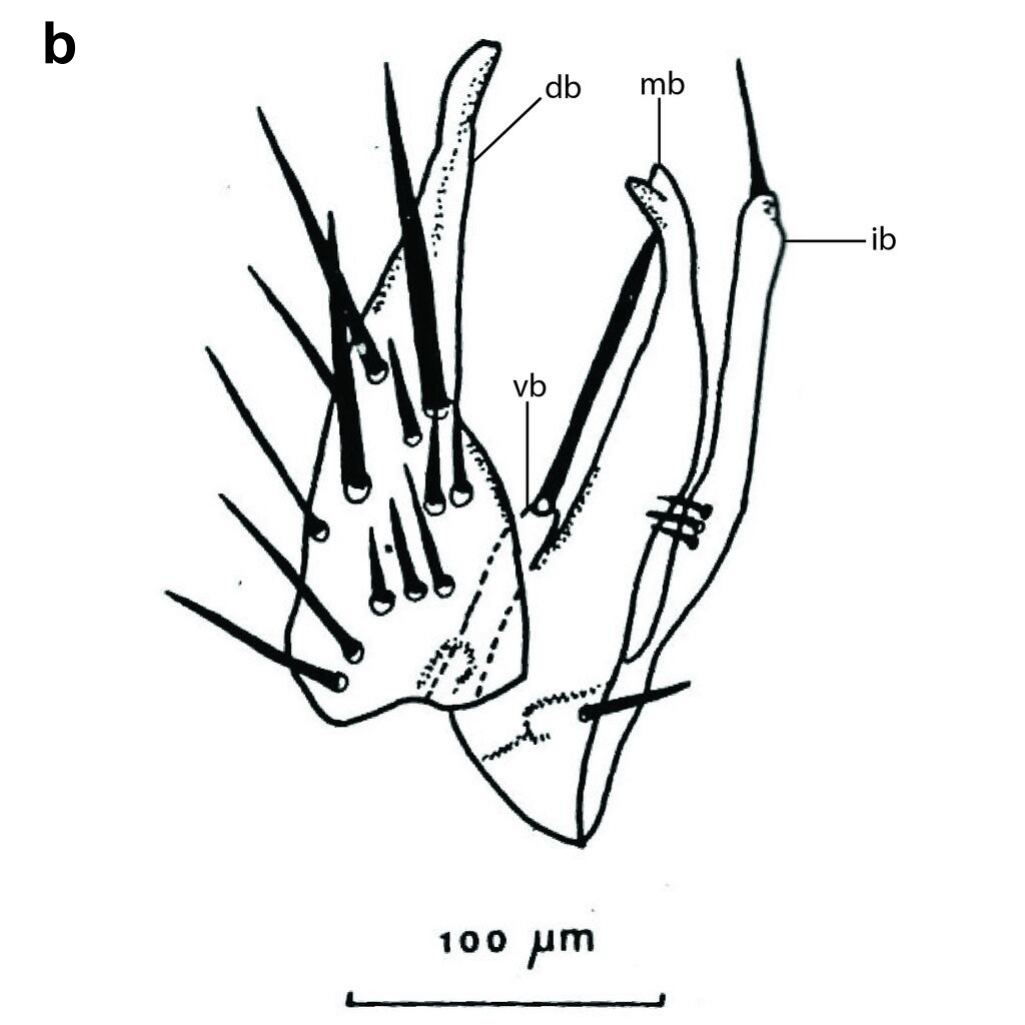
Left gonostylus in lateral view. Reprinted with permission from Chandler and Ribeiro (1995)

**Figure 20c.**
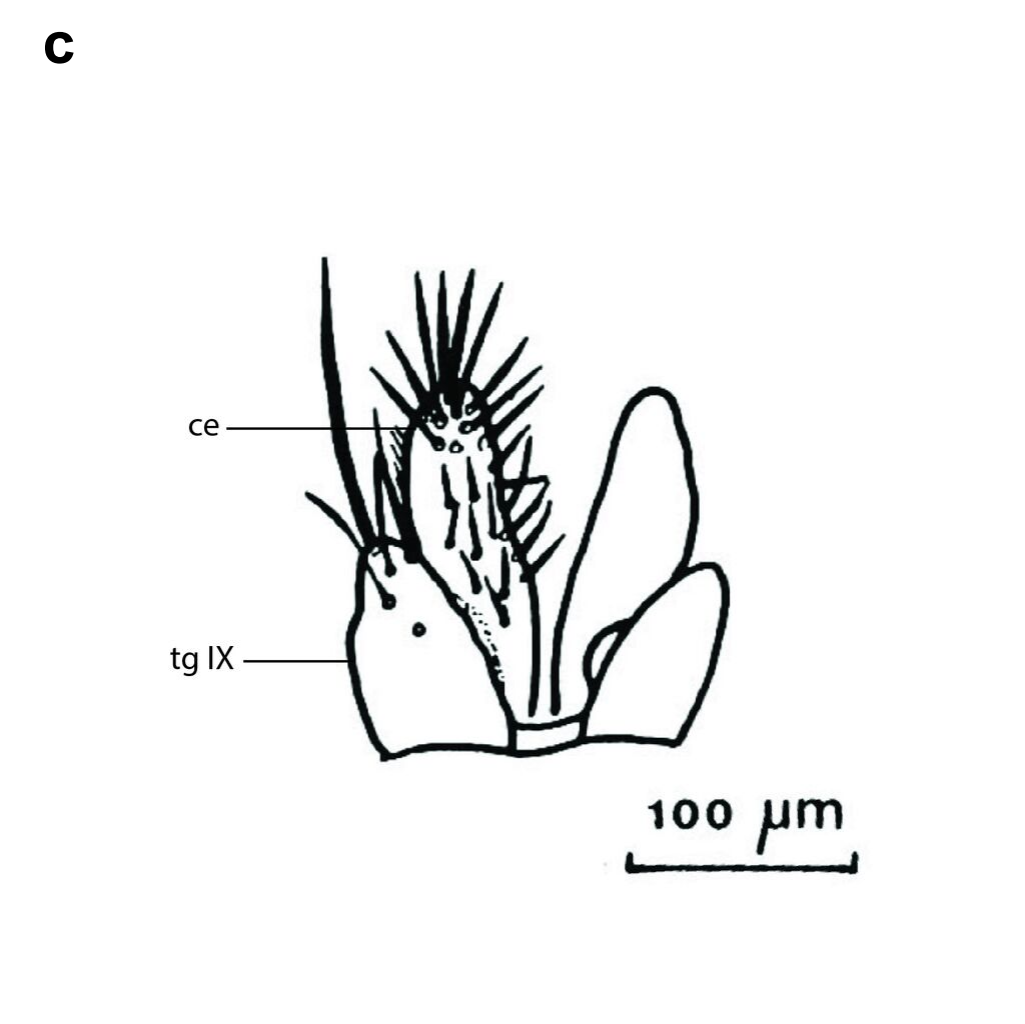
Tergite IX and cerci. Reprinted with permission from Chandler and Ribeiro (1995)

**Figure 20d.**
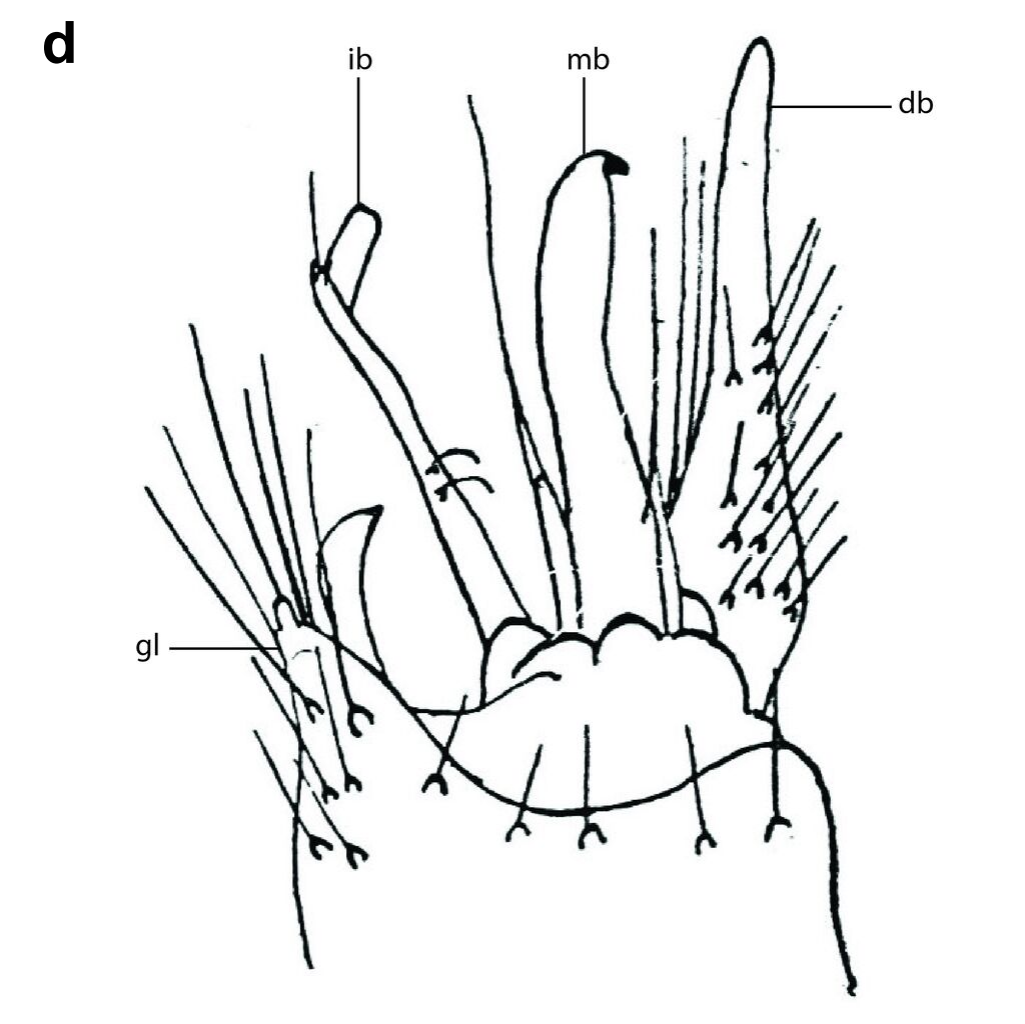
Lateral view. Reprinted from Storå (1941)

**Figure 21a.**
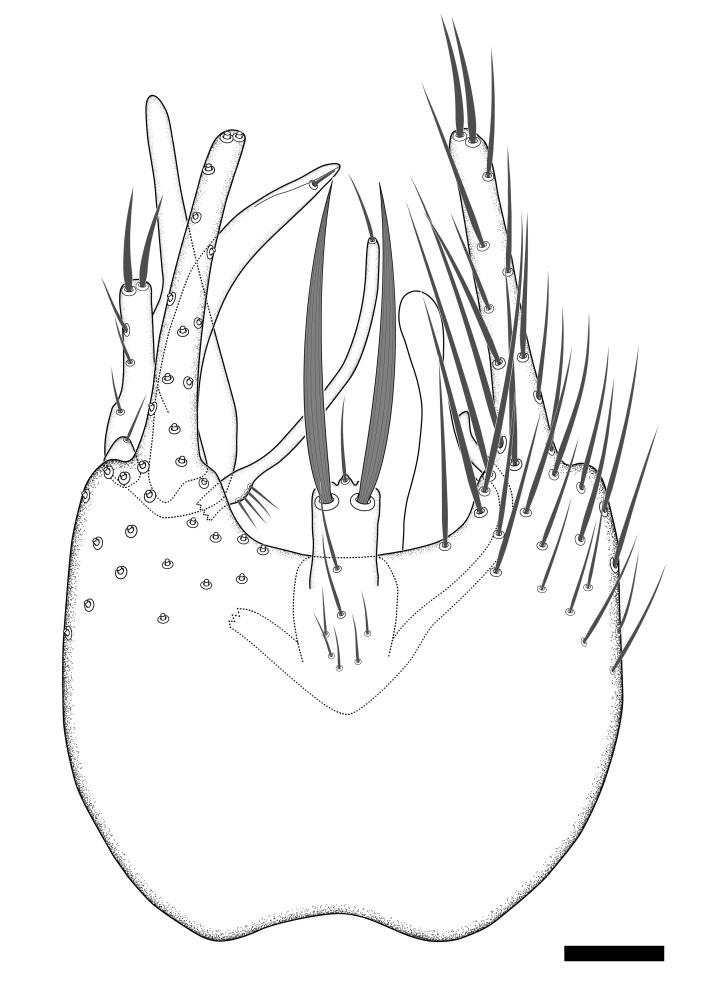
Ventral view. Left gonocoxal setae and right gonostylus not drawn. Scale = 50 μm.

**Figure 21b.**
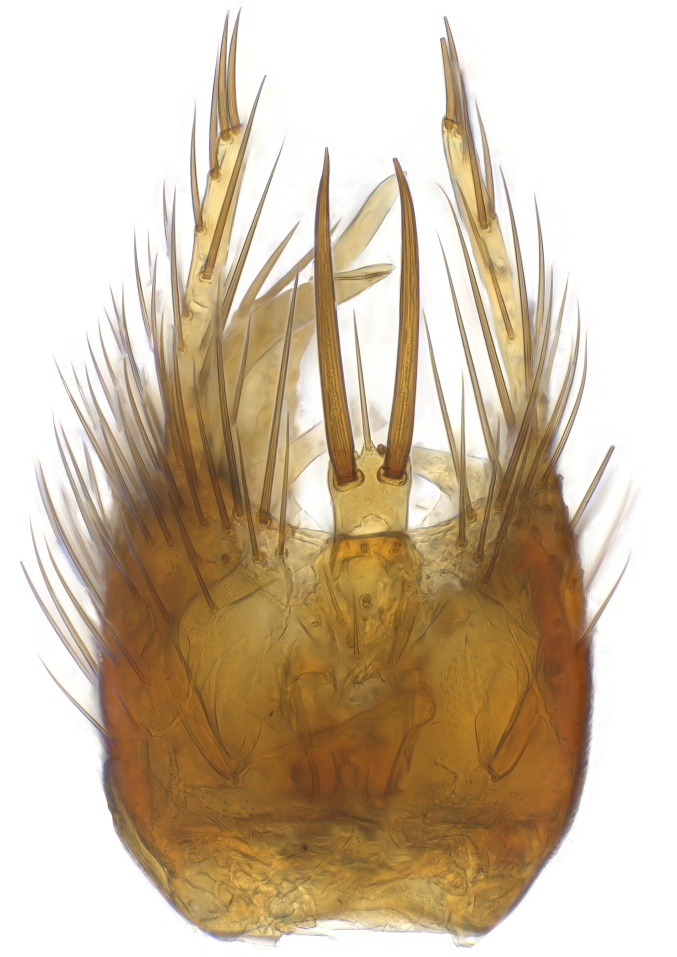
Ventral view. Photo.

**Figure 21c.**
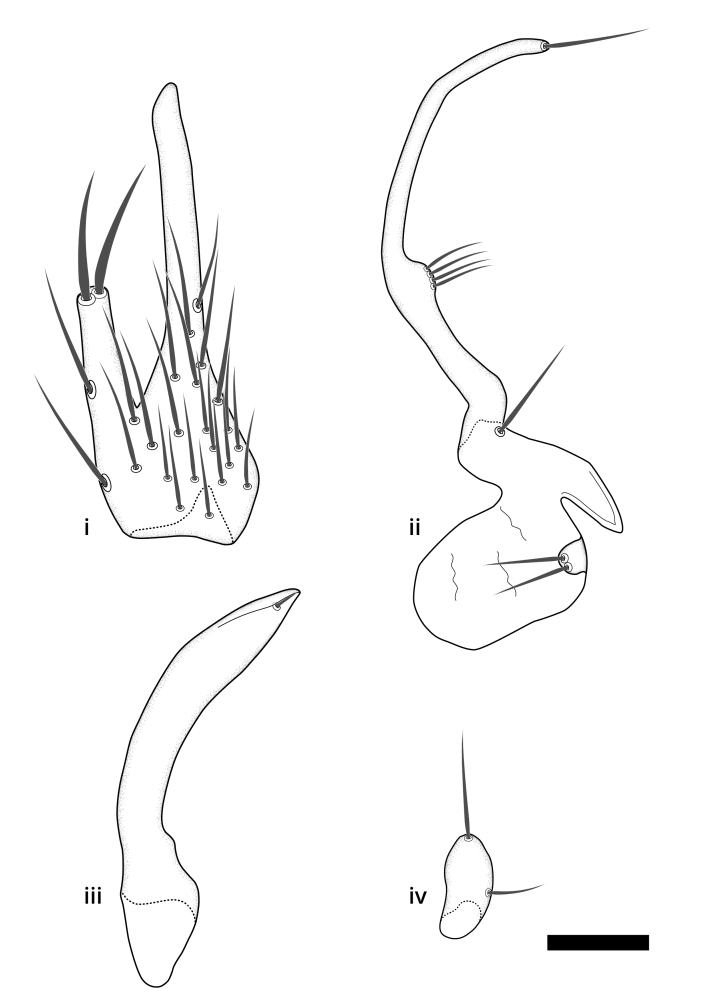
Right gonostylus with: **i** dorsal branch, **ii** internal branch, **iii** medial branch and **iv** ventral branch separated. Scale = 50 μm.

**Figure 22a.**
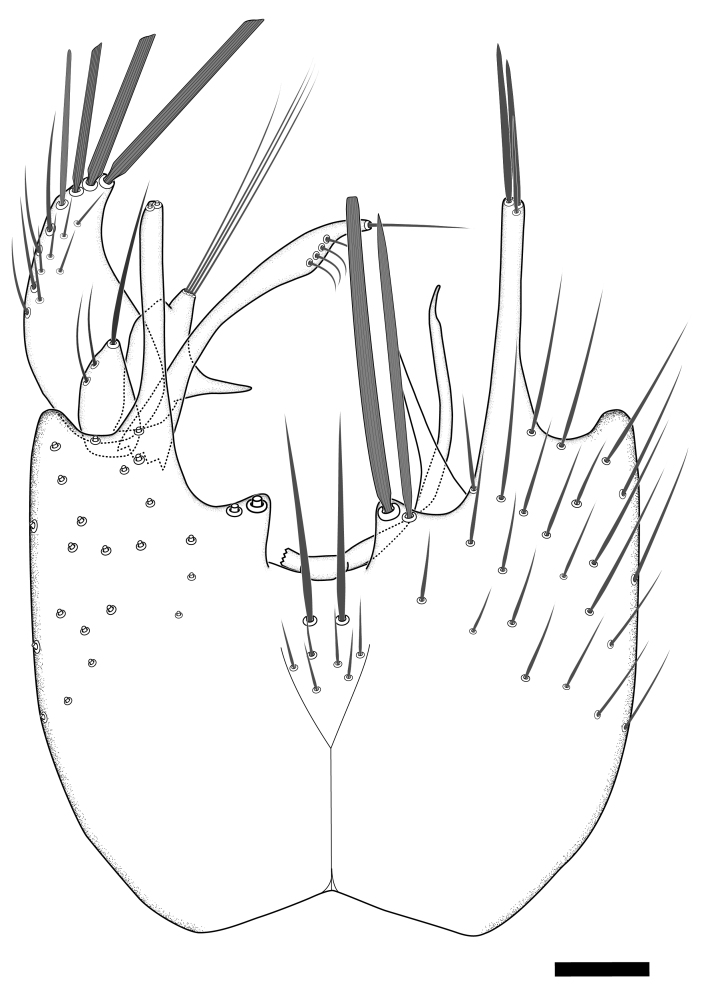
Ventral view. Left gonocoxal setae and right gonostylus not drawn. Scale = 50 μm.

**Figure 22b.**
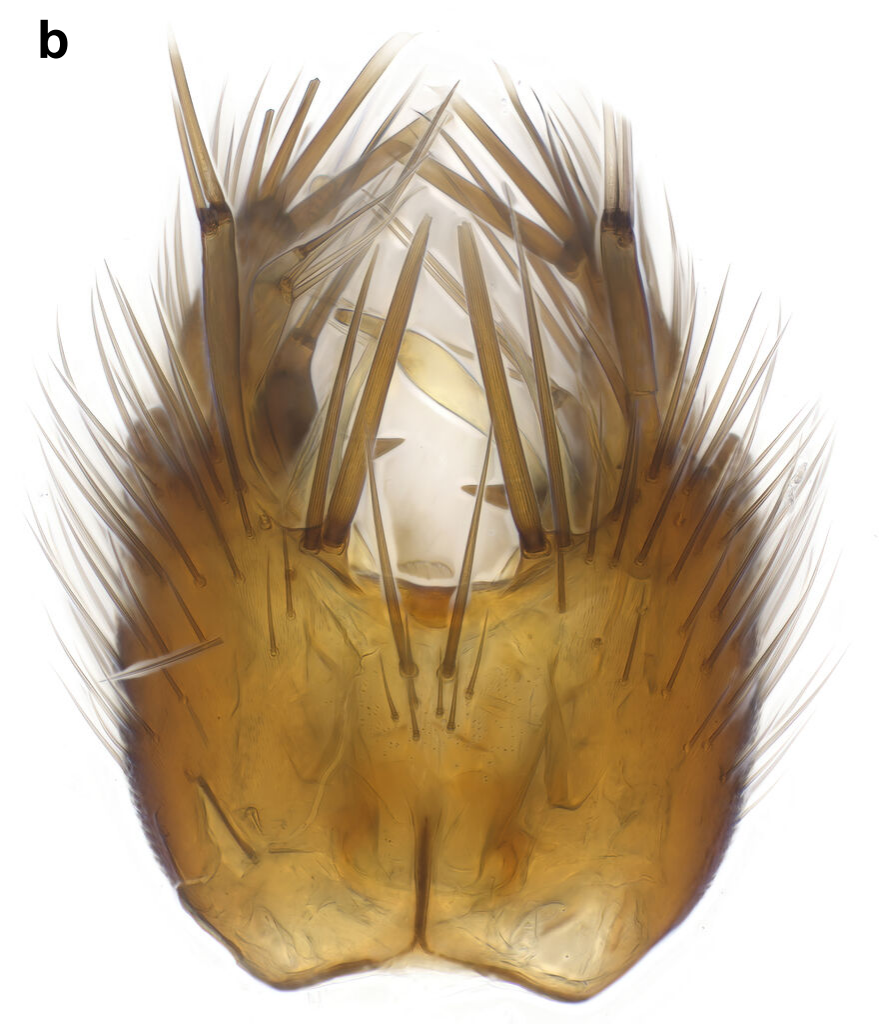
Ventral view. Photo.

**Figure 22c.**
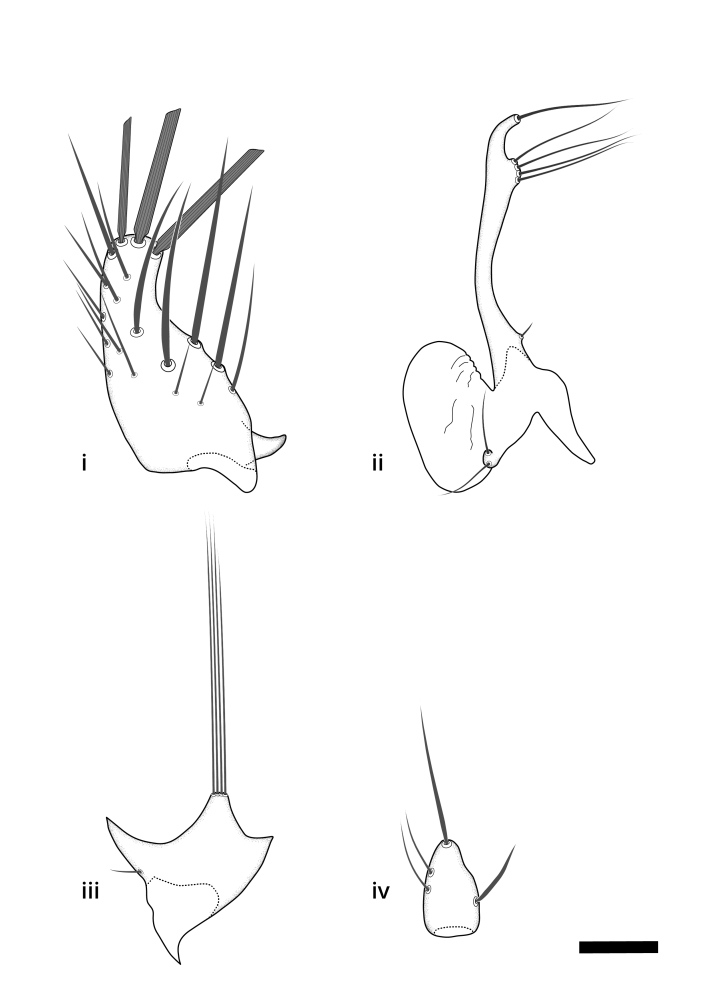
Right gonostylus with: **i** dorsal branch, **ii** internal branch, **iii** medial branch and **iv** ventral branch separated. Scale = 50 μm.

**Figure 23a.**
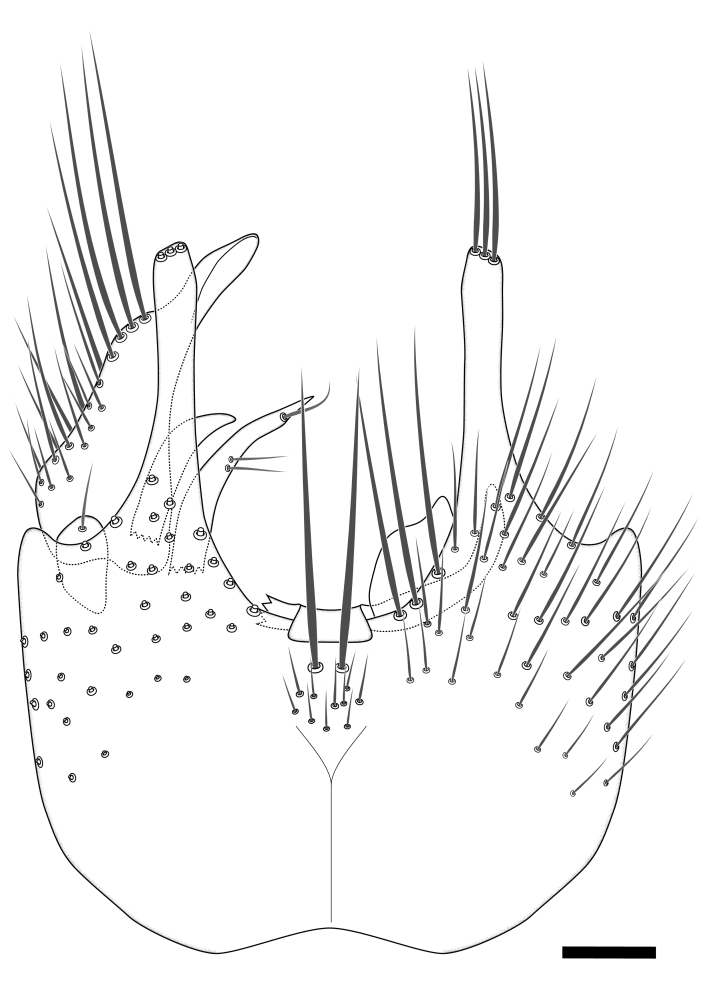
Male terminalia, ventral view.

**Figure 23b.**
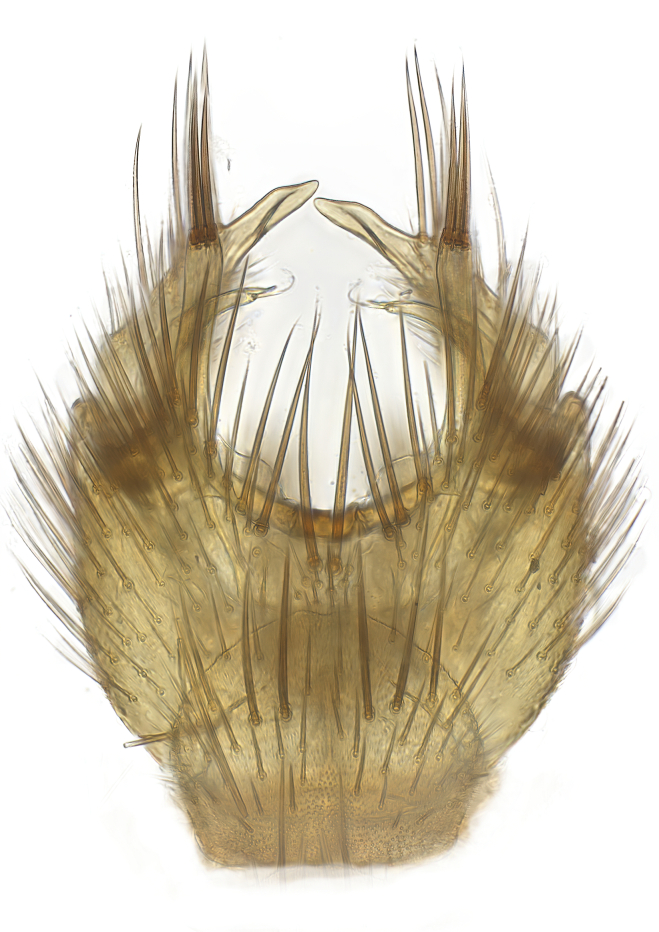
Male terminalia, ventral view. Right gonostylus and setae on left gonocoxite not drawn. Scale = 50 μm.

**Figure 23c.**
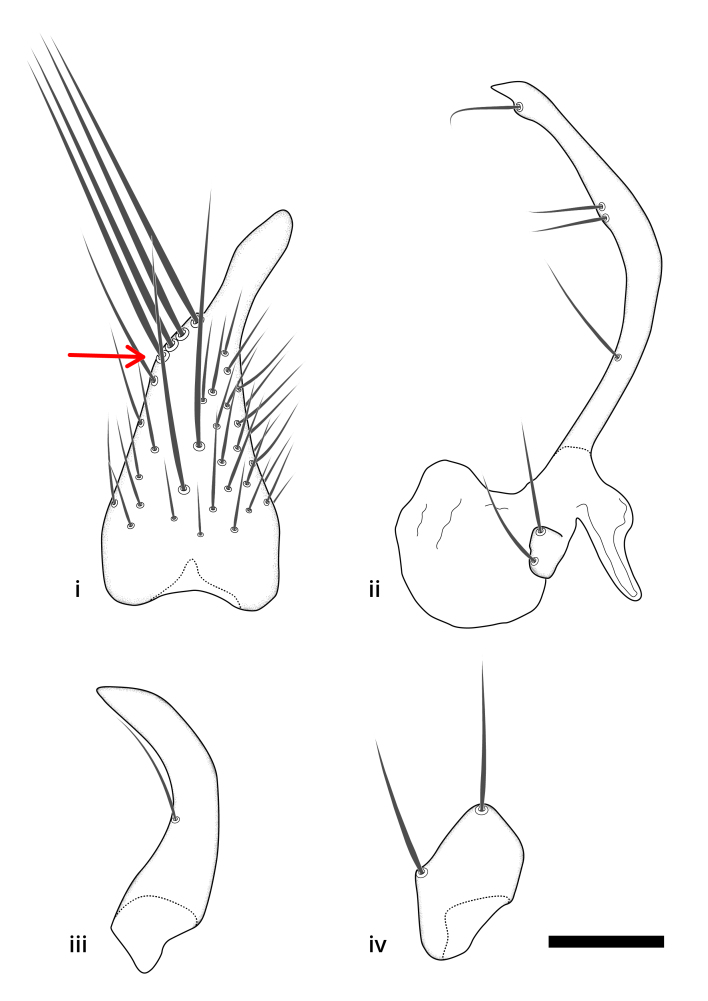
Male gonostylus with: **i** dorsal branch, **ii** internal branch, **iii** medial branch and **iv** ventral branch separated. Red arrow indicates angled external margin of the dorsal branch. Scale = 50 μm.

**Figure 23d.**
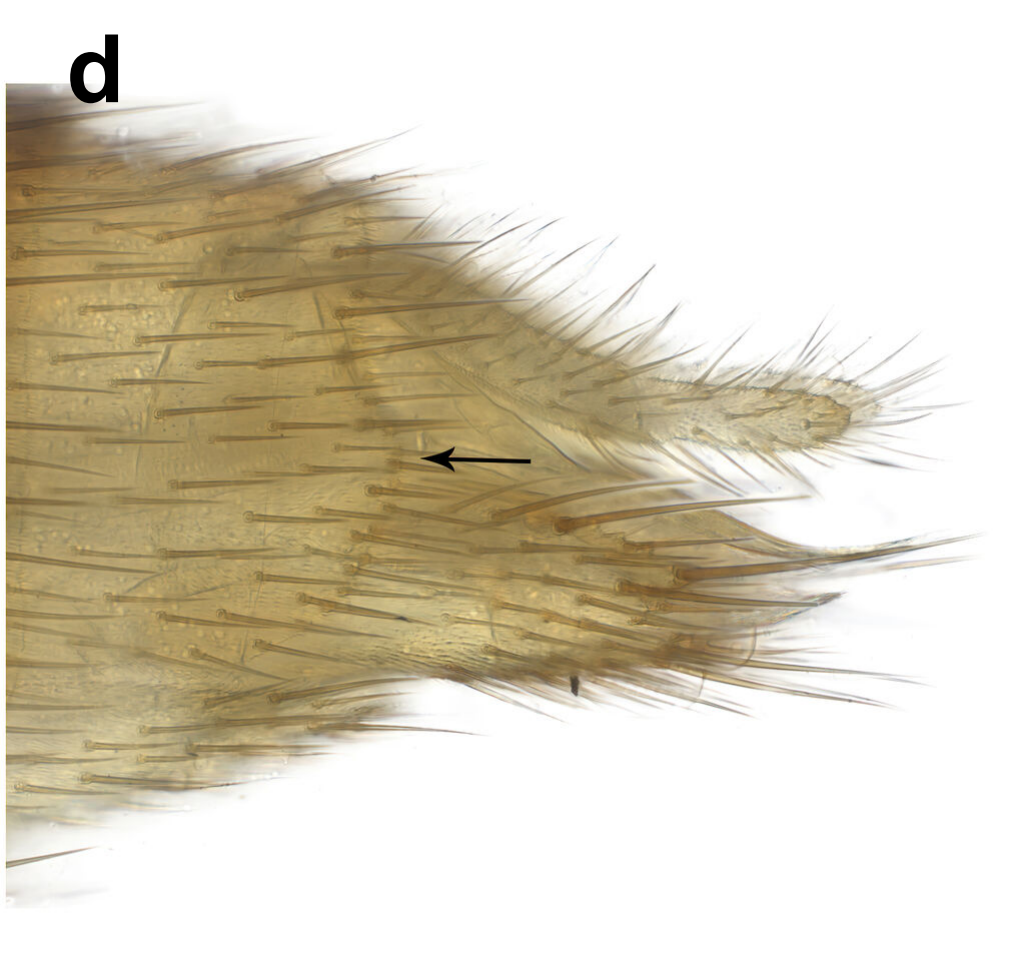
Female terminalia, lateral view. Photo.

**Figure 23e.**
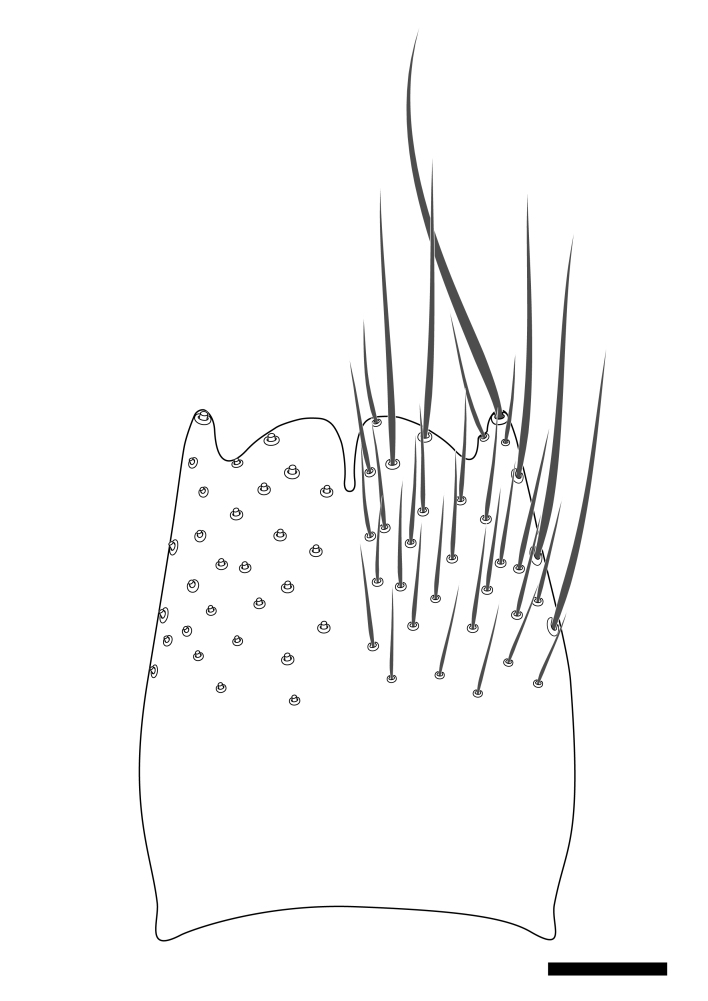
Female sternite VIII ventral view. Setae on left half not drawn. Scale = 50 μm.

**Figure 24a.**
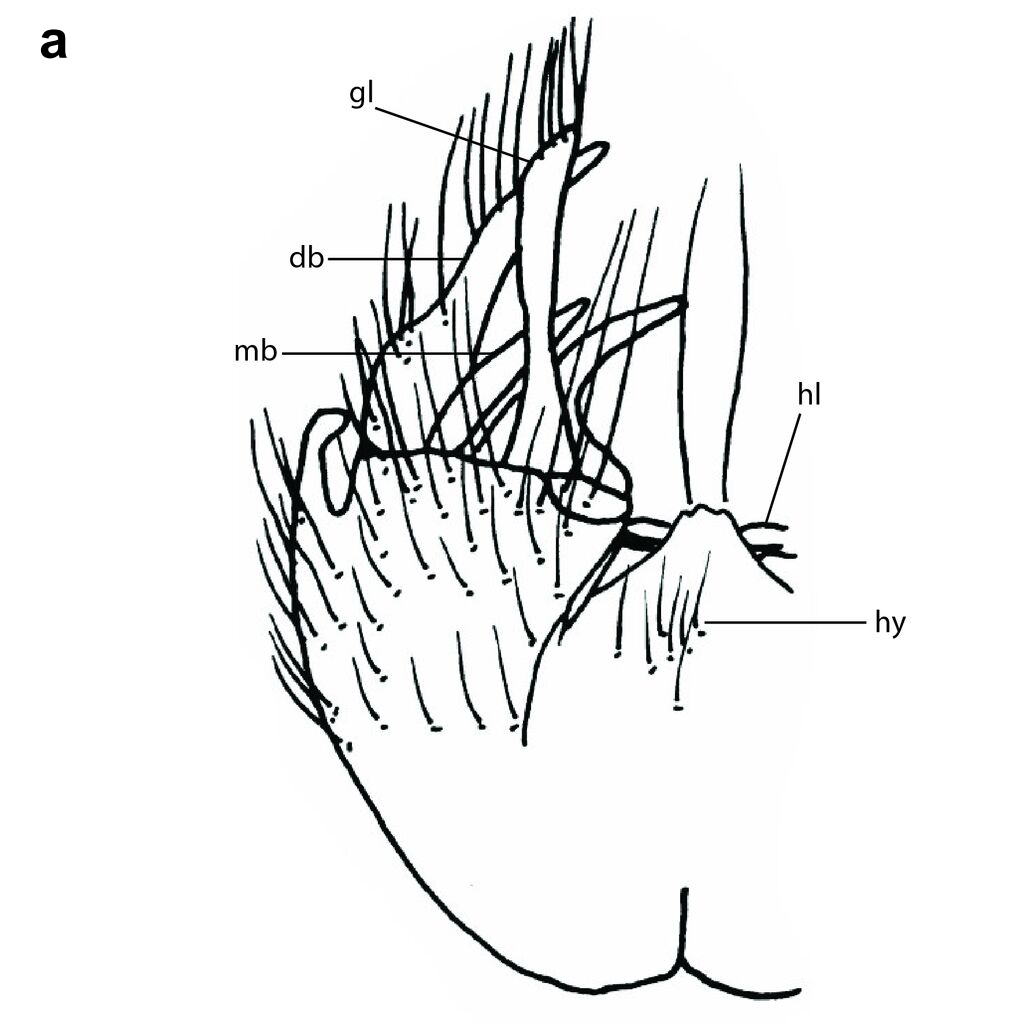
Ventral view.

**Figure 24b.**
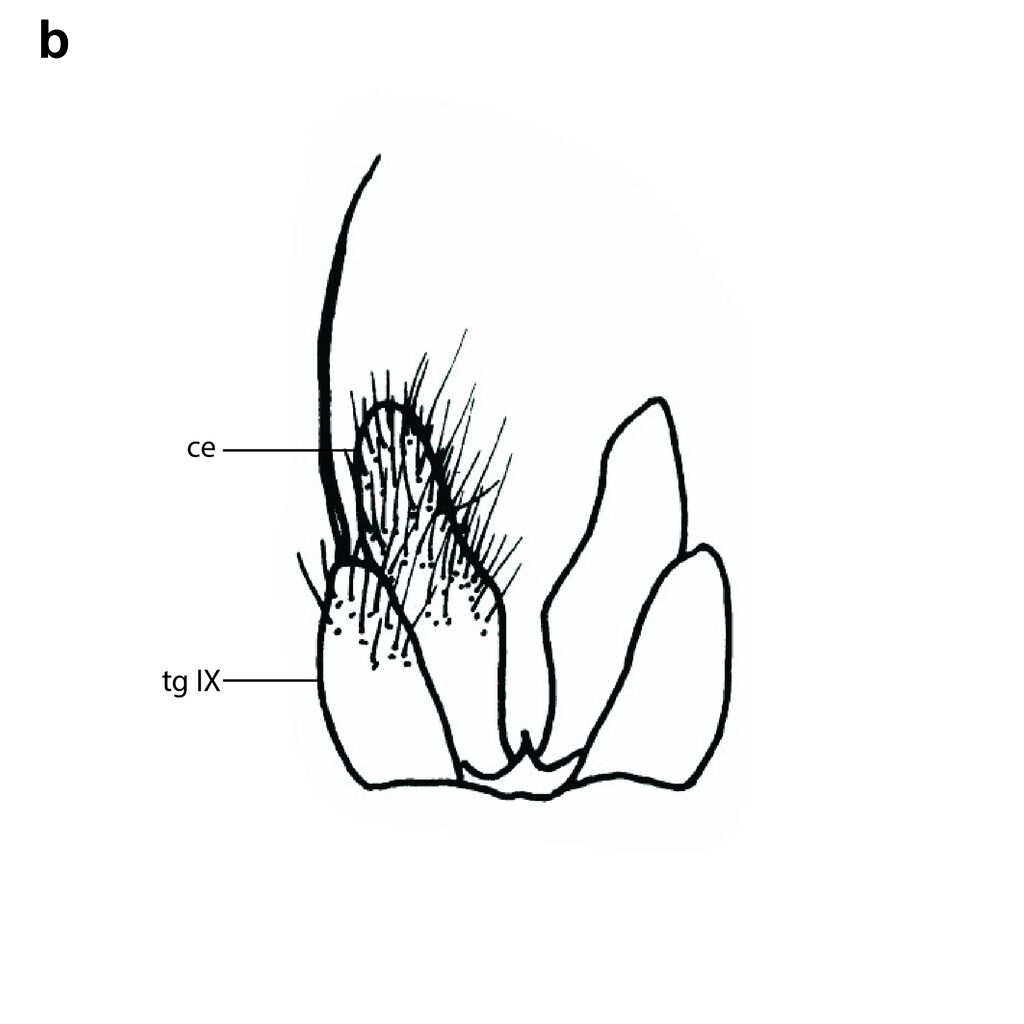
Tergite IX and cerci.

**Figure 25a.**
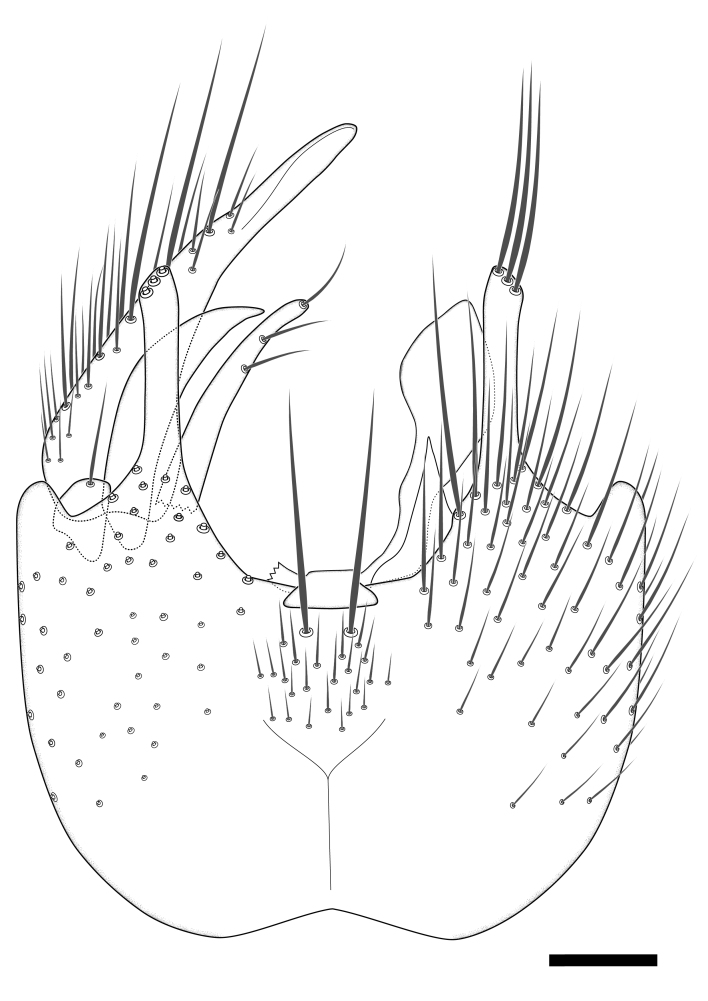
Ventral view. Right gonostylus and setae on left gonocoxite not drawn. Scale = 50 μm.

**Figure 25b.**
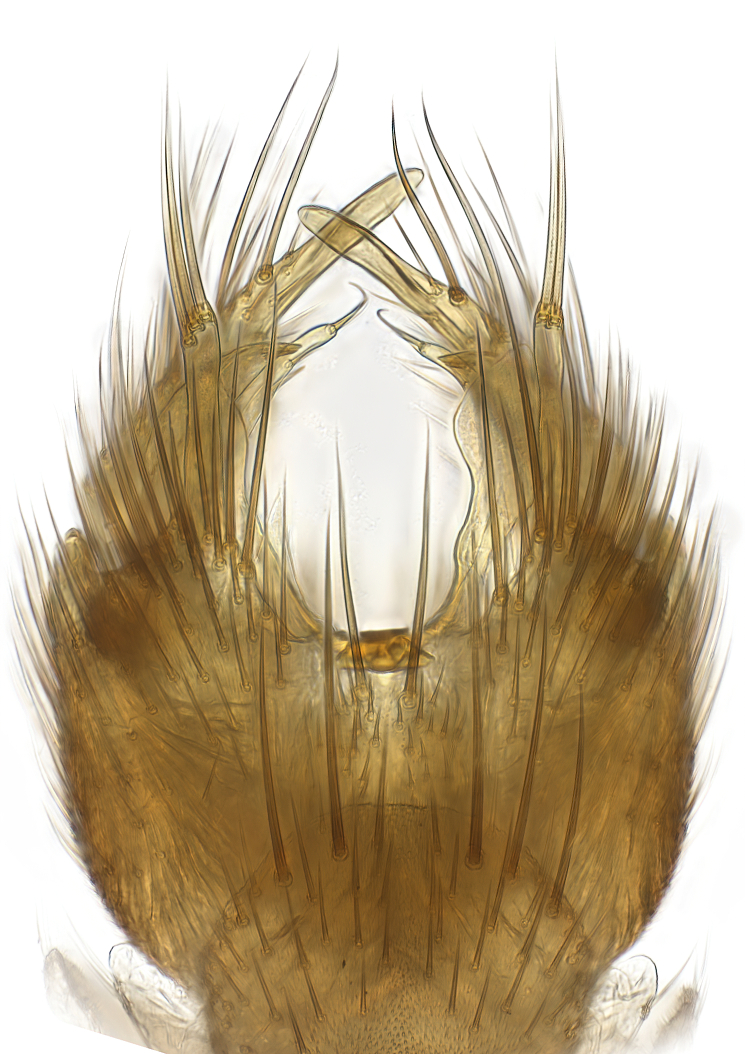
Ventral view. Photo.

**Figure 25c.**
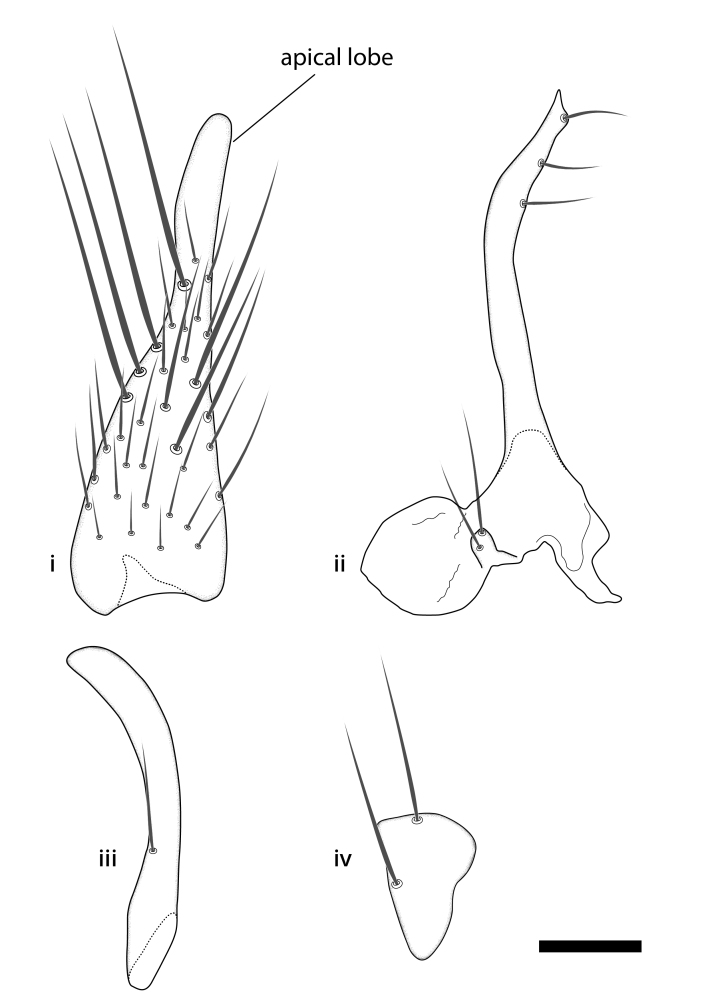
Right gonostylus with: **i** dorsal branch, **ii** internal branch, **iii** medial branch and **iv** ventral branch separated. Scale = 50 μm.

**Figure 26a.**
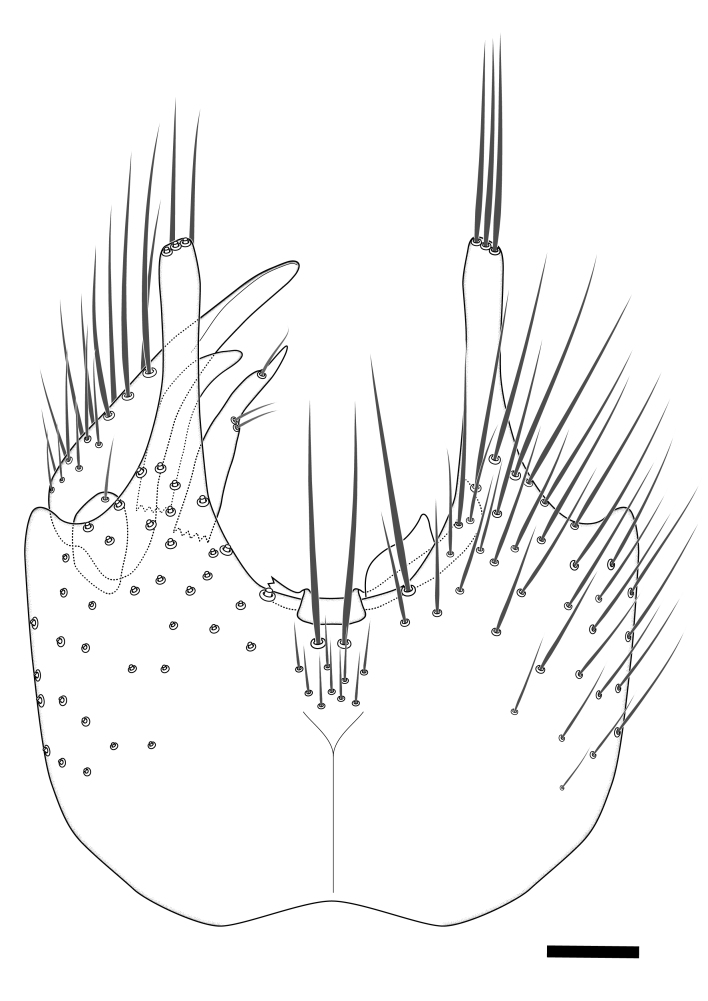
Ventral view. Right gonostylus and setae on left gonocoxite not drawn. Scale = 50 μm.

**Figure 26b.**
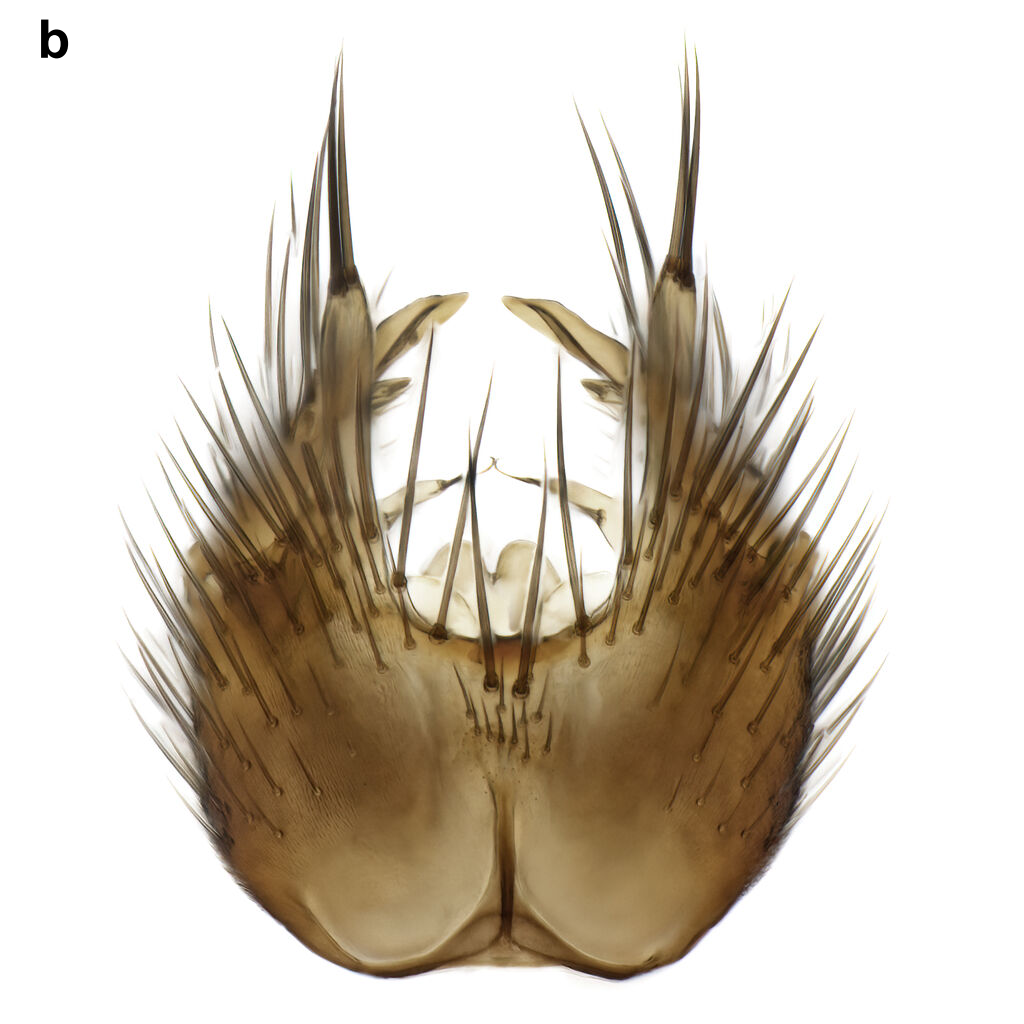
Ventral view. Photo.

**Figure 26c.**
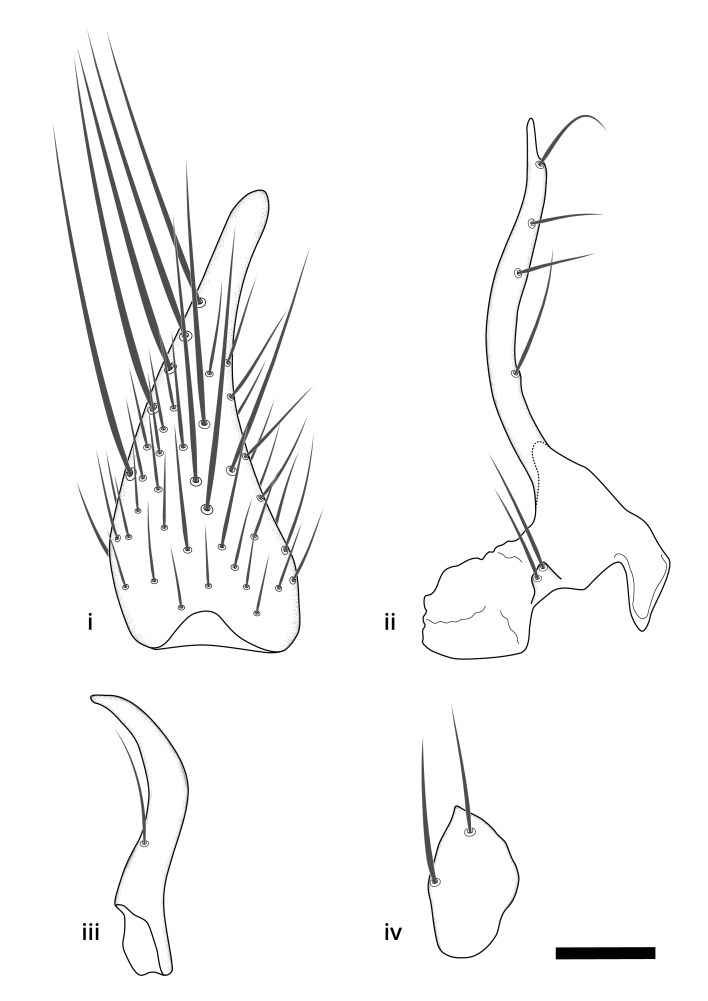
Right gonostylus with: **i** dorsal branch, **ii** internal branch, **iii** medial branch and **iv** ventral branch separated. Scale = 50 μm.

**Figure 27a.**
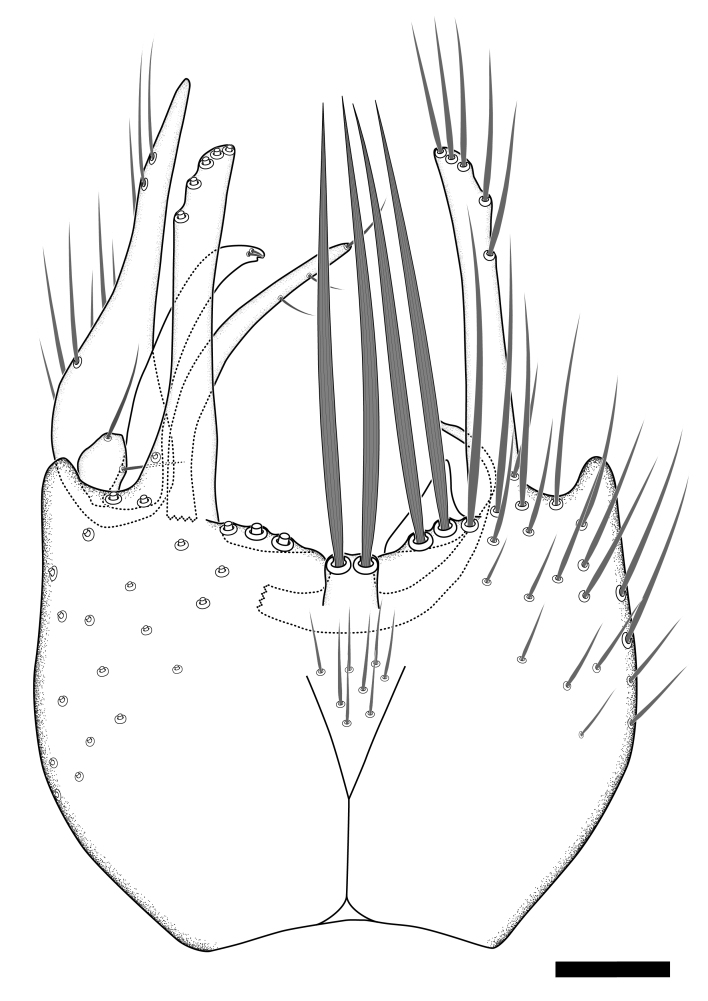
Ventral view. Left gonocoxal setae and right gonostylus not drawn. Scale = 50 μm.

**Figure 27b.**
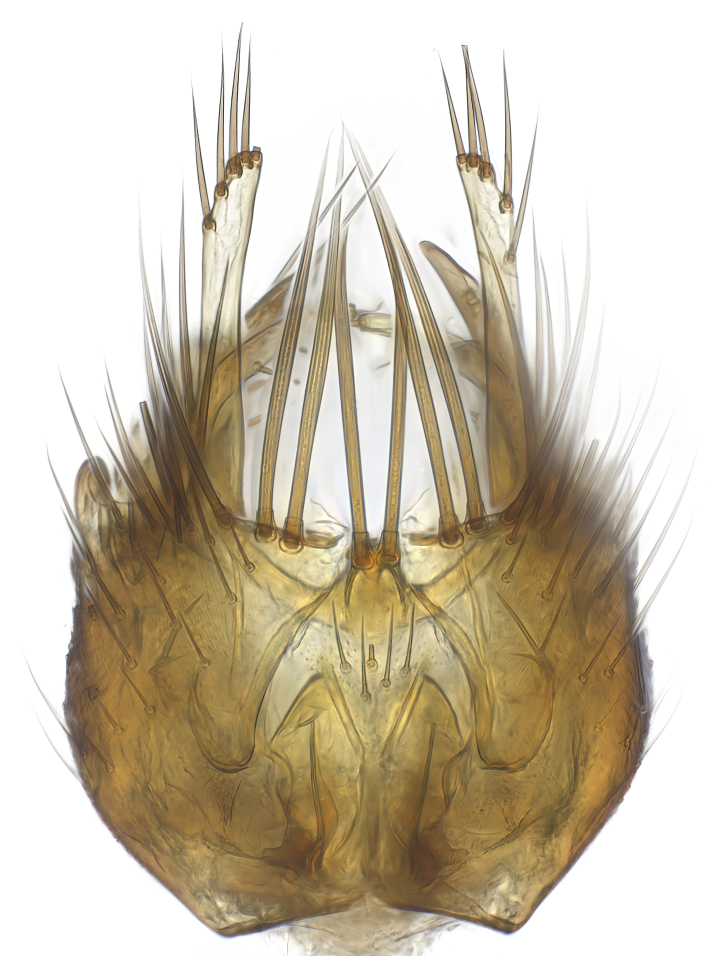
Ventral view. Photo.

**Figure 27c.**
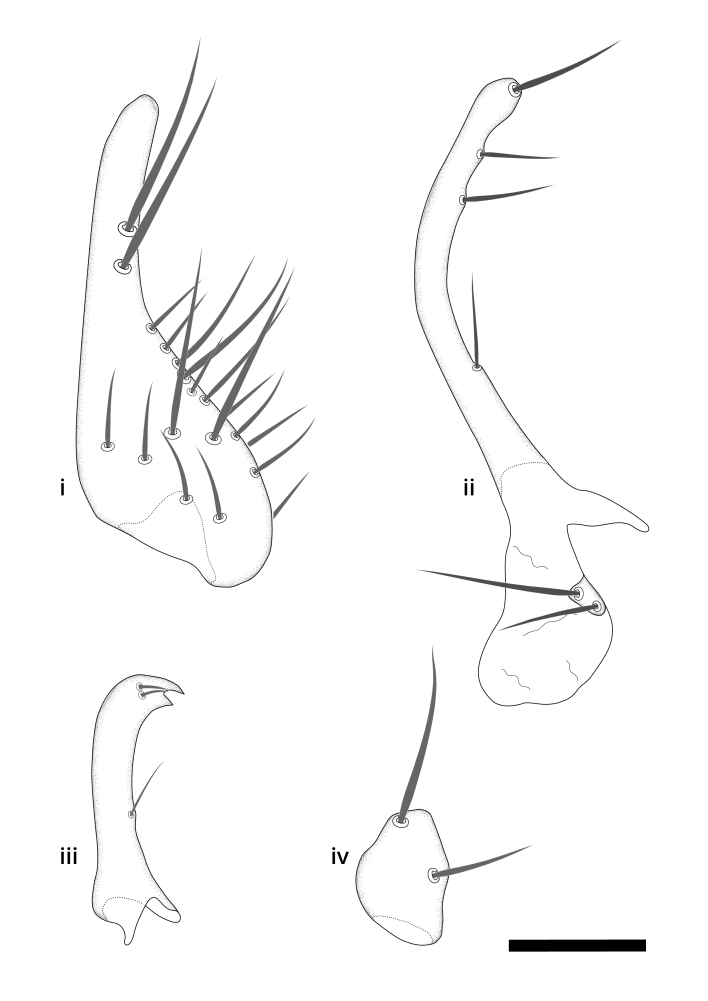
Right gonostylus with: **i** dorsal branch, **ii** internal branch, **iii** medial branch and **iv** ventral branch separated. Scale = 50 μm.

**Figure 28a.**
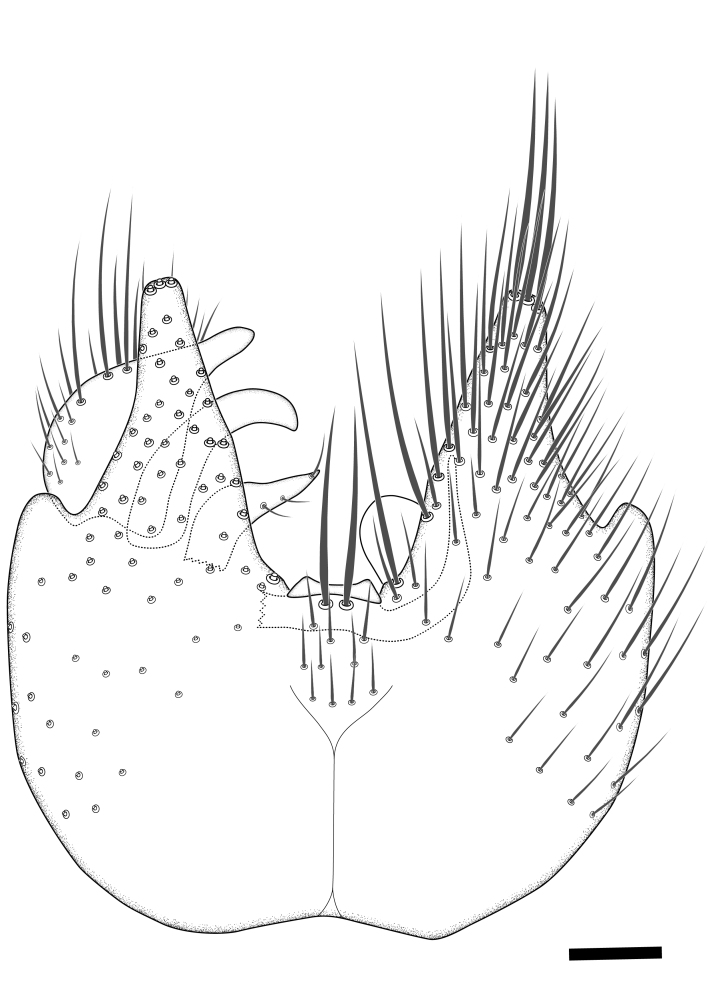
Male terminalia ventral view. Left gonocoxal setae and right gonostylus not drawn. Scale = 50 μm.

**Figure 28b.**
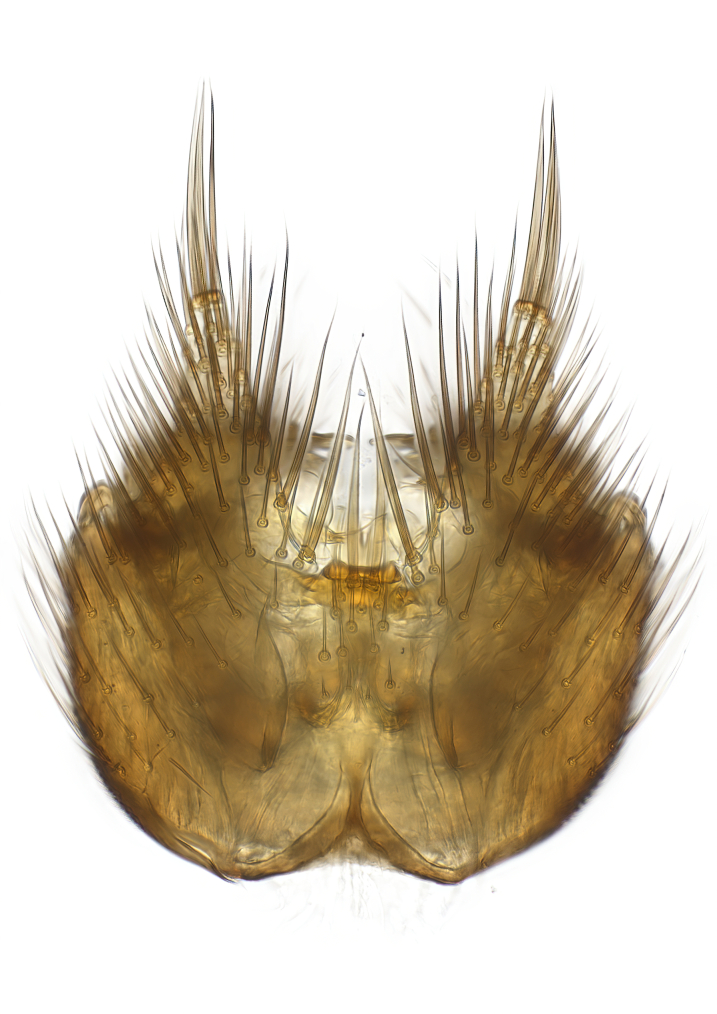
Male terminalia ventral view. Photo.

**Figure 28c.**
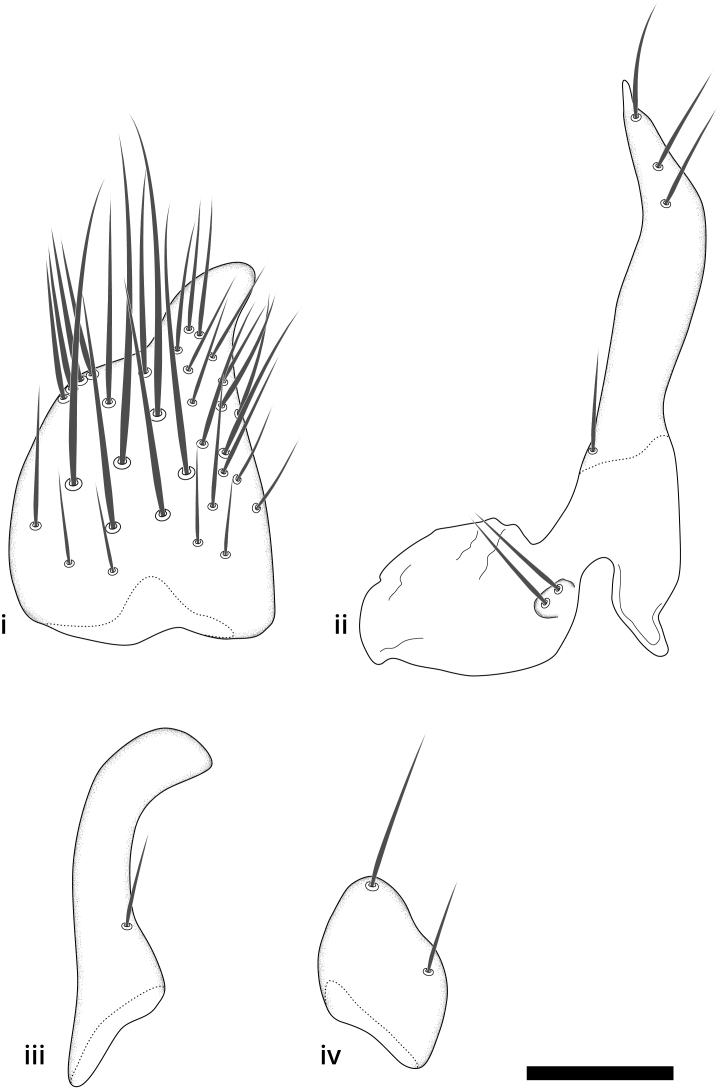
Male right gonostylus with: **i** dorsal branch, **ii** internal branch, **iii** medial branch and **iv** ventral branch separated. Scale = 50 μm.

**Figure 28d.**
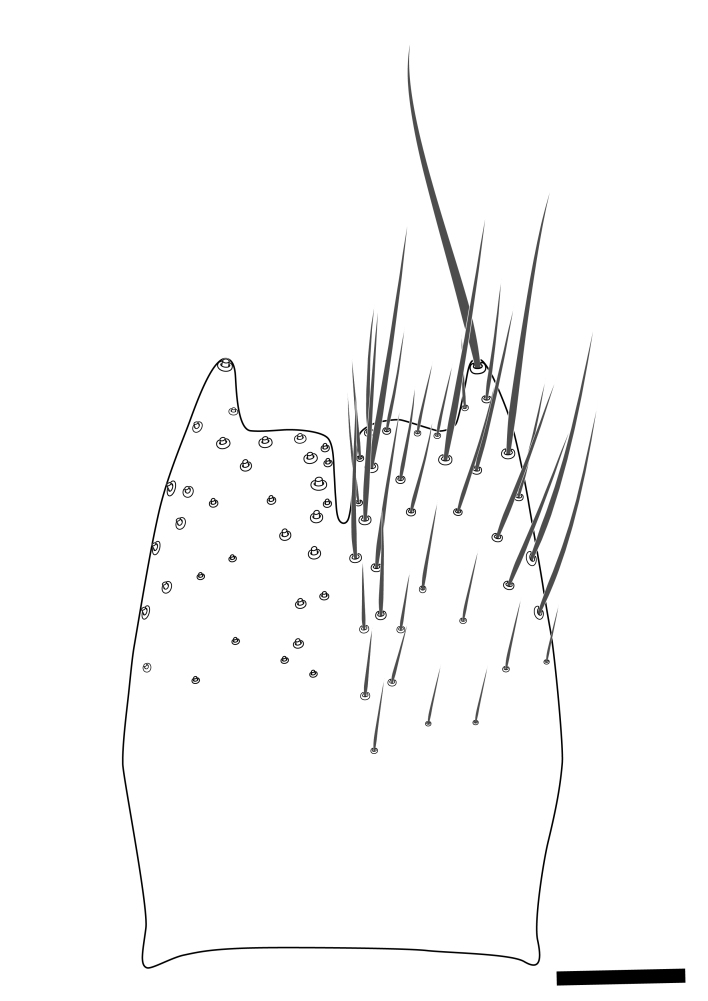
Female sternite VIII ventral view. Setae on left half not drawn. Scale = 50 μm.

**Figure 28e.**
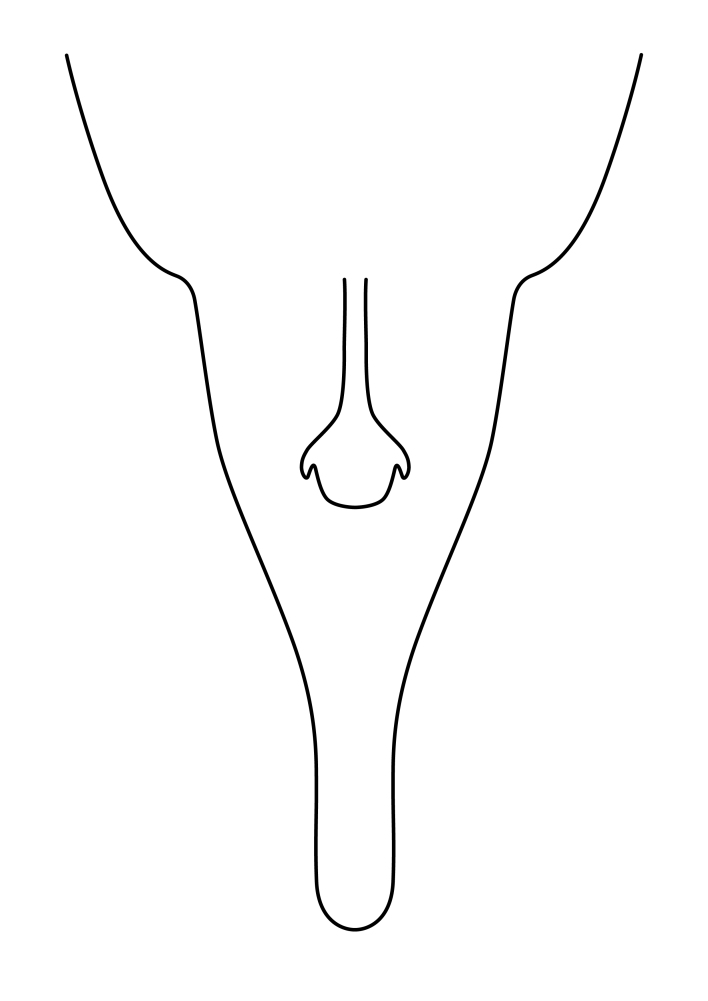
Female gonapophysis IX and spermathecal eminence in ventral view.

**Figure 29a.**
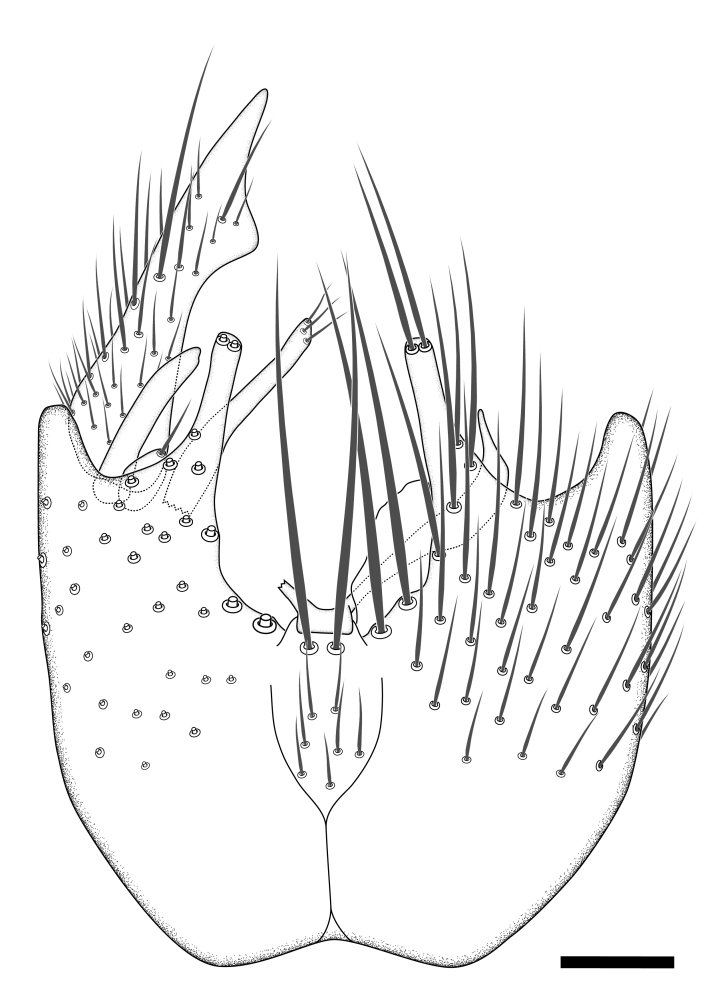
Male terminalia ventral view. Right gonostylus and setae on left gonocoxite not drawn. Scale = 50 μm.

**Figure 29b.**
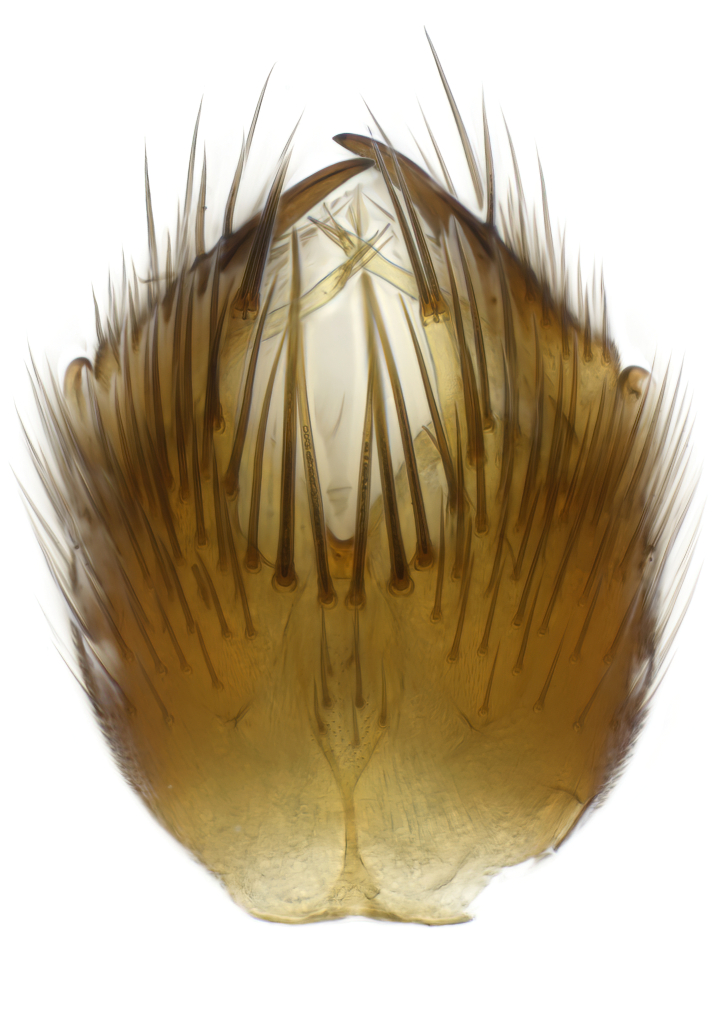
Male terminalia ventral view. Photo.

**Figure 29c.**
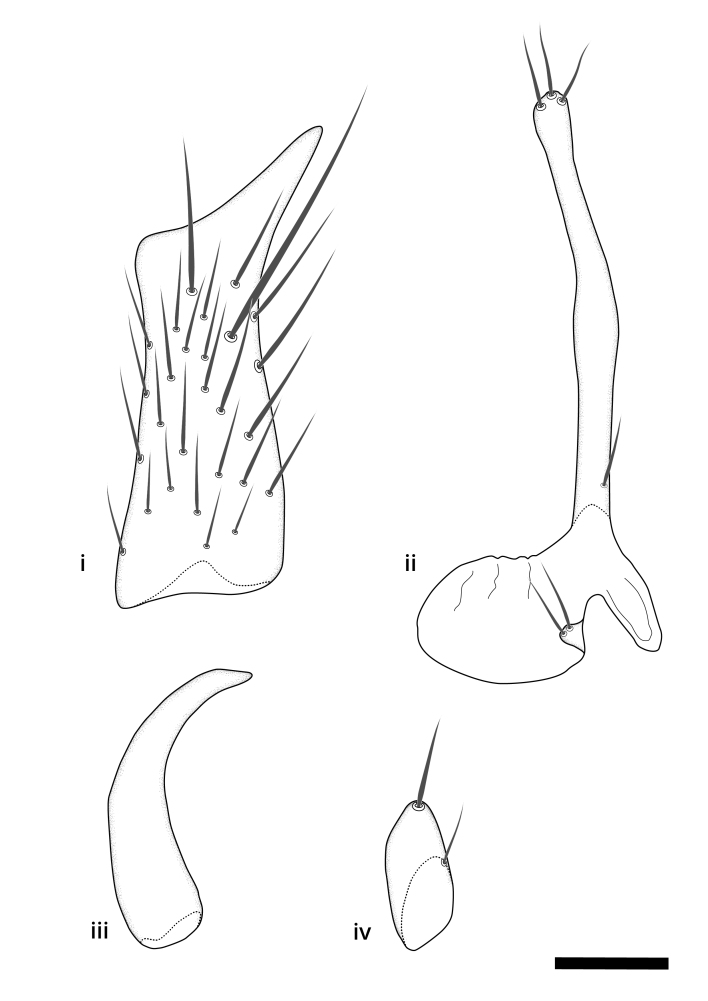
Male right gonostylus with: **i** dorsal branch, **ii** internal branch, **iii** medial branch and **iv** ventral branch separated. Scale = 50 μm.

**Figure 29d.**
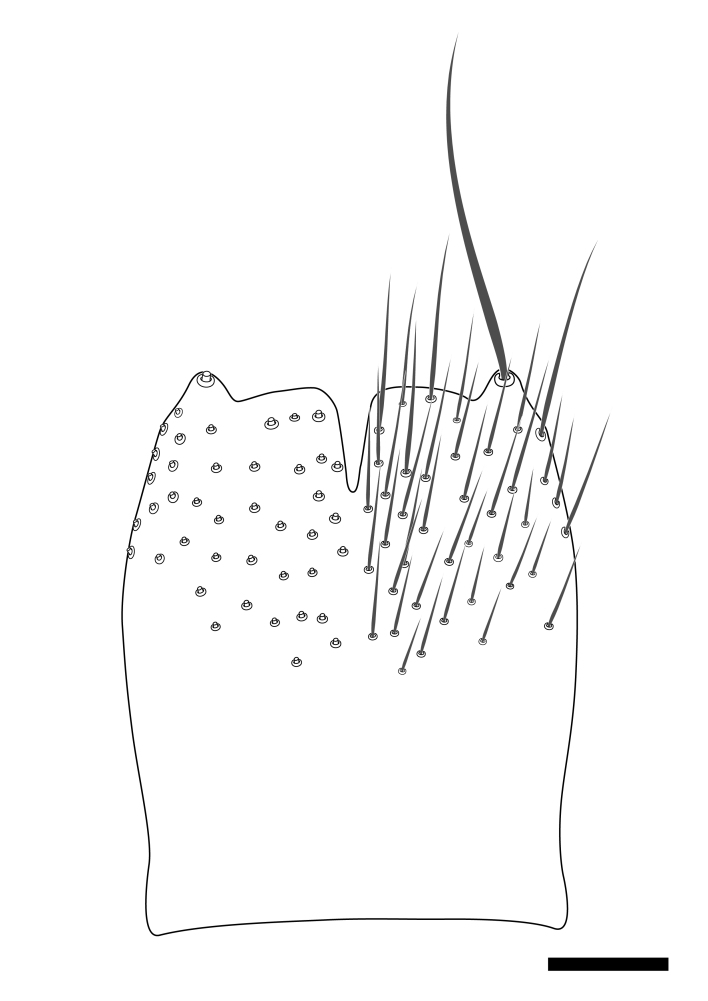
Female sternite VIII ventral view. Setae on left half not drawn. Scale = 50 μm.

**Figure 29e.**
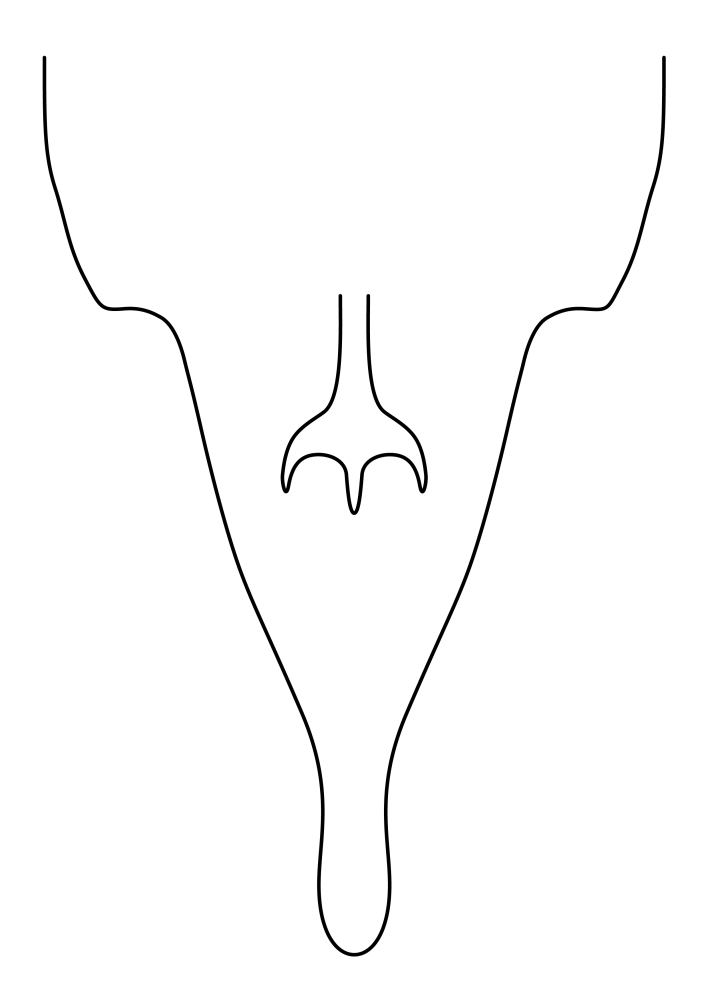
Female gonapophysis IX and spermathecal eminence in ventral view.

**Figure 30a.**
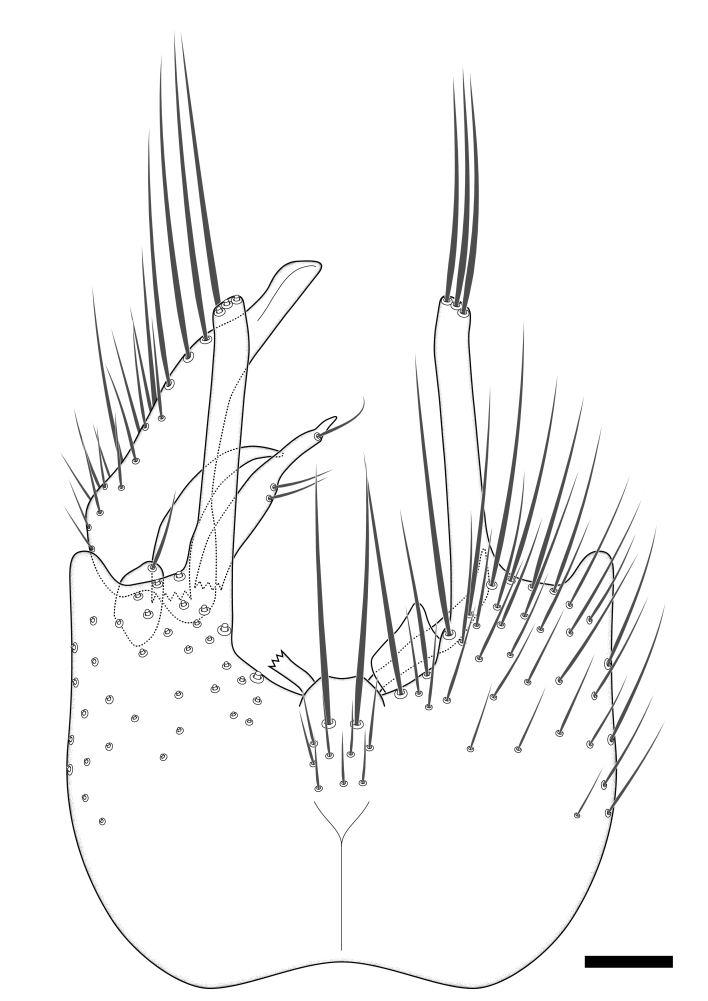
Ventral view. Right gonostylus and setae on left gonocoxite not drawn. Scale = 50 μm.

**Figure 30b.**
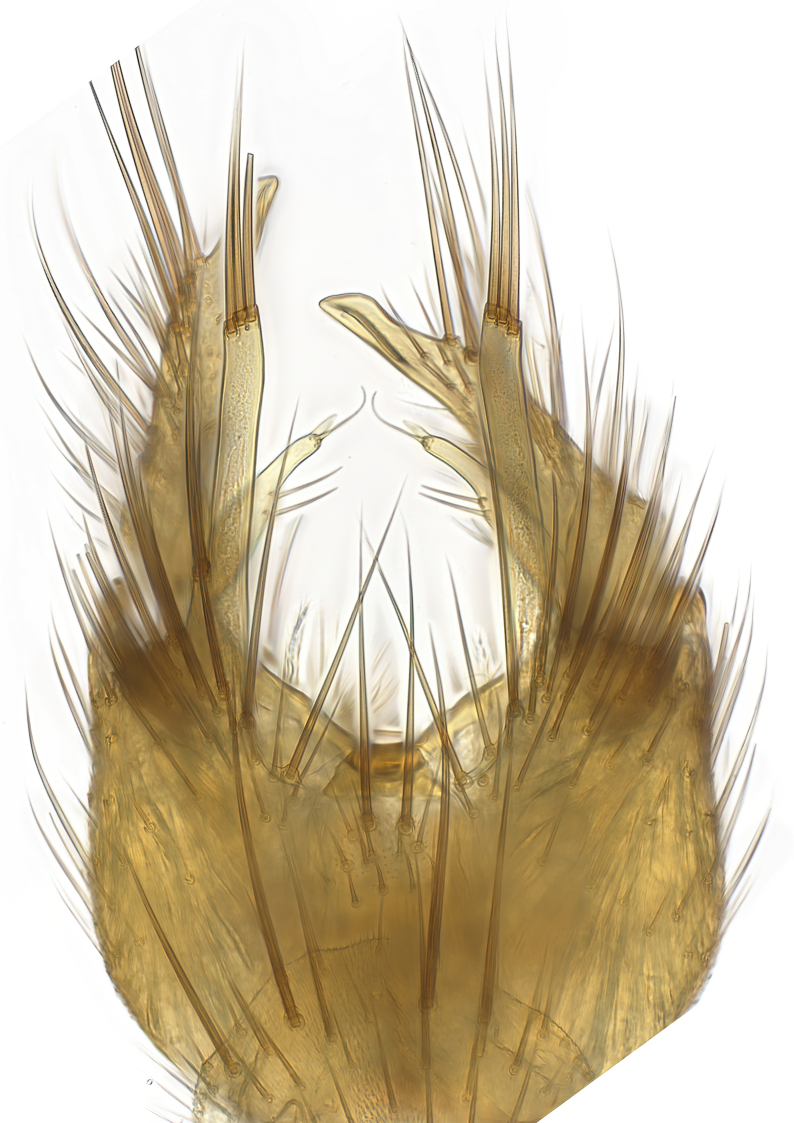
Ventral view. Photo.

**Figure 30c.**
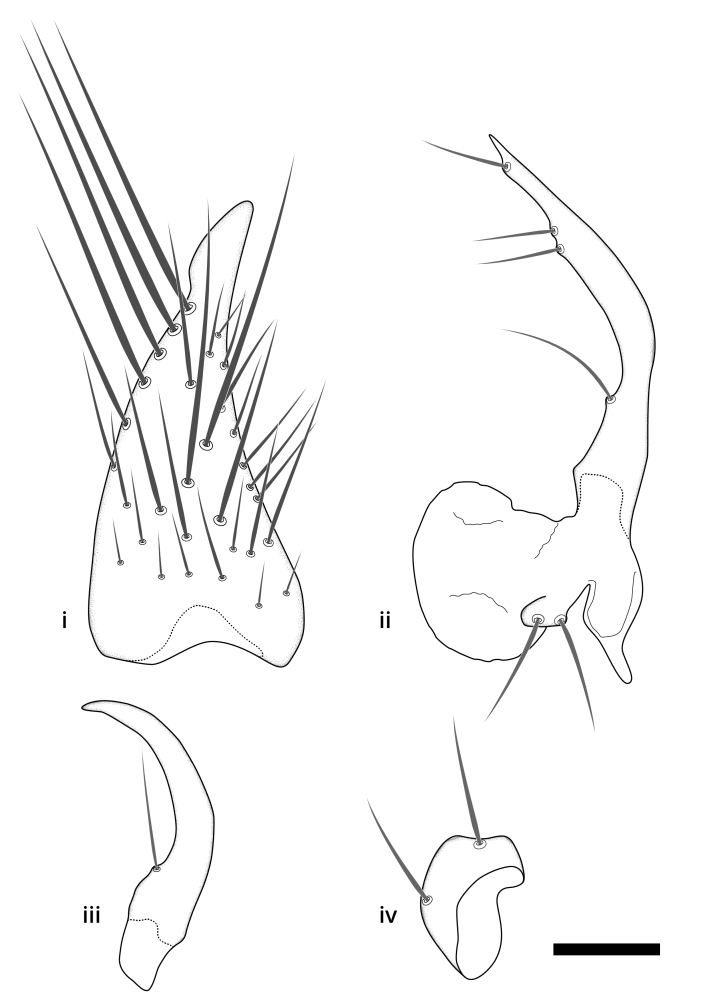
Right gonostylus with: **i** dorsal branch, **ii** internal branch, **iii** medial branch and **iv** ventral branch separated. Scale = 50 μm.

**Figure 31a.**
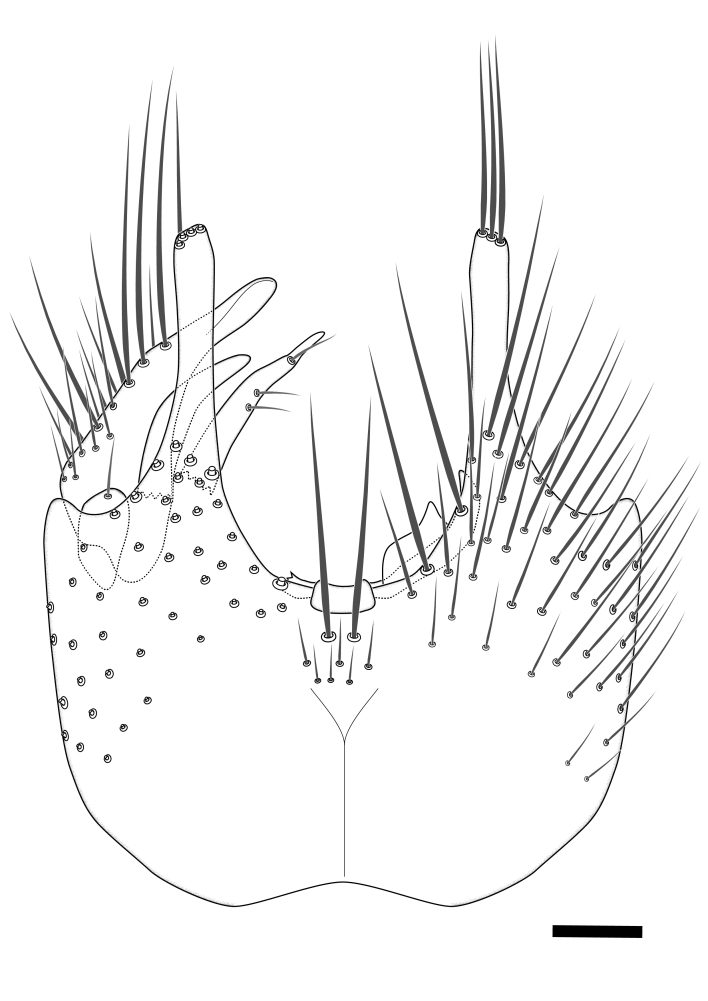
Male terminalia ventral view. Left gonocoxal setae and right gonostylus not drawn. Scale = 50 μm.

**Figure 31b.**
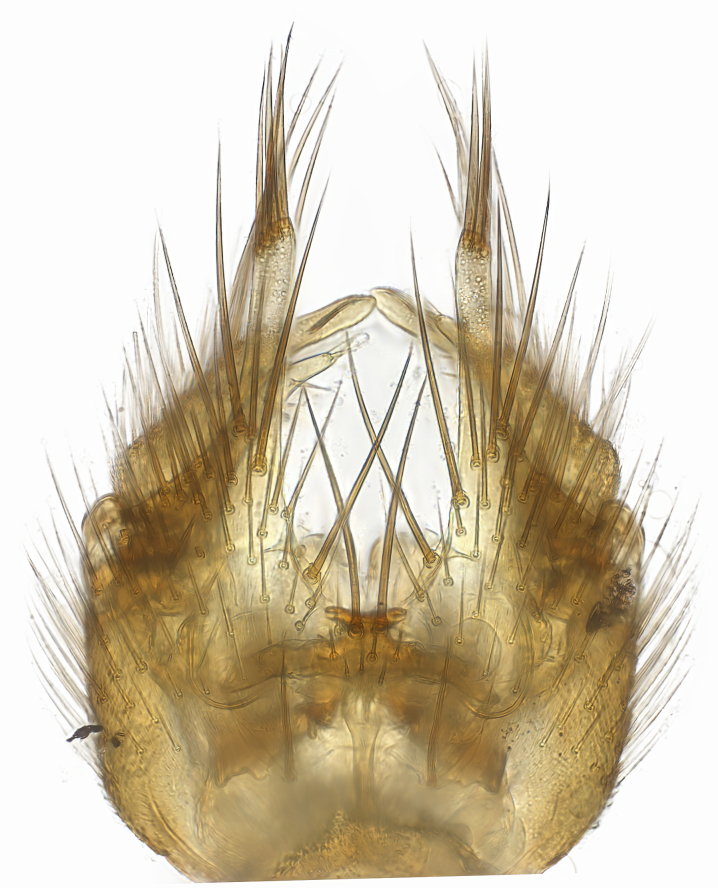
Male terminalia ventral view. Photo.

**Figure 31c.**
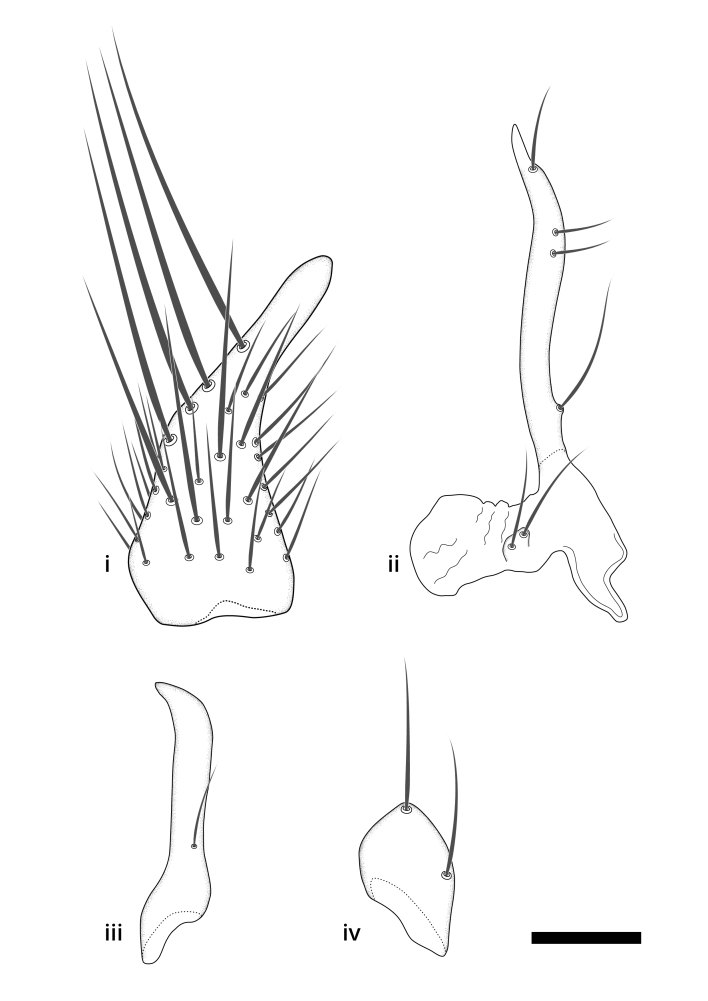
Male right gonostylus with: **i** dorsal branch, **ii** internal branch, **iii** medial branch and **iv** ventral branch separated. Scale = 50 μm.

**Figure 31d.**
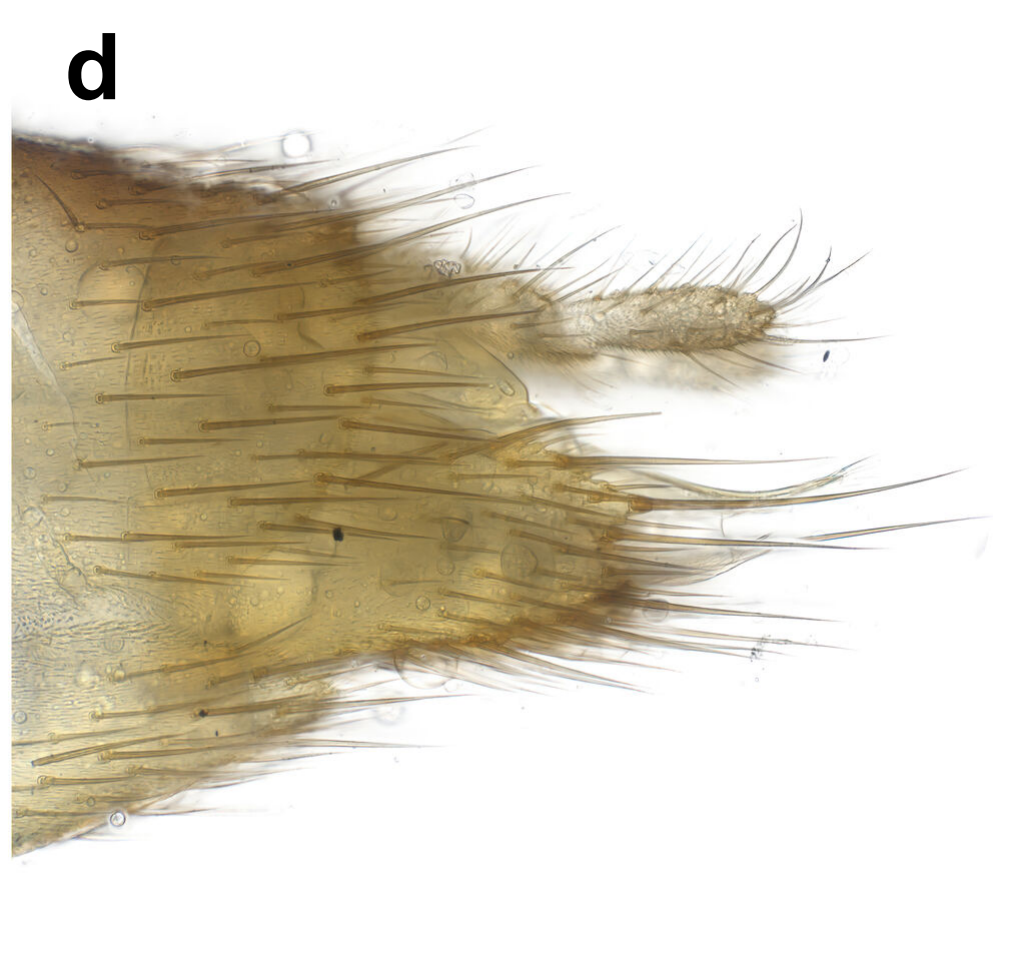
Female terminalia, lateral view. Photo.

**Figure 31e.**
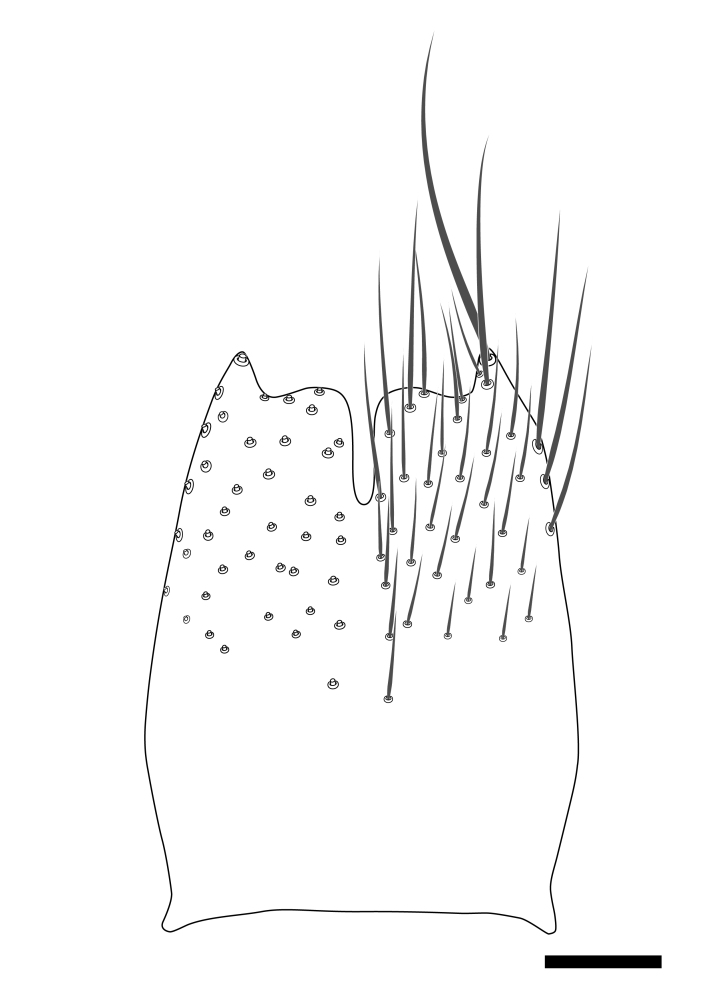
Female sternite VIII ventral view. Setae on left half not drawn. Scale = 50 μm.

**Figure 32a.**
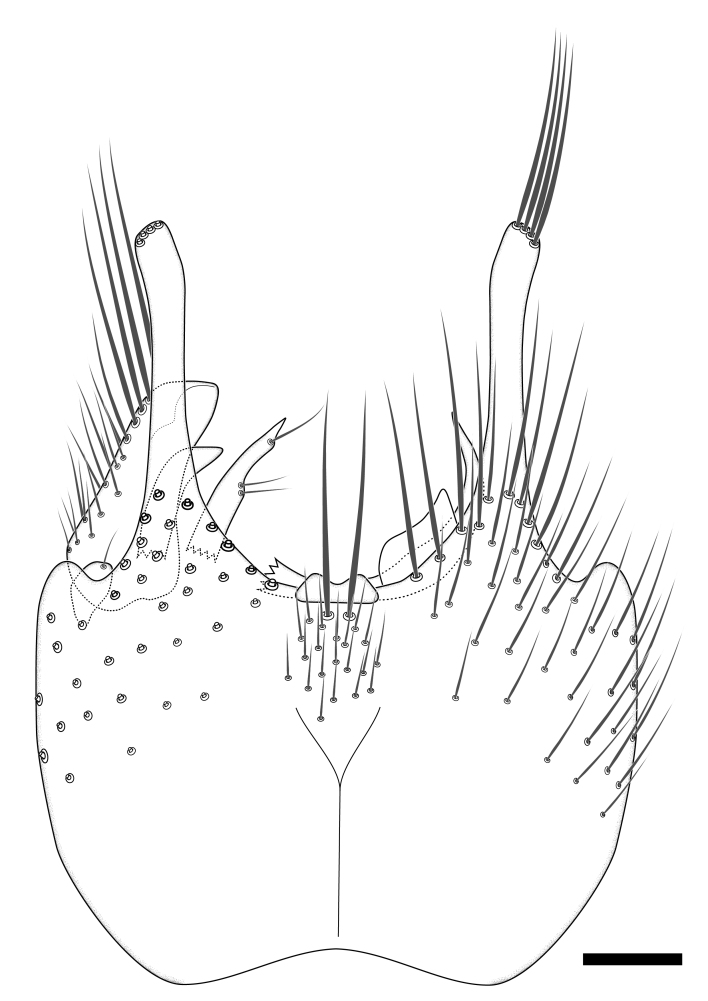
Ventral view. Right gonostylus and setae on left gonocoxite not drawn. Scale = 50 μm.

**Figure 32b.**
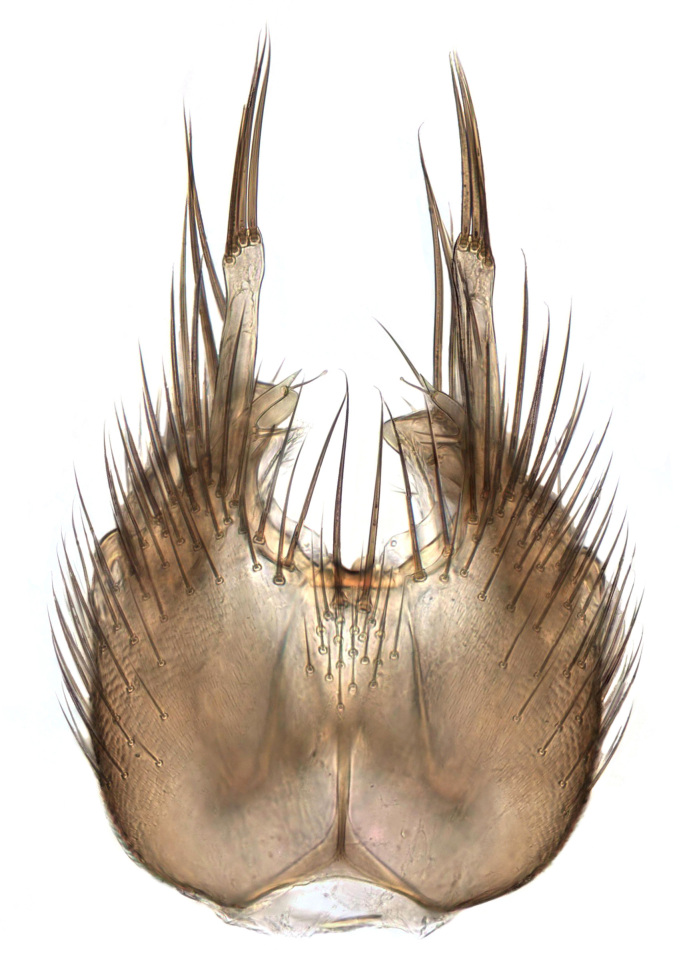
Ventral view. Photo by courtesy of Janet Graham.

**Figure 32c.**
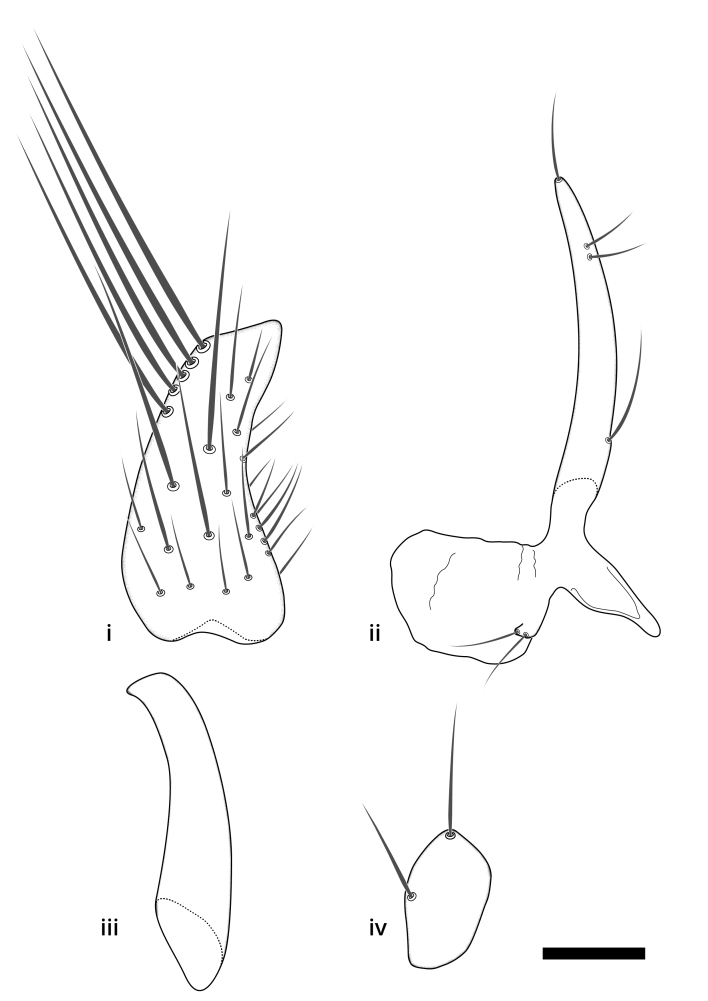
Right gonostylus with: **i** dorsal branch, **ii** internal branch, **iii** medial branch and **iv** ventral branch separated. Scale = 50 μm.

**Figure 32d.**
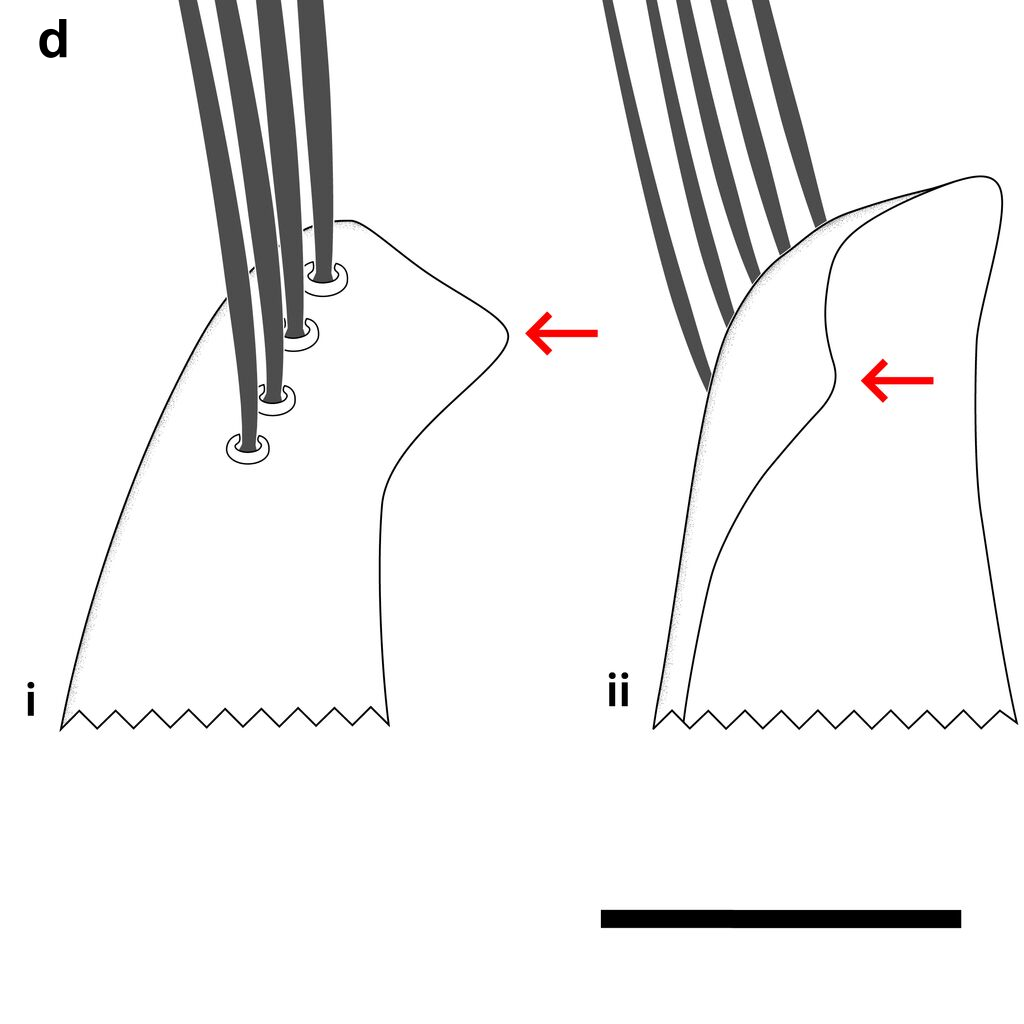
Apical part of dorsal branch of the gonostylus, **i** lateral view, **ii** ventral view. Red arrows indicate the apico-internal angular projection. Scale = 50 μm.

**Figure 33a.**
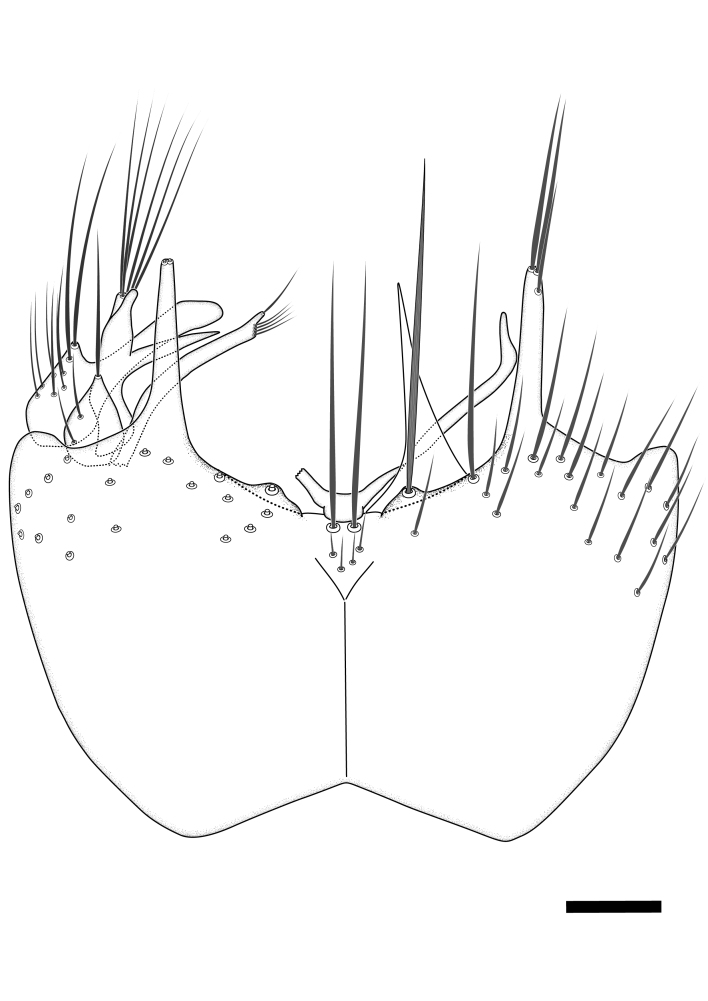
Ventral view. Right gonostylus and setae on left gonocoxite not drawn. Scale = 50 μm.

**Figure 33b.**
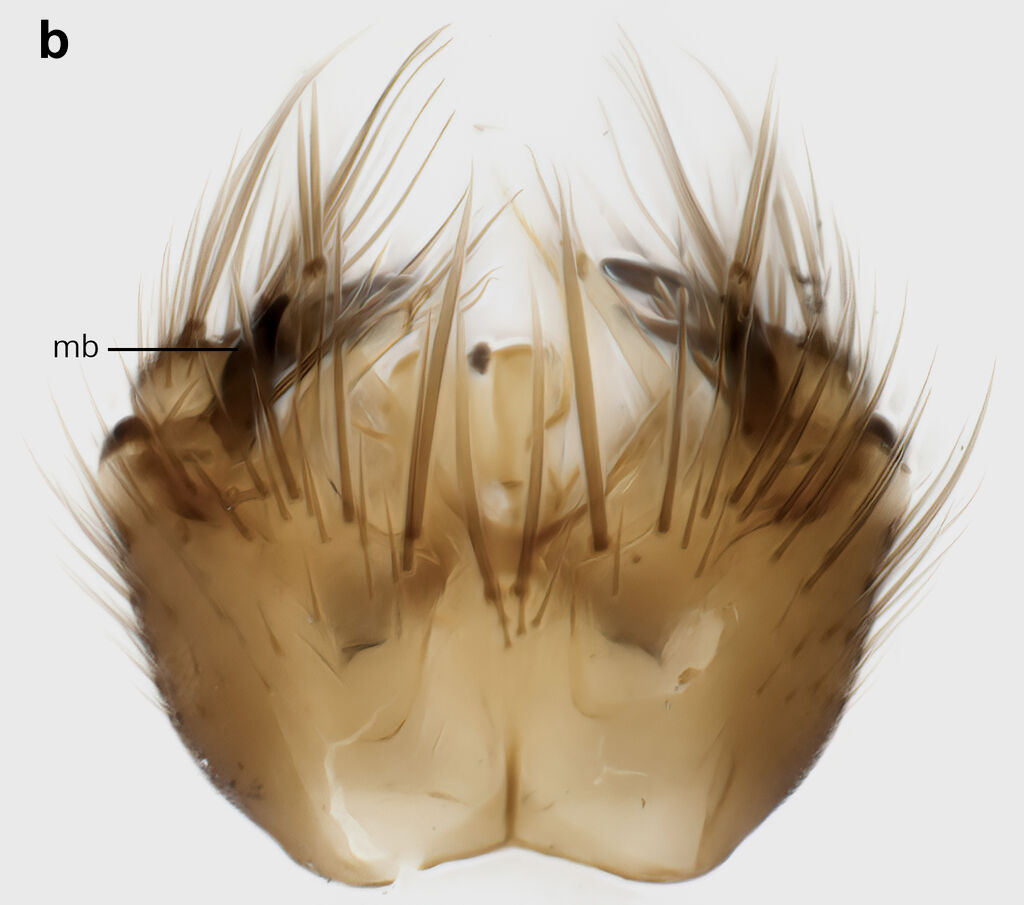
Ventral view. Photo. **Abbreviations**: mb = gonostylus medial branch.

**Figure 33c.**
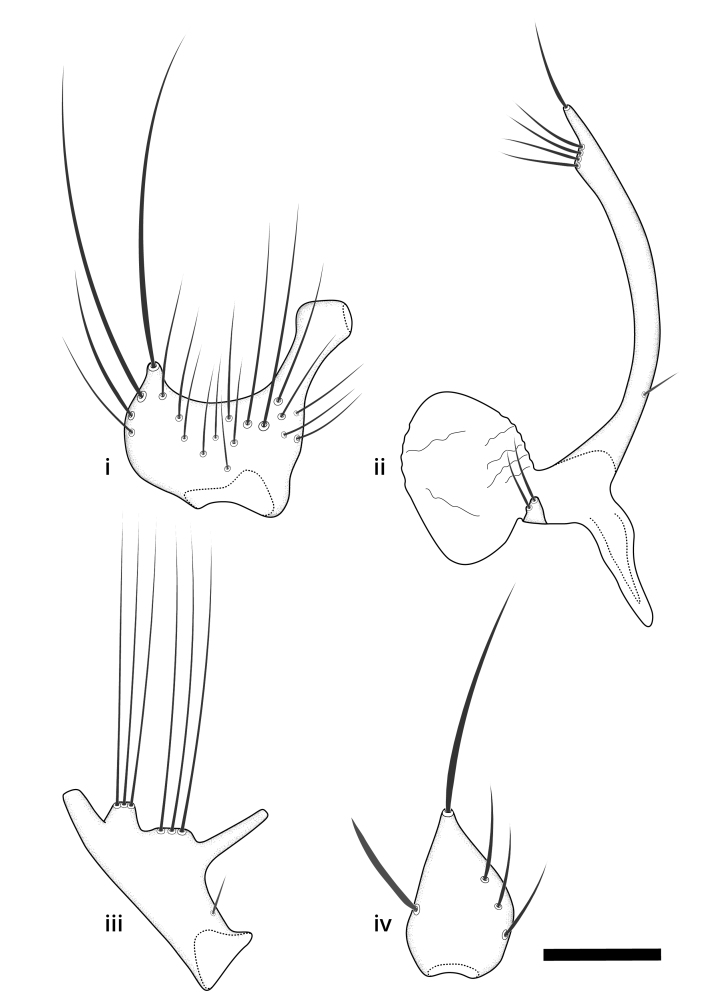
Right gonostylus with: **i** dorsal branch, **ii** internal branch, **iii** medial branch and **iv** ventral branch separated. Scale = 50 μm.

**Figure 33d.**
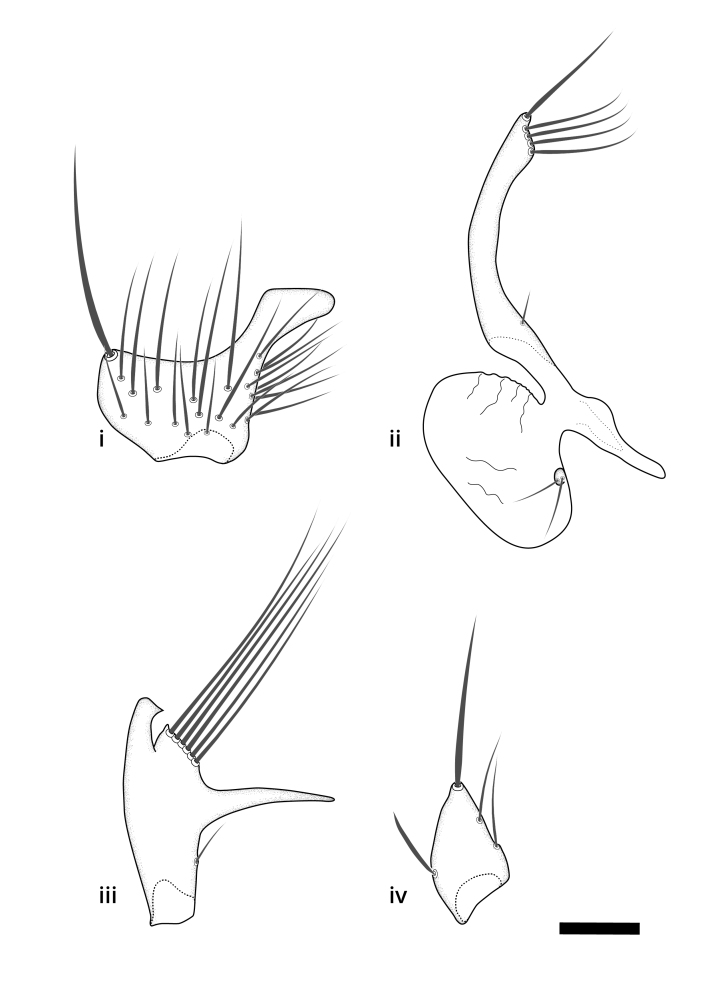
Right gonostylus observed in one variable specimen (Catalogue number TSZD-JKJ-108464), with: **i** dorsal branch, **ii** internal branch, **iii** medial branch and **iv** ventral branch separated. Scale = 50 μm.

**Figure 34a.**
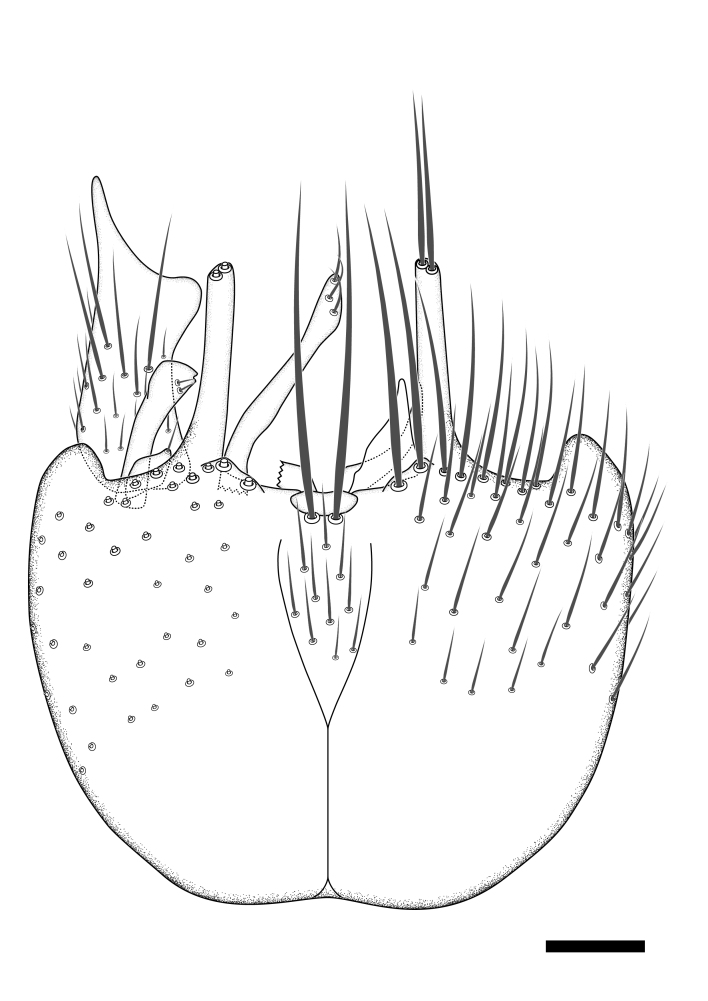
Male terminalia ventral view. Left gonocoxal setae and right gonostylus not drawn. Scale = 50 μm.

**Figure 34b.**
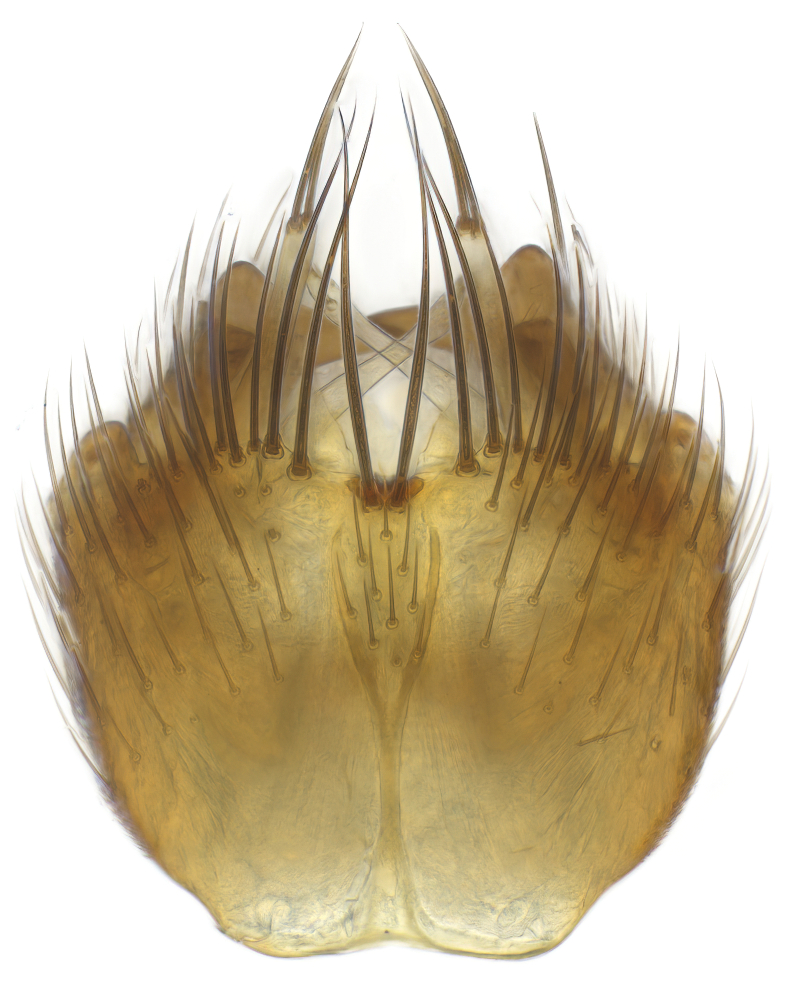
Male terminalia ventral view. Photo.

**Figure 34c.**
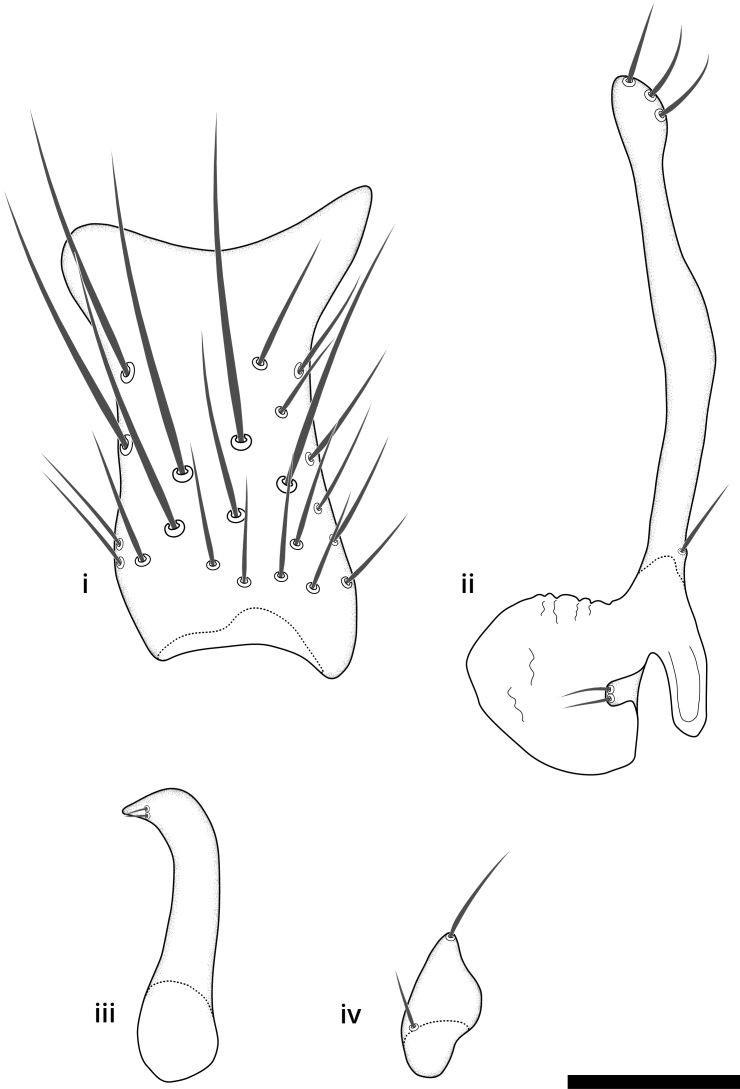
Male right gonostylus with: **i** dorsal branch, **ii** internal branch, **iii** medial branch and **iv** ventral branch separated. Scale = 50 μm.

**Figure 34d.**
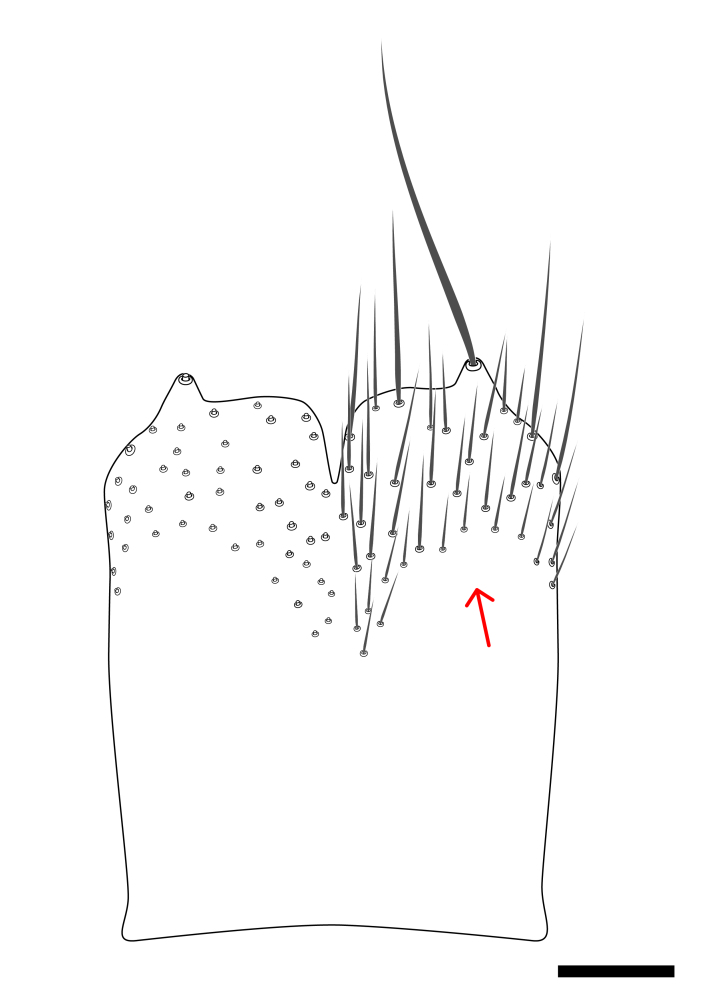
Female sternite VIII ventral view. Setae on left half not drawn. Red arrow indicates bare area. Scale = 50 μm.

**Figure 34e.**
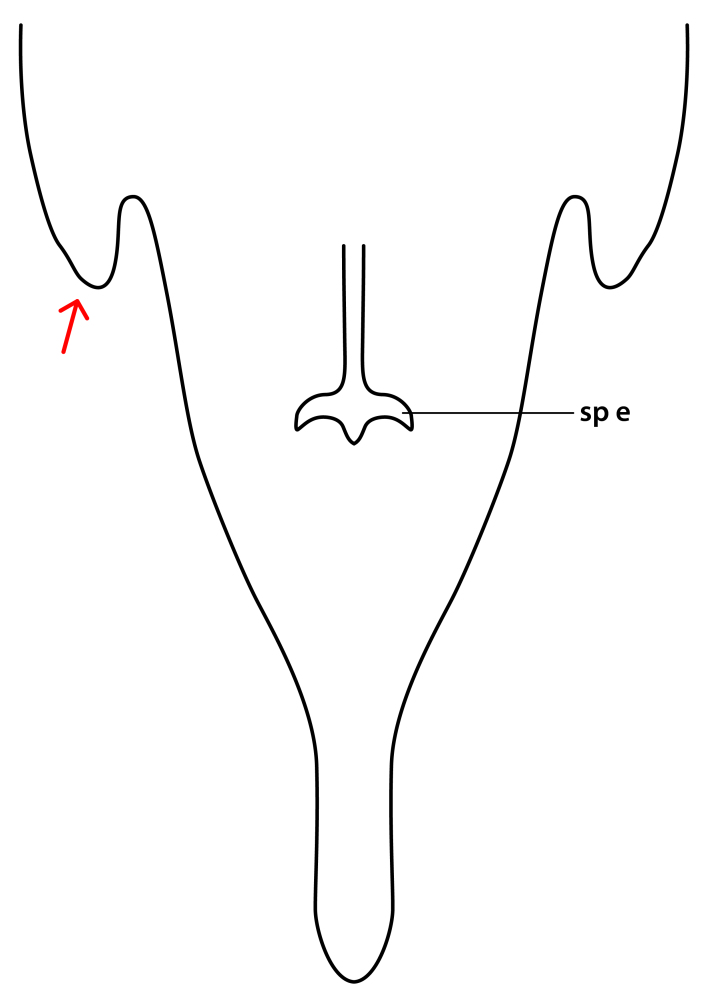
Female gonapophysis IX and spermathecal eminence in ventral view. Red arrow indicates distally expanded basolateral part. **Abbreviations**: sp e = spermathecal eminence.

**Figure 35a.**
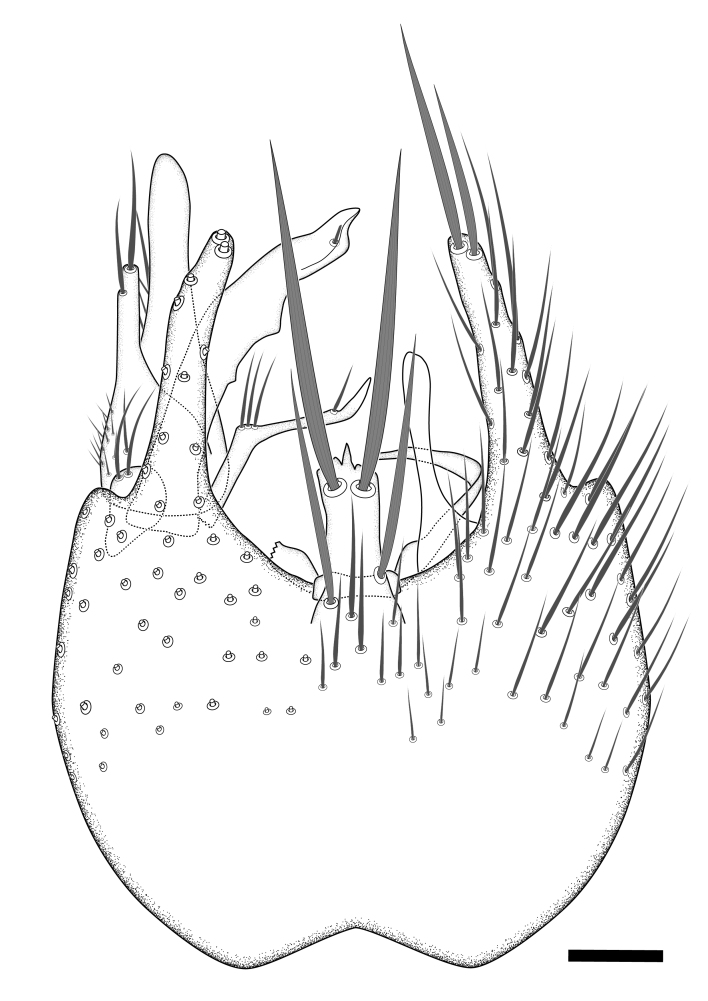
Ventral view. Right gonostylus and setae on left gonocoxite not drawn. Scale = 50 μm.

**Figure 35b.**
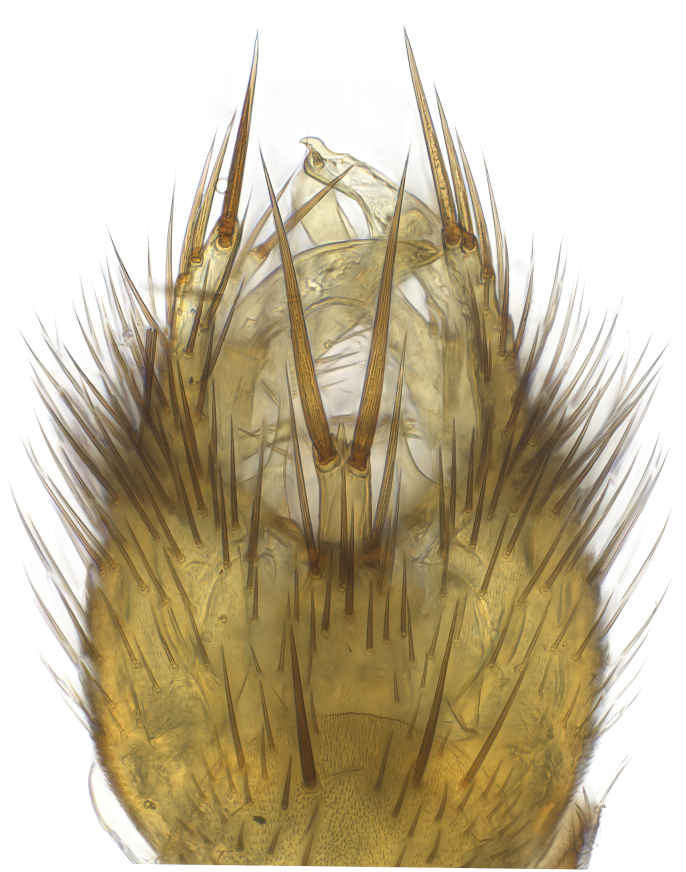
Ventral view. Photo.

**Figure 35c.**
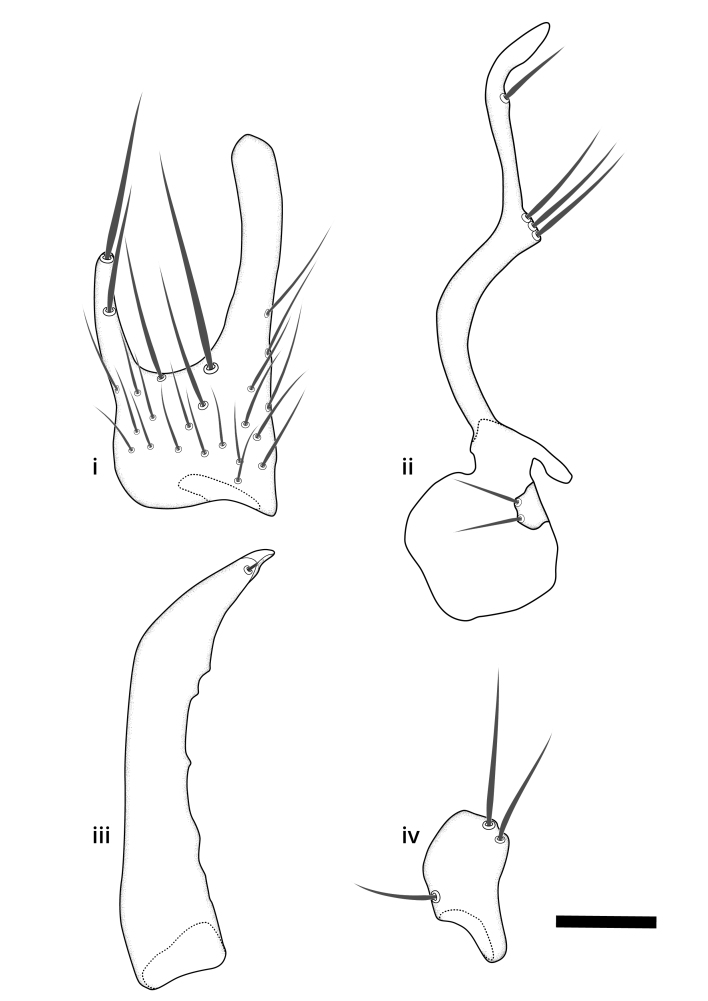
Right gonostylus with: **i** dorsal branch, **ii** internal branch, **iii** medial branch and **iv** ventral branch separated. Scale = 50 μm.

**Figure 36a.**
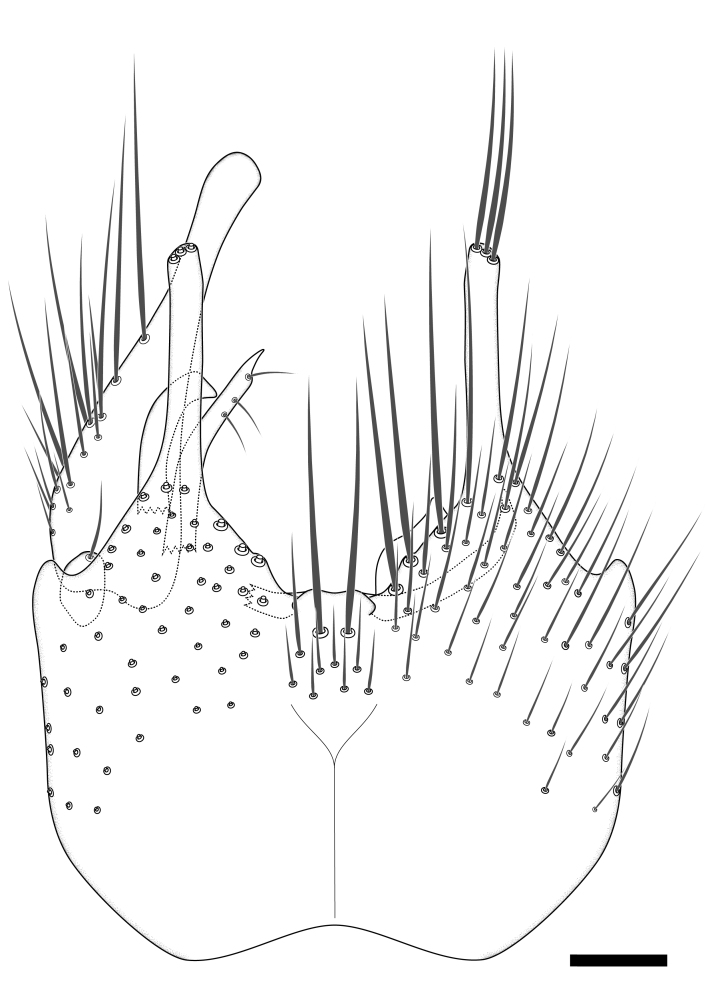
Ventral view. Right gonostylus and setae on left gonocoxite not drawn. Scale = 50 μm.

**Figure 36b.**
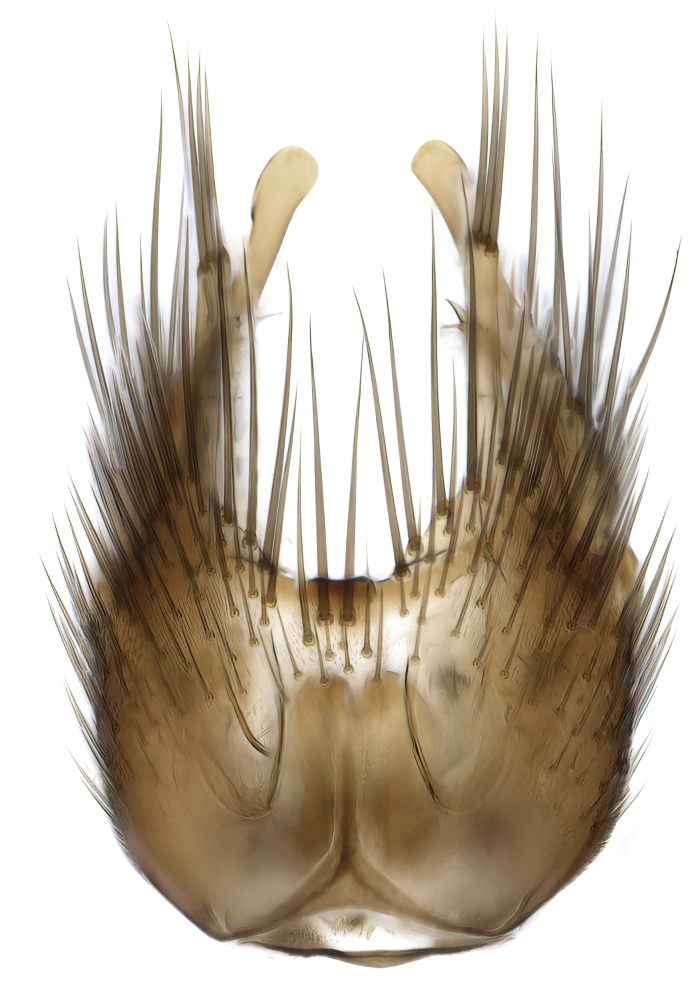
Ventral view. Photo

**Figure 36c.**
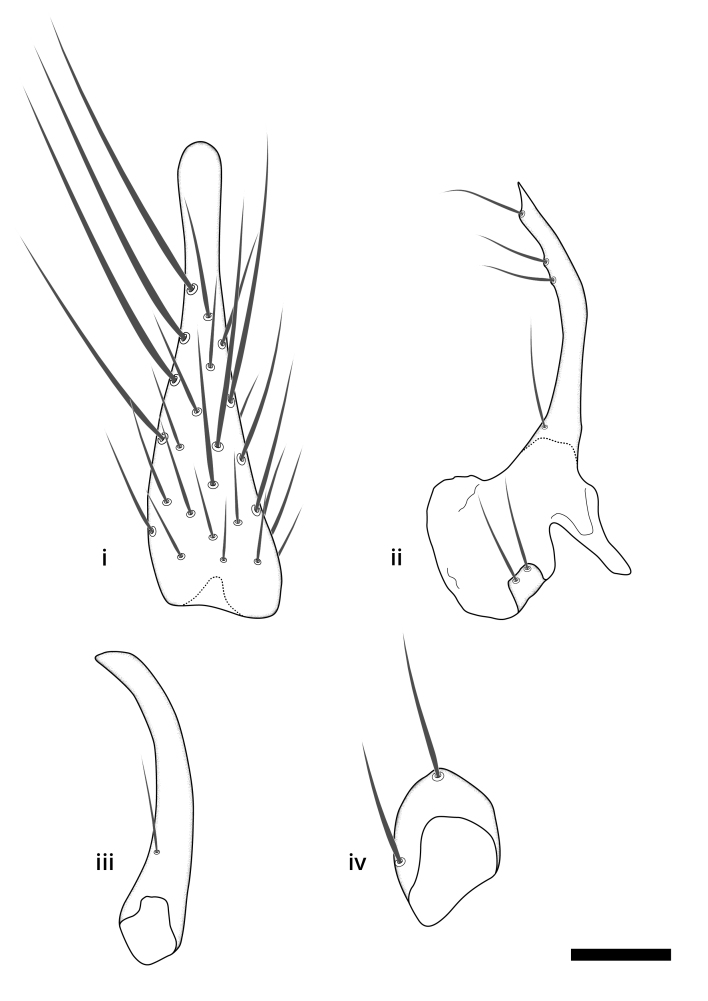
Right gonostylus with: **i** dorsal branch, **ii** internal branch, **iii** medial branch and **iv** ventral branch separated. Scale = 50 μm.

**Figure 37a.**
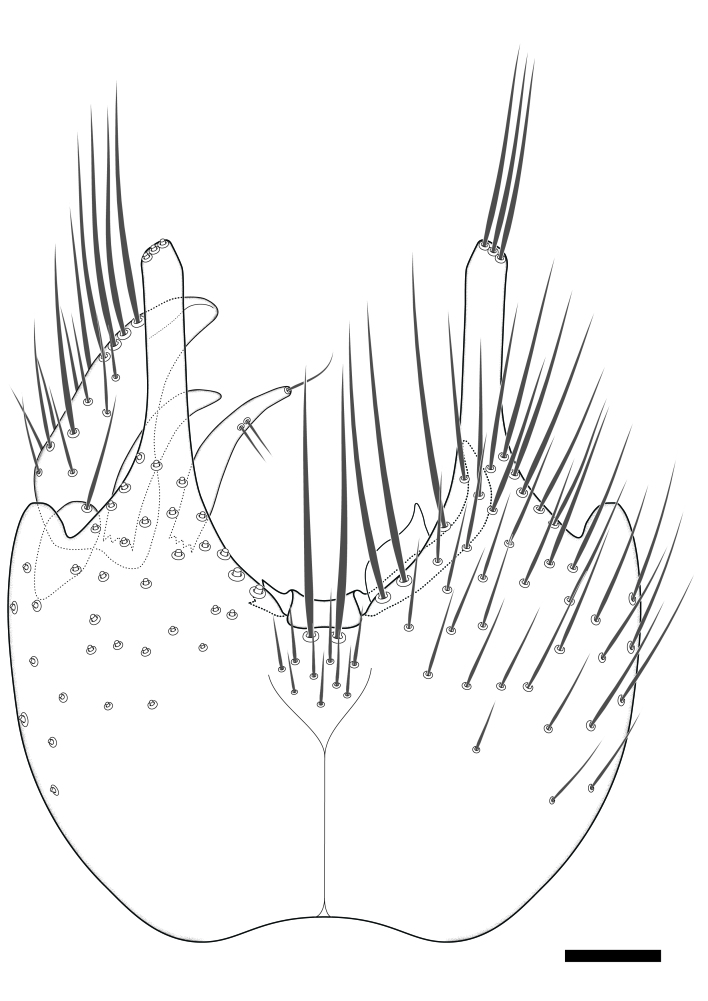
Male terminalia ventral view. Left gonocoxal setae and right gonostylus not drawn. Scale = 50 μm.

**Figure 37b.**
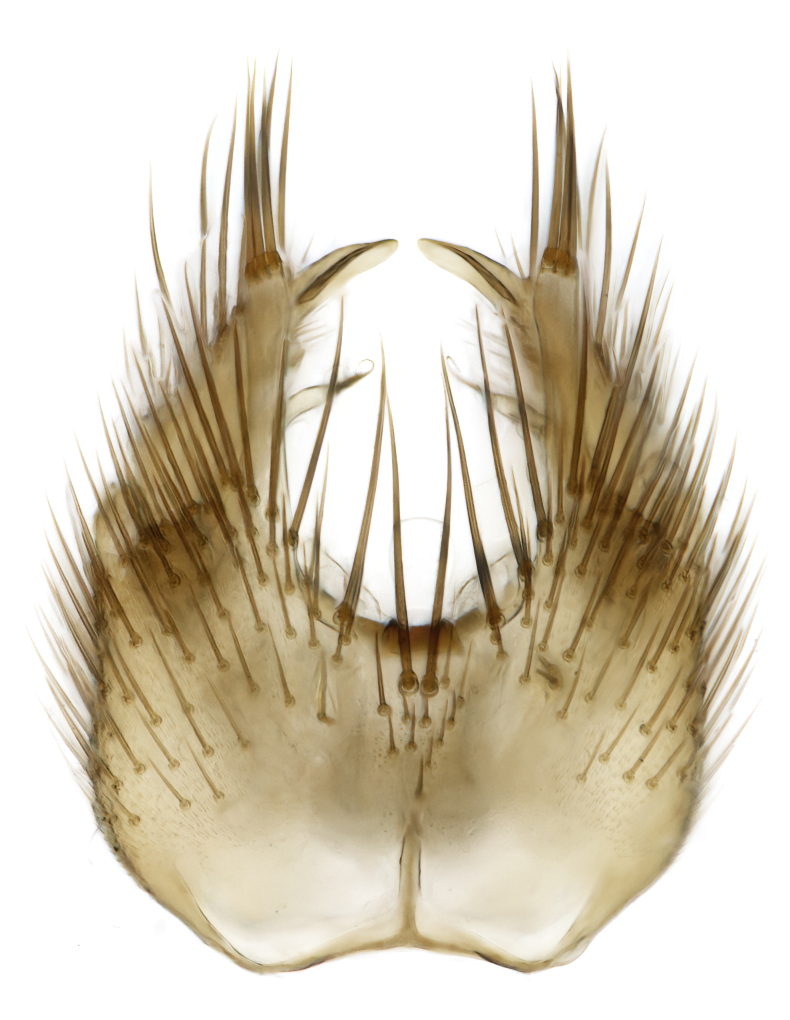
Male terminalia ventral view. Photo.

**Figure 37c.**
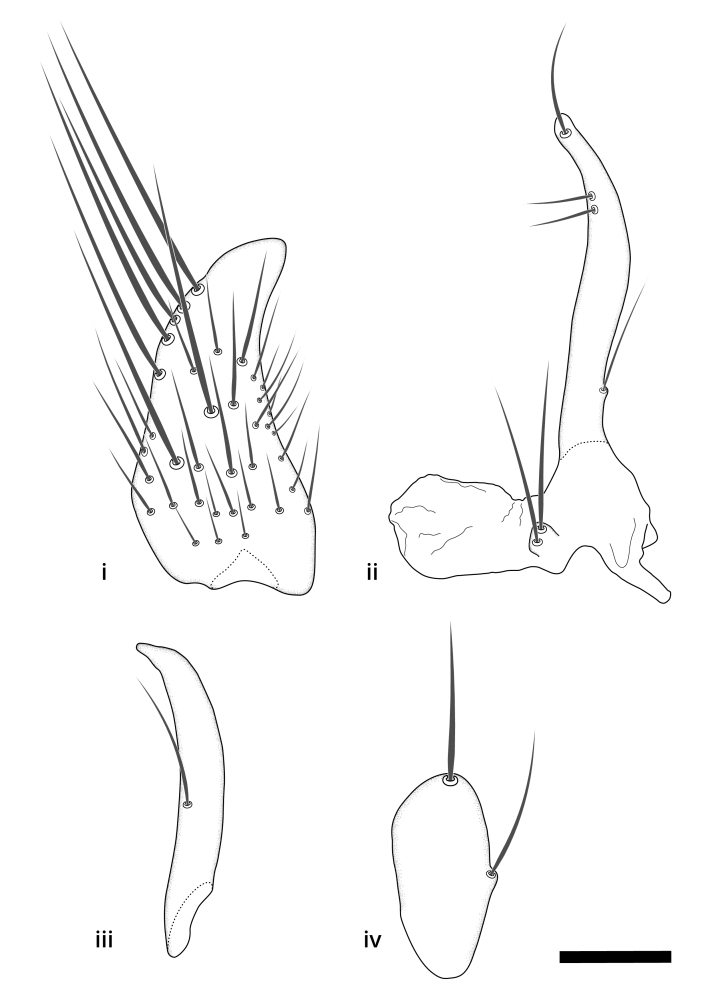
Male right gonostylus with: **i** dorsal branch, **ii** internal branch, **iii** medial branch and **iv** ventral branch separated. Scale = 50 μm.

**Figure 37d.**
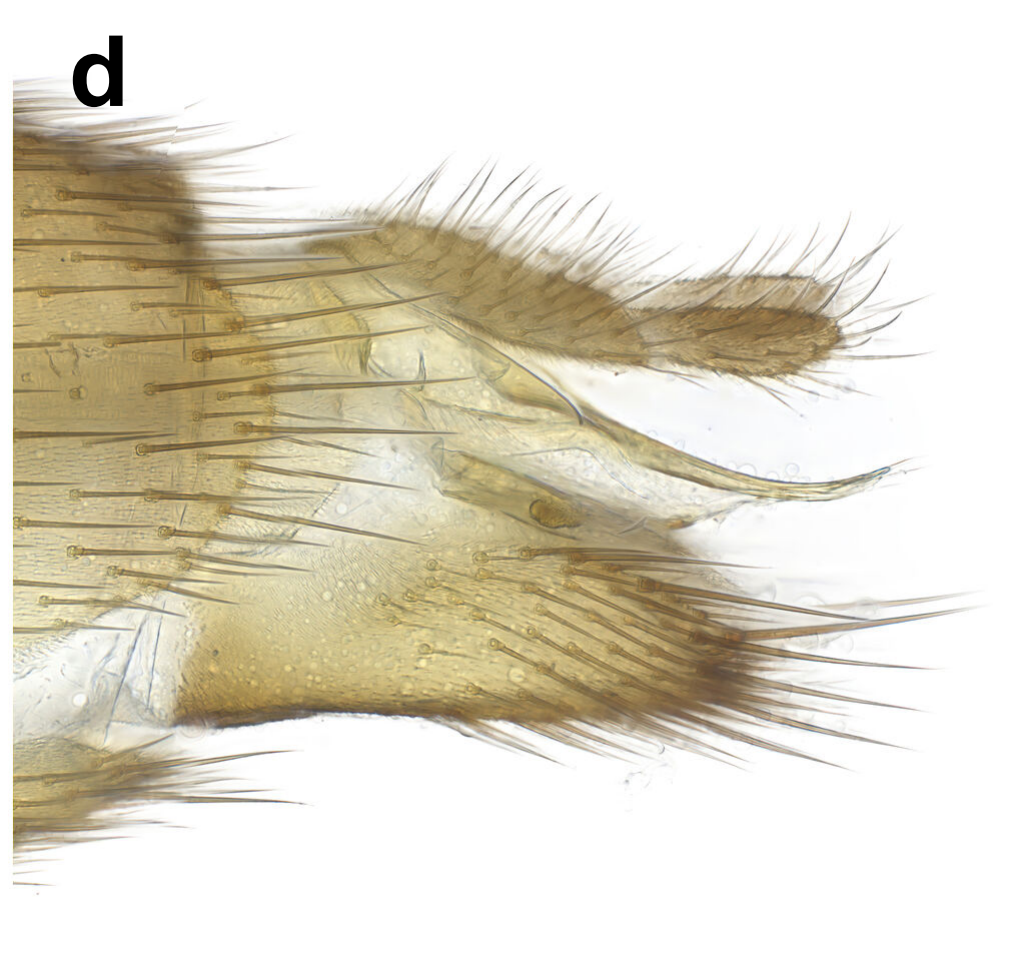
Female terminalia lateral view. Photo.

**Figure 37e.**
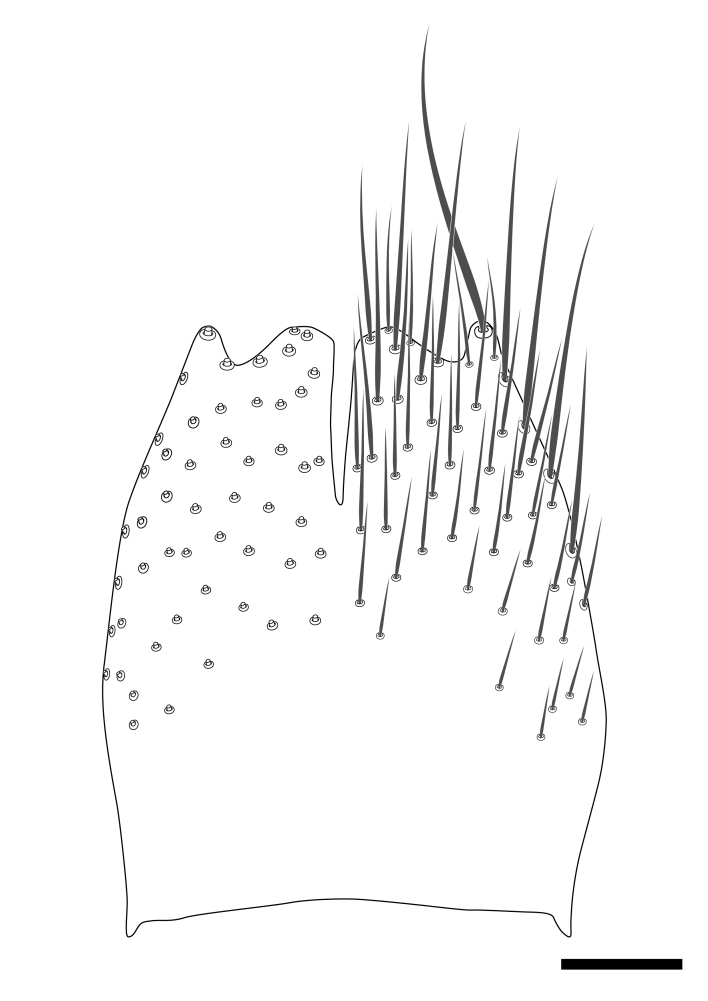
Female sternite VIII ventral view. Setae on left half not drawn. Scale = 50 μm.

**Figure 37f.**
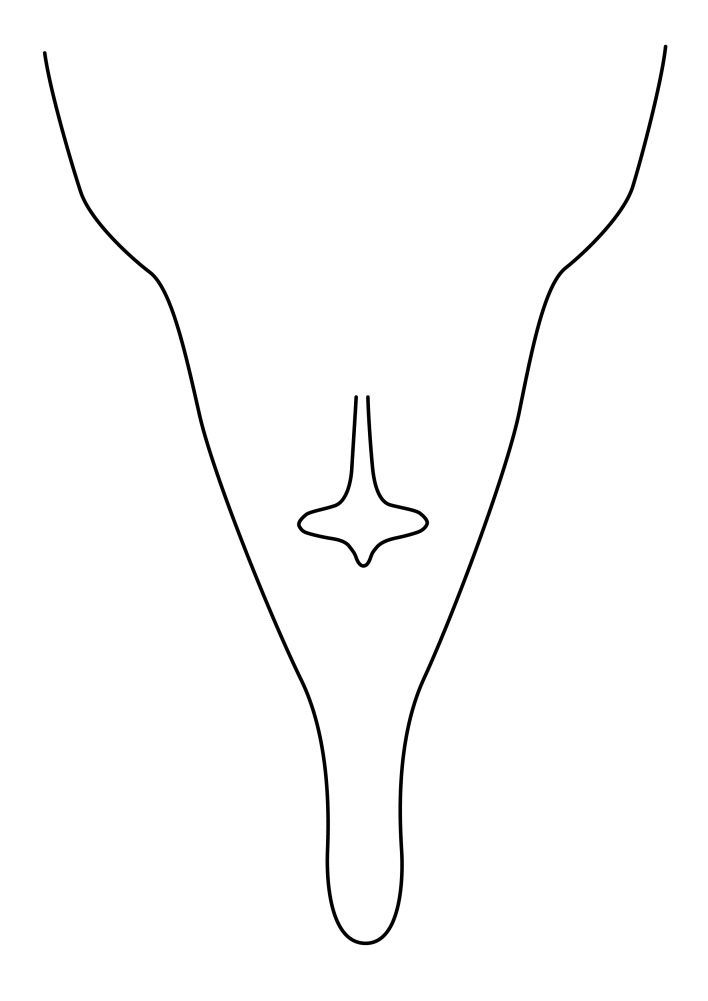
Female gonapophysis IX and spermathecal eminence in ventral view.

**Figure 38a.**
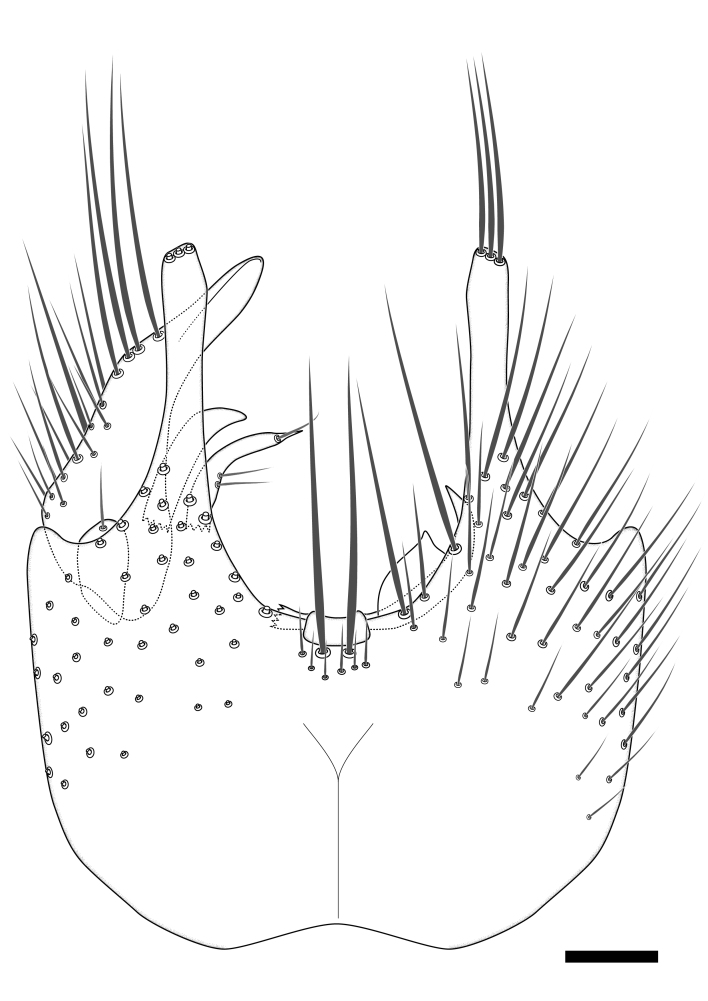
Male terminalia ventral view. Left gonocoxal setae and right gonostylus not drawn. Scale = 50 μm.

**Figure 38b.**
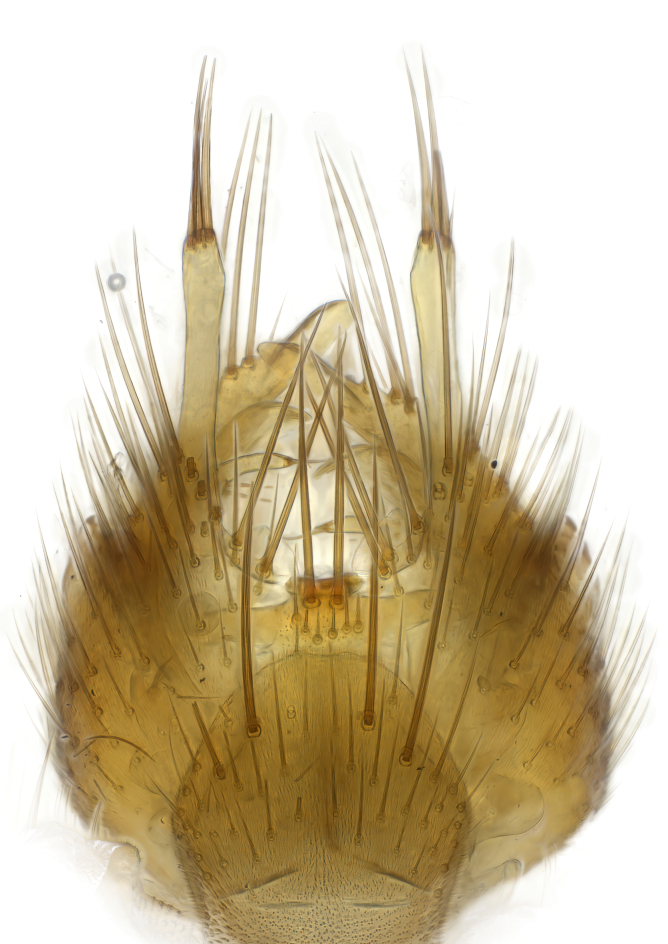
Male terminalia ventral view. Photo.

**Figure 38c.**
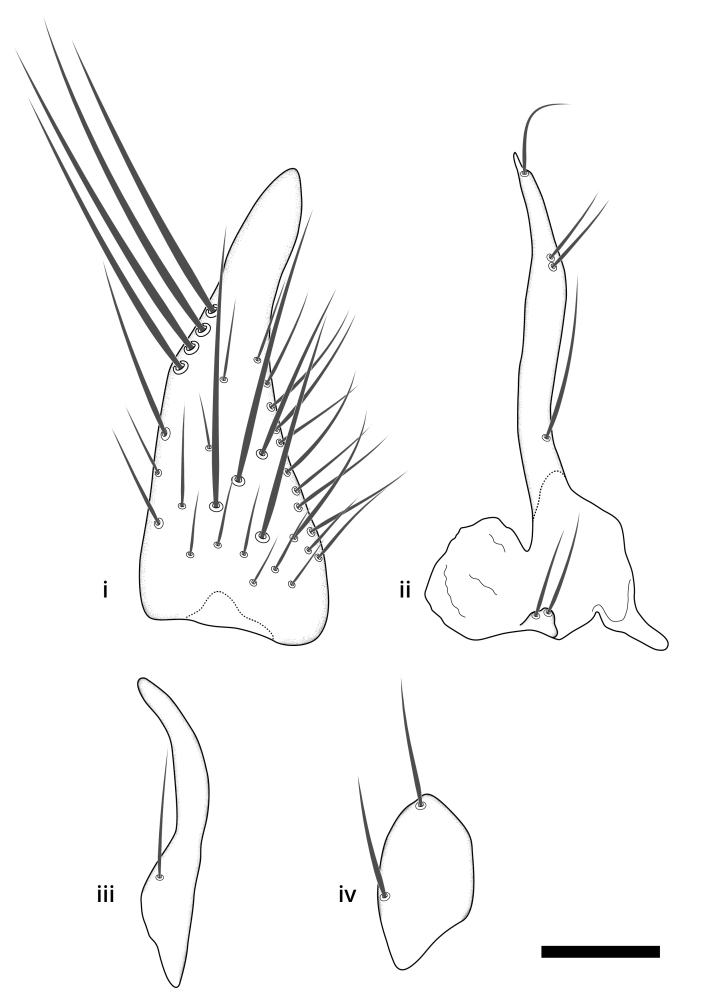
Male right gonostylus with: **i** dorsal branch, **ii** internal branch, **iii** medial branch and **iv** ventral branch separated. Scale = 50 μm.

**Figure 38d.**
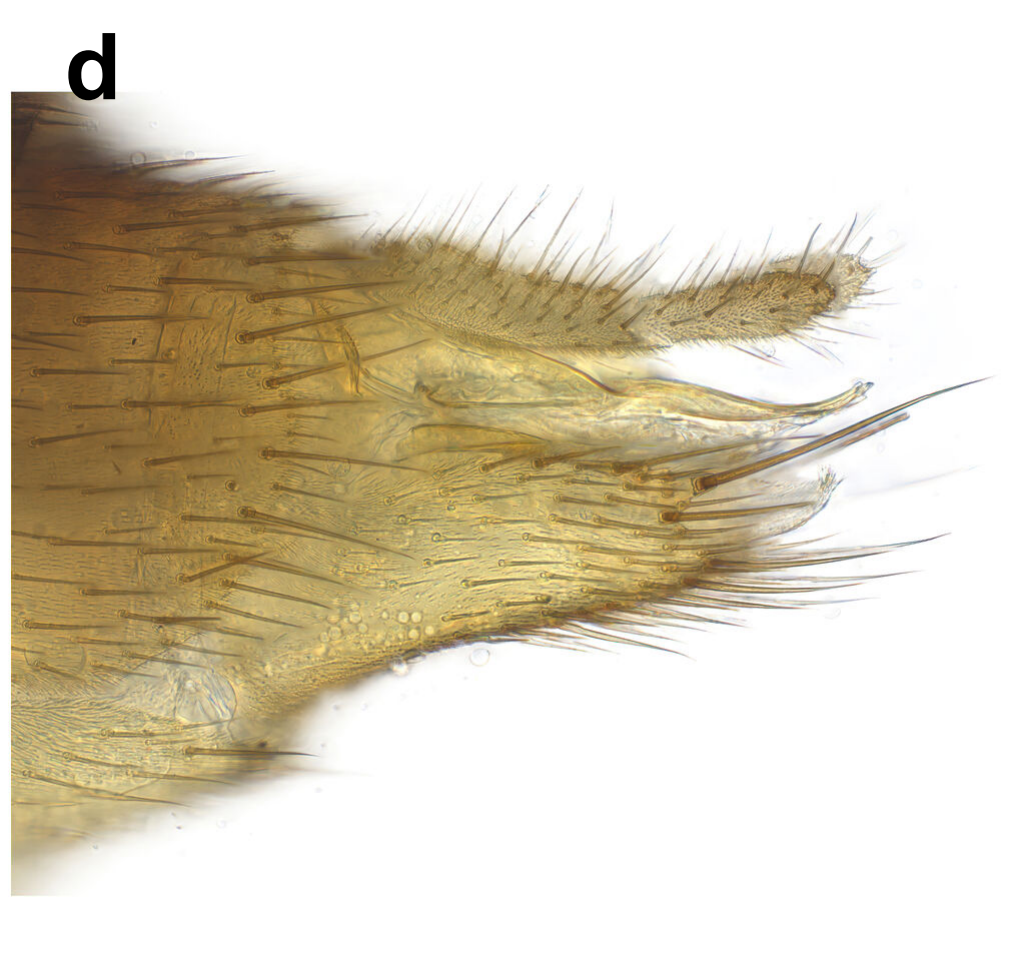
Female terminalia lateral view. Photo.

**Figure 38e.**
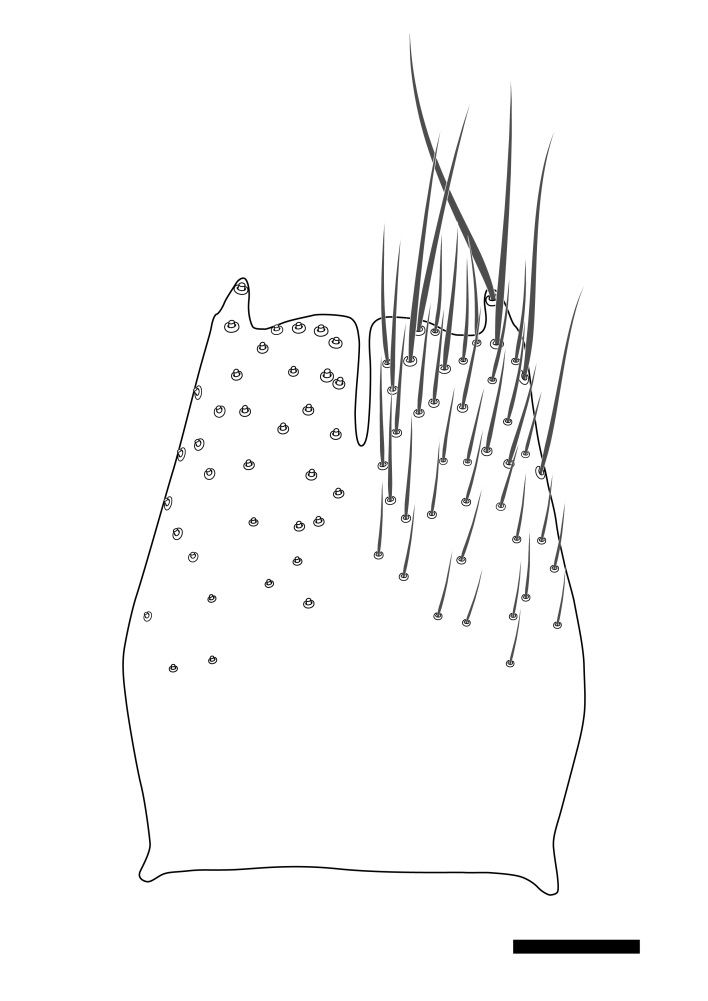
Female sternite VIII ventral view. Setae on left half not drawn. Scale = 50 μm.

**Figure 38f.**
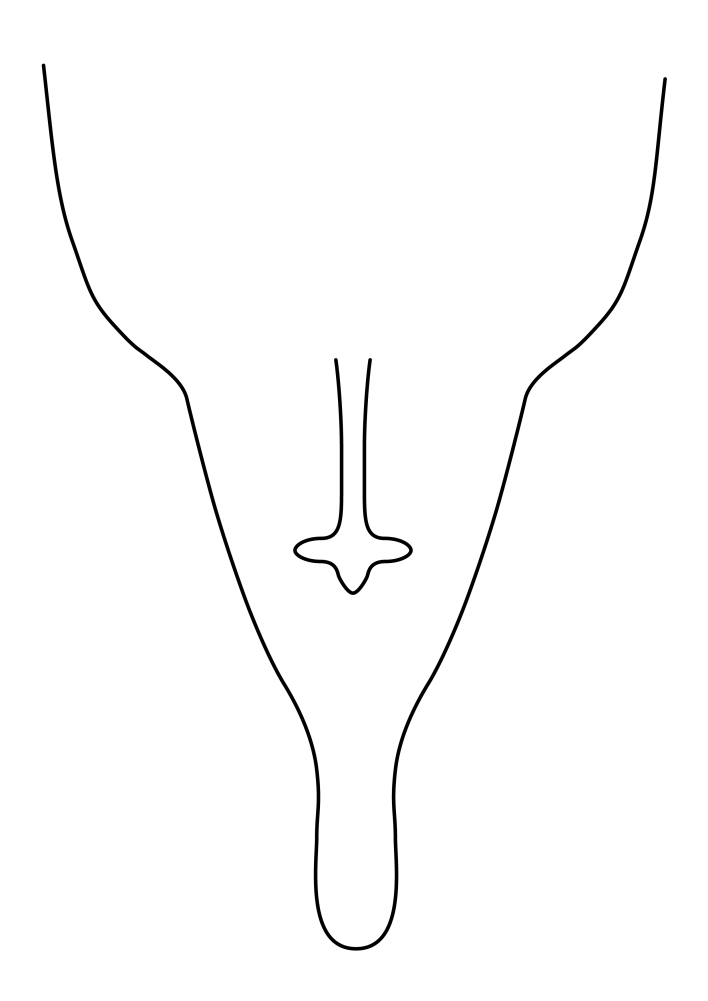
Female gonapophysis IX and spermathecal eminence in ventral view.

**Figure 39a.**
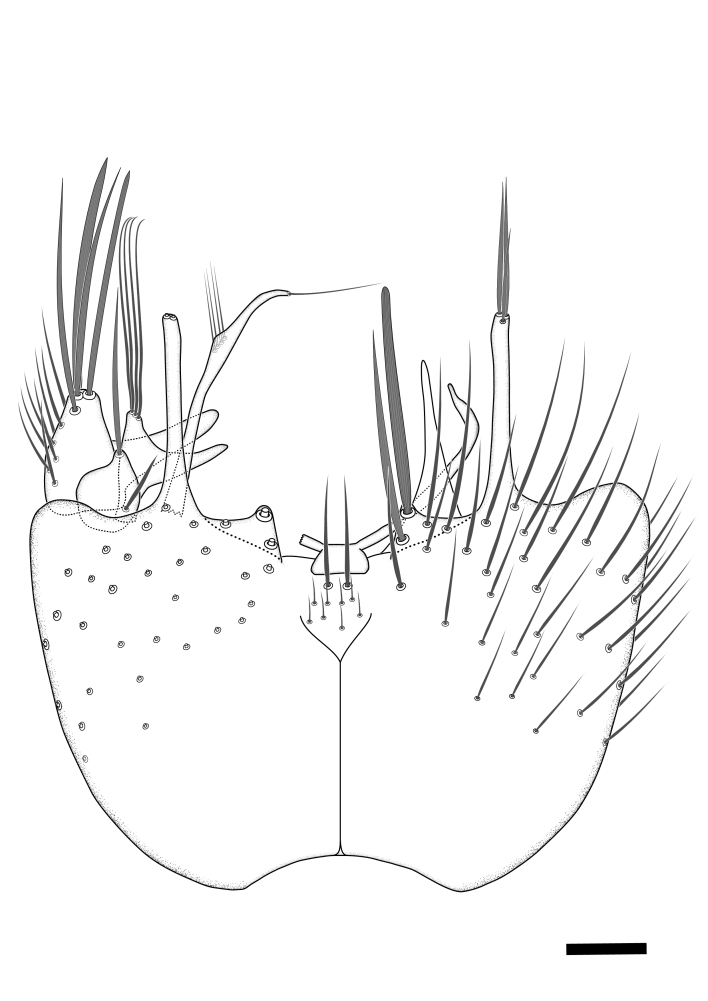
Ventral view. Right gonostylus and setae on left gonocoxite not drawn. Scale = 50 μm.

**Figure 39b.**
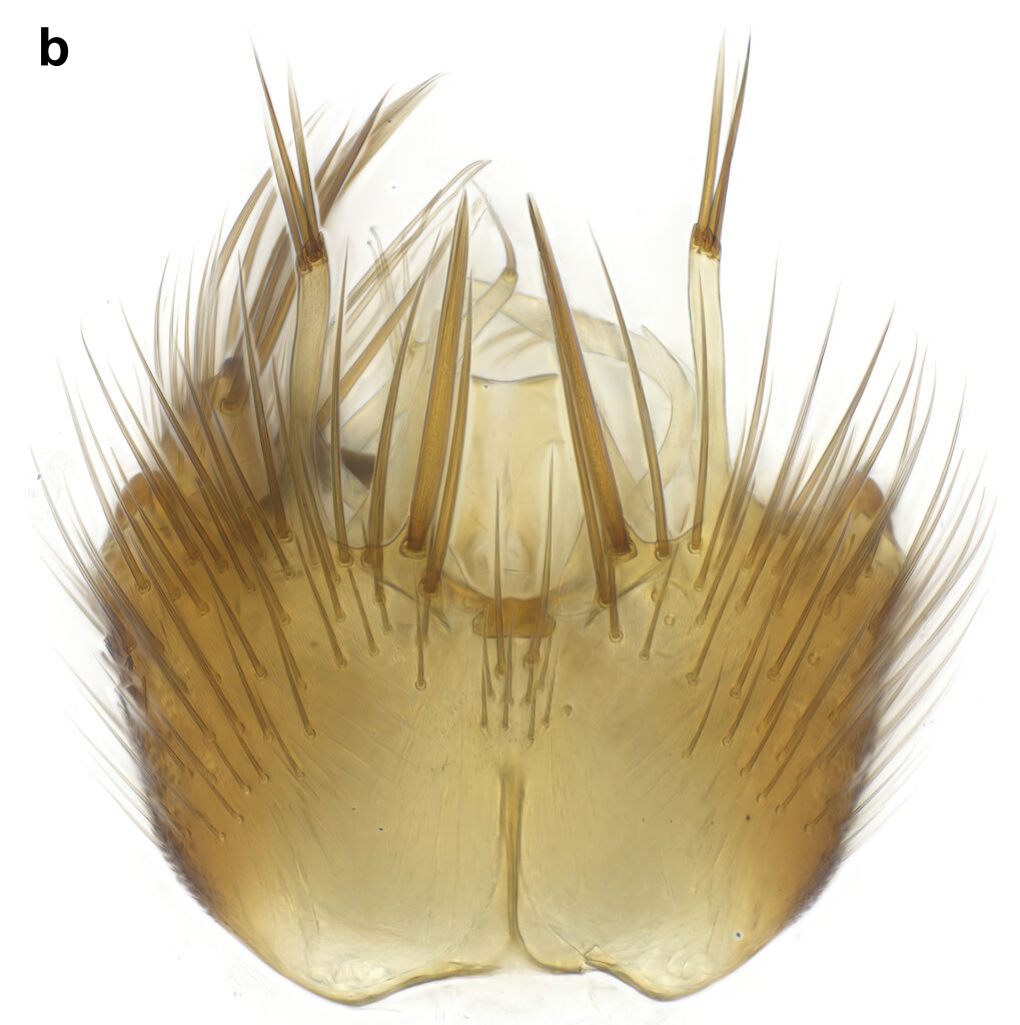
Ventral view. Photo.

**Figure 39c.**
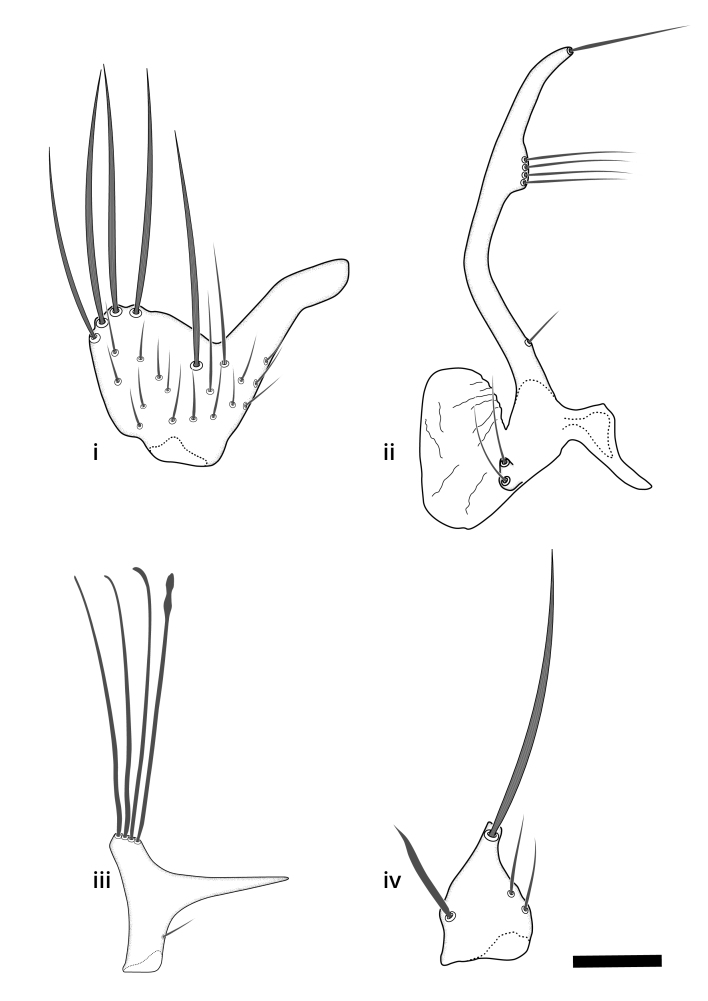
Right gonostylus with: **i** dorsal branch, **ii** internal branch, **iii** medial branch and **iv** ventral branch separated. Scale = 50 μm.

**Figure 40a.**
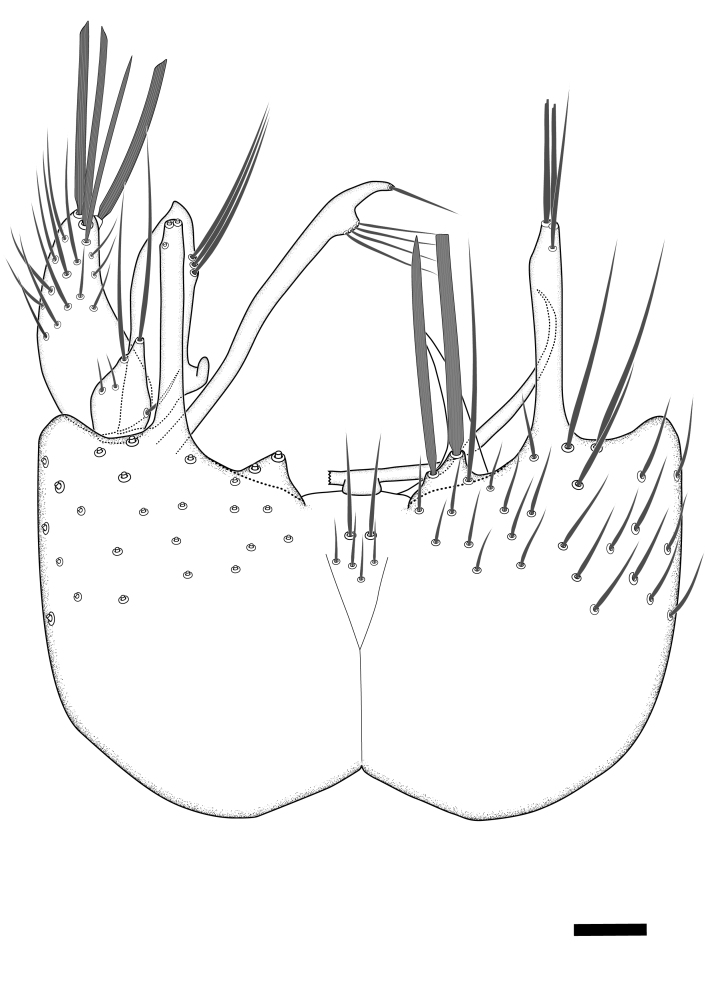
Ventral view. Right gonostylus and setae on left gonocoxite not drawn. Scale = 50 μm.

**Figure 40b.**
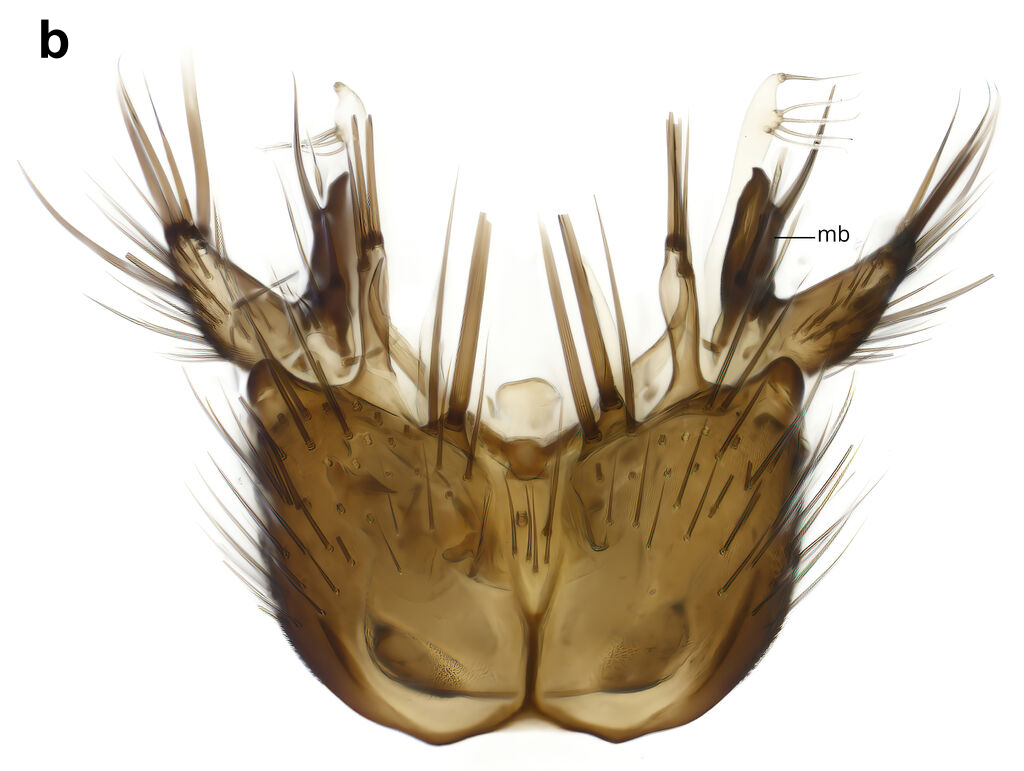
Ventral view. Photo. **Abbreviations**: mb = medial gonostylus branch.

**Figure 40c.**
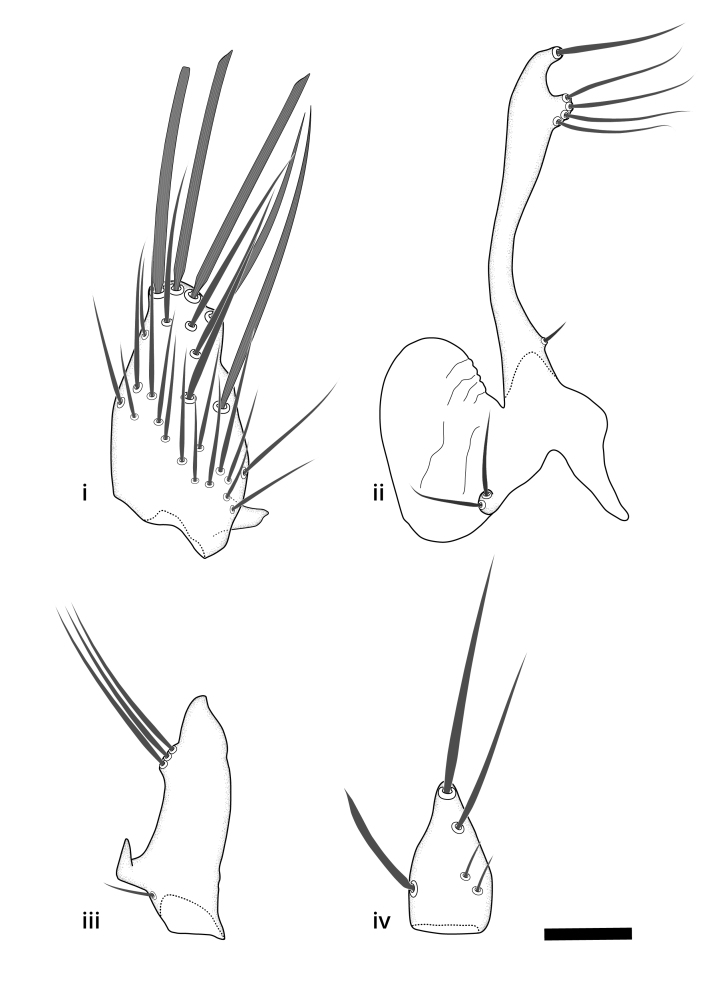
Right gonostylus with: **i** dorsal branch, **ii** internal branch, **iii** medial branch and **iv** ventral branch separated. Scale = 50 μm.

**Figure 41a.**
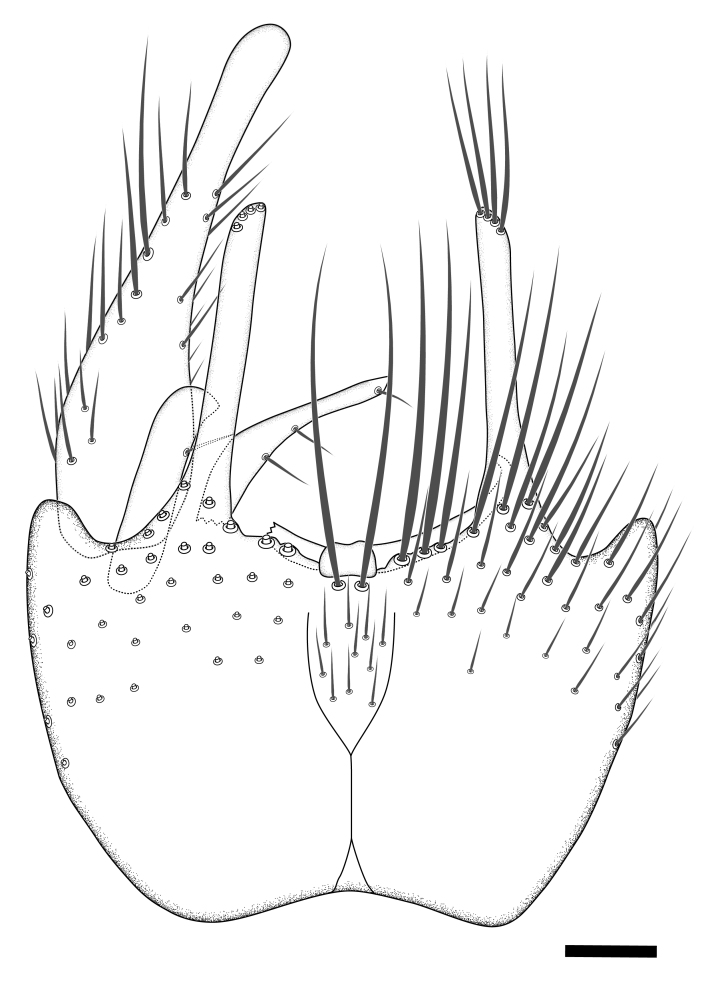
Ventral view. Right gonostylus and setae on left gonocoxite not drawn. Scale = 50 μm.

**Figure 41b.**
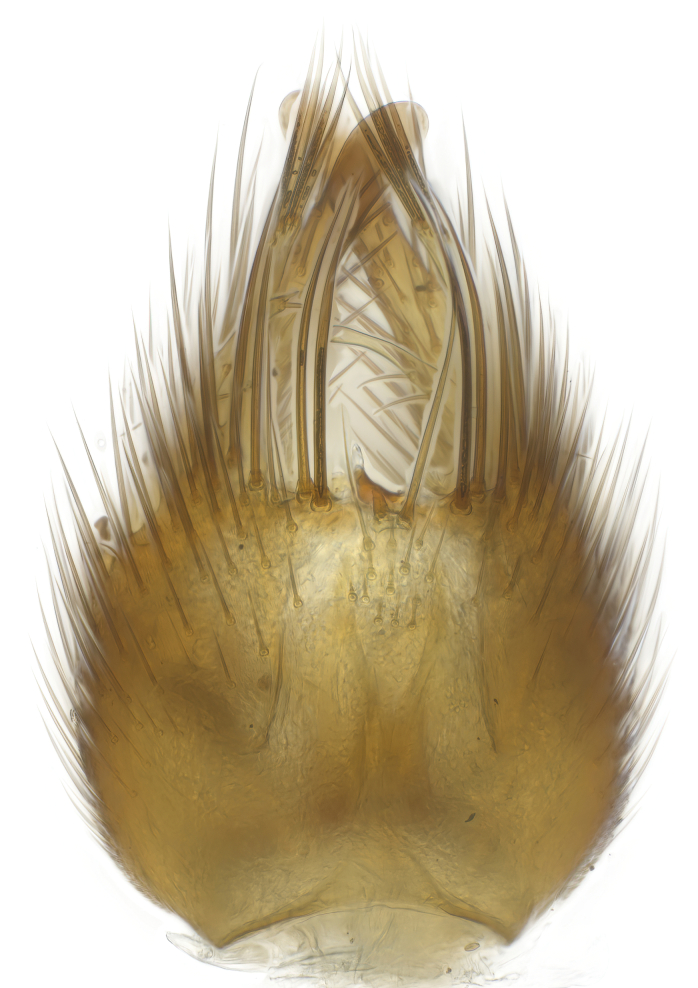
Ventral view. Photo.

**Figure 41c.**
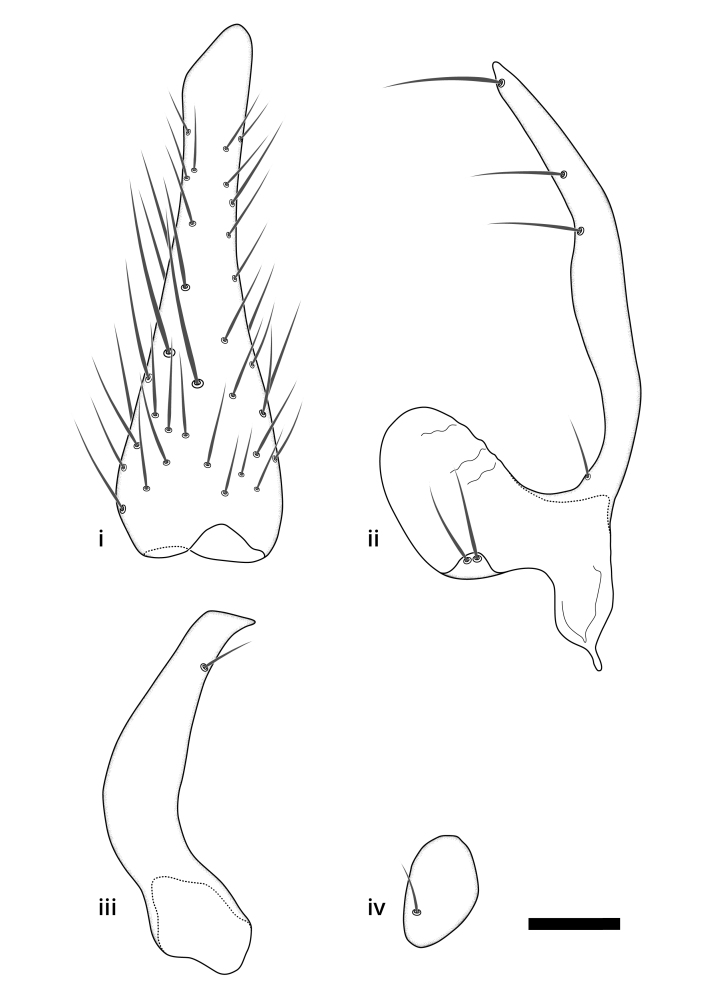
Right gonostylus with: **i** dorsal branch, **ii** internal branch, **iii** medial branch and **iv** ventral branch separated. Scale = 50 μm.
