# Supplementary material for: Revision of the Exechiaparva group (Diptera: Mycetophilidae)
Source: Biodivers Data J. 2021 Sep 24;9:e67134. doi: 10.3897/BDJ.9.e67134 (PMC8486760; doi:10.3897/BDJ.9.e67134)
Supplement: Supplementary material 1 — Table 1 [file bdj-09-e67134-s001.pdf]

Table 1 Mean genetic distances between species in the *E. parva* group calculated from CO1 sequences with a F81 substitution model. Average distance 0.075 ± 2SD [0.15-0.136].

	E. arctata	*E. ashleyi*	*E. breviflagellata*	*E. brevilobata*	*E. burundiensis*	*E. capillata*	*E. curvata*	*E. longilobata*	*E. neorepanda*	*E. parva*	*E. penicillata*	*E. rectiloba*	*E. repanda*	*E. rohdendorfi*	*E. sambai*	*E. spatulata*	*E. sphaerata*	*E. subrepanda*	*E. toyoheii*
E. ashleyi	0.109																		
E. breviflagellata	0.110	0.064																	
E. brevilobata	0.113	0.067	0.012																
E. burundiensis	0.111	0.077	0.080	0.084															
E. capillata	0.110	0.062	0.041	0.037	0.081														
E. curvata	0.118	0.062	0.049	0.052	0.082	0.050													
E. longilobata	0.119	0.058	0.037	0.039	0.082	0.048	0.054												
E. neorepanda	0.118	0.074	0.050	0.049	0.069	0.031	0.059	0.050											
E. parva	0.117	0.062	0.037	0.041	0.085	0.027	0.055	0.042	0.041										
E. penicillata	0.125	0.085	0.080	0.075	0.094	0.069	0.083	0.086	0.085	0.081									
E. rectiloba	0.117	0.067	0.049	0.054	0.087	0.056	0.055	0.049	0.059	0.053	0.090								
E. repanda	0.123	0.078	0.040	0.043	0.094	0.040	0.058	0.052	0.051	0.043	0.077	0.054							
E. rohdendorfi	0.147	0.103	0.108	0.112	0.134	0.115	0.112	0.118	0.127	0.118	0.118	0.117	0.116						
E. sambai	0.118	0.054	0.072	0.074	0.097	0.073	0.080	0.083	0.087	0.067	0.081	0.089	0.081	0.126					
E. spatulata	0.115	0.060	0.033	0.029	0.082	0.024	0.043	0.036	0.033	0.031	0.069	0.036	0.034	0.108	0.075				
E. sphaerata	0.116	0.060	0.034	0.032	0.083	0.029	0.046	0.034	0.038	0.032	0.076	0.040	0.033	0.108	0.078	0.016			
E. subrepanda	0.120	0.064	0.041	0.041	0.084	0.038	0.046	0.043	0.046	0.035	0.084	0.051	0.041	0.120	0.076	0.028	0.025		
E. toyoheii	0.137	0.085	0.096	0.094	0.124	0.088	0.087	0.098	0.084	0.084	0.123	0.092	0.096	0.098	0.111	0.088	0.085	0.092	
E. zuluensis	0.130	0.088	0.084	0.079	0.104	0.080	0.091	0.091	0.091	0.091	0.088	0.101	0.100	0.121	0.094	0.080	0.085	0.093	0.110
