# Supplementary material for: Revision of the Exechiaparva group (Diptera: Mycetophilidae)
Source: Biodivers Data J. 2021 Sep 24;9:e67134. doi: 10.3897/BDJ.9.e67134 (PMC8486760; doi:10.3897/BDJ.9.e67134)
Supplement: Supplementary material 2 — Table 2 [file bdj-09-e67134-s002.pdf]

Table 2 Mean genetic distances within species in the *E. parva* group calculated from CO1 sequences with a F81 substitution model. Average distance 0.005 ± 2SD [0-0.009]. Eight species are only represented by a single specimen and not applicable (NA) for this analysis.

	mean.dist
E. capillata	0.003
E. breviflagellata	0.000
E. sphaerata	0.006
E. curvata	0.010
E. repanda	0.008
E. neorepanda	0.005
E. parva	0.003
E. rectiloba	NA
E. toyoheii	NA
E. rohdendorfi	0.003
E. spatulata	0.002
E. arctata	NA
E. sambai	0.009
E. penicillata	0.003
E. ashleyi	NA
E. subrepanda	0.006
E. longilobata	NA
E. brevilobata	NA
E. zuluensis	NA
E. burundiensis	NA
