# Supplementary figures and images for: Revision of the Exechiaparva group (Diptera: Mycetophilidae)
Source: Biodivers Data J. 2021 Sep 24;9:e67134. doi: 10.3897/BDJ.9.e67134 (PMC8486760; doi:10.3897/BDJ.9.e67134)

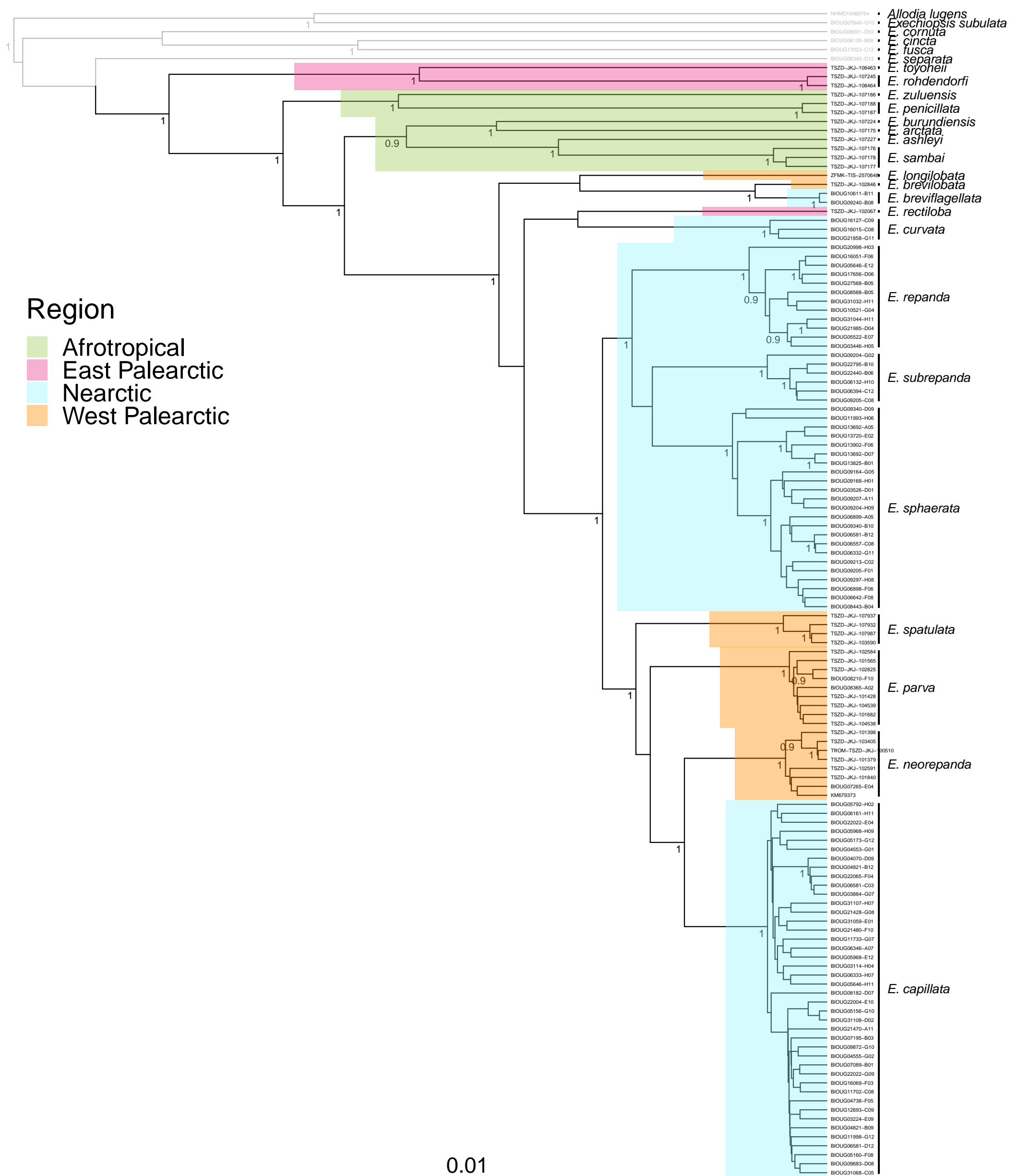

Supplement: Supplementary material 3 — R script [file bdj-09-e67134-s003.zip › jonpeder-The-Exechia-parva-group-b70767c/CO1_tree/co1.tree.pdf]
